# Using inpatient electronic medical records to study influenza for pandemic preparedness

**DOI:** 10.1111/irv.12921

**Published:** 2021-10-25

**Authors:** Candace C. Fuller, Austin Cosgrove, Kenneth Sands, Karla M. Miller, Russell E. Poland, Edward Rosen, Alfred Sorbello, Henry Francis, Robert Orr, Sarah K. Dutcher, Gregory T. Measer, Noelle M. Cocoros

**Affiliations:** ^1^ Department of Population Medicine Harvard Medical School and Harvard Pilgrim Health Care Institute Boston Massachusetts USA; ^2^ HCA Healthcare Nashville Tennessee USA; ^3^ United States Food and Drug Administration Silver Spring Maryland USA; ^4^ At the time of the project, Gregory Measer was with the United States Food and Drug Administration Silver Spring Maryland USA

**Keywords:** electronic medical records, influenza hospitalizations, public health surveillance

## Abstract

**Background:**

We assessed the ability to identify key data relevant to influenza and other respiratory virus surveillance in a large‐scale US‐based hospital electronic medical record (EMR) dataset using seasonal influenza as a use case. We describe characteristics and outcomes of hospitalized influenza cases across three seasons.

**Methods:**

We identified patients with an influenza diagnosis between March 2017 and March 2020 in 140 US hospitals as part of the US FDA's Sentinel System. We calculated descriptive statistics on the presence of high‐risk conditions, influenza antiviral administrations, and severity endpoints.

**Results:**

Among 5.1 million hospitalizations, we identified 29,520 hospitalizations with an influenza diagnosis; 64% were treated with an influenza antiviral within 2 days of admission, and 25% were treated >2 days after admission. Patients treated >2 days after admission had more comorbidities than patients treated within 2 days of admission. Patients never treated during hospitalization had more documentation of cardiovascular and other diseases than treated patients. We observed more severe endpoints in patients never treated (death = 3%, mechanical ventilation [MV] = 9%, intensive care unit [ICU] = 26%) or patients treated >2 days after admission (death = 2%, MV = 14%, ICU = 32%) than in patients treated earlier (treated on admission: death = 1%, MV = 5%, ICU = 23%, treated within 2 days of admission: death = 1%, MV = 7%, ICU = 27%).

**Conclusions:**

We identified important trends in influenza severity related to treatment timing in a large inpatient dataset, laying the groundwork for the use of this and other inpatient EMR data for influenza and other respiratory virus surveillance.

## 1 INTRODUCTION

The US Food and Drug Administration (FDA) has been exploring the feasibility of utilizing real world data, such as administrative claims and electronic medical records (EMR), to support decision making before or during a public health emergency.^1^ Administrative claims provide information regarding exposure to medication dispensings and many outcomes, but use of these databases during a public health emergency can be limited by the time it takes for these databases to “settle.”^2^ In addition, there is often limited ability to assess detailed information regarding inpatient medication use and care received during hospitalization in claims databases. EMR data can provide timely and detailed clinical information, and the feasibility of conducting public health surveillance with EMRs has previously been demonstrated.^3^, ^4^, ^5^, ^6^, ^7^, ^8^, ^9^ However, capturing and analyzing these data in real‐time during a public health emergency are a challenge unless systems are already in place. Simonsen and colleagues have highlighted the use of seasonal influenza as an example infection when developing systems built on “big data” for infectious disease surveillance.^10^

As part of the FDA's Sentinel System,^11^, ^12^ we explored the feasibility of utilizing inpatient EMR data for collecting and analyzing treatments and outcomes in hospitalized patients to support the FDA's need for timely information during a public health emergency.^13^ Sentinel is an active surveillance system that uses routinely collected electronic healthcare data to support FDA's regulatory decision making. Our objective was to assess the ability to identify medications, severity, and other key data relevant to seasonal and pandemic respiratory virus activity in hospitalized patients, using seasonal influenza as a use case. We describe the baseline characteristics, healthcare utilization, complications, and endpoints of hospitalized adults with an influenza diagnosis from March 2017 through March 2020, using data on discharged patients.

## 2 METHODS

### 2.1 Data sources and study population

This was a retrospective descriptive study among adults hospitalized from March 1, 2017, through March 31, 2020. We leveraged an existing EMR dataset from HCA Healthcare for our study which includes 140 hospitals and is updated frequently to support a pragmatic trial at HCA Healthcare.^14^, ^15^ This Sentinel System study was a public health surveillance activity conducted under the authority of the FDA and, accordingly, was not subject to Institutional Review Board oversight.^16^, ^17^, ^18^ March 2017 was selected as the study start as this is when the hospitals systematically began providing medication administration data. Please see Appendix S1for the study design diagram. Within this period, we identified hospitalizations with an influenza diagnosis (via International Classification of Diseases, Tenth Revision, Clinical Modification codes [ICD‐10‐CM]; see Appendix S2 for code lists). We included data on patients discharged and with complete billing only and did not include hospitalizations for patients still admitted or not completely coded when the datasets were created. For reference, we also examined characteristics of all hospitalizations captured in the database.

### 2.2 Demographics, high‐risk conditions, and treatments

We assessed demographics (age, sex, and race) on the date of hospital admission. We examined conditions that may increase a person's risk of serious complications from influenza, including chronic respiratory disease (asthma, chronic obstructive pulmonary disease [COPD], and other chronic respiratory disorders), chronic cardiovascular disease, liver or renal disorders, immune disorders, diabetes, obesity, hematological disorders, and smoking. These were identified via ICD‐10‐CM diagnosis codes documented throughout the hospitalization. We also assessed pregnancy status via diagnosis and procedure codes that are markers for pregnancy as well as those for gestational age recorded at any point during the hospitalization (see Appendix S2 for code lists.)

We identified influenza antiviral treatment administration (or lack thereof) on the day of admission, within 2 days of admission, and beyond 2 days of admission (oseltamivir, zanamivir, peramivir, baloxavir). We also examined antibiotic use during hospitalization. We used brand names, generic names, National Drug Codes (NDC), and Healthcare Common Procedure Coding System (HCPCS) procedure codes to define treatments. Please see Appendix S3 for the medication search strategy and relevant procedure codes.

### 2.3 Complications

We looked for complications coded during the hospital stay and examined those complications by whether they were coded as present on admission or after admission. Complications included pulmonary complications, inflammatory conditions, myocardial infarction, stroke, and sepsis (see Appendix S2 for code lists). We also examined death in the hospital (i.e., discharged expired).

To describe markers of illness severity we examined intensive care unit (ICU) stays, use of supplemental oxygen, bilevel positive airway pressure (BiPAP), mechanical ventilation (MV), and extracorporeal membrane oxygenation (ECMO) (see Appendix S2 for code lists). Length of stay was calculated based on admission and discharge dates.

### 2.4 Ordinal endpoints

We assessed select complications that are associated with severe influenza, and other respiratory infections, and may be of interest in future studies of MCM safety and effectiveness as ordinal endpoints. Ordinal endpoints were as follows: (1) death in hospital; (2) MV or ECMO; (3) ICU care with no MV or ECMO; (4) non‐ICU hospitalization requiring supplemental oxygen; (5) non‐ICU hospitalization with no supplemental oxygen. Although patients might have had evidence of multiple endpoints, each hospitalization was counted under only the most severe (numerically lower) endpoint. We identified ICU stays with revenue codes (see Appendix S2 for codes), and death in hospital with discharge disposition.

## 3 RESULTS

**FIGURE 1:**
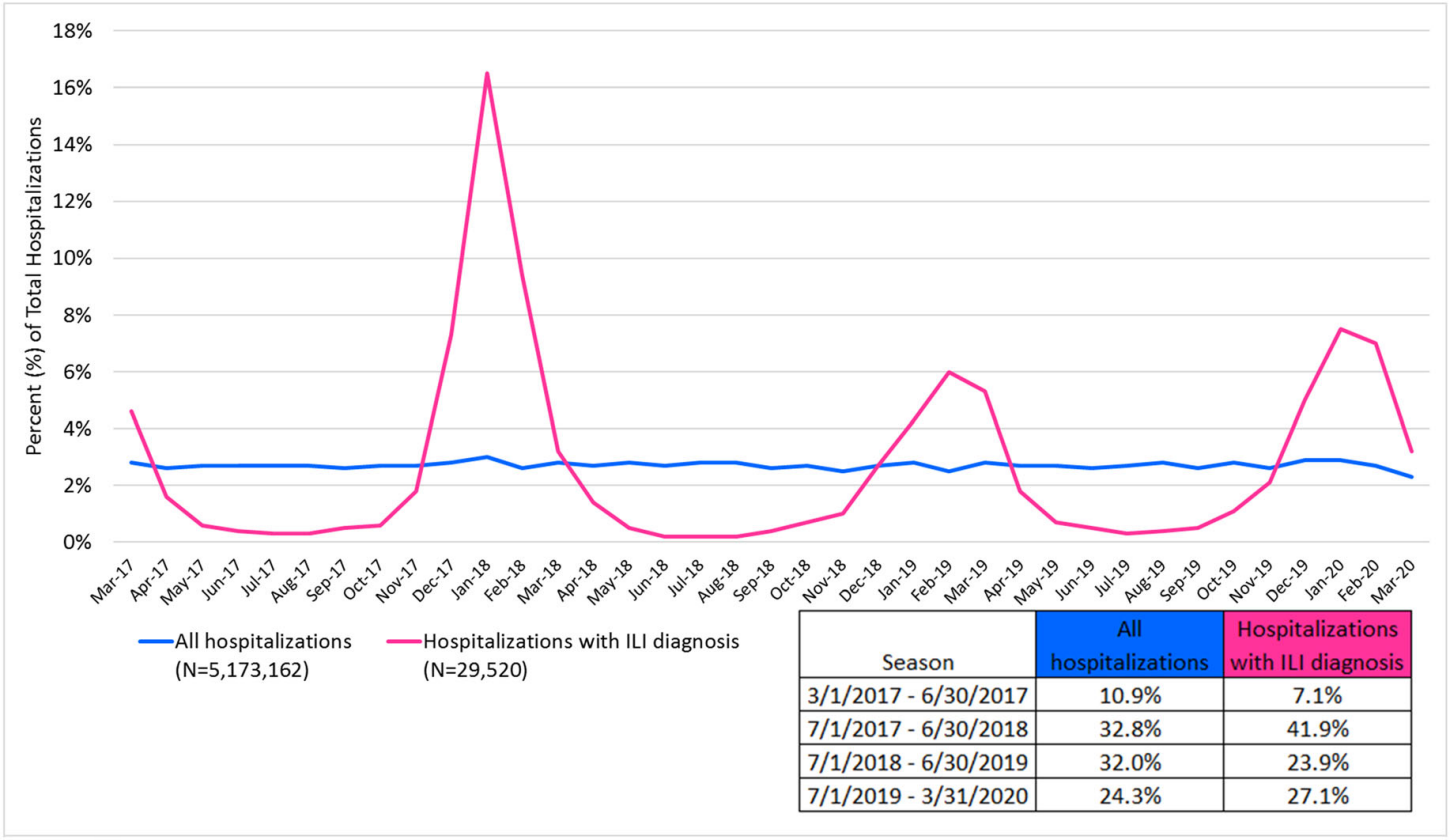
Proportion of hospitalizations with influenza diagnoses and of all inpatient hospitalizations by calendar month and year between March 1, 2017 and March 31, 2020

Among approximately 5.1 million hospitalizations, we identified 29,520 hospitalizations with an influenza diagnosis (*n* = 28,791 patients) between March 1, 2017, and March 31, 2020. Figure 1 examines hospitalizations by month; we observed expected time trends with respect to influenza seasonality.

### 3.1 Baseline characteristics of hospitalized patients with influenza diagnosis

Table 1 summarizes the number of hospitalizations with an influenza diagnosis, demographic characteristics, and high‐risk conditions captured during the hospital stay stratified by timing of influenza antiviral treatment. Among the influenza hospitalizations, there were more females than males (58%), and more than half of the patients were over the age of 65 years (56%) on admission. Race information was well captured with just slightly over 1% of hospitalizations missing race information.

**TABLE 1:** Baseline characteristics of hospitalized patients with an influenza diagnosis stratified by timing of antiviral treatment between March 1, 2017 and March 31, 2020

	All hospitalizations (*N* = 5,173,162)	Hospitalizations with influenza diagnosis (*N* = 29,520)	Hospitalizations with influenza, treated on admission date (*N* = 8,466)	Hospitalizations with influenza, treated ≤2 days after admission date (*N* = 10,348)	Hospitalizations with influenza, treated >2 days after admission date (*N* = 6,213)	Hospitalizations with influenza, never treated during hospitalization (*N* = 4,493)
	Mean/%	Mean/%	Mean/%	Mean/%	Mean/%	Mean/%
Demographics						
Mean age/STD	58.2 ± 20.4	64.4 ± 18.6	64.4 ± 18.8	64.3 ± 18.9	67.5 ± 16.8	61.9 ± 19.2
Age: 18–49	33.2%	20.1%	20.6%	21.0%	13.8%	25.6%
Age: 50–64	23.0%	23.7%	23.7%	23.1%	24.6%	23.9%
Age: 65–74	18.9%	22.4%	22.2%	21.8%	24.0%	21.6%
Age: 75+	24.9%	33.8%	33.5%	34.1%	37.5%	28.8%
Sex: Female	57.4%	57.6%	58.1%	57.3%	56.9%	58.3%
Sex: Male	42.6%	42.3%	41.7%	42.6%	43.0%	41.5%
Sex: Unknown	0.1%	0.1%	0.1%	0.1%	0.1%	0.2%
Race: White	70.9%	70.1%	69.1%	69.6%	71.2%	71.5%
Race: Black	15.3%	16.5%	17.5%	16.6%	16.0%	14.9%
Race: American Indian/Alaska Native	0.1%	0.1%	0.1%	0.1%	0.1%	0.2%
Race: Other	12.1%	12.2%	12.6%	12.7%	11.4%	11.7%
Race: Unknown	1.6%	1.1%	0.7%	1.0%	1.2%	1.7%
Length of stay						
Mean length of stay/STD	5.6 ± 5.8	5.8 ± 5.6	4.1 ± 3.3	4.9 ± 4.0	9.2 ± 7.6	6.4 ± 6.9
Elixhauser comorbidity index						
Mean score/STD	4.6 ± 8.8	7.7 ± 8.7	6.9 ± 8.3	7.2 ± 8.3	9.2 ± 9.4	8.0 ± 9.4
Mean count of comorbidities/STD	2.8 ± 2.1	3.4 ± 2.0	3.2 ± 1.9	3.3 ± 1.9	3.9 ± 2.1	3.5 ± 2.2
Principal admitting diagnosis						
Influenza principal diagnosis of encounter	0.2%	38.6%	52.2%	43.0%	29.3%	15.7%
High‐risk conditions documented during the stay						
Asthma	6.5%	13.5%	14.6%	14.2%	12.0%	11.7%
Chronic obstructive pulmonary disease	16.9%	32.7%	30.4%	32.6%	37.5%	30.9%
Diabetes	29.7%	36.2%	35.1%	35.7%	39.4%	34.8%
Heart failure	16.6%	21.8%	17.7%	19.8%	29.1%	23.9%
Hematological disorders	39.3%	35.0%	30.2%	32.3%	42.1%	40.5%
Immune disorders: disorders of humoral and cell‐mediated immunity, autoimmune diseases, graft‐versus‐host diseases, disorders of white blood cells, transplant and its complications, or HIV/AIDS	2.6%	6.3%	6.0%	6.2%	6.9%	6.4%
Ischemic heart disease	23.5%	27.8%	24.8%	26.9%	33.1%	28.2%
Liver and renal disorders	28.3%	36.1%	32.6%	34.2%	42.8%	37.5%
Nutritional disorders	7.1%	7.1%	5.3%	5.6%	10.7%	9.2%
Obesity	17.0%	19.6%	19.3%	18.4%	21.7%	19.7%
Other chronic respiratory disorders: cystic fibrosis, tuberculosis, or sarcoidosis	0.3%	0.5%	0.6%	0.4%	0.5%	0.7%
Other heart diseases	33.2%	40.3%	36.5%	38.6%	47.6%	41.1%
Pregnancy (pregnancy marker or gestational age)	11.8%	2.9%	2.9%	3.5%	1.5%	3.4%
Smoking	18.1%	18.4%	17.6%	18.3%	18.3%	20.0%

*Note*: Includes discharges for final billed patients only. The analysis does not include inpatient stays for patients still admitted or not completely coded by the data pull date.

As expected, high‐risk conditions for serious influenza complications were all recorded more frequently in hospitalizations with influenza diagnoses than in all‐cause hospitalizations captured during the study period, including asthma (14% vs. 7%), COPD (33% vs. 17%), diabetes (36% vs. 30%), obesity diagnosis (20% vs. 17%), ischemic heart disease (28% vs. 24%), heart failure (22% vs. 27%), liver or renal disorders (36% vs. 28%), and immune disorders (6% vs. 3%). Similar proportions of smokers were captured in hospitalizations with influenza diagnoses compared with all‐cause hospitalizations (18% vs. 18%). Approximately 3% of patients with an influenza diagnosis had evidence of pregnancy.

Antiviral treatment administrations were recorded in 85% of the influenza hospitalizations; 64% were treated on the admission date or ≤2 days after admission. Patients treated later, >2 days after admission, were older and generally had more high‐risk conditions than those treated earlier. Patients never treated with an influenza antiviral were younger and certain high‐risk conditions such as obesity, ischemic heart disease, heart failure, liver and renal failure, hematological disorders, and smoking were documented more frequently than patients treated early in their hospitalization. Influenza was the principal diagnosis in 52% of hospitalizations treated on the day of admission, 43% treated ≤2 days of admission, 29% treated >2 days of admission, and 16% of hospitalizations that had no evidence of treatment during their stay.

### 3.2 MCMs and oxygen delivery in influenza hospitalizations

Oseltamivir was by far the most commonly administered influenza antiviral medication, representing nearly all influenza antiviral administrations (99%). To examine broader use of other medications, we examined administration of any antibiotic during the hospitalization and found 77% of hospitalizations with influenza diagnosis also had evidence of an antibiotic administration. For oxygen delivery, we were able to identify MV (6% on admission, 6% after admission) and supplemental oxygen use (32% on admission, 51% after admission). We did not identify any BiPAP use with procedure codes and identified three hospitalizations with an influenza diagnosis and ECMO during their stay (Table 2).

**TABLE 2:** Potential influenza complications and oxygen‐related therapy recorded among hospitalizations with influenza diagnosis stratified by timing of antiviral treatment between March 1, 2017 and March 31, 2020

	All hospitalizations (*N* = 5,173,162)	Hospitalizations with influenza diagnosis (*N* = 29,520)	Hospitalizations with influenza, treated on admission date (*N* = 8,466)	Hospitalizations with influenza, treated ≤2 days after admission date (*N* = 10 ,348)	Hospitalizations with influenza, treated >2 days after admission date (*N* = 6,213)	Hospitalizations with influenza, never treated during hospitalization (*N* = 4,493)
	%	%	%	%	%	%
Pulmonary complications: Recorded on admission date						
Acute respiratory distress syndrome (ARDS)	0.1%	0.2%	0.1%	0.2%	0.3%	0.3%
Acute respiratory failure	6.9%	19.6%	18.0%	19.2%	24.4%	16.9%
Chronic respiratory failure	1.0%	1.2%	1.2%	1.2%	1.3%	1.0%
Pneumonia associated with influenza	7.4%	11.6%	9.2%	10.4%	15.9%	13.1%
Any pneumonia	9.2%	13.6%	10.1%	11.9%	19.1%	16.5%
Pulmonary complications: Recorded after admission date						
Acute respiratory distress syndrome (ARDS)	0.0%	0.0%	0.0%	0.0%	0.0%	0.0%
Acute respiratory failure	1.9%	3.2%	2.2%	2.8%	5.0%	3.6%
Chronic respiratory failure	0.0%	0.0%	0.0%	0.0%	0.0%	0.0%
Pneumonia associated with influenza	1.8%	2.1%	1.5%	1.8%	3.1%	2.9%
Any pneumonia	2.4%	2.8%	1.8%	2.3%	4.2%	4.2%
Other complications: Recorded on admission date						
Inflammation of the heart, brain, or muscle tissues: myocarditis, encephalitis, myositis, rhabdomyolysis, bacteremia, or myocardial infarction	17.7%	31.6%	29.7%	31.1%	36.7%	28.9%
Myocardial infarction	3.9%	4.8%	3.3%	4.3%	6.9%	5.6%
Sepsis	9.5%	25.4%	25.4%	25.7%	28.3%	20.9%
Stroke	3.5%	1.5%	1.0%	1.1%	2.3%	2.2%
Other complications: Recorded after admission date						
Inflammation of the heart, brain, or muscle tissues: myocarditis, encephalitis, myositis, rhabdomyolysis, bacteremia, or myocardial infarction	10.8%	16.6%	15.1%	16.1%	20.2%	16.0%
Myocardial infarction	1.5%	1.2%	0.7%	1.1%	1.9%	1.5%
Sepsis	5.2%	13.1%	12.9%	13.0%	14.8%	11.4%
Stroke	4.1%	2.7%	1.8%	2.3%	4.1%	3.2%
Oxygen‐related therapy on admission date						
Bilevel positive airway pressure (BiPAP)	0.0%	0.0%	0.0%	0.0%	0.0%	0.0%
Extracorporeal membrane oxygenation (ECMO)	0.0%	0.0%	0.0%	0.0%	0.0%	0.0%
Mechanical ventilation	4.1%	5.9%	3.9%	4.8%	9.6%	6.7%
Supplemental oxygen	10.5%	32.2%	32.0%	33.9%	34.8%	25.4%
Oxygen‐related therapy recorded after admission date						
Bilevel positive airway pressure (BiPAP)	0.0%	0.0%	0.0%	0.0%	0.0%	0.0%
Extracorporeal membrane oxygenation (ECMO)	0.0%	0.0%	0.0%	0.0%	0.0%	0.0%
Mechanical ventilation	4.9%	6.0%	3.0%	4.6%	11.1%	7.9%
Supplemental oxygen	19.3%	53.1%	50.4%	53.6%	63.4%	43.0%

*Note*: Includes discharges for final billed patients only. The analysis does not include inpatient stays for patients still admitted or not completely coded by the data pull date.

### 3.3 Complications and ordinal endpoints in influenza hospitalizations

Table 2 summarizes complications stratified by timing of influenza antiviral treatment. The most common pulmonary complications among hospitalizations with influenza diagnosis were acute respiratory failure (20% on admission, 3% after admission), any pneumonia (14% on admission, 3% after admission), and pneumonia associated with influenza (12% on admission, 2% after admissions). A higher proportion of patients treated >2 days after admission or never treated had pulmonary complications documented than patients treated earlier. Stroke, sepsis, and myocardial infarction were also more commonly documented in patients treated >2 days after admission or never treated.

Table 3 presents unadjusted rates of the endpoints per 1,000 hospital stays, by timing of influenza antiviral treatment. Overall, we observed lower rates of complications per 1,000 stays in patients treated on admission (any complication: 776, severe complication: 350) or within 2 days of admission (any complication: 779, severe complication: 413) than patients treated >2 days of admission (any complication: 868, severe complication: 563) or never treated with an influenza antiviral during hospitalization (any complication: 737, severe complication: 460). Notably, patients not treated with an influenza antiviral had high unadjusted rates of severe complications such as death and MV, especially in reference to patients treated earlier. The unadjusted rate of any pneumonia was also higher in patients not treated.

**TABLE 3:** Unadjusted rates of complications among hospitalizations with influenza diagnosis per 1,000 stays by timing of antiviral treatment between March 1, 2017 and March 31, 2020

	All hospitalizations (*N* = 5,173,162)	Hospitalizations with influenza diagnosis (*N* = 29,520)	Hospitalizations with influenza, treated on admission date (*N* = 8,466)	Hospitalizations with influenza, treated ≤2 days after admission date (*N* = 10,348)	Hospitalizations with influenza, treated >2 days after admission date (*N* = 6,213)	Hospitalizations with influenza, never treated during hospitalization (*N* = 4,493)
	Rate per 1,000 stays	Rate per 1,000 stays	Rate per 1,000 stays	Rate per 1,000 stays	Rate per 1,000 stays	Rate per 1,000 stays
Any complication^a^	487	797	776	799	868	737
Severe complication^b^	343	427	347	408	554	446
Any pneumonia	101	148	109	128	211	179
Mechanical ventilation	67	90	54	72	155	109
Intensive care unit (ICU) stay^c^	293	349	275	335	462	361
Mortality in hospital	22	17	11	11	23	30

*Note*: Includes discharges for final billed patients only. The analysis does not include inpatient stays for patients still admitted or not completely coded by the data pull date.

^a^ Any complication includes: Pneumonia associated with influenza, acute respiratory failure, chronic respiratory failure, ARDS, supplemental oxygen, BiPAP, mechanical ventilation, ECMO, inflammation of heart, brain, or muscle tissue, myocardial infarction, ischemic stroke, sepsis, ICU stay, or death.

^b^ Severe complication includes the following: pneumonia associated with influenza, mechanical ventilation, ICU stay, or death.

^c^ ICU defined via revenue codes.

**FIGURE 2:**
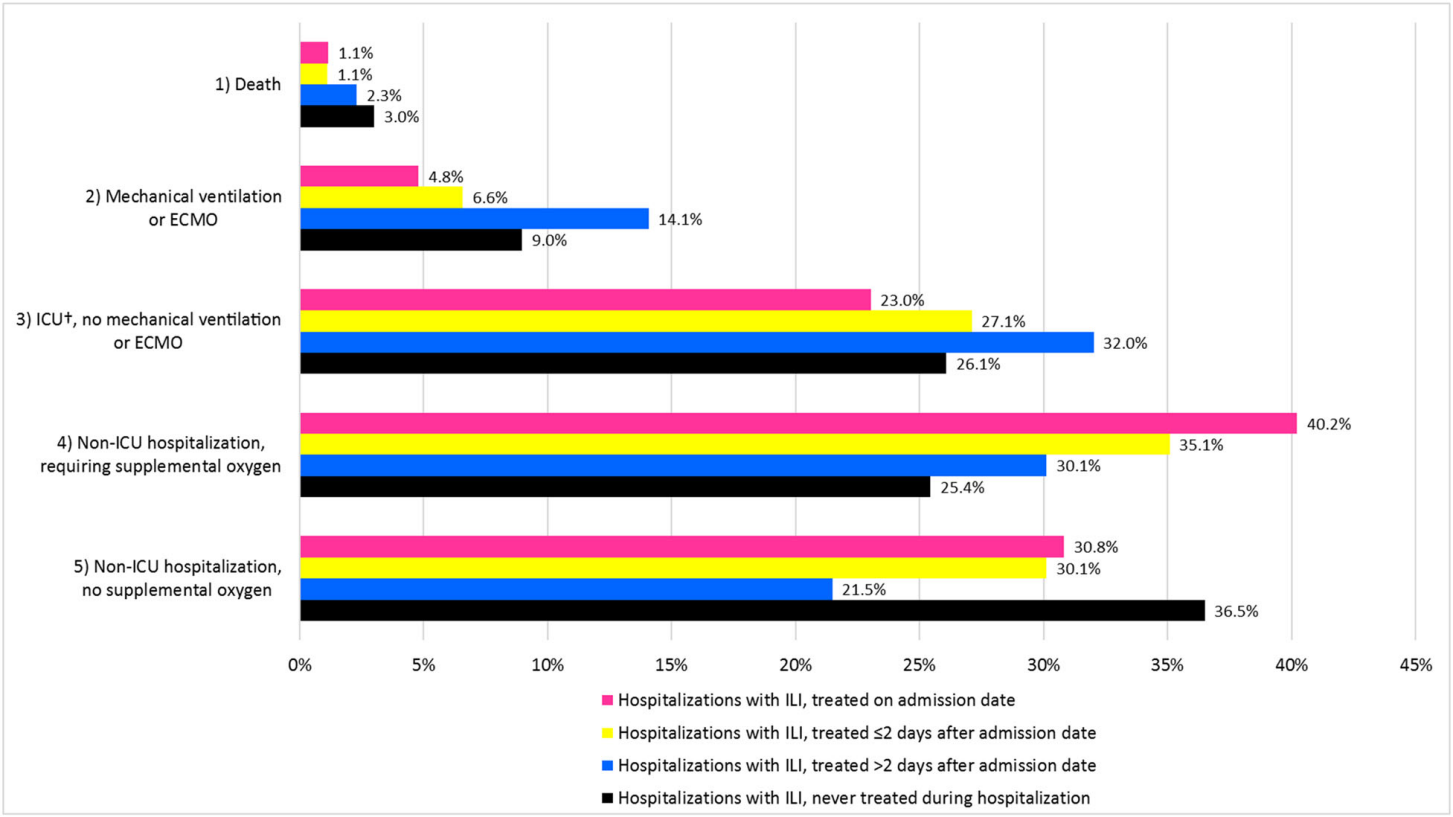
Frequency of a five‐category ordinal endpoint capturing increasing severity of illness among hospitalizations with an influenza diagnosis etween March 1, 2017 and March 31, 2020

Figure 2 shows the frequency of our five‐category ordinal outcomes by treatment timing. We observed more severe ordinal endpoints in patients never treated (death = 3%, MV = 9%, ICU = 26%) or patients treated >2 days after admission (death = 2%, MV = 14%, ICU = 32%) than in patients treated earlier (treated on admission: death = 1%, MV = 5%, ICU = 23%, treated within 2 days of admission: death = 1%, MV = 7%, ICU = 27%). In patients never treated with an antiviral, both the most severe (death) and least severe endpoints (hospitalization not requiring oxygen related or ICU care) were recorded more frequently than among than among patients ever treated during their stay.

## 4 DISCUSSION

### 4.1 Main findings

In this exploratory analysis of influenza‐related data in a large inpatient EMR data source, we identified hospitalizations with influenza diagnoses and administrations of antiviral treatments. As ordinal‐scale endpoints reflecting increasing levels of illness severity have been advocated as useful for evaluating treatment of hospitalized influenza patients^19^, ^20^, ^21^ and during the COVID‐19 pandemic,^22^ we explored the feasibility of capturing these endpoints and reported unadjusted rates to inform future studies. We found that using coded data to capture severe endpoints such as in‐hospital death, ICU stays, and MV during influenza hospitalizations was feasible.

We found the majority (85%) of hospitalizations with an influenza diagnosis had record of an antiviral treatment administration during their stay, and 64% had evidence of treatment within 2 days of admission (29% on admission date, 35% ≤ 2 days following admission). Patients treated >2 days after admission had more comorbidities than patients treated earlier. Similar to another recent study, severe endpoints were lowest among those treated on admission, and highest among patients treated >2 days after admission or not treated during their stay.^23^ The exception was death, which occurred most frequently in patients not treated during their stay.

There was no evidence of influenza antiviral administration in 15% of hospitalizations with influenza diagnosis, and we observed frequent documentation of cardiovascular conditions (e.g., ischemic heart disease and heart failure), obesity, and smoking among these patients. In addition, just 16% of hospitalizations without antiviral administrations had influenza as the principal diagnosis code. It is possible some patients without evidence of antiviral treatment during hospitalization did not truly have influenza, and influenza was a differential diagnosis. However, we were unable to confirm this hypothesis due to the lack of available influenza testing data at the time of this analysis.

The FDA plays a key role in ensuring access to safe and effective medical countermeasures (MCMs; e.g., diagnostic and treatments) during a public health emergency.^1^ Information about MCM safety and effectiveness becomes even more important when an investigational MCM is made available during an emergency. However, capturing and analyzing real‐time information during an emergency remains a challenge. Our study established the capacity for these inpatient EMR data to be used in an emergency while also providing important information about seasonal influenza for future work.

In our study, we were able to capture influenza antiviral treatments along with administration dates and times. This bodes well for future studies using inpatient EMR data to examine medications administered in the hospital. However, it is important that future studies explore medications of interest within their data source. Understanding how medications are captured in data sources used for future studies, and recognizing situations when they may not be completely captured, especially within specific care settings (e.g., intra‐operatively administered medications)^24^ is not a challenge unique to this study. Considerations for ensuring real‐world data are fit for purpose have been commented on previously.^2^, ^25^

We examined oxygen delivery as well as ordinal endpoints in hospitalizations with influenza diagnosis codes. Although we found that up to 40% of influenza non‐ICU hospitalizations had evidence of supplemental oxygen use, we understand that oxygen use may be underestimated if only procedure codes are relied upon.^26^ We were unable to identify BiPAP in this study, which was not unexpected, as billing practices may bundle this with other care and our study identified oxygen delivery based on diagnosis and procedure codes. We expect that both oxygen supplementation and BiPAP use are included in nursing documentation within many EMR systems, and thus it may be possible to extract such information as needed. Future studies in similar inpatient datasets should consider exploring the feasibility of retrieving and analyzing nursing documentation to examine the capture of and ability to attain more specific information regarding type and duration of oxygen therapy.

### 4.2 Strengths and limitations

The major strength of this study was the size of the data source. We identified hospitalizations with influenza diagnoses in an inpatient EMR database that included 140 hospitals and over 5 million inpatient hospitalizations. These data can be refreshed frequently, and we were able to attain data that were updated through March 31, 2020, at the time of our final analyses in late April 2020. The Sentinel System's partnership with HCA Healthcare provides the FDA opportunities to rapidly examine MCM use and other questions of concern during a public health emergency. Although we did not use it extensively in this analysis, the ability to access the rich clinical information collected during a hospitalization such as procedure dates and medication administration dates and times during a hospitalization is often necessary. Inpatient EMR data allow for capture and examination of information not routinely available in other electronic sources such as claims.

We restricted our analysis to only discharged patients with complete billing information. While this means the data were not as “fresh” as possible, it also means the data are complete. Others have asserted that during an evolving public health emergency, information used for decision making should be stable and complete.^2^

There are several details to consider when interpreting our study results. We were unable to examine patient characteristics, medication use, or care delivered before or after the hospitalization and relied on conditions coded during hospitalization to examine baseline and high‐risk conditions. We used diagnosis and procedure codes to examine conditions and procedures and used revenue codes to define ICU stays. We did not have access to laboratory results to confirm influenza diagnosis at the time of this study, although such data are available in this source. Illness onset dates were also not available, a limitation that will be common in claims data sources as well as other EMR data relying on standardized information only (such detail may be captured in notes). Rates of endpoints are unadjusted and not informed by symptom onset. As influenza antivirals are recommended for use within 2 days of symptom onset, this limits conclusions that can be drawn regarding antiviral treatment timing. Patients are often not well tracked across hospitals, and thus our unit of analysis was limited to the individual hospitalization. The study did not include children, but future studies could explore expanding the dataset to include pediatric patients. Finally, the majority of data used in this study were collected prior to the COVD‐19 pandemic beginning in early 2020. Data collected in situations where healthcare systems are strained due to a public health emergency should be interpreted with consideration of circumstances under which clinical care was provided. It is possible that usual coding practices may not be adhered to,^2^ which could influence future analyses using similar data sources during a public health emergency.

## 5 CONCLUSIONS

In conclusion, we found that a large‐scale source of inpatient EMR data within the Sentinel System can provide useful information to FDA on patient care, patient characteristics, and medication use during seasonal influenza, a proxy for other respiratory viruses more broadly. This ability to examine the rich clinical information collected during a hospitalization, including procedure and medication administration dates, has been leveraged for the FDA's COVID‐19 response.^27^ For seasonal influenza, we also report important information on treatment patterns, high‐risk conditions, complications, and ordinal endpoints. Inpatient EMR systems at large can provide important sources of timely information during public health emergencies.

## AUTHOR CONTRIBUTIONS

**Austin Cosgrove:** Data curation; formal analysis; investigation; methodology; visualization. **Kenneth Sands:** Investigation. **Karla M. Miller:** Data curation; investigation; project administration; validation. **Russell E. Poland:** Data curation; formal analysis; investigation; project administration; supervision; validation. **Edward Rosen:** Data curation; formal analysis; investigation; validation; visualization. **Alfred Sorbello:** Conceptualization; investigation; methodology; resources. **Henry Francis:** Conceptualization; funding acquisition; investigation; project administration; resources. **Robert Orr:** Conceptualization; formal analysis; funding acquisition; investigation; project administration; supervision. **Sarah K. Dutcher:** Investigation; methodology; project administration; resources; supervision. **Gregory T Measer:** Conceptualization; funding acquisition; investigation; methodology; project administration. **Noelle M. Cocoros:** Conceptualization; funding acquisition; investigation; methodology; project administration; resources; supervision.

## CONFLICT OF INTEREST

The authors have no conflicts of interest to declare.

## DISCLAIMER

The views expressed in this publication represent those of the author(s) and do not necessarily represent the official views of HCA Healthcare or any of its affiliated entities.

## Supporting information


**Appendix S1.** Pictorial Design Diagram for Assessing Influenza‐Like Illness (ILI) during Inpatient Encounters
**Appendix S2.** List of International Classification of Diseases, Tenth Revision, Clinical Modification (ICD‐10‐CM), International Classification of Diseases, Tenth Revision, Procedure Coding System (ICD‐10‐PCS), Healthcare Common Procedure Coding System (HCPCS), Current Procedural Terminology (CPT), and Revenue Codes Used in the Analysis
**Appendix S3.** List of Generic and Brand Names Used to Define Medical Product Exposures in this Analysis

## References

[1] Measer GT , Maher CT , Hu‐Primmer J . Monitoring and assessment of medical countermeasures as part of a public health emergency response. Am J Public Health. 2018;108(Suppl 3):S224‐S226. 30192659 10.2105/AJPH.2018.304526 PMC6129665

[2] Pottegård A , Kurz X , Moore N , Christiansen CF , Klungel O . Considerations for pharmacoepidemiological analyses in the SARS‐CoV‐2 pandemic. Pharmacoepidemiol Drug Saf. 2020;29(8):825‐831. 32369865 10.1002/pds.5029

[3] Curtis LH , Brown J , Platt R . Four health data networks illustrate the potential for a shared national multipurpose big‐data network. Health Aff Proj Hope. 2014;33(7):1178‐1186. 10.1377/hlthaff.2014.0121 25006144

[4] Newton‐Dame R , McVeigh KH , Schreibstein L , et al. Design of the New York City macroscope: innovations in population health surveillance using electronic health records. EGEMS Wash DC. 2016;4(1):1265. 28154835 10.13063/2327-9214.1265 PMC5226383

[5] Eggleston EM , Weitzman ER . Innovative uses of electronic health records and social media for public health surveillance. Curr Diab Rep. 2014;14(3):468. 24488369 10.1007/s11892-013-0468-7

[6] Klompas M , McVetta J , Lazarus R , et al. Integrating clinical practice and public health surveillance using electronic medical record systems. Am J Public Health. 2012;102(Suppl 3):S325‐S332. 22690967 10.2105/AJPH.2012.300811 PMC3478075

[7] Vogel J , Brown JS , Land T , Platt R , Klompas M . MDPHnet: secure, distributed sharing of electronic health record data for public health surveillance, evaluation, and planning. Am J Public Health. 2014;104(12):2265‐2270. 25322301 10.2105/AJPH.2014.302103 PMC4232140

[8] Klompas M , Cocoros NM , Menchaca JT , et al. State and local chronic disease surveillance using electronic health record systems. Am J Public Health. 2017;107(9):1406‐1412. 28727539 10.2105/AJPH.2017.303874 PMC5551591

[9] Birkhead GS , Klompas M , Shah NR . Uses of electronic health records for public health surveillance to advance public health. Annu Rev Public Health. 2015;36(1):345‐359. 25581157 10.1146/annurev-publhealth-031914-122747

[10] Simonsen L , Gog JR , Olson D , Viboud C . Infectious disease surveillance in the big data era: towards faster and locally relevant systems. J Infect Dis. 2016;214(suppl_4):S380‐S385. 28830112 10.1093/infdis/jiw376 PMC5144901

[11] Platt R , Brown JS , Robb M , et al. The FDA Sentinel initiative—an evolving national resource. N Engl J Med. 2018;379(22):2091‐2093. 30485777 10.1056/NEJMp1809643

[12] Ball R , Robb M , Anderson SA , Dal PG . The FDA's Sentinel initiative—a comprehensive approach to medical product surveillance. Clin Pharmacol Ther. 2016;99(3):265‐268. 26667601 10.1002/cpt.320

[13] Sentinel . Assessing Sentinel System Capability to Collect and Analyze Medical Countermeasure Data for the FDA Office of Counterterrorism and Emerging Threats (OCET) [Internet]. Sentin. Initiat. 2020 [cited 2020 16];Available from: https://www.sentinelinitiative.org/methods-data-tools/methods/assessing-sentinel-system-capability-collect-and-analyze-medical

[14] The INSPIRE‐ASP PNA Trial . ‐ Full Text View ‐ ClinicalTrials.gov [Internet]. [cited 2020 Sep 7];Available from: https://clinicaltrials.gov/ct2/show/NCT03697070

[15] HCA Healthc . Who We Are [Internet]. [cited 2020 Sep 7];Available from: https://hcahealthcare.com/about/

[16] Basic HHS Policy for protection of human research subjects [Internet]. Available from: https://www.hhs.gov/ohrp/regulations-and-policy/regulations/45-cfr-46/index.html 11664145

[17] Federal Policy for the Protection of Human Subjects , 82 Federal Register. 2017. 28106360

[18] Rosati K , Jorgensen N , Soliz M , Evans B . Sentinel initiative principles and policies: HIPAA and Common Rule Compliance in the Sentinel Initiative. [Internet]. 2018. Available from: https://www.sentinelinitiative.org/sites/default/files/communications/publications-presentations/HIPAA-Common-Rule-Compliance-in-Sentinel-Initiative.pdf

[19] King JC , Beigel JH , Ison MG , et al. Clinical development of therapeutic agents for hospitalized patients with influenza: challengees and innovations. Open Forum Infect Dis. 2019;6(4):ofz137. 31037242 10.1093/ofid/ofz137 PMC6479095

[20] Peterson RL , Vock DM , Babiker A , et al. Comparison of an ordinal endpoint to time‐to‐event, longitudinal, and binary endpoints for use in evaluating treatments for severe influenza requiring hospitalization. Contemp Clin Trials Commun. 2019;15:100401. 31312748 10.1016/j.conctc.2019.100401 PMC6609815

[21] King J , Zhou J , Katzen J , et al. Evaluation of a novel ordinal scale endpoint in hospitalized patients with severe influenza illness. Open Forum Infect Dis [Internet] 2016 [cited 2020 Sep 7];3(suppl_1). Available from: https://academic.oup.com/ofid/article/3/suppl_1/638/2637016

[22] W Blueprint Novel Coronavirus. COVID‐19_Treatment_Trial_Design_Master_Protocol_synopsis_Final_18022020.pdf [Internet]. [cited 2020 Sep 7];Available from: https://www.who.int/blueprint/priority-diseases/key-action/COVID-19_Treatment_Trial_Design_Master_Protocol_synopsis_Final_18022020.pdf

[23] Garcia C . 2019 ICPE poster: characteristics and treatment status of those with influenza‐like illness: implications for effectiveness studies and surveillance [Internet]. Sentin. Initiat. 2019 [cited 2020 Nov 5];Available from: https://www.sentinelinitiative.org/news-events/publications-presentations/2019-icpe-poster-characteristics-and-treatment-status-those

[24] Sentinel . 2017 ICPE presentation: curating inpatient medication use data from a hospital network electronic medication administration record (eMAR) system: lessons from the sentinel system about expanding drug safety surveillance potential [Internet]. Sentin. Initiat. 2017 [cited 2020 Sep 7];Available from: https://www.sentinelinitiative.org/news-events/publications-presentations/2017-icpe-presentation-curating-inpatient-medication-use-data

[25] Reynolds MW , Bourke A , Dreyer NA . Considerations when evaluating real‐world data quality in the context of fitness for purpose. Pharmacoepidemiol Drug Saf [Internet] [cited 2020 Oct 16]. Available from:. 2020;29(10):1316‐1318. 10.1002/pds.5010 32374042 PMC7687257

[26] Cocoros NM , Fuller CC , Adimadhyam S , et al. A COVID‐19‐ready public health surveillance system: the FDA's Sentinel System. Pharmacoepidemiol Drug Saf. 2021;30(7):827‐837. 33797815 10.1002/pds.5240 PMC8250843

[27] Sentinel . HCA COVID‐19 activities: monitoring of critical drugs and assessment of natural history of disease [Internet]. Sentin. Initiat. 2020 [cited 2020 Sep 11]; available from: https://www.sentinelinitiative.org/methods-surveillance-tools/methods/hca-covid-19-activities-monitoring-critical-drugs-and-assessment-natural-history

