# Supplementary material for: Using inpatient electronic medical records to study influenza for pandemic preparedness
Source: Influenza Other Respir Viruses. 2021 Oct 25;16(2):265–75. doi: 10.1111/irv.12921 (PMC8818824; doi:10.1111/irv.12921)
Supplement: Supplementary file 1 — Appendix S1. Pictorial Design Diagram for Assessing Influenza‐Like Illness (ILI) during Inpatient Encounters Appendix S2. List of International Classification of Diseases, Tenth Revision, Clinical Modification (ICD‐10‐CM), International Classification of Diseases, Tenth Revision, Procedure Coding System (ICD‐10‐PCS), Healthcare Common Procedure Coding System (HCPCS), Current Procedural Terminology (CPT), and Revenue Codes Used in the Analysis Appendix S3. List of Generic and Brand Names Used to Define Medical Product Exposures in this Analysis [file IRV-16-265-s001.docx]

Supplementary Appendix S1. Pictorial Design Diagram for Assessing Influenza-Like Illness (ILI) during Inpatient Encounters


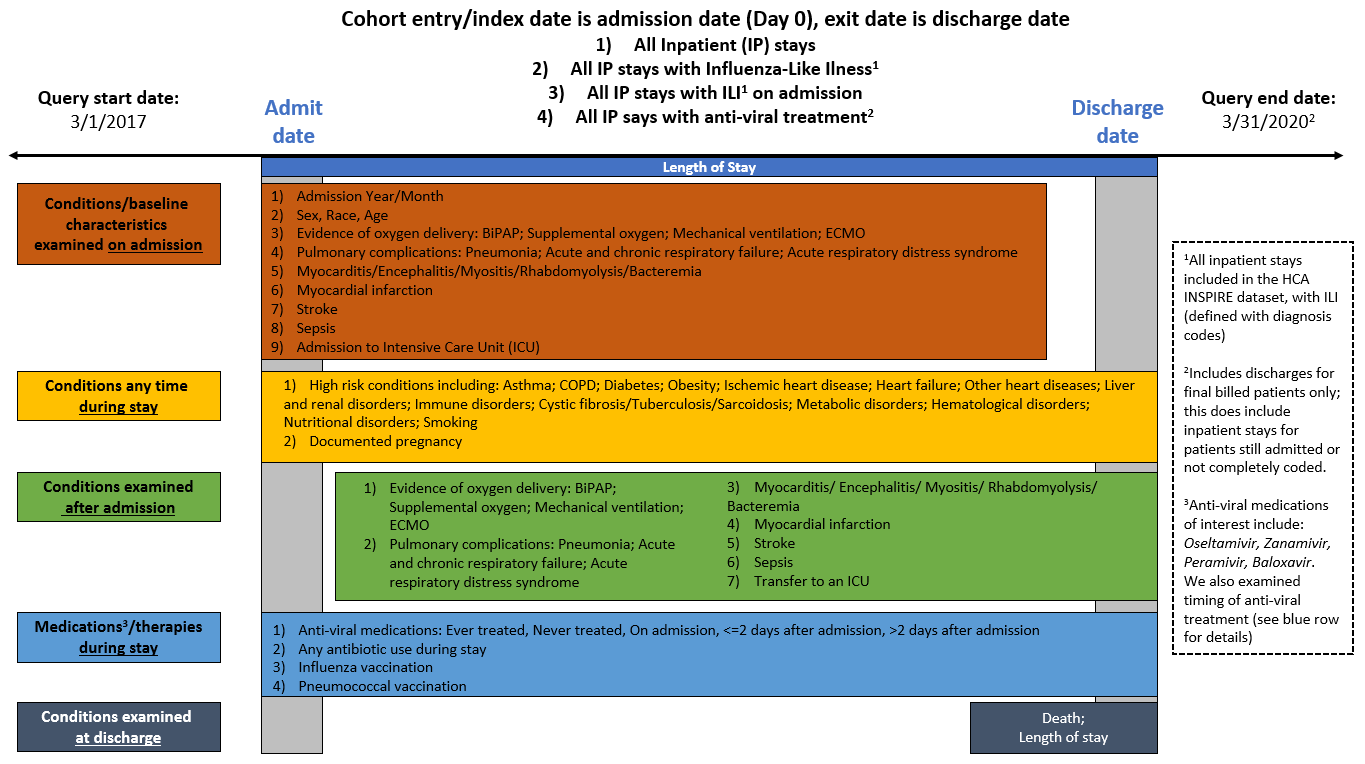


Supplementary Appendix S2. List of International Classification of Diseases, Tenth Revision, Clinical Modification (ICD-10-CM), International Classification of Diseases, Tenth Revision, Procedure Coding System (ICD-10-PCS), Healthcare Common Procedure Coding System (HCPCS), Current Procedural Terminology (CPT), and Revenue Codes Used in the Analysis

| **Code** | **Description** | **Code Category** | **Code Type** |
| --- | --- | --- | --- |
| **Influenza-Like Illness (ILI)** | | | |
| J09.X2 | Influenza due to identified novel influenza A virus with other respiratory manifestations | Diagnosis | ICD-10-CM |
| J09.X3 | Influenza due to identified novel influenza A virus with gastrointestinal manifestations | Diagnosis | ICD-10-CM |
| J09.X9 | Influenza due to identified novel influenza A virus with other manifestations | Diagnosis | ICD-10-CM |
| J10.1 | Influenza due to other identified influenza virus with other respiratory manifestations | Diagnosis | ICD-10-CM |
| J10.2 | Influenza due to other identified influenza virus with gastrointestinal manifestations | Diagnosis | ICD-10-CM |
| J10.81 | Influenza due to other identified influenza virus with encephalopathy | Diagnosis | ICD-10-CM |
| J10.82 | Influenza due to other identified influenza virus with myocarditis | Diagnosis | ICD-10-CM |
| J10.83 | Influenza due to other identified influenza virus with otitis media | Diagnosis | ICD-10-CM |
| J10.89 | Influenza due to other identified influenza virus with other manifestations | Diagnosis | ICD-10-CM |
| J11.1 | Influenza due to unidentified influenza virus with other respiratory manifestations | Diagnosis | ICD-10-CM |
| J11.2 | Influenza due to unidentified influenza virus with gastrointestinal manifestations | Diagnosis | ICD-10-CM |
| J11.81 | Influenza due to unidentified influenza virus with encephalopathy | Diagnosis | ICD-10-CM |
| J11.82 | Influenza due to unidentified influenza virus with myocarditis | Diagnosis | ICD-10-CM |
| J11.83 | Influenza due to unidentified influenza virus with otitis media | Diagnosis | ICD-10-CM |
| J11.89 | Influenza due to unidentified influenza virus with other manifestations | Diagnosis | ICD-10-CM |
| **Supplemental Oxygen** | | | |
| Z99.81 | Dependence on supplemental oxygen | Diagnosis | ICD-10-CM |
| 3E0F7GC | Introduction of Other Therapeutic Substance into Respiratory Tract, Via Natural or Artificial Opening | Procedure | ICD-10-PCS |
| 94640 | Pressurized or nonpressurized inhalation treatment for acute airway obstruction or for sputum induction for diagnostic purposes (eg, with an aerosol generator, nebulizer, metered dose inhaler or intermittent positive pressure breathing [IPPB] device) | Procedure | CPT-4 |
| 94644 | Continuous inhalation treatment with aerosol medication for acute airway obstruction; first hour | Procedure | CPT-4 |
| 94645 | Continuous inhalation treatment with aerosol medication for acute airway obstruction; each additional hour (List separately in addition to code for primary procedure) | Procedure | CPT-4 |
| **Bilevel Positive Airway Pressure (BiPAP)** | | | |
| A7027 | Combination oral/nasal mask, used with continuous positive airway pressure device, each | Procedure | HCPCS |
| A7030 | Full face mask used with positive airway pressure device, each | Procedure | HCPCS |
| A7034 | Nasal interface (mask or cannula type) used with positive airway pressure device, with or without head strap | Procedure | HCPCS |
| A7044 | Oral interface used with positive airway pressure device, each | Procedure | HCPCS |
| A7045 | Exhalation port with or without swivel used with accessories for positive airway devices, replacement only | Procedure | HCPCS |
| A7046 | Water chamber for humidifier, used with positive airway pressure device, replacement, each | Procedure | HCPCS |
| E0470 | Respiratory assist device, bi-level pressure capability, without backup rate feature, used with noninvasive interface, e.g., nasal or facial mask (intermittent assist device with continuous positive airway pressure device) | Procedure | HCPCS |
| E0471 | Respiratory assist device, bi-level pressure capability, with back-up rate feature, used with noninvasive interface, e.g., nasal or facial mask (intermittent assist device with continuous positive airway pressure device) | Procedure | HCPCS |
| E0472 | Respiratory assist device, bi-level pressure capability, with backup rate feature, used with invasive interface, e.g., tracheostomy tube (intermittent assist device with continuous positive airway pressure device) | Procedure | HCPCS |
| E0561 | Humidifier, nonheated, used with positive airway pressure device | Procedure | HCPCS |
| E0562 | Humidifier, heated, used with positive airway pressure device | Procedure | HCPCS |
| **Mechanical Ventilation** | | | |
| 5A09357 | Assistance with Respiratory Ventilation, Less than 24 Consecutive Hours, Continuous Positive Airway Pressure | Procedure | ICD-10-PCS |
| 5A09457 | Assistance with Respiratory Ventilation, 24-96 Consecutive Hours, Continuous Positive Airway Pressure | Procedure | ICD-10-PCS |
| 5A09557 | Assistance with Respiratory Ventilation, Greater than 96 Consecutive Hours, Continuous Positive Airway Pressure | Procedure | ICD-10-PCS |
| 0B717DZ | Dilation of Trachea with Intraluminal Device, Via Natural or Artificial Opening | Procedure | ICD-10-PCS |
| 0B718DZ | Dilation of Trachea with Intraluminal Device, Via Natural or Artificial Opening Endoscopic | Procedure | ICD-10-PCS |
| 0BH072Z | Insertion of Monitoring Device into Tracheobronchial Tree, Via Natural or Artificial Opening | Procedure | ICD-10-PCS |
| 0BH073Z | Insertion of Infusion Device into Tracheobronchial Tree, Via Natural or Artificial Opening | Procedure | ICD-10-PCS |
| 0BH07DZ | Insertion of Intraluminal Device into Tracheobronchial Tree, Via Natural or Artificial Opening | Procedure | ICD-10-PCS |
| 0BH07YZ | Insertion of Other Device into Tracheobronchial Tree, Via Natural or Artificial Opening | Procedure | ICD-10-PCS |
| 0BH172Z | Insertion of Monitoring Device into Trachea, Via Natural or Artificial Opening | Procedure | ICD-10-PCS |
| 0BH17EZ | Insertion of Endotracheal Airway into Trachea, Via Natural or Artificial Opening | Procedure | ICD-10-PCS |
| 0BH17YZ | Insertion of Other Device into Trachea, Via Natural or Artificial Opening | Procedure | ICD-10-PCS |
| 0BH182Z | Insertion of Monitoring Device into Trachea, Via Natural or Artificial Opening Endoscopic | Procedure | ICD-10-PCS |
| 0BH18EZ | Insertion of Endotracheal Airway into Trachea, Via Natural or Artificial Opening Endoscopic | Procedure | ICD-10-PCS |
| 0BH18YZ | Insertion of Other Device into Trachea, Via Natural or Artificial Opening Endoscopic | Procedure | ICD-10-PCS |
| 0BHK72Z | Insertion of Monitoring Device into Right Lung, Via Natural or Artificial Opening | Procedure | ICD-10-PCS |
| 0BHK73Z | Insertion of Infusion Device into Right Lung, Via Natural or Artificial Opening | Procedure | ICD-10-PCS |
| 0BHK7YZ | Insertion of Other Device into Right Lung, Via Natural or Artificial Opening | Procedure | ICD-10-PCS |
| 0BHK82Z | Insertion of Monitoring Device into Right Lung, Via Natural or Artificial Opening Endoscopic | Procedure | ICD-10-PCS |
| 0BHK83Z | Insertion of Infusion Device into Right Lung, Via Natural or Artificial Opening Endoscopic | Procedure | ICD-10-PCS |
| 0BHK8YZ | Insertion of Other Device into Right Lung, Via Natural or Artificial Opening Endoscopic | Procedure | ICD-10-PCS |
| 0BHL72Z | Insertion of Monitoring Device into Left Lung, Via Natural or Artificial Opening | Procedure | ICD-10-PCS |
| 0BHL73Z | Insertion of Infusion Device into Left Lung, Via Natural or Artificial Opening | Procedure | ICD-10-PCS |
| 0BHL7YZ | Insertion of Other Device into Left Lung, Via Natural or Artificial Opening | Procedure | ICD-10-PCS |
| 0BHL82Z | Insertion of Monitoring Device into Left Lung, Via Natural or Artificial Opening Endoscopic | Procedure | ICD-10-PCS |
| 0BHL83Z | Insertion of Infusion Device into Left Lung, Via Natural or Artificial Opening Endoscopic | Procedure | ICD-10-PCS |
| 0BHL8YZ | Insertion of Other Device into Left Lung, Via Natural or Artificial Opening Endoscopic | Procedure | ICD-10-PCS |
| 0DH57BZ | Insertion of Airway into Esophagus, Via Natural or Artificial Opening | Procedure | ICD-10-PCS |
| 0DH58BZ | Insertion of Airway into Esophagus, Via Natural or Artificial Opening Endoscopic | Procedure | ICD-10-PCS |
| 0WHQ73Z | Insertion of Infusion Device into Respiratory Tract, Via Natural or Artificial Opening | Procedure | ICD-10-PCS |
| 0WHQ7YZ | Insertion of Other Device into Respiratory Tract, Via Natural or Artificial Opening | Procedure | ICD-10-PCS |
| 5A1935Z | Respiratory Ventilation, Less than 24 Consecutive Hours | Procedure | ICD-10-PCS |
| 5A1945Z | Respiratory Ventilation, 24-96 Consecutive Hours | Procedure | ICD-10-PCS |
| 5A1955Z | Respiratory Ventilation, Greater than 96 Consecutive Hours | Procedure | ICD-10-PCS |
| 5A09358 | Assistance with Respiratory Ventilation, Less than 24 Consecutive Hours, Intermittent Positive Airway Pressure | Procedure | ICD-10-PCS |
| 5A0955B | Assistance with Respiratory Ventilation, Greater than 96 Consecutive Hours, Intermittent Negative Airway Pressure | Procedure | ICD-10-PCS |
| 5A09558 | Assistance with Respiratory Ventilation, Greater than 96 Consecutive Hours, Intermittent Positive Airway Pressure | Procedure | ICD-10-PCS |
| 5A09559 | Assistance with Respiratory Ventilation, Greater than 96 Consecutive Hours, Continuous Negative Airway Pressure | Procedure | ICD-10-PCS |
| 5A09459 | Assistance with Respiratory Ventilation, 24-96 Consecutive Hours, Continuous Negative Airway Pressure | Procedure | ICD-10-PCS |
| 5A0945B | Assistance with Respiratory Ventilation, 24-96 Consecutive Hours, Intermittent Negative Airway Pressure | Procedure | ICD-10-PCS |
| 5A09458 | Assistance with Respiratory Ventilation, 24-96 Consecutive Hours, Intermittent Positive Airway Pressure | Procedure | ICD-10-PCS |
| 5A0935B | Assistance with Respiratory Ventilation, Less than 24 Consecutive Hours, Intermittent Negative Airway Pressure | Procedure | ICD-10-PCS |
| 5A09359 | Assistance with Respiratory Ventilation, Less than 24 Consecutive Hours, Continuous Negative Airway Pressure | Procedure | ICD-10-PCS |
| 5A0935Z | Assistance with Respiratory Ventilation, Less than 24 Consecutive Hours | Procedure | ICD-10-PCS |
| 5A0955Z | Assistance with Respiratory Ventilation, Greater than 96 Consecutive Hours | Procedure | ICD-10-PCS |
| 5A0945Z | Assistance with Respiratory Ventilation, 24-96 Consecutive Hours | Procedure | ICD-10-PCS |
| 4168F | Patient receiving care in the intensive care unit (ICU) and receiving mechanical ventilation, 24 hours or less (CRIT) | Procedure | CPT Category II |
| 94662 | continuous negative pressure ventilator | Procedure | CPT-4 |
| 94656 | ventilation assist and management, initiation of pressure or volume preset ventilator for assisted or controlled breathing: first day | Procedure | CPT-4 |
| 94657 | ventilation assist and management, initiation of pressure or volume preset ventilator for assisted or controlled breathing: subsequent days | Procedure | CPT-4 |
| 94002 | ventilation assist and management, initiation of pressure or volume preset ventilators for assisted or controlled breathing; hospital inpatient/observation, initial day | Procedure | CPT-4 |
| 94003 | ventilation assist and management, initiation of pressure or volume preset ventilators for assisted or controlled breathing; hospital inpatient/observation, each subsequent day | Procedure | CPT-4 |
| 94004 | Ventilation assist and management, initiation of pressure or volume preset ventilators for assisted or controlled breathing; nursing facility, per day | Procedure | CPT-4 |
| **Extracorporeal Membrane Oxygenation (ECMO)** | | | |
| 5A15223 | Extracorporeal Membrane Oxygenation, Continuous | Procedure | ICD-10-PCS |
| 33988 | Insertion of left heart vent by thoracic incision (eg, sternotomy, thoracotomy) for ECMO/ECLS | Procedure | CPT-4 |
| 33947 | Extracorporeal membrane oxygenation (ECMO)/extracorporeal life support (ECLS) provided by physician; initiation, veno-arterial | Procedure | CPT-4 |
| 33965 | Extracorporeal membrane oxygenation (ECMO)/extracorporeal life support (ECLS) provided by physician; removal of peripheral (arterial and/or venous) cannula(e), percutaneous, birth through 5 years of age | Procedure | CPT-4 |
| 33955 | Extracorporeal membrane oxygenation (ECMO)/extracorporeal life support (ECLS) provided by physician; insertion of central cannula(e) by sternotomy or thoracotomy, birth through 5 years of age | Procedure | CPT-4 |
| 33964 | Extracorporeal membrane oxygenation (ECMO)/extracorporeal life support (ECLS) provided by physician; reposition central cannula(e) by sternotomy or thoracotomy, 6 years and older (includes fluoroscopic guidance, when performed) | Procedure | CPT-4 |
| 33953 | Extracorporeal membrane oxygenation (ECMO)/extracorporeal life support (ECLS) provided by physician; insertion of peripheral (arterial and/or venous) cannula(e), open, birth through 5 years of age | Procedure | CPT-4 |
| 33959 | Extracorporeal membrane oxygenation (ECMO)/extracorporeal life support (ECLS) provided by physician; reposition peripheral (arterial and/or venous) cannula(e), open, birth through 5 years of age (includes fluoroscopic guidance, when performed) | Procedure | CPT-4 |
| 33984 | Extracorporeal membrane oxygenation (ECMO)/extracorporeal life support (ECLS) provided by physician; removal of peripheral (arterial and/or venous) cannula(e), open, 6 years and older | Procedure | CPT-4 |
| 33962 | Extracorporeal membrane oxygenation (ECMO)/extracorporeal life support (ECLS) provided by physician; reposition peripheral (arterial and/or venous) cannula(e), open, 6 years and older (includes fluoroscopic guidance, when performed) | Procedure | CPT-4 |
| 33958 | Extracorporeal membrane oxygenation (ECMO)/extracorporeal life support (ECLS) provided by physician; reposition peripheral (arterial and/or venous) cannula(e), percutaneous, 6 years and older (includes fluoroscopic guidance, when performed) | Procedure | CPT-4 |
| 33986 | Extracorporeal membrane oxygenation (ECMO)/extracorporeal life support (ECLS) provided by physician; removal of central cannula(e) by sternotomy or thoracotomy, 6 years and older | Procedure | CPT-4 |
| 33952 | Extracorporeal membrane oxygenation (ECMO)/extracorporeal life support (ECLS) provided by physician; insertion of peripheral (arterial and/or venous) cannula(e), percutaneous, 6 years and older (includes fluoroscopic guidance, when performed) | Procedure | CPT-4 |
| 33948 | Extracorporeal membrane oxygenation (ECMO)/extracorporeal life support (ECLS) provided by physician; daily management, each day, veno-venous | Procedure | CPT-4 |
| 33989 | Removal of left heart vent by thoracic incision (eg, sternotomy, thoracotomy) for ECMO/ECLS | Procedure | CPT-4 |
| 33946 | Extracorporeal membrane oxygenation (ECMO)/extracorporeal life support (ECLS) provided by physician; initiation, veno-venous | Procedure | CPT-4 |
| 36822 | Insertion of cannula(s) for prolonged extracorporeal circulation for cardiopulmonary insufficiency (ECMO) (separate procedure) | Procedure | CPT-4 |
| 33954 | Extracorporeal membrane oxygenation (ECMO)/extracorporeal life support (ECLS) provided by physician; insertion of peripheral (arterial and/or venous) cannula(e), open, 6 years and older | Procedure | CPT-4 |
| 33951 | Extracorporeal membrane oxygenation (ECMO)/extracorporeal life support (ECLS) provided by physician; insertion of peripheral (arterial and/or venous) cannula(e), percutaneous, birth through 5 years of age (includes fluoroscopic guidance, when performed) | Procedure | CPT-4 |
| 33963 | Extracorporeal membrane oxygenation (ECMO)/extracorporeal life support (ECLS) provided by physician; reposition of central cannula(e) by sternotomy or thoracotomy, birth through 5 years of age (includes fluoroscopic guidance, when performed) | Procedure | CPT-4 |
| 33969 | Extracorporeal membrane oxygenation (ECMO)/extracorporeal life support (ECLS) provided by physician; removal of peripheral (arterial and/or venous) cannula(e), open, birth through 5 years of age | Procedure | CPT-4 |
| 33966 | Extracorporeal membrane oxygenation (ECMO)/extracorporeal life support (ECLS) provided by physician; removal of peripheral (arterial and/or venous) cannula(e), percutaneous, 6 years and older | Procedure | CPT-4 |
| 33987 | Arterial exposure with creation of graft conduit (eg, chimney graft) to facilitate arterial perfusion for ECMO/ECLS (List separately in addition to code for primary procedure) | Procedure | CPT-4 |
| 33957 | Extracorporeal membrane oxygenation (ECMO)/extracorporeal life support (ECLS) provided by physician; reposition peripheral (arterial and/or venous) cannula(e), percutaneous, birth through 5 years of age (includes fluoroscopic guidance, when performed) | Procedure | CPT-4 |
| 33985 | Extracorporeal membrane oxygenation (ECMO)/extracorporeal life support (ECLS) provided by physician; removal of central cannula(e) by sternotomy or thoracotomy, birth through 5 years of age | Procedure | CPT-4 |
| 33949 | Extracorporeal membrane oxygenation (ECMO)/extracorporeal life support (ECLS) provided by physician; daily management, each day, veno-arterial | Procedure | CPT-4 |
| 33956 | Extracorporeal membrane oxygenation (ECMO)/extracorporeal life support (ECLS) provided by physician; insertion of central cannula(e) by sternotomy or thoracotomy, 6 years and older | Procedure | CPT-4 |
| 33960 | Prolonged extracorporeal circulation for cardiopulmonary insufficiency; initial day | Procedure | CPT-4 |
| 33961 | Prolonged extracorporeal circulation for cardiopulmonary insufficiency; each subsequent day | Procedure | CPT-4 |
| **Intensive Care Unit (ICU)** | | | |
| 200 | Intensive care unit, general | Procedure | Revenue |
| 201 | Intensive care unit, surgical | Procedure | Revenue |
| 202 | Intensive care unit, medical | Procedure | Revenue |
| 203 | Intensive care unit, pediatric | Procedure | Revenue |
| 204 | Intensive care unit, psychiatric stay | Procedure | Revenue |
| 206 | Intensive care unit, intermediate | Procedure | Revenue |
| 207 | Intensive care unit, burn care | Procedure | Revenue |
| 208 | Intensive care unit, trauma | Procedure | Revenue |
| 209 | Intensive care unit, other | Procedure | Revenue |
| 210 | Cardiac care unit, general | Procedure | Revenue |
| 211 | Cardiac care unit, myocardial infarction | Procedure | Revenue |
| 212 | Cardiac care unit, pulmonary care | Procedure | Revenue |
| 213 | Cardiac care unit, heart transplant | Procedure | Revenue |
| 214 | Cardiac care unit, intermediate | Procedure | Revenue |
| 219 | Cardiac care unit, other | Procedure | Revenue |
| **Pneumonia Associated with Influenza** | | | |
| J12.89 | Other viral pneumonia | Diagnosis | ICD-10-CM |
| J12.9 | Viral pneumonia, unspecified | Diagnosis | ICD-10-CM |
| J13 | Pneumonia due to Streptococcus pneumoniae | Diagnosis | ICD-10-CM |
| J14 | Pneumonia due to Hemophilus influenzae | Diagnosis | ICD-10-CM |
| J15.0 | Pneumonia due to Klebsiella pneumoniae | Diagnosis | ICD-10-CM |
| J15.1 | Pneumonia due to Pseudomonas | Diagnosis | ICD-10-CM |
| J15.20 | Pneumonia due to staphylococcus, unspecified | Diagnosis | ICD-10-CM |
| J15.211 | Pneumonia due to Methicillin susceptible Staphylococcus aureus | Diagnosis | ICD-10-CM |
| J15.212 | Pneumonia due to Methicillin resistant Staphylococcus aureus | Diagnosis | ICD-10-CM |
| J15.29 | Pneumonia due to other staphylococcus | Diagnosis | ICD-10-CM |
| J15.3 | Pneumonia due to streptococcus, group B | Diagnosis | ICD-10-CM |
| J15.4 | Pneumonia due to other streptococci | Diagnosis | ICD-10-CM |
| J15.6 | Pneumonia due to other Gram-negative bacteria | Diagnosis | ICD-10-CM |
| J15.8 | Pneumonia due to other specified bacteria | Diagnosis | ICD-10-CM |
| J15.9 | Unspecified bacterial pneumonia | Diagnosis | ICD-10-CM |
| J16.0 | Chlamydial pneumonia | Diagnosis | ICD-10-CM |
| J16.8 | Pneumonia due to other specified infectious organisms | Diagnosis | ICD-10-CM |
| J17 | Pneumonia in diseases classified elsewhere | Diagnosis | ICD-10-CM |
| J18.0 | Bronchopneumonia, unspecified organism | Diagnosis | ICD-10-CM |
| J18.1 | Lobar pneumonia, unspecified organism | Diagnosis | ICD-10-CM |
| J18.8 | Other pneumonia, unspecified organism | Diagnosis | ICD-10-CM |
| J18.9 | Pneumonia, unspecified organism | Diagnosis | ICD-10-CM |
| J09.X1 | Influenza due to identified novel influenza A virus with pneumonia | Diagnosis | ICD-10-CM |
| J10.00 | Influenza due to other identified influenza virus with unspecified type of pneumonia | Diagnosis | ICD-10-CM |
| J10.01 | Influenza due to other identified influenza virus with the same other identified influenza virus pneumonia | Diagnosis | ICD-10-CM |
| J10.08 | Influenza due to other identified influenza virus with other specified pneumonia | Diagnosis | ICD-10-CM |
| J11.00 | Influenza due to unidentified influenza virus with unspecified type of pneumonia | Diagnosis | ICD-10-CM |
| J11.08 | Influenza due to unidentified influenza virus with specified pneumonia | Diagnosis | ICD-10-CM |
| J12.9 | Viral pneumonia, unspecified | Diagnosis | ICD-10-CM |
| **Any Pneumonia** | | | |
| A22.1 | Pulmonary anthrax | Diagnosis | ICD-10-CM |
| A37.01 | Whooping cough due to Bordetella pertussis with pneumonia | Diagnosis | ICD-10-CM |
| A37.11 | Whooping cough due to Bordetella parapertussis with pneumonia | Diagnosis | ICD-10-CM |
| A37.81 | Whooping cough due to other Bordetella species with pneumonia | Diagnosis | ICD-10-CM |
| A37.91 | Whooping cough, unspecified species with pneumonia | Diagnosis | ICD-10-CM |
| A48.1 | Legionnaires' disease | Diagnosis | ICD-10-CM |
| B25.0 | Cytomegaloviral pneumonitis | Diagnosis | ICD-10-CM |
| B44.0 | Invasive pulmonary aspergillosis | Diagnosis | ICD-10-CM |
| B77.81 | Ascariasis pneumonia | Diagnosis | ICD-10-CM |
| J12.0 | Adenoviral pneumonia | Diagnosis | ICD-10-CM |
| J12.1 | Respiratory syncytial virus pneumonia | Diagnosis | ICD-10-CM |
| J12.2 | Parainfluenza virus pneumonia | Diagnosis | ICD-10-CM |
| J12.3 | Human metapneumovirus pneumonia | Diagnosis | ICD-10-CM |
| J12.81 | Pneumonia due to SARS-associated coronavirus | Diagnosis | ICD-10-CM |
| J12.89 | Other viral pneumonia | Diagnosis | ICD-10-CM |
| J12.9 | Viral pneumonia, unspecified | Diagnosis | ICD-10-CM |
| J13 | Pneumonia due to Streptococcus pneumoniae | Diagnosis | ICD-10-CM |
| J14 | Pneumonia due to Hemophilus influenzae | Diagnosis | ICD-10-CM |
| J15.0 | Pneumonia due to Klebsiella pneumoniae | Diagnosis | ICD-10-CM |
| J15.1 | Pneumonia due to Pseudomonas | Diagnosis | ICD-10-CM |
| J15.20 | Pneumonia due to staphylococcus, unspecified | Diagnosis | ICD-10-CM |
| J15.211 | Pneumonia due to Methicillin susceptible Staphylococcus aureus | Diagnosis | ICD-10-CM |
| J15.212 | Pneumonia due to Methicillin resistant Staphylococcus aureus | Diagnosis | ICD-10-CM |
| J15.29 | Pneumonia due to other staphylococcus | Diagnosis | ICD-10-CM |
| J15.3 | Pneumonia due to streptococcus, group B | Diagnosis | ICD-10-CM |
| J15.4 | Pneumonia due to other streptococci | Diagnosis | ICD-10-CM |
| J15.5 | Pneumonia due to Escherichia coli | Diagnosis | ICD-10-CM |
| J15.6 | Pneumonia due to other Gram-negative bacteria | Diagnosis | ICD-10-CM |
| J15.7 | Pneumonia due to Mycoplasma pneumoniae | Diagnosis | ICD-10-CM |
| J15.8 | Pneumonia due to other specified bacteria | Diagnosis | ICD-10-CM |
| J15.9 | Unspecified bacterial pneumonia | Diagnosis | ICD-10-CM |
| J16.0 | Chlamydial pneumonia | Diagnosis | ICD-10-CM |
| J16.8 | Pneumonia due to other specified infectious organisms | Diagnosis | ICD-10-CM |
| J17 | Pneumonia in diseases classified elsewhere | Diagnosis | ICD-10-CM |
| J18.0 | Bronchopneumonia, unspecified organism | Diagnosis | ICD-10-CM |
| J18.1 | Lobar pneumonia, unspecified organism | Diagnosis | ICD-10-CM |
| J18.8 | Other pneumonia, unspecified organism | Diagnosis | ICD-10-CM |
| J18.9 | Pneumonia, unspecified organism | Diagnosis | ICD-10-CM |
| J69.0 | Pneumonitis due to inhalation of food and vomit | Diagnosis | ICD-10-CM |
| J69.1 | Pneumonitis due to inhalation of oils and essences | Diagnosis | ICD-10-CM |
| J69.8 | Pneumonitis due to inhalation of other solids and liquids | Diagnosis | ICD-10-CM |
| J95.4 | Chemical pneumonitis due to anesthesia | Diagnosis | ICD-10-CM |
| J95.5 | Postprocedural subglottic stenosis | Diagnosis | ICD-10-CM |
| J95.851 | Ventilator associated pneumonia | Diagnosis | ICD-10-CM |
| J95.859 | Other complication of respirator [ventilator] | Diagnosis | ICD-10-CM |
| J95.88 | Other intraoperative complications of respiratory system, not elsewhere classified | Diagnosis | ICD-10-CM |
| J95.89 | Other postprocedural complications and disorders of respiratory system, not elsewhere classified | Diagnosis | ICD-10-CM |
| J09.X1 | Influenza due to identified novel influenza A virus with pneumonia | Diagnosis | ICD-10-CM |
| J10.00 | Influenza due to other identified influenza virus with unspecified type of pneumonia | Diagnosis | ICD-10-CM |
| J10.01 | Influenza due to other identified influenza virus with the same other identified influenza virus pneumonia | Diagnosis | ICD-10-CM |
| J10.08 | Influenza due to other identified influenza virus with other specified pneumonia | Diagnosis | ICD-10-CM |
| J11.00 | Influenza due to unidentified influenza virus with unspecified type of pneumonia | Diagnosis | ICD-10-CM |
| J11.08 | Influenza due to unidentified influenza virus with specified pneumonia | Diagnosis | ICD-10-CM |
| **Acute Respiratory Failure** | | | |
| J96.00 | Acute respiratory failure, unspecified whether with hypoxia or hypercapnia | Diagnosis | ICD-10-CM |
| J96.01 | Acute respiratory failure with hypoxia | Diagnosis | ICD-10-CM |
| J96.02 | Acute respiratory failure with hypercapnia | Diagnosis | ICD-10-CM |
| J96.90 | Respiratory failure, unspecified, unspecified whether with hypoxia or hypercapnia | Diagnosis | ICD-10-CM |
| J96.91 | Respiratory failure, unspecified with hypoxia | Diagnosis | ICD-10-CM |
| J96.92 | Respiratory failure, unspecified with hypercapnia | Diagnosis | ICD-10-CM |
| **Chronic Respiratory Failure** | | | |
| J96.10 | Chronic respiratory failure, unspecified whether with hypoxia or hypercapnia | Diagnosis | ICD-10-CM |
| J96.11 | Chronic respiratory failure with hypoxia | Diagnosis | ICD-10-CM |
| J96.12 | Chronic respiratory failure with hypercapnia | Diagnosis | ICD-10-CM |
| **Acute Respiratory Distress Syndrome** | | | |
| J80 | Acute respiratory distress syndrome | Diagnosis | ICD-10-CM |
| **Inflammation of the Heart, Brain, or Muscle Tissues** | | | |
| I51.4 | Myocarditis, unspecified | Diagnosis | ICD-10-CM |
| I51.5 | Myocardial degeneration | Diagnosis | ICD-10-CM |
| G04.00 | Acute disseminated encephalitis and encephalomyelitis, unspecified | Diagnosis | ICD-10-CM |
| G04.01 | Postinfectious acute disseminated encephalitis and encephalomyelitis (postinfectious ADEM) | Diagnosis | ICD-10-CM |
| G04.02 | Postimmunization acute disseminated encephalitis, myelitis and encephalomyelitis | Diagnosis | ICD-10-CM |
| G04.30 | Acute necrotizing hemorrhagic encephalopathy, unspecified | Diagnosis | ICD-10-CM |
| G04.31 | Postinfectious acute necrotizing hemorrhagic encephalopathy | Diagnosis | ICD-10-CM |
| G04.32 | Postimmunization acute necrotizing hemorrhagic encephalopathy | Diagnosis | ICD-10-CM |
| G04.39 | Other acute necrotizing hemorrhagic encephalopathy | Diagnosis | ICD-10-CM |
| G04.81 | Other encephalitis and encephalomyelitis | Diagnosis | ICD-10-CM |
| G04.89 | Other myelitis | Diagnosis | ICD-10-CM |
| G04.90 | Encephalitis and encephalomyelitis, unspecified | Diagnosis | ICD-10-CM |
| G04.91 | Myelitis, unspecified | Diagnosis | ICD-10-CM |
| G05.3 | Encephalitis and encephalomyelitis in diseases classified elsewhere | Diagnosis | ICD-10-CM |
| G05.4 | Myelitis in diseases classified elsewhere | Diagnosis | ICD-10-CM |
| G37.4 | Subacute necrotizing myelitis of central nervous system | Diagnosis | ICD-10-CM |
| G92 | Toxic encephalopathy | Diagnosis | ICD-10-CM |
| M60 | Myositis | Diagnosis | ICD-10-CM |
| M60.0 | Infective myositis | Diagnosis | ICD-10-CM |
| M60.00 | Infective myositis, unspecified site | Diagnosis | ICD-10-CM |
| M60.000 | Infective myositis, unspecified right arm | Diagnosis | ICD-10-CM |
| M60.001 | Infective myositis, unspecified left arm | Diagnosis | ICD-10-CM |
| M60.002 | Infective myositis, unspecified arm | Diagnosis | ICD-10-CM |
| M60.003 | Infective myositis, unspecified right leg | Diagnosis | ICD-10-CM |
| M60.004 | Infective myositis, unspecified left leg | Diagnosis | ICD-10-CM |
| M60.005 | Infective myositis, unspecified leg | Diagnosis | ICD-10-CM |
| M60.009 | Infective myositis, unspecified site | Diagnosis | ICD-10-CM |
| M60.01 | Infective myositis, shoulder | Diagnosis | ICD-10-CM |
| M60.011 | Infective myositis, right shoulder | Diagnosis | ICD-10-CM |
| M60.012 | Infective myositis, left shoulder | Diagnosis | ICD-10-CM |
| M60.019 | Infective myositis, unspecified shoulder | Diagnosis | ICD-10-CM |
| M60.02 | Infective myositis, upper arm | Diagnosis | ICD-10-CM |
| M60.021 | Infective myositis, right upper arm | Diagnosis | ICD-10-CM |
| M60.022 | Infective myositis, left upper arm | Diagnosis | ICD-10-CM |
| M60.029 | Infective myositis, unspecified upper arm | Diagnosis | ICD-10-CM |
| M60.03 | Infective myositis, forearm | Diagnosis | ICD-10-CM |
| M60.031 | Infective myositis, right forearm | Diagnosis | ICD-10-CM |
| M60.032 | Infective myositis, left forearm | Diagnosis | ICD-10-CM |
| M60.039 | Infective myositis, unspecified forearm | Diagnosis | ICD-10-CM |
| M60.04 | Infective myositis, hand and fingers | Diagnosis | ICD-10-CM |
| M60.041 | Infective myositis, right hand | Diagnosis | ICD-10-CM |
| M60.042 | Infective myositis, left hand | Diagnosis | ICD-10-CM |
| M60.043 | Infective myositis, unspecified hand | Diagnosis | ICD-10-CM |
| M60.044 | Infective myositis, right finger(s) | Diagnosis | ICD-10-CM |
| M60.045 | Infective myositis, left finger(s) | Diagnosis | ICD-10-CM |
| M60.046 | Infective myositis, unspecified finger(s) | Diagnosis | ICD-10-CM |
| M60.05 | Infective myositis, thigh | Diagnosis | ICD-10-CM |
| M60.051 | Infective myositis, right thigh | Diagnosis | ICD-10-CM |
| M60.052 | Infective myositis, left thigh | Diagnosis | ICD-10-CM |
| M60.059 | Infective myositis, unspecified thigh | Diagnosis | ICD-10-CM |
| M60.06 | Infective myositis, lower leg | Diagnosis | ICD-10-CM |
| M60.061 | Infective myositis, right lower leg | Diagnosis | ICD-10-CM |
| M60.062 | Infective myositis, left lower leg | Diagnosis | ICD-10-CM |
| M60.069 | Infective myositis, unspecified lower leg | Diagnosis | ICD-10-CM |
| M60.07 | Infective myositis, ankle, foot and toes | Diagnosis | ICD-10-CM |
| M60.070 | Infective myositis, right ankle | Diagnosis | ICD-10-CM |
| M60.071 | Infective myositis, left ankle | Diagnosis | ICD-10-CM |
| M60.072 | Infective myositis, unspecified ankle | Diagnosis | ICD-10-CM |
| M60.073 | Infective myositis, right foot | Diagnosis | ICD-10-CM |
| M60.074 | Infective myositis, left foot | Diagnosis | ICD-10-CM |
| M60.075 | Infective myositis, unspecified foot | Diagnosis | ICD-10-CM |
| M60.076 | Infective myositis, right toe(s) | Diagnosis | ICD-10-CM |
| M60.077 | Infective myositis, left toe(s) | Diagnosis | ICD-10-CM |
| M60.078 | Infective myositis, unspecified toe(s) | Diagnosis | ICD-10-CM |
| M60.08 | Infective myositis, other site | Diagnosis | ICD-10-CM |
| M60.09 | Infective myositis, multiple sites | Diagnosis | ICD-10-CM |
| M60.1 | Interstitial myositis | Diagnosis | ICD-10-CM |
| M60.10 | Interstitial myositis of unspecified site | Diagnosis | ICD-10-CM |
| M60.11 | Interstitial myositis, shoulder | Diagnosis | ICD-10-CM |
| M60.111 | Interstitial myositis, right shoulder | Diagnosis | ICD-10-CM |
| M60.112 | Interstitial myositis, left shoulder | Diagnosis | ICD-10-CM |
| M60.119 | Interstitial myositis, unspecified shoulder | Diagnosis | ICD-10-CM |
| M60.12 | Interstitial myositis, upper arm | Diagnosis | ICD-10-CM |
| M60.121 | Interstitial myositis, right upper arm | Diagnosis | ICD-10-CM |
| M60.122 | Interstitial myositis, left upper arm | Diagnosis | ICD-10-CM |
| M60.129 | Interstitial myositis, unspecified upper arm | Diagnosis | ICD-10-CM |
| M60.13 | Interstitial myositis, forearm | Diagnosis | ICD-10-CM |
| M60.131 | Interstitial myositis, right forearm | Diagnosis | ICD-10-CM |
| M60.132 | Interstitial myositis, left forearm | Diagnosis | ICD-10-CM |
| M60.139 | Interstitial myositis, unspecified forearm | Diagnosis | ICD-10-CM |
| M60.14 | Interstitial myositis, hand | Diagnosis | ICD-10-CM |
| M60.141 | Interstitial myositis, right hand | Diagnosis | ICD-10-CM |
| M60.142 | Interstitial myositis, left hand | Diagnosis | ICD-10-CM |
| M60.149 | Interstitial myositis, unspecified hand | Diagnosis | ICD-10-CM |
| M60.15 | Interstitial myositis, thigh | Diagnosis | ICD-10-CM |
| M60.151 | Interstitial myositis, right thigh | Diagnosis | ICD-10-CM |
| M60.152 | Interstitial myositis, left thigh | Diagnosis | ICD-10-CM |
| M60.159 | Interstitial myositis, unspecified thigh | Diagnosis | ICD-10-CM |
| M60.16 | Interstitial myositis, lower leg | Diagnosis | ICD-10-CM |
| M60.161 | Interstitial myositis, right lower leg | Diagnosis | ICD-10-CM |
| M60.162 | Interstitial myositis, left lower leg | Diagnosis | ICD-10-CM |
| M60.169 | Interstitial myositis, unspecified lower leg | Diagnosis | ICD-10-CM |
| M60.17 | Interstitial myositis, ankle and foot | Diagnosis | ICD-10-CM |
| M60.171 | Interstitial myositis, right ankle and foot | Diagnosis | ICD-10-CM |
| M60.172 | Interstitial myositis, left ankle and foot | Diagnosis | ICD-10-CM |
| M60.179 | Interstitial myositis, unspecified ankle and foot | Diagnosis | ICD-10-CM |
| M60.18 | Interstitial myositis, other site | Diagnosis | ICD-10-CM |
| M60.19 | Interstitial myositis, multiple sites | Diagnosis | ICD-10-CM |
| M60.2 | Foreign body granuloma of soft tissue, not elsewhere classified | Diagnosis | ICD-10-CM |
| M60.20 | Foreign body granuloma of soft tissue, not elsewhere classified, unspecified site | Diagnosis | ICD-10-CM |
| M60.21 | Foreign body granuloma of soft tissue, not elsewhere classified, shoulder | Diagnosis | ICD-10-CM |
| M60.211 | Foreign body granuloma of soft tissue, not elsewhere classified, right shoulder | Diagnosis | ICD-10-CM |
| M60.212 | Foreign body granuloma of soft tissue, not elsewhere classified, left shoulder | Diagnosis | ICD-10-CM |
| M60.219 | Foreign body granuloma of soft tissue, not elsewhere classified, unspecified shoulder | Diagnosis | ICD-10-CM |
| M60.22 | Foreign body granuloma of soft tissue, not elsewhere classified, upper arm | Diagnosis | ICD-10-CM |
| M60.221 | Foreign body granuloma of soft tissue, not elsewhere classified, right upper arm | Diagnosis | ICD-10-CM |
| M60.222 | Foreign body granuloma of soft tissue, not elsewhere classified, left upper arm | Diagnosis | ICD-10-CM |
| M60.229 | Foreign body granuloma of soft tissue, not elsewhere classified, unspecified upper arm | Diagnosis | ICD-10-CM |
| M60.23 | Foreign body granuloma of soft tissue, not elsewhere classified, forearm | Diagnosis | ICD-10-CM |
| M60.231 | Foreign body granuloma of soft tissue, not elsewhere classified, right forearm | Diagnosis | ICD-10-CM |
| M60.232 | Foreign body granuloma of soft tissue, not elsewhere classified, left forearm | Diagnosis | ICD-10-CM |
| M60.239 | Foreign body granuloma of soft tissue, not elsewhere classified, unspecified forearm | Diagnosis | ICD-10-CM |
| M60.24 | Foreign body granuloma of soft tissue, not elsewhere classified, hand | Diagnosis | ICD-10-CM |
| M60.241 | Foreign body granuloma of soft tissue, not elsewhere classified, right hand | Diagnosis | ICD-10-CM |
| M60.242 | Foreign body granuloma of soft tissue, not elsewhere classified, left hand | Diagnosis | ICD-10-CM |
| M60.249 | Foreign body granuloma of soft tissue, not elsewhere classified, unspecified hand | Diagnosis | ICD-10-CM |
| M60.25 | Foreign body granuloma of soft tissue, not elsewhere classified, thigh | Diagnosis | ICD-10-CM |
| M60.251 | Foreign body granuloma of soft tissue, not elsewhere classified, right thigh | Diagnosis | ICD-10-CM |
| M60.252 | Foreign body granuloma of soft tissue, not elsewhere classified, left thigh | Diagnosis | ICD-10-CM |
| M60.259 | Foreign body granuloma of soft tissue, not elsewhere classified, unspecified thigh | Diagnosis | ICD-10-CM |
| M60.26 | Foreign body granuloma of soft tissue, not elsewhere classified, lower leg | Diagnosis | ICD-10-CM |
| M60.261 | Foreign body granuloma of soft tissue, not elsewhere classified, right lower leg | Diagnosis | ICD-10-CM |
| M60.262 | Foreign body granuloma of soft tissue, not elsewhere classified, left lower leg | Diagnosis | ICD-10-CM |
| M60.269 | Foreign body granuloma of soft tissue, not elsewhere classified, unspecified lower leg | Diagnosis | ICD-10-CM |
| M60.27 | Foreign body granuloma of soft tissue, not elsewhere classified, ankle and foot | Diagnosis | ICD-10-CM |
| M60.271 | Foreign body granuloma of soft tissue, not elsewhere classified, right ankle and foot | Diagnosis | ICD-10-CM |
| M60.272 | Foreign body granuloma of soft tissue, not elsewhere classified, left ankle and foot | Diagnosis | ICD-10-CM |
| M60.279 | Foreign body granuloma of soft tissue, not elsewhere classified, unspecified ankle and foot | Diagnosis | ICD-10-CM |
| M60.28 | Foreign body granuloma of soft tissue, not elsewhere classified, other site | Diagnosis | ICD-10-CM |
| M60.8 | Other myositis | Diagnosis | ICD-10-CM |
| M60.80 | Other myositis, unspecified site | Diagnosis | ICD-10-CM |
| M60.81 | Other myositis shoulder | Diagnosis | ICD-10-CM |
| M60.811 | Other myositis, right shoulder | Diagnosis | ICD-10-CM |
| M60.812 | Other myositis, left shoulder | Diagnosis | ICD-10-CM |
| M60.819 | Other myositis, unspecified shoulder | Diagnosis | ICD-10-CM |
| M60.82 | Other myositis, upper arm | Diagnosis | ICD-10-CM |
| M60.821 | Other myositis, right upper arm | Diagnosis | ICD-10-CM |
| M60.822 | Other myositis, left upper arm | Diagnosis | ICD-10-CM |
| M60.829 | Other myositis, unspecified upper arm | Diagnosis | ICD-10-CM |
| M60.83 | Other myositis, forearm | Diagnosis | ICD-10-CM |
| M60.831 | Other myositis, right forearm | Diagnosis | ICD-10-CM |
| M60.832 | Other myositis, left forearm | Diagnosis | ICD-10-CM |
| M60.839 | Other myositis, unspecified forearm | Diagnosis | ICD-10-CM |
| M60.84 | Other myositis, hand | Diagnosis | ICD-10-CM |
| M60.841 | Other myositis, right hand | Diagnosis | ICD-10-CM |
| M60.842 | Other myositis, left hand | Diagnosis | ICD-10-CM |
| M60.849 | Other myositis, unspecified hand | Diagnosis | ICD-10-CM |
| M60.85 | Other myositis, thigh | Diagnosis | ICD-10-CM |
| M60.851 | Other myositis, right thigh | Diagnosis | ICD-10-CM |
| M60.852 | Other myositis, left thigh | Diagnosis | ICD-10-CM |
| M60.859 | Other myositis, unspecified thigh | Diagnosis | ICD-10-CM |
| M60.86 | Other myositis, lower leg | Diagnosis | ICD-10-CM |
| M60.861 | Other myositis, right lower leg | Diagnosis | ICD-10-CM |
| M60.862 | Other myositis, left lower leg | Diagnosis | ICD-10-CM |
| M60.869 | Other myositis, unspecified lower leg | Diagnosis | ICD-10-CM |
| M60.87 | Other myositis, ankle and foot | Diagnosis | ICD-10-CM |
| M60.871 | Other myositis, right ankle and foot | Diagnosis | ICD-10-CM |
| M60.872 | Other myositis, left ankle and foot | Diagnosis | ICD-10-CM |
| M60.879 | Other myositis, unspecified ankle and foot | Diagnosis | ICD-10-CM |
| M60.88 | Other myositis, other site | Diagnosis | ICD-10-CM |
| M60.89 | Other myositis, multiple sites | Diagnosis | ICD-10-CM |
| M60.9 | Myositis, unspecified | Diagnosis | ICD-10-CM |
| M62.82 | Rhabdomyolysis | Diagnosis | ICD-10-CM |
| R78.81 | Bacteremia | Diagnosis | ICD-10-CM |
| I21.01 | ST elevation (STEMI) myocardial infarction involving left main coronary artery | Diagnosis | ICD-10-CM |
| I21.02 | ST elevation (STEMI) myocardial infarction involving left anterior descending coronary artery | Diagnosis | ICD-10-CM |
| I21.09 | ST elevation (STEMI) myocardial infarction involving other coronary artery of anterior wall | Diagnosis | ICD-10-CM |
| I21.11 | ST elevation (STEMI) myocardial infarction involving right coronary artery | Diagnosis | ICD-10-CM |
| I21.19 | ST elevation (STEMI) myocardial infarction involving other coronary artery of inferior wall | Diagnosis | ICD-10-CM |
| I21.21 | ST elevation (STEMI) myocardial infarction involving left circumflex coronary artery | Diagnosis | ICD-10-CM |
| I21.29 | ST elevation (STEMI) myocardial infarction involving other sites | Diagnosis | ICD-10-CM |
| I21.3 | ST elevation (STEMI) myocardial infarction of unspecified site | Diagnosis | ICD-10-CM |
| I21.4 | Non-ST elevation (NSTEMI) myocardial infarction | Diagnosis | ICD-10-CM |
| I21.9 | Acute myocardial infarction, unspecified | Diagnosis | ICD-10-CM |
| I21.A1 | Myocardial infarction type 2 | Diagnosis | ICD-10-CM |
| I21.A9 | Other myocardial infarction type | Diagnosis | ICD-10-CM |
| I22.0 | Subsequent ST elevation (STEMI) myocardial infarction of anterior wall | Diagnosis | ICD-10-CM |
| I22.1 | Subsequent ST elevation (STEMI) myocardial infarction of inferior wall | Diagnosis | ICD-10-CM |
| I22.2 | Subsequent non-ST elevation (NSTEMI) myocardial infarction | Diagnosis | ICD-10-CM |
| I22.8 | Subsequent ST elevation (STEMI) myocardial infarction of other sites | Diagnosis | ICD-10-CM |
| I22.9 | Subsequent ST elevation (STEMI) myocardial infarction of unspecified site | Diagnosis | ICD-10-CM |
| **Myocardial Infarction** | | | |
| I21.01 | ST elevation (STEMI) myocardial infarction involving left main coronary artery | Diagnosis | ICD-10-CM |
| I21.02 | ST elevation (STEMI) myocardial infarction involving left anterior descending coronary artery | Diagnosis | ICD-10-CM |
| I21.09 | ST elevation (STEMI) myocardial infarction involving other coronary artery of anterior wall | Diagnosis | ICD-10-CM |
| I21.11 | ST elevation (STEMI) myocardial infarction involving right coronary artery | Diagnosis | ICD-10-CM |
| I21.19 | ST elevation (STEMI) myocardial infarction involving other coronary artery of inferior wall | Diagnosis | ICD-10-CM |
| I21.21 | ST elevation (STEMI) myocardial infarction involving left circumflex coronary artery | Diagnosis | ICD-10-CM |
| I21.29 | ST elevation (STEMI) myocardial infarction involving other sites | Diagnosis | ICD-10-CM |
| I21.3 | ST elevation (STEMI) myocardial infarction of unspecified site | Diagnosis | ICD-10-CM |
| I21.4 | Non-ST elevation (NSTEMI) myocardial infarction | Diagnosis | ICD-10-CM |
| I21.9 | Acute myocardial infarction, unspecified | Diagnosis | ICD-10-CM |
| I21.A1 | Myocardial infarction type 2 | Diagnosis | ICD-10-CM |
| I21.A9 | Other myocardial infarction type | Diagnosis | ICD-10-CM |
| I22.0 | Subsequent ST elevation (STEMI) myocardial infarction of anterior wall | Diagnosis | ICD-10-CM |
| I22.1 | Subsequent ST elevation (STEMI) myocardial infarction of inferior wall | Diagnosis | ICD-10-CM |
| I22.2 | Subsequent non-ST elevation (NSTEMI) myocardial infarction | Diagnosis | ICD-10-CM |
| I22.8 | Subsequent ST elevation (STEMI) myocardial infarction of other sites | Diagnosis | ICD-10-CM |
| I22.9 | Subsequent ST elevation (STEMI) myocardial infarction of unspecified site | Diagnosis | ICD-10-CM |
| **Stroke** | | | |
| I60.00 | Nontraumatic subarachnoid hemorrhage from unspecified carotid siphon and bifurcation | Diagnosis | ICD-10-CM |
| I60.01 | Nontraumatic subarachnoid hemorrhage from right carotid siphon and bifurcation | Diagnosis | ICD-10-CM |
| I60.02 | Nontraumatic subarachnoid hemorrhage from left carotid siphon and bifurcation | Diagnosis | ICD-10-CM |
| I60.10 | Nontraumatic subarachnoid hemorrhage from unspecified middle cerebral artery | Diagnosis | ICD-10-CM |
| I60.11 | Nontraumatic subarachnoid hemorrhage from right middle cerebral artery | Diagnosis | ICD-10-CM |
| I60.12 | Nontraumatic subarachnoid hemorrhage from left middle cerebral artery | Diagnosis | ICD-10-CM |
| I60.2 | Nontraumatic subarachnoid hemorrhage from anterior communicating artery | Diagnosis | ICD-10-CM |
| I60.30 | Nontraumatic subarachnoid hemorrhage from unspecified posterior communicating artery | Diagnosis | ICD-10-CM |
| I60.31 | Nontraumatic subarachnoid hemorrhage from right posterior communicating artery | Diagnosis | ICD-10-CM |
| I60.32 | Nontraumatic subarachnoid hemorrhage from left posterior communicating artery | Diagnosis | ICD-10-CM |
| I60.4 | Nontraumatic subarachnoid hemorrhage from basilar artery | Diagnosis | ICD-10-CM |
| I60.50 | Nontraumatic subarachnoid hemorrhage from unspecified vertebral artery | Diagnosis | ICD-10-CM |
| I60.51 | Nontraumatic subarachnoid hemorrhage from right vertebral artery | Diagnosis | ICD-10-CM |
| I60.52 | Nontraumatic subarachnoid hemorrhage from left vertebral artery | Diagnosis | ICD-10-CM |
| I60.6 | Nontraumatic subarachnoid hemorrhage from other intracranial arteries | Diagnosis | ICD-10-CM |
| I60.7 | Nontraumatic subarachnoid hemorrhage from unspecified intracranial artery | Diagnosis | ICD-10-CM |
| I60.8 | Other nontraumatic subarachnoid hemorrhage | Diagnosis | ICD-10-CM |
| I60.9 | Nontraumatic subarachnoid hemorrhage, unspecified | Diagnosis | ICD-10-CM |
| I61.0 | Nontraumatic intracerebral hemorrhage in hemisphere, subcortical | Diagnosis | ICD-10-CM |
| I61.1 | Nontraumatic intracerebral hemorrhage in hemisphere, cortical | Diagnosis | ICD-10-CM |
| I61.2 | Nontraumatic intracerebral hemorrhage in hemisphere, unspecified | Diagnosis | ICD-10-CM |
| I61.3 | Nontraumatic intracerebral hemorrhage in brain stem | Diagnosis | ICD-10-CM |
| I61.4 | Nontraumatic intracerebral hemorrhage in cerebellum | Diagnosis | ICD-10-CM |
| I61.5 | Nontraumatic intracerebral hemorrhage, intraventricular | Diagnosis | ICD-10-CM |
| I61.6 | Nontraumatic intracerebral hemorrhage, multiple localized | Diagnosis | ICD-10-CM |
| I61.8 | Other nontraumatic intracerebral hemorrhage | Diagnosis | ICD-10-CM |
| I61.9 | Nontraumatic intracerebral hemorrhage, unspecified | Diagnosis | ICD-10-CM |
| I62.9 | Nontraumatic intracranial hemorrhage, unspecified | Diagnosis | ICD-10-CM |
| I63.00 | Cerebral infarction due to thrombosis of unspecified precerebral artery | Diagnosis | ICD-10-CM |
| I63.011 | Cerebral infarction due to thrombosis of right vertebral artery | Diagnosis | ICD-10-CM |
| I63.012 | Cerebral infarction due to thrombosis of left vertebral artery | Diagnosis | ICD-10-CM |
| I63.013 | Cerebral infarction due to thrombosis of bilateral vertebral arteries | Diagnosis | ICD-10-CM |
| I63.019 | Cerebral infarction due to thrombosis of unspecified vertebral artery | Diagnosis | ICD-10-CM |
| I63.02 | Cerebral infarction due to thrombosis of basilar artery | Diagnosis | ICD-10-CM |
| I63.031 | Cerebral infarction due to thrombosis of right carotid artery | Diagnosis | ICD-10-CM |
| I63.032 | Cerebral infarction due to thrombosis of left carotid artery | Diagnosis | ICD-10-CM |
| I63.033 | Cerebral infarction due to thrombosis of bilateral carotid arteries | Diagnosis | ICD-10-CM |
| I63.039 | Cerebral infarction due to thrombosis of unspecified carotid artery | Diagnosis | ICD-10-CM |
| I63.09 | Cerebral infarction due to thrombosis of other precerebral artery | Diagnosis | ICD-10-CM |
| I63.10 | Cerebral infarction due to embolism of unspecified precerebral artery | Diagnosis | ICD-10-CM |
| I63.111 | Cerebral infarction due to embolism of right vertebral artery | Diagnosis | ICD-10-CM |
| I63.112 | Cerebral infarction due to embolism of left vertebral artery | Diagnosis | ICD-10-CM |
| I63.113 | Cerebral infarction due to embolism of bilateral vertebral arteries | Diagnosis | ICD-10-CM |
| I63.119 | Cerebral infarction due to embolism of unspecified vertebral artery | Diagnosis | ICD-10-CM |
| I63.12 | Cerebral infarction due to embolism of basilar artery | Diagnosis | ICD-10-CM |
| I63.131 | Cerebral infarction due to embolism of right carotid artery | Diagnosis | ICD-10-CM |
| I63.132 | Cerebral infarction due to embolism of left carotid artery | Diagnosis | ICD-10-CM |
| I63.133 | Cerebral infarction due to embolism of bilateral carotid arteries | Diagnosis | ICD-10-CM |
| I63.139 | Cerebral infarction due to embolism of unspecified carotid artery | Diagnosis | ICD-10-CM |
| I63.19 | Cerebral infarction due to embolism of other precerebral artery | Diagnosis | ICD-10-CM |
| I63.20 | Cerebral infarction due to unspecified occlusion or stenosis of unspecified precerebral arteries | Diagnosis | ICD-10-CM |
| I63.211 | Cerebral infarction due to unspecified occlusion or stenosis of right vertebral artery | Diagnosis | ICD-10-CM |
| I63.212 | Cerebral infarction due to unspecified occlusion or stenosis of left vertebral artery | Diagnosis | ICD-10-CM |
| I63.213 | Cerebral infarction due to unspecified occlusion or stenosis of bilateral vertebral arteries | Diagnosis | ICD-10-CM |
| I63.219 | Cerebral infarction due to unspecified occlusion or stenosis of unspecified vertebral artery | Diagnosis | ICD-10-CM |
| I63.22 | Cerebral infarction due to unspecified occlusion or stenosis of basilar artery | Diagnosis | ICD-10-CM |
| I63.231 | Cerebral infarction due to unspecified occlusion or stenosis of right carotid arteries | Diagnosis | ICD-10-CM |
| I63.232 | Cerebral infarction due to unspecified occlusion or stenosis of left carotid arteries | Diagnosis | ICD-10-CM |
| I63.233 | Cerebral infarction due to unspecified occlusion or stenosis of bilateral carotid arteries | Diagnosis | ICD-10-CM |
| I63.239 | Cerebral infarction due to unspecified occlusion or stenosis of unspecified carotid artery | Diagnosis | ICD-10-CM |
| I63.29 | Cerebral infarction due to unspecified occlusion or stenosis of other precerebral arteries | Diagnosis | ICD-10-CM |
| I63.30 | Cerebral infarction due to thrombosis of unspecified cerebral artery | Diagnosis | ICD-10-CM |
| I63.311 | Cerebral infarction due to thrombosis of right middle cerebral artery | Diagnosis | ICD-10-CM |
| I63.312 | Cerebral infarction due to thrombosis of left middle cerebral artery | Diagnosis | ICD-10-CM |
| I63.313 | Cerebral infarction due to thrombosis of bilateral middle cerebral arteries | Diagnosis | ICD-10-CM |
| I63.319 | Cerebral infarction due to thrombosis of unspecified middle cerebral artery | Diagnosis | ICD-10-CM |
| I63.321 | Cerebral infarction due to thrombosis of right anterior cerebral artery | Diagnosis | ICD-10-CM |
| I63.322 | Cerebral infarction due to thrombosis of left anterior cerebral artery | Diagnosis | ICD-10-CM |
| I63.323 | Cerebral infarction due to thrombosis of bilateral anterior cerebral arteries | Diagnosis | ICD-10-CM |
| I63.329 | Cerebral infarction due to thrombosis of unspecified anterior cerebral artery | Diagnosis | ICD-10-CM |
| I63.331 | Cerebral infarction due to thrombosis of right posterior cerebral artery | Diagnosis | ICD-10-CM |
| I63.332 | Cerebral infarction due to thrombosis of left posterior cerebral artery | Diagnosis | ICD-10-CM |
| I63.333 | Cerebral infarction due to thrombosis of bilateral posterior cerebral arteries | Diagnosis | ICD-10-CM |
| I63.339 | Cerebral infarction due to thrombosis of unspecified posterior cerebral artery | Diagnosis | ICD-10-CM |
| I63.341 | Cerebral infarction due to thrombosis of right cerebellar artery | Diagnosis | ICD-10-CM |
| I63.342 | Cerebral infarction due to thrombosis of left cerebellar artery | Diagnosis | ICD-10-CM |
| I63.343 | Cerebral infarction due to thrombosis of bilateral cerebellar arteries | Diagnosis | ICD-10-CM |
| I63.349 | Cerebral infarction due to thrombosis of unspecified cerebellar artery | Diagnosis | ICD-10-CM |
| I63.39 | Cerebral infarction due to thrombosis of other cerebral artery | Diagnosis | ICD-10-CM |
| I63.40 | Cerebral infarction due to embolism of unspecified cerebral artery | Diagnosis | ICD-10-CM |
| I63.411 | Cerebral infarction due to embolism of right middle cerebral artery | Diagnosis | ICD-10-CM |
| I63.412 | Cerebral infarction due to embolism of left middle cerebral artery | Diagnosis | ICD-10-CM |
| I63.413 | Cerebral infarction due to embolism of bilateral middle cerebral arteries | Diagnosis | ICD-10-CM |
| I63.419 | Cerebral infarction due to embolism of unspecified middle cerebral artery | Diagnosis | ICD-10-CM |
| I63.421 | Cerebral infarction due to embolism of right anterior cerebral artery | Diagnosis | ICD-10-CM |
| I63.422 | Cerebral infarction due to embolism of left anterior cerebral artery | Diagnosis | ICD-10-CM |
| I63.423 | Cerebral infarction due to embolism of bilateral anterior cerebral arteries | Diagnosis | ICD-10-CM |
| I63.429 | Cerebral infarction due to embolism of unspecified anterior cerebral artery | Diagnosis | ICD-10-CM |
| I63.431 | Cerebral infarction due to embolism of right posterior cerebral artery | Diagnosis | ICD-10-CM |
| I63.432 | Cerebral infarction due to embolism of left posterior cerebral artery | Diagnosis | ICD-10-CM |
| I63.433 | Cerebral infarction due to embolism of bilateral posterior cerebral arteries | Diagnosis | ICD-10-CM |
| I63.439 | Cerebral infarction due to embolism of unspecified posterior cerebral artery | Diagnosis | ICD-10-CM |
| I63.441 | Cerebral infarction due to embolism of right cerebellar artery | Diagnosis | ICD-10-CM |
| I63.442 | Cerebral infarction due to embolism of left cerebellar artery | Diagnosis | ICD-10-CM |
| I63.443 | Cerebral infarction due to embolism of bilateral cerebellar arteries | Diagnosis | ICD-10-CM |
| I63.449 | Cerebral infarction due to embolism of unspecified cerebellar artery | Diagnosis | ICD-10-CM |
| I63.49 | Cerebral infarction due to embolism of other cerebral artery | Diagnosis | ICD-10-CM |
| I63.50 | Cerebral infarction due to unspecified occlusion or stenosis of unspecified cerebral artery | Diagnosis | ICD-10-CM |
| I63.511 | Cerebral infarction due to unspecified occlusion or stenosis of right middle cerebral artery | Diagnosis | ICD-10-CM |
| I63.512 | Cerebral infarction due to unspecified occlusion or stenosis of left middle cerebral artery | Diagnosis | ICD-10-CM |
| I63.513 | Cerebral infarction due to unspecified occlusion or stenosis of bilateral middle cerebral arteries | Diagnosis | ICD-10-CM |
| I63.519 | Cerebral infarction due to unspecified occlusion or stenosis of unspecified middle cerebral artery | Diagnosis | ICD-10-CM |
| I63.521 | Cerebral infarction due to unspecified occlusion or stenosis of right anterior cerebral artery | Diagnosis | ICD-10-CM |
| I63.522 | Cerebral infarction due to unspecified occlusion or stenosis of left anterior cerebral artery | Diagnosis | ICD-10-CM |
| I63.523 | Cerebral infarction due to unspecified occlusion or stenosis of bilateral anterior cerebral arteries | Diagnosis | ICD-10-CM |
| I63.529 | Cerebral infarction due to unspecified occlusion or stenosis of unspecified anterior cerebral artery | Diagnosis | ICD-10-CM |
| I63.531 | Cerebral infarction due to unspecified occlusion or stenosis of right posterior cerebral artery | Diagnosis | ICD-10-CM |
| I63.532 | Cerebral infarction due to unspecified occlusion or stenosis of left posterior cerebral artery | Diagnosis | ICD-10-CM |
| I63.533 | Cerebral infarction due to unspecified occlusion or stenosis of bilateral posterior cerebral arteries | Diagnosis | ICD-10-CM |
| I63.539 | Cerebral infarction due to unspecified occlusion or stenosis of unspecified posterior cerebral artery | Diagnosis | ICD-10-CM |
| I63.541 | Cerebral infarction due to unspecified occlusion or stenosis of right cerebellar artery | Diagnosis | ICD-10-CM |
| I63.542 | Cerebral infarction due to unspecified occlusion or stenosis of left cerebellar artery | Diagnosis | ICD-10-CM |
| I63.543 | Cerebral infarction due to unspecified occlusion or stenosis of bilateral cerebellar arteries | Diagnosis | ICD-10-CM |
| I63.549 | Cerebral infarction due to unspecified occlusion or stenosis of unspecified cerebellar artery | Diagnosis | ICD-10-CM |
| I63.59 | Cerebral infarction due to unspecified occlusion or stenosis of other cerebral artery | Diagnosis | ICD-10-CM |
| I63.6 | Cerebral infarction due to cerebral venous thrombosis, nonpyogenic | Diagnosis | ICD-10-CM |
| I63.8 | Other cerebral infarction | Diagnosis | ICD-10-CM |
| I63.9 | Cerebral infarction, unspecified | Diagnosis | ICD-10-CM |
| I65.01 | Occlusion and stenosis of right vertebral artery | Diagnosis | ICD-10-CM |
| I65.02 | Occlusion and stenosis of left vertebral artery | Diagnosis | ICD-10-CM |
| I65.03 | Occlusion and stenosis of bilateral vertebral arteries | Diagnosis | ICD-10-CM |
| I65.09 | Occlusion and stenosis of unspecified vertebral artery | Diagnosis | ICD-10-CM |
| I65.1 | Occlusion and stenosis of basilar artery | Diagnosis | ICD-10-CM |
| I65.21 | Occlusion and stenosis of right carotid artery | Diagnosis | ICD-10-CM |
| I65.22 | Occlusion and stenosis of left carotid artery | Diagnosis | ICD-10-CM |
| I65.23 | Occlusion and stenosis of bilateral carotid arteries | Diagnosis | ICD-10-CM |
| I65.29 | Occlusion and stenosis of unspecified carotid artery | Diagnosis | ICD-10-CM |
| I65.8 | Occlusion and stenosis of other precerebral arteries | Diagnosis | ICD-10-CM |
| I65.9 | Occlusion and stenosis of unspecified precerebral artery | Diagnosis | ICD-10-CM |
| I66.01 | Occlusion and stenosis of right middle cerebral artery | Diagnosis | ICD-10-CM |
| I66.02 | Occlusion and stenosis of left middle cerebral artery | Diagnosis | ICD-10-CM |
| I66.03 | Occlusion and stenosis of bilateral middle cerebral arteries | Diagnosis | ICD-10-CM |
| I66.09 | Occlusion and stenosis of unspecified middle cerebral artery | Diagnosis | ICD-10-CM |
| I66.11 | Occlusion and stenosis of right anterior cerebral artery | Diagnosis | ICD-10-CM |
| I66.12 | Occlusion and stenosis of left anterior cerebral artery | Diagnosis | ICD-10-CM |
| I66.13 | Occlusion and stenosis of bilateral anterior cerebral arteries | Diagnosis | ICD-10-CM |
| I66.19 | Occlusion and stenosis of unspecified anterior cerebral artery | Diagnosis | ICD-10-CM |
| I66.21 | Occlusion and stenosis of right posterior cerebral artery | Diagnosis | ICD-10-CM |
| I66.22 | Occlusion and stenosis of left posterior cerebral artery | Diagnosis | ICD-10-CM |
| I66.23 | Occlusion and stenosis of bilateral posterior cerebral arteries | Diagnosis | ICD-10-CM |
| I66.29 | Occlusion and stenosis of unspecified posterior cerebral artery | Diagnosis | ICD-10-CM |
| I66.3 | Occlusion and stenosis of cerebellar arteries | Diagnosis | ICD-10-CM |
| I66.8 | Occlusion and stenosis of other cerebral arteries | Diagnosis | ICD-10-CM |
| I66.9 | Occlusion and stenosis of unspecified cerebral artery | Diagnosis | ICD-10-CM |
| I67.89 | Other cerebrovascular disease | Diagnosis | ICD-10-CM |
| I69.00 | Unspecified sequelae of nontraumatic subarachnoid hemorrhage | Diagnosis | ICD-10-CM |
| I69.010 | Attention and concentration deficit following nontraumatic subarachnoid hemorrhage | Diagnosis | ICD-10-CM |
| I69.011 | Memory deficit following nontraumatic subarachnoid hemorrhage | Diagnosis | ICD-10-CM |
| I69.012 | Visuospatial deficit and spatial neglect following nontraumatic subarachnoid hemorrhage | Diagnosis | ICD-10-CM |
| I69.013 | Psychomotor deficit following nontraumatic subarachnoid hemorrhage | Diagnosis | ICD-10-CM |
| I69.014 | Frontal lobe and executive function deficit following nontraumatic subarachnoid hemorrhage | Diagnosis | ICD-10-CM |
| I69.015 | Cognitive social or emotional deficit following nontraumatic subarachnoid hemorrhage | Diagnosis | ICD-10-CM |
| I69.018 | Other symptoms and signs involving cognitive functions following nontraumatic subarachnoid hemorrhage | Diagnosis | ICD-10-CM |
| I69.019 | Unspecified symptoms and signs involving cognitive functions following nontraumatic subarachnoid hemorrhage | Diagnosis | ICD-10-CM |
| I69.020 | Aphasia following nontraumatic subarachnoid hemorrhage | Diagnosis | ICD-10-CM |
| I69.021 | Dysphasia following nontraumatic subarachnoid hemorrhage | Diagnosis | ICD-10-CM |
| I69.022 | Dysarthria following nontraumatic subarachnoid hemorrhage | Diagnosis | ICD-10-CM |
| I69.023 | Fluency disorder following nontraumatic subarachnoid hemorrhage | Diagnosis | ICD-10-CM |
| I69.028 | Other speech and language deficits following nontraumatic subarachnoid hemorrhage | Diagnosis | ICD-10-CM |
| I69.031 | Monoplegia of upper limb following nontraumatic subarachnoid hemorrhage affecting right dominant side | Diagnosis | ICD-10-CM |
| I69.032 | Monoplegia of upper limb following nontraumatic subarachnoid hemorrhage affecting left dominant side | Diagnosis | ICD-10-CM |
| I69.033 | Monoplegia of upper limb following nontraumatic subarachnoid hemorrhage affecting right non-dominant side | Diagnosis | ICD-10-CM |
| I69.034 | Monoplegia of upper limb following nontraumatic subarachnoid hemorrhage affecting left non-dominant side | Diagnosis | ICD-10-CM |
| I69.039 | Monoplegia of upper limb following nontraumatic subarachnoid hemorrhage affecting unspecified side | Diagnosis | ICD-10-CM |
| I69.041 | Monoplegia of lower limb following nontraumatic subarachnoid hemorrhage affecting right dominant side | Diagnosis | ICD-10-CM |
| I69.042 | Monoplegia of lower limb following nontraumatic subarachnoid hemorrhage affecting left dominant side | Diagnosis | ICD-10-CM |
| I69.043 | Monoplegia of lower limb following nontraumatic subarachnoid hemorrhage affecting right non-dominant side | Diagnosis | ICD-10-CM |
| I69.044 | Monoplegia of lower limb following nontraumatic subarachnoid hemorrhage affecting left non-dominant side | Diagnosis | ICD-10-CM |
| I69.049 | Monoplegia of lower limb following nontraumatic subarachnoid hemorrhage affecting unspecified side | Diagnosis | ICD-10-CM |
| I69.051 | Hemiplegia and hemiparesis following nontraumatic subarachnoid hemorrhage affecting right dominant side | Diagnosis | ICD-10-CM |
| I69.052 | Hemiplegia and hemiparesis following nontraumatic subarachnoid hemorrhage affecting left dominant side | Diagnosis | ICD-10-CM |
| I69.053 | Hemiplegia and hemiparesis following nontraumatic subarachnoid hemorrhage affecting right non-dominant side | Diagnosis | ICD-10-CM |
| I69.054 | Hemiplegia and hemiparesis following nontraumatic subarachnoid hemorrhage affecting left non-dominant side | Diagnosis | ICD-10-CM |
| I69.059 | Hemiplegia and hemiparesis following nontraumatic subarachnoid hemorrhage affecting unspecified side | Diagnosis | ICD-10-CM |
| I69.061 | Other paralytic syndrome following nontraumatic subarachnoid hemorrhage affecting right dominant side | Diagnosis | ICD-10-CM |
| I69.062 | Other paralytic syndrome following nontraumatic subarachnoid hemorrhage affecting left dominant side | Diagnosis | ICD-10-CM |
| I69.063 | Other paralytic syndrome following nontraumatic subarachnoid hemorrhage affecting right non-dominant side | Diagnosis | ICD-10-CM |
| I69.064 | Other paralytic syndrome following nontraumatic subarachnoid hemorrhage affecting left non-dominant side | Diagnosis | ICD-10-CM |
| I69.069 | Other paralytic syndrome following nontraumatic subarachnoid hemorrhage affecting unspecified side | Diagnosis | ICD-10-CM |
| I69.090 | Apraxia following nontraumatic subarachnoid hemorrhage | Diagnosis | ICD-10-CM |
| I69.091 | Dysphagia following nontraumatic subarachnoid hemorrhage | Diagnosis | ICD-10-CM |
| I69.092 | Facial weakness following nontraumatic subarachnoid hemorrhage | Diagnosis | ICD-10-CM |
| I69.093 | Ataxia following nontraumatic subarachnoid hemorrhage | Diagnosis | ICD-10-CM |
| I69.098 | Other sequelae following nontraumatic subarachnoid hemorrhage | Diagnosis | ICD-10-CM |
| I69.10 | Unspecified sequelae of nontraumatic intracerebral hemorrhage | Diagnosis | ICD-10-CM |
| I69.110 | Attention and concentration deficit following nontraumatic intracerebral hemorrhage | Diagnosis | ICD-10-CM |
| I69.111 | Memory deficit following nontraumatic intracerebral hemorrhage | Diagnosis | ICD-10-CM |
| I69.112 | Visuospatial deficit and spatial neglect following nontraumatic intracerebral hemorrhage | Diagnosis | ICD-10-CM |
| I69.113 | Psychomotor deficit following nontraumatic intracerebral hemorrhage | Diagnosis | ICD-10-CM |
| I69.114 | Frontal lobe and executive function deficit following nontraumatic intracerebral hemorrhage | Diagnosis | ICD-10-CM |
| I69.115 | Cognitive social or emotional deficit following nontraumatic intracerebral hemorrhage | Diagnosis | ICD-10-CM |
| I69.118 | Other symptoms and signs involving cognitive functions following nontraumatic intracerebral hemorrhage | Diagnosis | ICD-10-CM |
| I69.119 | Unspecified symptoms and signs involving cognitive functions following nontraumatic intracerebral hemorrhage | Diagnosis | ICD-10-CM |
| I69.120 | Aphasia following nontraumatic intracerebral hemorrhage | Diagnosis | ICD-10-CM |
| I69.121 | Dysphasia following nontraumatic intracerebral hemorrhage | Diagnosis | ICD-10-CM |
| I69.122 | Dysarthria following nontraumatic intracerebral hemorrhage | Diagnosis | ICD-10-CM |
| I69.123 | Fluency disorder following nontraumatic intracerebral hemorrhage | Diagnosis | ICD-10-CM |
| I69.128 | Other speech and language deficits following nontraumatic intracerebral hemorrhage | Diagnosis | ICD-10-CM |
| I69.131 | Monoplegia of upper limb following nontraumatic intracerebral hemorrhage affecting right dominant side | Diagnosis | ICD-10-CM |
| I69.132 | Monoplegia of upper limb following nontraumatic intracerebral hemorrhage affecting left dominant side | Diagnosis | ICD-10-CM |
| I69.133 | Monoplegia of upper limb following nontraumatic intracerebral hemorrhage affecting right non-dominant side | Diagnosis | ICD-10-CM |
| I69.134 | Monoplegia of upper limb following nontraumatic intracerebral hemorrhage affecting left non-dominant side | Diagnosis | ICD-10-CM |
| I69.139 | Monoplegia of upper limb following nontraumatic intracerebral hemorrhage affecting unspecified side | Diagnosis | ICD-10-CM |
| I69.141 | Monoplegia of lower limb following nontraumatic intracerebral hemorrhage affecting right dominant side | Diagnosis | ICD-10-CM |
| I69.142 | Monoplegia of lower limb following nontraumatic intracerebral hemorrhage affecting left dominant side | Diagnosis | ICD-10-CM |
| I69.143 | Monoplegia of lower limb following nontraumatic intracerebral hemorrhage affecting right non-dominant side | Diagnosis | ICD-10-CM |
| I69.144 | Monoplegia of lower limb following nontraumatic intracerebral hemorrhage affecting left non-dominant side | Diagnosis | ICD-10-CM |
| I69.149 | Monoplegia of lower limb following nontraumatic intracerebral hemorrhage affecting unspecified side | Diagnosis | ICD-10-CM |
| I69.151 | Hemiplegia and hemiparesis following nontraumatic intracerebral hemorrhage affecting right dominant side | Diagnosis | ICD-10-CM |
| I69.152 | Hemiplegia and hemiparesis following nontraumatic intracerebral hemorrhage affecting left dominant side | Diagnosis | ICD-10-CM |
| I69.153 | Hemiplegia and hemiparesis following nontraumatic intracerebral hemorrhage affecting right non-dominant side | Diagnosis | ICD-10-CM |
| I69.154 | Hemiplegia and hemiparesis following nontraumatic intracerebral hemorrhage affecting left non-dominant side | Diagnosis | ICD-10-CM |
| I69.159 | Hemiplegia and hemiparesis following nontraumatic intracerebral hemorrhage affecting unspecified side | Diagnosis | ICD-10-CM |
| I69.161 | Other paralytic syndrome following nontraumatic intracerebral hemorrhage affecting right dominant side | Diagnosis | ICD-10-CM |
| I69.162 | Other paralytic syndrome following nontraumatic intracerebral hemorrhage affecting left dominant side | Diagnosis | ICD-10-CM |
| I69.163 | Other paralytic syndrome following nontraumatic intracerebral hemorrhage affecting right non-dominant side | Diagnosis | ICD-10-CM |
| I69.164 | Other paralytic syndrome following nontraumatic intracerebral hemorrhage affecting left non-dominant side | Diagnosis | ICD-10-CM |
| I69.169 | Other paralytic syndrome following nontraumatic intracerebral hemorrhage affecting unspecified side | Diagnosis | ICD-10-CM |
| I69.190 | Apraxia following nontraumatic intracerebral hemorrhage | Diagnosis | ICD-10-CM |
| I69.191 | Dysphagia following nontraumatic intracerebral hemorrhage | Diagnosis | ICD-10-CM |
| I69.192 | Facial weakness following nontraumatic intracerebral hemorrhage | Diagnosis | ICD-10-CM |
| I69.193 | Ataxia following nontraumatic intracerebral hemorrhage | Diagnosis | ICD-10-CM |
| I69.198 | Other sequelae of nontraumatic intracerebral hemorrhage | Diagnosis | ICD-10-CM |
| I69.20 | Unspecified sequelae of other nontraumatic intracranial hemorrhage | Diagnosis | ICD-10-CM |
| I69.210 | Attention and concentration deficit following other nontraumatic intracranial hemorrhage | Diagnosis | ICD-10-CM |
| I69.211 | Memory deficit following other nontraumatic intracranial hemorrhage | Diagnosis | ICD-10-CM |
| I69.212 | Visuospatial deficit and spatial neglect following other nontraumatic intracranial hemorrhage | Diagnosis | ICD-10-CM |
| I69.213 | Psychomotor deficit following other nontraumatic intracranial hemorrhage | Diagnosis | ICD-10-CM |
| I69.214 | Frontal lobe and executive function deficit following other nontraumatic intracranial hemorrhage | Diagnosis | ICD-10-CM |
| I69.215 | Cognitive social or emotional deficit following other nontraumatic intracranial hemorrhage | Diagnosis | ICD-10-CM |
| I69.218 | Other symptoms and signs involving cognitive functions following other nontraumatic intracranial hemorrhage | Diagnosis | ICD-10-CM |
| I69.219 | Unspecified symptoms and signs involving cognitive functions following other nontraumatic intracranial hemorrhage | Diagnosis | ICD-10-CM |
| I69.220 | Aphasia following other nontraumatic intracranial hemorrhage | Diagnosis | ICD-10-CM |
| I69.221 | Dysphasia following other nontraumatic intracranial hemorrhage | Diagnosis | ICD-10-CM |
| I69.222 | Dysarthria following other nontraumatic intracranial hemorrhage | Diagnosis | ICD-10-CM |
| I69.223 | Fluency disorder following other nontraumatic intracranial hemorrhage | Diagnosis | ICD-10-CM |
| I69.228 | Other speech and language deficits following other nontraumatic intracranial hemorrhage | Diagnosis | ICD-10-CM |
| I69.231 | Monoplegia of upper limb following other nontraumatic intracranial hemorrhage affecting right dominant side | Diagnosis | ICD-10-CM |
| I69.232 | Monoplegia of upper limb following other nontraumatic intracranial hemorrhage affecting left dominant side | Diagnosis | ICD-10-CM |
| I69.233 | Monoplegia of upper limb following other nontraumatic intracranial hemorrhage affecting right non-dominant side | Diagnosis | ICD-10-CM |
| I69.234 | Monoplegia of upper limb following other nontraumatic intracranial hemorrhage affecting left non-dominant side | Diagnosis | ICD-10-CM |
| I69.239 | Monoplegia of upper limb following other nontraumatic intracranial hemorrhage affecting unspecified side | Diagnosis | ICD-10-CM |
| I69.241 | Monoplegia of lower limb following other nontraumatic intracranial hemorrhage affecting right dominant side | Diagnosis | ICD-10-CM |
| I69.242 | Monoplegia of lower limb following other nontraumatic intracranial hemorrhage affecting left dominant side | Diagnosis | ICD-10-CM |
| I69.243 | Monoplegia of lower limb following other nontraumatic intracranial hemorrhage affecting right non-dominant side | Diagnosis | ICD-10-CM |
| I69.244 | Monoplegia of lower limb following other nontraumatic intracranial hemorrhage affecting left non-dominant side | Diagnosis | ICD-10-CM |
| I69.249 | Monoplegia of lower limb following other nontraumatic intracranial hemorrhage affecting unspecified side | Diagnosis | ICD-10-CM |
| I69.251 | Hemiplegia and hemiparesis following other nontraumatic intracranial hemorrhage affecting right dominant side | Diagnosis | ICD-10-CM |
| I69.252 | Hemiplegia and hemiparesis following other nontraumatic intracranial hemorrhage affecting left dominant side | Diagnosis | ICD-10-CM |
| I69.253 | Hemiplegia and hemiparesis following other nontraumatic intracranial hemorrhage affecting right non-dominant side | Diagnosis | ICD-10-CM |
| I69.254 | Hemiplegia and hemiparesis following other nontraumatic intracranial hemorrhage affecting left non-dominant side | Diagnosis | ICD-10-CM |
| I69.259 | Hemiplegia and hemiparesis following other nontraumatic intracranial hemorrhage affecting unspecified side | Diagnosis | ICD-10-CM |
| I69.261 | Other paralytic syndrome following other nontraumatic intracranial hemorrhage affecting right dominant side | Diagnosis | ICD-10-CM |
| I69.262 | Other paralytic syndrome following other nontraumatic intracranial hemorrhage affecting left dominant side | Diagnosis | ICD-10-CM |
| I69.263 | Other paralytic syndrome following other nontraumatic intracranial hemorrhage affecting right non-dominant side | Diagnosis | ICD-10-CM |
| I69.264 | Other paralytic syndrome following other nontraumatic intracranial hemorrhage affecting left non-dominant side | Diagnosis | ICD-10-CM |
| I69.269 | Other paralytic syndrome following other nontraumatic intracranial hemorrhage affecting unspecified side | Diagnosis | ICD-10-CM |
| I69.290 | Apraxia following other nontraumatic intracranial hemorrhage | Diagnosis | ICD-10-CM |
| I69.291 | Dysphagia following other nontraumatic intracranial hemorrhage | Diagnosis | ICD-10-CM |
| I69.292 | Facial weakness following other nontraumatic intracranial hemorrhage | Diagnosis | ICD-10-CM |
| I69.293 | Ataxia following other nontraumatic intracranial hemorrhage | Diagnosis | ICD-10-CM |
| I69.298 | Other sequelae of other nontraumatic intracranial hemorrhage | Diagnosis | ICD-10-CM |
| I69.30 | Unspecified sequelae of cerebral infarction | Diagnosis | ICD-10-CM |
| I69.310 | Attention and concentration deficit following cerebral infarction | Diagnosis | ICD-10-CM |
| I69.311 | Memory deficit following cerebral infarction | Diagnosis | ICD-10-CM |
| I69.312 | Visuospatial deficit and spatial neglect following cerebral infarction | Diagnosis | ICD-10-CM |
| I69.313 | Psychomotor deficit following cerebral infarction | Diagnosis | ICD-10-CM |
| I69.314 | Frontal lobe and executive function deficit following cerebral infarction | Diagnosis | ICD-10-CM |
| I69.315 | Cognitive social or emotional deficit following cerebral infarction | Diagnosis | ICD-10-CM |
| I69.318 | Other symptoms and signs involving cognitive functions following cerebral infarction | Diagnosis | ICD-10-CM |
| I69.319 | Unspecified symptoms and signs involving cognitive functions following cerebral infarction | Diagnosis | ICD-10-CM |
| I69.320 | Aphasia following cerebral infarction | Diagnosis | ICD-10-CM |
| I69.321 | Dysphasia following cerebral infarction | Diagnosis | ICD-10-CM |
| I69.322 | Dysarthria following cerebral infarction | Diagnosis | ICD-10-CM |
| I69.323 | Fluency disorder following cerebral infarction | Diagnosis | ICD-10-CM |
| I69.328 | Other speech and language deficits following cerebral infarction | Diagnosis | ICD-10-CM |
| I69.331 | Monoplegia of upper limb following cerebral infarction affecting right dominant side | Diagnosis | ICD-10-CM |
| I69.332 | Monoplegia of upper limb following cerebral infarction affecting left dominant side | Diagnosis | ICD-10-CM |
| I69.333 | Monoplegia of upper limb following cerebral infarction affecting right non-dominant side | Diagnosis | ICD-10-CM |
| I69.334 | Monoplegia of upper limb following cerebral infarction affecting left non-dominant side | Diagnosis | ICD-10-CM |
| I69.339 | Monoplegia of upper limb following cerebral infarction affecting unspecified side | Diagnosis | ICD-10-CM |
| I69.341 | Monoplegia of lower limb following cerebral infarction affecting right dominant side | Diagnosis | ICD-10-CM |
| I69.342 | Monoplegia of lower limb following cerebral infarction affecting left dominant side | Diagnosis | ICD-10-CM |
| I69.343 | Monoplegia of lower limb following cerebral infarction affecting right non-dominant side | Diagnosis | ICD-10-CM |
| I69.344 | Monoplegia of lower limb following cerebral infarction affecting left non-dominant side | Diagnosis | ICD-10-CM |
| I69.349 | Monoplegia of lower limb following cerebral infarction affecting unspecified side | Diagnosis | ICD-10-CM |
| I69.351 | Hemiplegia and hemiparesis following cerebral infarction affecting right dominant side | Diagnosis | ICD-10-CM |
| I69.352 | Hemiplegia and hemiparesis following cerebral infarction affecting left dominant side | Diagnosis | ICD-10-CM |
| I69.353 | Hemiplegia and hemiparesis following cerebral infarction affecting right non-dominant side | Diagnosis | ICD-10-CM |
| I69.354 | Hemiplegia and hemiparesis following cerebral infarction affecting left non-dominant side | Diagnosis | ICD-10-CM |
| I69.359 | Hemiplegia and hemiparesis following cerebral infarction affecting unspecified side | Diagnosis | ICD-10-CM |
| I69.361 | Other paralytic syndrome following cerebral infarction affecting right dominant side | Diagnosis | ICD-10-CM |
| I69.362 | Other paralytic syndrome following cerebral infarction affecting left dominant side | Diagnosis | ICD-10-CM |
| I69.363 | Other paralytic syndrome following cerebral infarction affecting right non-dominant side | Diagnosis | ICD-10-CM |
| I69.364 | Other paralytic syndrome following cerebral infarction affecting left non-dominant side | Diagnosis | ICD-10-CM |
| I69.369 | Other paralytic syndrome following cerebral infarction affecting unspecified side | Diagnosis | ICD-10-CM |
| I69.390 | Apraxia following cerebral infarction | Diagnosis | ICD-10-CM |
| I69.391 | Dysphagia following cerebral infarction | Diagnosis | ICD-10-CM |
| I69.392 | Facial weakness following cerebral infarction | Diagnosis | ICD-10-CM |
| I69.393 | Ataxia following cerebral infarction | Diagnosis | ICD-10-CM |
| I69.398 | Other sequelae of cerebral infarction | Diagnosis | ICD-10-CM |
| I69.80 | Unspecified sequelae of other cerebrovascular disease | Diagnosis | ICD-10-CM |
| I69.810 | Attention and concentration deficit following other cerebrovascular disease | Diagnosis | ICD-10-CM |
| I69.811 | Memory deficit following other cerebrovascular disease | Diagnosis | ICD-10-CM |
| I69.812 | Visuospatial deficit and spatial neglect following other cerebrovascular disease | Diagnosis | ICD-10-CM |
| I69.813 | Psychomotor deficit following other cerebrovascular disease | Diagnosis | ICD-10-CM |
| I69.814 | Frontal lobe and executive function deficit following other cerebrovascular disease | Diagnosis | ICD-10-CM |
| I69.815 | Cognitive social or emotional deficit following other cerebrovascular disease | Diagnosis | ICD-10-CM |
| I69.818 | Other symptoms and signs involving cognitive functions following other cerebrovascular disease | Diagnosis | ICD-10-CM |
| I69.819 | Unspecified symptoms and signs involving cognitive functions following other cerebrovascular disease | Diagnosis | ICD-10-CM |
| I69.820 | Aphasia following other cerebrovascular disease | Diagnosis | ICD-10-CM |
| I69.821 | Dysphasia following other cerebrovascular disease | Diagnosis | ICD-10-CM |
| I69.822 | Dysarthria following other cerebrovascular disease | Diagnosis | ICD-10-CM |
| I69.823 | Fluency disorder following other cerebrovascular disease | Diagnosis | ICD-10-CM |
| I69.828 | Other speech and language deficits following other cerebrovascular disease | Diagnosis | ICD-10-CM |
| I69.831 | Monoplegia of upper limb following other cerebrovascular disease affecting right dominant side | Diagnosis | ICD-10-CM |
| I69.832 | Monoplegia of upper limb following other cerebrovascular disease affecting left dominant side | Diagnosis | ICD-10-CM |
| I69.833 | Monoplegia of upper limb following other cerebrovascular disease affecting right non-dominant side | Diagnosis | ICD-10-CM |
| I69.834 | Monoplegia of upper limb following other cerebrovascular disease affecting left non-dominant side | Diagnosis | ICD-10-CM |
| I69.839 | Monoplegia of upper limb following other cerebrovascular disease affecting unspecified side | Diagnosis | ICD-10-CM |
| I69.841 | Monoplegia of lower limb following other cerebrovascular disease affecting right dominant side | Diagnosis | ICD-10-CM |
| I69.842 | Monoplegia of lower limb following other cerebrovascular disease affecting left dominant side | Diagnosis | ICD-10-CM |
| I69.843 | Monoplegia of lower limb following other cerebrovascular disease affecting right non-dominant side | Diagnosis | ICD-10-CM |
| I69.844 | Monoplegia of lower limb following other cerebrovascular disease affecting left non-dominant side | Diagnosis | ICD-10-CM |
| I69.849 | Monoplegia of lower limb following other cerebrovascular disease affecting unspecified side | Diagnosis | ICD-10-CM |
| I69.851 | Hemiplegia and hemiparesis following other cerebrovascular disease affecting right dominant side | Diagnosis | ICD-10-CM |
| I69.852 | Hemiplegia and hemiparesis following other cerebrovascular disease affecting left dominant side | Diagnosis | ICD-10-CM |
| I69.853 | Hemiplegia and hemiparesis following other cerebrovascular disease affecting right non-dominant side | Diagnosis | ICD-10-CM |
| I69.854 | Hemiplegia and hemiparesis following other cerebrovascular disease affecting left non-dominant side | Diagnosis | ICD-10-CM |
| I69.859 | Hemiplegia and hemiparesis following other cerebrovascular disease affecting unspecified side | Diagnosis | ICD-10-CM |
| I69.861 | Other paralytic syndrome following other cerebrovascular disease affecting right dominant side | Diagnosis | ICD-10-CM |
| I69.862 | Other paralytic syndrome following other cerebrovascular disease affecting left dominant side | Diagnosis | ICD-10-CM |
| I69.863 | Other paralytic syndrome following other cerebrovascular disease affecting right non-dominant side | Diagnosis | ICD-10-CM |
| I69.864 | Other paralytic syndrome following other cerebrovascular disease affecting left non-dominant side | Diagnosis | ICD-10-CM |
| I69.869 | Other paralytic syndrome following other cerebrovascular disease affecting unspecified side | Diagnosis | ICD-10-CM |
| I69.890 | Apraxia following other cerebrovascular disease | Diagnosis | ICD-10-CM |
| I69.891 | Dysphagia following other cerebrovascular disease | Diagnosis | ICD-10-CM |
| I69.892 | Facial weakness following other cerebrovascular disease | Diagnosis | ICD-10-CM |
| I69.893 | Ataxia following other cerebrovascular disease | Diagnosis | ICD-10-CM |
| I69.898 | Other sequelae of other cerebrovascular disease | Diagnosis | ICD-10-CM |
| I69.90 | Unspecified sequelae of unspecified cerebrovascular disease | Diagnosis | ICD-10-CM |
| I69.910 | Attention and concentration deficit following unspecified cerebrovascular disease | Diagnosis | ICD-10-CM |
| I69.911 | Memory deficit following unspecified cerebrovascular disease | Diagnosis | ICD-10-CM |
| I69.912 | Visuospatial deficit and spatial neglect following unspecified cerebrovascular disease | Diagnosis | ICD-10-CM |
| I69.913 | Psychomotor deficit following unspecified cerebrovascular disease | Diagnosis | ICD-10-CM |
| I69.914 | Frontal lobe and executive function deficit following unspecified cerebrovascular disease | Diagnosis | ICD-10-CM |
| I69.915 | Cognitive social or emotional deficit following unspecified cerebrovascular disease | Diagnosis | ICD-10-CM |
| I69.918 | Other symptoms and signs involving cognitive functions following unspecified cerebrovascular disease | Diagnosis | ICD-10-CM |
| I69.919 | Unspecified symptoms and signs involving cognitive functions following unspecified cerebrovascular disease | Diagnosis | ICD-10-CM |
| I69.920 | Aphasia following unspecified cerebrovascular disease | Diagnosis | ICD-10-CM |
| I69.921 | Dysphasia following unspecified cerebrovascular disease | Diagnosis | ICD-10-CM |
| I69.922 | Dysarthria following unspecified cerebrovascular disease | Diagnosis | ICD-10-CM |
| I69.923 | Fluency disorder following unspecified cerebrovascular disease | Diagnosis | ICD-10-CM |
| I69.928 | Other speech and language deficits following unspecified cerebrovascular disease | Diagnosis | ICD-10-CM |
| I69.931 | Monoplegia of upper limb following unspecified cerebrovascular disease affecting right dominant side | Diagnosis | ICD-10-CM |
| I69.932 | Monoplegia of upper limb following unspecified cerebrovascular disease affecting left dominant side | Diagnosis | ICD-10-CM |
| I69.933 | Monoplegia of upper limb following unspecified cerebrovascular disease affecting right non-dominant side | Diagnosis | ICD-10-CM |
| I69.934 | Monoplegia of upper limb following unspecified cerebrovascular disease affecting left non-dominant side | Diagnosis | ICD-10-CM |
| I69.939 | Monoplegia of upper limb following unspecified cerebrovascular disease affecting unspecified side | Diagnosis | ICD-10-CM |
| I69.941 | Monoplegia of lower limb following unspecified cerebrovascular disease affecting right dominant side | Diagnosis | ICD-10-CM |
| I69.942 | Monoplegia of lower limb following unspecified cerebrovascular disease affecting left dominant side | Diagnosis | ICD-10-CM |
| I69.943 | Monoplegia of lower limb following unspecified cerebrovascular disease affecting right non-dominant side | Diagnosis | ICD-10-CM |
| I69.944 | Monoplegia of lower limb following unspecified cerebrovascular disease affecting left non-dominant side | Diagnosis | ICD-10-CM |
| I69.949 | Monoplegia of lower limb following unspecified cerebrovascular disease affecting unspecified side | Diagnosis | ICD-10-CM |
| I69.951 | Hemiplegia and hemiparesis following unspecified cerebrovascular disease affecting right dominant side | Diagnosis | ICD-10-CM |
| I69.952 | Hemiplegia and hemiparesis following unspecified cerebrovascular disease affecting left dominant side | Diagnosis | ICD-10-CM |
| I69.953 | Hemiplegia and hemiparesis following unspecified cerebrovascular disease affecting right non-dominant side | Diagnosis | ICD-10-CM |
| I69.954 | Hemiplegia and hemiparesis following unspecified cerebrovascular disease affecting left non-dominant side | Diagnosis | ICD-10-CM |
| I69.959 | Hemiplegia and hemiparesis following unspecified cerebrovascular disease affecting unspecified side | Diagnosis | ICD-10-CM |
| I69.961 | Other paralytic syndrome following unspecified cerebrovascular disease affecting right dominant side | Diagnosis | ICD-10-CM |
| I69.962 | Other paralytic syndrome following unspecified cerebrovascular disease affecting left dominant side | Diagnosis | ICD-10-CM |
| I69.963 | Other paralytic syndrome following unspecified cerebrovascular disease affecting right non-dominant side | Diagnosis | ICD-10-CM |
| I69.964 | Other paralytic syndrome following unspecified cerebrovascular disease affecting left non-dominant side | Diagnosis | ICD-10-CM |
| I69.969 | Other paralytic syndrome following unspecified cerebrovascular disease affecting unspecified side | Diagnosis | ICD-10-CM |
| I69.990 | Apraxia following unspecified cerebrovascular disease | Diagnosis | ICD-10-CM |
| I69.991 | Dysphagia following unspecified cerebrovascular disease | Diagnosis | ICD-10-CM |
| I69.992 | Facial weakness following unspecified cerebrovascular disease | Diagnosis | ICD-10-CM |
| I69.993 | Ataxia following unspecified cerebrovascular disease | Diagnosis | ICD-10-CM |
| I69.998 | Other sequelae following unspecified cerebrovascular disease | Diagnosis | ICD-10-CM |
| 61645 | Percutaneous arterial transluminal mechanical thrombectomy and/or infusion for thrombolysis, intracranial, any method, including diagnostic angiography, fluoroscopic guidance, catheter placement, and intraprocedural pharmacological thrombolytic injection(s) | Procedure | CPT-4 |
| **Sepsis** | | | |
| A02.1 | Salmonella sepsis | Diagnosis | ICD-10-CM |
| A22.7 | Anthrax sepsis | Diagnosis | ICD-10-CM |
| A26.7 | Erysipelothrix sepsis | Diagnosis | ICD-10-CM |
| A32.7 | Listerial sepsis | Diagnosis | ICD-10-CM |
| A40.0 | Sepsis due to streptococcus, group A | Diagnosis | ICD-10-CM |
| A40.1 | Sepsis due to streptococcus, group B | Diagnosis | ICD-10-CM |
| A40.3 | Sepsis due to Streptococcus pneumoniae | Diagnosis | ICD-10-CM |
| A40.8 | Other streptococcal sepsis | Diagnosis | ICD-10-CM |
| A40.9 | Streptococcal sepsis, unspecified | Diagnosis | ICD-10-CM |
| A41.0 | Sepsis due to Staphylococcus aureus | Diagnosis | ICD-10-CM |
| A41.01 | Sepsis due to Methicillin susceptible Staphylococcus aureus | Diagnosis | ICD-10-CM |
| A41.02 | Sepsis due to Methicillin resistant Staphylococcus aureus | Diagnosis | ICD-10-CM |
| A41.0Z16 | Sepsis due to Staphylococcus aureusInfection with drug resistant microorganisms | Diagnosis | ICD-10-CM |
| A41.1 | Sepsis due to other specified staphylococcus | Diagnosis | ICD-10-CM |
| A41.2 | Sepsis due to unspecified staphylococcus | Diagnosis | ICD-10-CM |
| A41.3 | Sepsis due to Hemophilus influenzae | Diagnosis | ICD-10-CM |
| A41.4 | Sepsis due to anaerobes | Diagnosis | ICD-10-CM |
| A41.50 | Gram-negative sepsis, unspecified | Diagnosis | ICD-10-CM |
| A41.51 | Sepsis due to Escherichia coli [E. coli] | Diagnosis | ICD-10-CM |
| A41.52 | Sepsis due to Pseudomonas | Diagnosis | ICD-10-CM |
| A41.53 | Sepsis due to Serratia | Diagnosis | ICD-10-CM |
| A41.59 | Other Gram-negative sepsis | Diagnosis | ICD-10-CM |
| A41.81 | Sepsis due to Enterococcus | Diagnosis | ICD-10-CM |
| A41.89 | Other specified sepsis | Diagnosis | ICD-10-CM |
| A41.9 | Sepsis, unspecified organism | Diagnosis | ICD-10-CM |
| A42.7 | Actinomycotic sepsis | Diagnosis | ICD-10-CM |
| A54.86 | Gonococcal sepsis | Diagnosis | ICD-10-CM |
| B37.7 | Candidal sepsis | Diagnosis | ICD-10-CM |
| R65.20 | Severe sepsis without septic shock | Diagnosis | ICD-10-CM |
| R65.21 | Severe sepsis with septic shock | Diagnosis | ICD-10-CM |
| A40 | Streptococcal sepsis | Diagnosis | ICD-10-CM |
| A41 | Other sepsis | Diagnosis | ICD-10-CM |
| A41.5 | Sepsis due to other Gram-negative organisms | Diagnosis | ICD-10-CM |
| A41.8 | Other specified sepsis | Diagnosis | ICD-10-CM |
| O03.37 | Sepsis following incomplete spontaneous abortion | Diagnosis | ICD-10-CM |
| O03.87 | Sepsis following complete or unspecified spontaneous abortion | Diagnosis | ICD-10-CM |
| O04.87 | Sepsis following (induced) termination of pregnancy | Diagnosis | ICD-10-CM |
| O07.37 | Sepsis following failed attempted termination of pregnancy | Diagnosis | ICD-10-CM |
| O08.82 | Sepsis following ectopic and molar pregnancy | Diagnosis | ICD-10-CM |
| O85 | Puerperal sepsis | Diagnosis | ICD-10-CM |
| O86.04 | Sepsis following an obstetrical procedure | Diagnosis | ICD-10-CM |
| P36 | Bacterial sepsis of newborn | Diagnosis | ICD-10-CM |
| P36.0 | Sepsis of newborn due to streptococcus, group B | Diagnosis | ICD-10-CM |
| P36.1 | Sepsis of newborn due to other and unspecified streptococci | Diagnosis | ICD-10-CM |
| P36.10 | Sepsis of newborn due to unspecified streptococci | Diagnosis | ICD-10-CM |
| P36.19 | Sepsis of newborn due to other streptococci | Diagnosis | ICD-10-CM |
| P36.2 | Sepsis of newborn due to Staphylococcus aureus | Diagnosis | ICD-10-CM |
| P36.3 | Sepsis of newborn due to other and unspecified staphylococci | Diagnosis | ICD-10-CM |
| P36.30 | Sepsis of newborn due to unspecified staphylococci | Diagnosis | ICD-10-CM |
| P36.39 | Sepsis of newborn due to other staphylococci | Diagnosis | ICD-10-CM |
| P36.4 | Sepsis of newborn due to Escherichia coli | Diagnosis | ICD-10-CM |
| P36.5 | Sepsis of newborn due to anaerobes | Diagnosis | ICD-10-CM |
| P36.8 | Other bacterial sepsis of newborn | Diagnosis | ICD-10-CM |
| P36.9 | Bacterial sepsis of newborn, unspecified | Diagnosis | ICD-10-CM |
| R65.2 | Severe sepsis | Diagnosis | ICD-10-CM |
| T81.44 | Sepsis following a procedure | Diagnosis | ICD-10-CM |
| T81.44XA | Sepsis following a procedure, initial encounter | Diagnosis | ICD-10-CM |
| T81.44XD | Sepsis following a procedure, subsequent encounter | Diagnosis | ICD-10-CM |
| T81.44XS | Sepsis following a procedure, sequela | Diagnosis | ICD-10-CM |
| **Asthma** | | | |
| J45.20 | Mild intermittent asthma, uncomplicated | Diagnosis | ICD-10-CM |
| J45.21 | Mild intermittent asthma with (acute) exacerbation | Diagnosis | ICD-10-CM |
| J45.22 | Mild intermittent asthma with status asthmaticus | Diagnosis | ICD-10-CM |
| J45.30 | Mild persistent asthma, uncomplicated | Diagnosis | ICD-10-CM |
| J45.31 | Mild persistent asthma with (acute) exacerbation | Diagnosis | ICD-10-CM |
| J45.32 | Mild persistent asthma with status asthmaticus | Diagnosis | ICD-10-CM |
| J45.40 | Moderate persistent asthma, uncomplicated | Diagnosis | ICD-10-CM |
| J45.41 | Moderate persistent asthma with (acute) exacerbation | Diagnosis | ICD-10-CM |
| J45.42 | Moderate persistent asthma with status asthmaticus | Diagnosis | ICD-10-CM |
| J45.50 | Severe persistent asthma, uncomplicated | Diagnosis | ICD-10-CM |
| J45.51 | Severe persistent asthma with (acute) exacerbation | Diagnosis | ICD-10-CM |
| J45.52 | Severe persistent asthma with status asthmaticus | Diagnosis | ICD-10-CM |
| J45.901 | Unspecified asthma with (acute) exacerbation | Diagnosis | ICD-10-CM |
| J45.902 | Unspecified asthma with status asthmaticus | Diagnosis | ICD-10-CM |
| J45.909 | Unspecified asthma, uncomplicated | Diagnosis | ICD-10-CM |
| J45.990 | Exercise induced bronchospasm | Diagnosis | ICD-10-CM |
| J45.991 | Cough variant asthma | Diagnosis | ICD-10-CM |
| J45.998 | Other asthma | Diagnosis | ICD-10-CM |
| **Chronic Obstructive Pulmonary Disease** | | | |
| J40 | Bronchitis, not specified as acute or chronic | Diagnosis | ICD-10-CM |
| J41.0 | Simple chronic bronchitis | Diagnosis | ICD-10-CM |
| J41.1 | Mucopurulent chronic bronchitis | Diagnosis | ICD-10-CM |
| J41.8 | Mixed simple and mucopurulent chronic bronchitis | Diagnosis | ICD-10-CM |
| J42 | Unspecified chronic bronchitis | Diagnosis | ICD-10-CM |
| J43.0 | Unilateral pulmonary emphysema [MacLeod's syndrome] | Diagnosis | ICD-10-CM |
| J43.1 | Panlobular emphysema | Diagnosis | ICD-10-CM |
| J43.2 | Centrilobular emphysema | Diagnosis | ICD-10-CM |
| J43.8 | Other emphysema | Diagnosis | ICD-10-CM |
| J43.9 | Emphysema, unspecified | Diagnosis | ICD-10-CM |
| J44.0 | Chronic obstructive pulmonary disease with acute lower respiratory infection | Diagnosis | ICD-10-CM |
| J44.1 | Chronic obstructive pulmonary disease with (acute) exacerbation | Diagnosis | ICD-10-CM |
| J44.9 | Chronic obstructive pulmonary disease, unspecified | Diagnosis | ICD-10-CM |
| J47.0 | Bronchiectasis with acute lower respiratory infection | Diagnosis | ICD-10-CM |
| J47.1 | Bronchiectasis with (acute) exacerbation | Diagnosis | ICD-10-CM |
| J47.9 | Bronchiectasis, uncomplicated | Diagnosis | ICD-10-CM |
| J67.0 | Farmer's lung | Diagnosis | ICD-10-CM |
| J67.1 | Bagassosis | Diagnosis | ICD-10-CM |
| J67.2 | Bird fancier's lung | Diagnosis | ICD-10-CM |
| J67.3 | Suberosis | Diagnosis | ICD-10-CM |
| J67.4 | Maltworker's lung | Diagnosis | ICD-10-CM |
| J67.5 | Mushroom-worker's lung | Diagnosis | ICD-10-CM |
| J67.6 | Maple-bark-stripper's lung | Diagnosis | ICD-10-CM |
| J67.7 | Air conditioner and humidifier lung | Diagnosis | ICD-10-CM |
| J67.8 | Hypersensitivity pneumonitis due to other organic dusts | Diagnosis | ICD-10-CM |
| J67.9 | Hypersensitivity pneumonitis due to unspecified organic dust | Diagnosis | ICD-10-CM |
| **Diabetes** | | | |
| E10.10 | Type 1 diabetes mellitus with ketoacidosis without coma | Diagnosis | ICD-10-CM |
| E10.11 | Type 1 diabetes mellitus with ketoacidosis with coma | Diagnosis | ICD-10-CM |
| E10.21 | Type 1 diabetes mellitus with diabetic nephropathy | Diagnosis | ICD-10-CM |
| E10.22 | Type 1 diabetes mellitus with diabetic chronic kidney disease | Diagnosis | ICD-10-CM |
| E10.29 | Type 1 diabetes mellitus with other diabetic kidney complication | Diagnosis | ICD-10-CM |
| E10.311 | Type 1 diabetes mellitus with unspecified diabetic retinopathy with macular edema | Diagnosis | ICD-10-CM |
| E10.319 | Type 1 diabetes mellitus with unspecified diabetic retinopathy without macular edema | Diagnosis | ICD-10-CM |
| E10.3211 | Type 1 diabetes mellitus with mild nonproliferative diabetic retinopathy with macular edema, right eye | Diagnosis | ICD-10-CM |
| E10.3212 | Type 1 diabetes mellitus with mild nonproliferative diabetic retinopathy with macular edema, left eye | Diagnosis | ICD-10-CM |
| E10.3213 | Type 1 diabetes mellitus with mild nonproliferative diabetic retinopathy with macular edema, bilateral | Diagnosis | ICD-10-CM |
| E10.3219 | Type 1 diabetes mellitus with mild nonproliferative diabetic retinopathy with macular edema, unspecified eye | Diagnosis | ICD-10-CM |
| E10.3291 | Type 1 diabetes mellitus with mild nonproliferative diabetic retinopathy without macular edema, right eye | Diagnosis | ICD-10-CM |
| E10.3292 | Type 1 diabetes mellitus with mild nonproliferative diabetic retinopathy without macular edema, left eye | Diagnosis | ICD-10-CM |
| E10.3293 | Type 1 diabetes mellitus with mild nonproliferative diabetic retinopathy without macular edema, bilateral | Diagnosis | ICD-10-CM |
| E10.3299 | Type 1 diabetes mellitus with mild nonproliferative diabetic retinopathy without macular edema, unspecified eye | Diagnosis | ICD-10-CM |
| E10.3311 | Type 1 diabetes mellitus with moderate nonproliferative diabetic retinopathy with macular edema, right eye | Diagnosis | ICD-10-CM |
| E10.3312 | Type 1 diabetes mellitus with moderate nonproliferative diabetic retinopathy with macular edema, left eye | Diagnosis | ICD-10-CM |
| E10.3313 | Type 1 diabetes mellitus with moderate nonproliferative diabetic retinopathy with macular edema, bilateral | Diagnosis | ICD-10-CM |
| E10.3319 | Type 1 diabetes mellitus with moderate nonproliferative diabetic retinopathy with macular edema, unspecified eye | Diagnosis | ICD-10-CM |
| E10.3391 | Type 1 diabetes mellitus with moderate nonproliferative diabetic retinopathy without macular edema, right eye | Diagnosis | ICD-10-CM |
| E10.3392 | Type 1 diabetes mellitus with moderate nonproliferative diabetic retinopathy without macular edema, left eye | Diagnosis | ICD-10-CM |
| E10.3393 | Type 1 diabetes mellitus with moderate nonproliferative diabetic retinopathy without macular edema, bilateral | Diagnosis | ICD-10-CM |
| E10.3399 | Type 1 diabetes mellitus with moderate nonproliferative diabetic retinopathy without macular edema, unspecified eye | Diagnosis | ICD-10-CM |
| E10.3411 | Type 1 diabetes mellitus with severe nonproliferative diabetic retinopathy with macular edema, right eye | Diagnosis | ICD-10-CM |
| E10.3412 | Type 1 diabetes mellitus with severe nonproliferative diabetic retinopathy with macular edema, left eye | Diagnosis | ICD-10-CM |
| E10.3413 | Type 1 diabetes mellitus with severe nonproliferative diabetic retinopathy with macular edema, bilateral | Diagnosis | ICD-10-CM |
| E10.3419 | Type 1 diabetes mellitus with severe nonproliferative diabetic retinopathy with macular edema, unspecified eye | Diagnosis | ICD-10-CM |
| E10.3491 | Type 1 diabetes mellitus with severe nonproliferative diabetic retinopathy without macular edema, right eye | Diagnosis | ICD-10-CM |
| E10.3492 | Type 1 diabetes mellitus with severe nonproliferative diabetic retinopathy without macular edema, left eye | Diagnosis | ICD-10-CM |
| E10.3493 | Type 1 diabetes mellitus with severe nonproliferative diabetic retinopathy without macular edema, bilateral | Diagnosis | ICD-10-CM |
| E10.3499 | Type 1 diabetes mellitus with severe nonproliferative diabetic retinopathy without macular edema, unspecified eye | Diagnosis | ICD-10-CM |
| E10.3511 | Type 1 diabetes mellitus with proliferative diabetic retinopathy with macular edema, right eye | Diagnosis | ICD-10-CM |
| E10.3512 | Type 1 diabetes mellitus with proliferative diabetic retinopathy with macular edema, left eye | Diagnosis | ICD-10-CM |
| E10.3513 | Type 1 diabetes mellitus with proliferative diabetic retinopathy with macular edema, bilateral | Diagnosis | ICD-10-CM |
| E10.3519 | Type 1 diabetes mellitus with proliferative diabetic retinopathy with macular edema, unspecified eye | Diagnosis | ICD-10-CM |
| E10.3521 | Type 1 diabetes mellitus with proliferative diabetic retinopathy with traction retinal detachment involving the macula, right eye | Diagnosis | ICD-10-CM |
| E10.3522 | Type 1 diabetes mellitus with proliferative diabetic retinopathy with traction retinal detachment involving the macula, left eye | Diagnosis | ICD-10-CM |
| E10.3523 | Type 1 diabetes mellitus with proliferative diabetic retinopathy with traction retinal detachment involving the macula, bilateral | Diagnosis | ICD-10-CM |
| E10.3529 | Type 1 diabetes mellitus with proliferative diabetic retinopathy with traction retinal detachment involving the macula, unspecified eye | Diagnosis | ICD-10-CM |
| E10.3531 | Type 1 diabetes mellitus with proliferative diabetic retinopathy with traction retinal detachment not involving the macula, right eye | Diagnosis | ICD-10-CM |
| E10.3532 | Type 1 diabetes mellitus with proliferative diabetic retinopathy with traction retinal detachment not involving the macula, left eye | Diagnosis | ICD-10-CM |
| E10.3533 | Type 1 diabetes mellitus with proliferative diabetic retinopathy with traction retinal detachment not involving the macula, bilateral | Diagnosis | ICD-10-CM |
| E10.3539 | Type 1 diabetes mellitus with proliferative diabetic retinopathy with traction retinal detachment not involving the macula, unspecified eye | Diagnosis | ICD-10-CM |
| E10.3541 | Type 1 diabetes mellitus with proliferative diabetic retinopathy with combined traction retinal detachment and rhegmatogenous retinal detachment, right eye | Diagnosis | ICD-10-CM |
| E10.3542 | Type 1 diabetes mellitus with proliferative diabetic retinopathy with combined traction retinal detachment and rhegmatogenous retinal detachment, left eye | Diagnosis | ICD-10-CM |
| E10.3543 | Type 1 diabetes mellitus with proliferative diabetic retinopathy with combined traction retinal detachment and rhegmatogenous retinal detachment, bilateral | Diagnosis | ICD-10-CM |
| E10.3549 | Type 1 diabetes mellitus with proliferative diabetic retinopathy with combined traction retinal detachment and rhegmatogenous retinal detachment, unspecified eye | Diagnosis | ICD-10-CM |
| E10.3551 | Type 1 diabetes mellitus with stable proliferative diabetic retinopathy, right eye | Diagnosis | ICD-10-CM |
| E10.3552 | Type 1 diabetes mellitus with stable proliferative diabetic retinopathy, left eye | Diagnosis | ICD-10-CM |
| E10.3553 | Type 1 diabetes mellitus with stable proliferative diabetic retinopathy, bilateral | Diagnosis | ICD-10-CM |
| E10.3559 | Type 1 diabetes mellitus with stable proliferative diabetic retinopathy, unspecified eye | Diagnosis | ICD-10-CM |
| E10.3591 | Type 1 diabetes mellitus with proliferative diabetic retinopathy without macular edema, right eye | Diagnosis | ICD-10-CM |
| E10.3592 | Type 1 diabetes mellitus with proliferative diabetic retinopathy without macular edema, left eye | Diagnosis | ICD-10-CM |
| E10.3593 | Type 1 diabetes mellitus with proliferative diabetic retinopathy without macular edema, bilateral | Diagnosis | ICD-10-CM |
| E10.3599 | Type 1 diabetes mellitus with proliferative diabetic retinopathy without macular edema, unspecified eye | Diagnosis | ICD-10-CM |
| E10.36 | Type 1 diabetes mellitus with diabetic cataract | Diagnosis | ICD-10-CM |
| E10.37X1 | Type 1 diabetes mellitus with diabetic macular edema, resolved following treatment, right eye | Diagnosis | ICD-10-CM |
| E10.37X2 | Type 1 diabetes mellitus with diabetic macular edema, resolved following treatment, left eye | Diagnosis | ICD-10-CM |
| E10.37X3 | Type 1 diabetes mellitus with diabetic macular edema, resolved following treatment, bilateral | Diagnosis | ICD-10-CM |
| E10.37X9 | Type 1 diabetes mellitus with diabetic macular edema, resolved following treatment, unspecified eye | Diagnosis | ICD-10-CM |
| E10.39 | Type 1 diabetes mellitus with other diabetic ophthalmic complication | Diagnosis | ICD-10-CM |
| E10.40 | Type 1 diabetes mellitus with diabetic neuropathy, unspecified | Diagnosis | ICD-10-CM |
| E10.41 | Type 1 diabetes mellitus with diabetic mononeuropathy | Diagnosis | ICD-10-CM |
| E10.42 | Type 1 diabetes mellitus with diabetic polyneuropathy | Diagnosis | ICD-10-CM |
| E10.43 | Type 1 diabetes mellitus with diabetic autonomic (poly)neuropathy | Diagnosis | ICD-10-CM |
| E10.44 | Type 1 diabetes mellitus with diabetic amyotrophy | Diagnosis | ICD-10-CM |
| E10.49 | Type 1 diabetes mellitus with other diabetic neurological complication | Diagnosis | ICD-10-CM |
| E10.51 | Type 1 diabetes mellitus with diabetic peripheral angiopathy without gangrene | Diagnosis | ICD-10-CM |
| E10.52 | Type 1 diabetes mellitus with diabetic peripheral angiopathy with gangrene | Diagnosis | ICD-10-CM |
| E10.59 | Type 1 diabetes mellitus with other circulatory complications | Diagnosis | ICD-10-CM |
| E10.610 | Type 1 diabetes mellitus with diabetic neuropathic arthropathy | Diagnosis | ICD-10-CM |
| E10.618 | Type 1 diabetes mellitus with other diabetic arthropathy | Diagnosis | ICD-10-CM |
| E10.620 | Type 1 diabetes mellitus with diabetic dermatitis | Diagnosis | ICD-10-CM |
| E10.621 | Type 1 diabetes mellitus with foot ulcer | Diagnosis | ICD-10-CM |
| E10.622 | Type 1 diabetes mellitus with other skin ulcer | Diagnosis | ICD-10-CM |
| E10.628 | Type 1 diabetes mellitus with other skin complications | Diagnosis | ICD-10-CM |
| E10.630 | Type 1 diabetes mellitus with periodontal disease | Diagnosis | ICD-10-CM |
| E10.638 | Type 1 diabetes mellitus with other oral complications | Diagnosis | ICD-10-CM |
| E10.641 | Type 1 diabetes mellitus with hypoglycemia with coma | Diagnosis | ICD-10-CM |
| E10.649 | Type 1 diabetes mellitus with hypoglycemia without coma | Diagnosis | ICD-10-CM |
| E10.65 | Type 1 diabetes mellitus with hyperglycemia | Diagnosis | ICD-10-CM |
| E10.69 | Type 1 diabetes mellitus with other specified complication | Diagnosis | ICD-10-CM |
| E10.8 | Type 1 diabetes mellitus with unspecified complications | Diagnosis | ICD-10-CM |
| E10.9 | Type 1 diabetes mellitus without complications | Diagnosis | ICD-10-CM |
| E11.00 | Type 2 diabetes mellitus with hyperosmolarity without nonketotic hyperglycemic-hyperosmolar coma (NKHHC) | Diagnosis | ICD-10-CM |
| E11.01 | Type 2 diabetes mellitus with hyperosmolarity with coma | Diagnosis | ICD-10-CM |
| E11.10 | Type 2 diabetes mellitus with ketoacidosis without coma | Diagnosis | ICD-10-CM |
| E11.11 | Type 2 diabetes mellitus with ketoacidosis with coma | Diagnosis | ICD-10-CM |
| E11.21 | Type 2 diabetes mellitus with diabetic nephropathy | Diagnosis | ICD-10-CM |
| E11.22 | Type 2 diabetes mellitus with diabetic chronic kidney disease | Diagnosis | ICD-10-CM |
| E11.29 | Type 2 diabetes mellitus with other diabetic kidney complication | Diagnosis | ICD-10-CM |
| E11.311 | Type 2 diabetes mellitus with unspecified diabetic retinopathy with macular edema | Diagnosis | ICD-10-CM |
| E11.319 | Type 2 diabetes mellitus with unspecified diabetic retinopathy without macular edema | Diagnosis | ICD-10-CM |
| E11.3211 | Type 2 diabetes mellitus with mild nonproliferative diabetic retinopathy with macular edema, right eye | Diagnosis | ICD-10-CM |
| E11.3212 | Type 2 diabetes mellitus with mild nonproliferative diabetic retinopathy with macular edema, left eye | Diagnosis | ICD-10-CM |
| E11.3213 | Type 2 diabetes mellitus with mild nonproliferative diabetic retinopathy with macular edema, bilateral | Diagnosis | ICD-10-CM |
| E11.3219 | Type 2 diabetes mellitus with mild nonproliferative diabetic retinopathy with macular edema, unspecified eye | Diagnosis | ICD-10-CM |
| E11.3291 | Type 2 diabetes mellitus with mild nonproliferative diabetic retinopathy without macular edema, right eye | Diagnosis | ICD-10-CM |
| E11.3292 | Type 2 diabetes mellitus with mild nonproliferative diabetic retinopathy without macular edema, left eye | Diagnosis | ICD-10-CM |
| E11.3293 | Type 2 diabetes mellitus with mild nonproliferative diabetic retinopathy without macular edema, bilateral | Diagnosis | ICD-10-CM |
| E11.3299 | Type 2 diabetes mellitus with mild nonproliferative diabetic retinopathy without macular edema, unspecified eye | Diagnosis | ICD-10-CM |
| E11.3311 | Type 2 diabetes mellitus with moderate nonproliferative diabetic retinopathy with macular edema, right eye | Diagnosis | ICD-10-CM |
| E11.3312 | Type 2 diabetes mellitus with moderate nonproliferative diabetic retinopathy with macular edema, left eye | Diagnosis | ICD-10-CM |
| E11.3313 | Type 2 diabetes mellitus with moderate nonproliferative diabetic retinopathy with macular edema, bilateral | Diagnosis | ICD-10-CM |
| E11.3319 | Type 2 diabetes mellitus with moderate nonproliferative diabetic retinopathy with macular edema, unspecified eye | Diagnosis | ICD-10-CM |
| E11.3391 | Type 2 diabetes mellitus with moderate nonproliferative diabetic retinopathy without macular edema, right eye | Diagnosis | ICD-10-CM |
| E11.3392 | Type 2 diabetes mellitus with moderate nonproliferative diabetic retinopathy without macular edema, left eye | Diagnosis | ICD-10-CM |
| E11.3393 | Type 2 diabetes mellitus with moderate nonproliferative diabetic retinopathy without macular edema, bilateral | Diagnosis | ICD-10-CM |
| E11.3399 | Type 2 diabetes mellitus with moderate nonproliferative diabetic retinopathy without macular edema, unspecified eye | Diagnosis | ICD-10-CM |
| E11.3411 | Type 2 diabetes mellitus with severe nonproliferative diabetic retinopathy with macular edema, right eye | Diagnosis | ICD-10-CM |
| E11.3412 | Type 2 diabetes mellitus with severe nonproliferative diabetic retinopathy with macular edema, left eye | Diagnosis | ICD-10-CM |
| E11.3413 | Type 2 diabetes mellitus with severe nonproliferative diabetic retinopathy with macular edema, bilateral | Diagnosis | ICD-10-CM |
| E11.3419 | Type 2 diabetes mellitus with severe nonproliferative diabetic retinopathy with macular edema, unspecified eye | Diagnosis | ICD-10-CM |
| E11.3491 | Type 2 diabetes mellitus with severe nonproliferative diabetic retinopathy without macular edema, right eye | Diagnosis | ICD-10-CM |
| E11.3492 | Type 2 diabetes mellitus with severe nonproliferative diabetic retinopathy without macular edema, left eye | Diagnosis | ICD-10-CM |
| E11.3493 | Type 2 diabetes mellitus with severe nonproliferative diabetic retinopathy without macular edema, bilateral | Diagnosis | ICD-10-CM |
| E11.3499 | Type 2 diabetes mellitus with severe nonproliferative diabetic retinopathy without macular edema, unspecified eye | Diagnosis | ICD-10-CM |
| E11.3511 | Type 2 diabetes mellitus with proliferative diabetic retinopathy with macular edema, right eye | Diagnosis | ICD-10-CM |
| E11.3512 | Type 2 diabetes mellitus with proliferative diabetic retinopathy with macular edema, left eye | Diagnosis | ICD-10-CM |
| E11.3513 | Type 2 diabetes mellitus with proliferative diabetic retinopathy with macular edema, bilateral | Diagnosis | ICD-10-CM |
| E11.3519 | Type 2 diabetes mellitus with proliferative diabetic retinopathy with macular edema, unspecified eye | Diagnosis | ICD-10-CM |
| E11.3521 | Type 2 diabetes mellitus with proliferative diabetic retinopathy with traction retinal detachment involving the macula, right eye | Diagnosis | ICD-10-CM |
| E11.3522 | Type 2 diabetes mellitus with proliferative diabetic retinopathy with traction retinal detachment involving the macula, left eye | Diagnosis | ICD-10-CM |
| E11.3523 | Type 2 diabetes mellitus with proliferative diabetic retinopathy with traction retinal detachment involving the macula, bilateral | Diagnosis | ICD-10-CM |
| E11.3529 | Type 2 diabetes mellitus with proliferative diabetic retinopathy with traction retinal detachment involving the macula, unspecified eye | Diagnosis | ICD-10-CM |
| E11.3531 | Type 2 diabetes mellitus with proliferative diabetic retinopathy with traction retinal detachment not involving the macula, right eye | Diagnosis | ICD-10-CM |
| E11.3532 | Type 2 diabetes mellitus with proliferative diabetic retinopathy with traction retinal detachment not involving the macula, left eye | Diagnosis | ICD-10-CM |
| E11.3533 | Type 2 diabetes mellitus with proliferative diabetic retinopathy with traction retinal detachment not involving the macula, bilateral | Diagnosis | ICD-10-CM |
| E11.3539 | Type 2 diabetes mellitus with proliferative diabetic retinopathy with traction retinal detachment not involving the macula, unspecified eye | Diagnosis | ICD-10-CM |
| E11.3541 | Type 2 diabetes mellitus with proliferative diabetic retinopathy with combined traction retinal detachment and rhegmatogenous retinal detachment, right eye | Diagnosis | ICD-10-CM |
| E11.3542 | Type 2 diabetes mellitus with proliferative diabetic retinopathy with combined traction retinal detachment and rhegmatogenous retinal detachment, left eye | Diagnosis | ICD-10-CM |
| E11.3543 | Type 2 diabetes mellitus with proliferative diabetic retinopathy with combined traction retinal detachment and rhegmatogenous retinal detachment, bilateral | Diagnosis | ICD-10-CM |
| E11.3549 | Type 2 diabetes mellitus with proliferative diabetic retinopathy with combined traction retinal detachment and rhegmatogenous retinal detachment, unspecified eye | Diagnosis | ICD-10-CM |
| E11.3551 | Type 2 diabetes mellitus with stable proliferative diabetic retinopathy, right eye | Diagnosis | ICD-10-CM |
| E11.3552 | Type 2 diabetes mellitus with stable proliferative diabetic retinopathy, left eye | Diagnosis | ICD-10-CM |
| E11.3553 | Type 2 diabetes mellitus with stable proliferative diabetic retinopathy, bilateral | Diagnosis | ICD-10-CM |
| E11.3559 | Type 2 diabetes mellitus with stable proliferative diabetic retinopathy, unspecified eye | Diagnosis | ICD-10-CM |
| E11.3591 | Type 2 diabetes mellitus with proliferative diabetic retinopathy without macular edema, right eye | Diagnosis | ICD-10-CM |
| E11.3592 | Type 2 diabetes mellitus with proliferative diabetic retinopathy without macular edema, left eye | Diagnosis | ICD-10-CM |
| E11.3593 | Type 2 diabetes mellitus with proliferative diabetic retinopathy without macular edema, bilateral | Diagnosis | ICD-10-CM |
| E11.3599 | Type 2 diabetes mellitus with proliferative diabetic retinopathy without macular edema, unspecified eye | Diagnosis | ICD-10-CM |
| E11.36 | Type 2 diabetes mellitus with diabetic cataract | Diagnosis | ICD-10-CM |
| E11.37X1 | Type 2 diabetes mellitus with diabetic macular edema, resolved following treatment, right eye | Diagnosis | ICD-10-CM |
| E11.37X2 | Type 2 diabetes mellitus with diabetic macular edema, resolved following treatment, left eye | Diagnosis | ICD-10-CM |
| E11.37X3 | Type 2 diabetes mellitus with diabetic macular edema, resolved following treatment, bilateral | Diagnosis | ICD-10-CM |
| E11.37X9 | Type 2 diabetes mellitus with diabetic macular edema, resolved following treatment, unspecified eye | Diagnosis | ICD-10-CM |
| E11.39 | Type 2 diabetes mellitus with other diabetic ophthalmic complication | Diagnosis | ICD-10-CM |
| E11.40 | Type 2 diabetes mellitus with diabetic neuropathy, unspecified | Diagnosis | ICD-10-CM |
| E11.41 | Type 2 diabetes mellitus with diabetic mononeuropathy | Diagnosis | ICD-10-CM |
| E11.42 | Type 2 diabetes mellitus with diabetic polyneuropathy | Diagnosis | ICD-10-CM |
| E11.43 | Type 2 diabetes mellitus with diabetic autonomic (poly)neuropathy | Diagnosis | ICD-10-CM |
| E11.44 | Type 2 diabetes mellitus with diabetic amyotrophy | Diagnosis | ICD-10-CM |
| E11.49 | Type 2 diabetes mellitus with other diabetic neurological complication | Diagnosis | ICD-10-CM |
| E11.51 | Type 2 diabetes mellitus with diabetic peripheral angiopathy without gangrene | Diagnosis | ICD-10-CM |
| E11.52 | Type 2 diabetes mellitus with diabetic peripheral angiopathy with gangrene | Diagnosis | ICD-10-CM |
| E11.59 | Type 2 diabetes mellitus with other circulatory complications | Diagnosis | ICD-10-CM |
| E11.610 | Type 2 diabetes mellitus with diabetic neuropathic arthropathy | Diagnosis | ICD-10-CM |
| E11.618 | Type 2 diabetes mellitus with other diabetic arthropathy | Diagnosis | ICD-10-CM |
| E11.620 | Type 2 diabetes mellitus with diabetic dermatitis | Diagnosis | ICD-10-CM |
| E11.621 | Type 2 diabetes mellitus with foot ulcer | Diagnosis | ICD-10-CM |
| E11.622 | Type 2 diabetes mellitus with other skin ulcer | Diagnosis | ICD-10-CM |
| E11.628 | Type 2 diabetes mellitus with other skin complications | Diagnosis | ICD-10-CM |
| E11.630 | Type 2 diabetes mellitus with periodontal disease | Diagnosis | ICD-10-CM |
| E11.638 | Type 2 diabetes mellitus with other oral complications | Diagnosis | ICD-10-CM |
| E11.641 | Type 2 diabetes mellitus with hypoglycemia with coma | Diagnosis | ICD-10-CM |
| E11.649 | Type 2 diabetes mellitus with hypoglycemia without coma | Diagnosis | ICD-10-CM |
| E11.65 | Type 2 diabetes mellitus with hyperglycemia | Diagnosis | ICD-10-CM |
| E11.69 | Type 2 diabetes mellitus with other specified complication | Diagnosis | ICD-10-CM |
| E11.8 | Type 2 diabetes mellitus with unspecified complications | Diagnosis | ICD-10-CM |
| E11.9 | Type 2 diabetes mellitus without complications | Diagnosis | ICD-10-CM |
| E13.00 | Other specified diabetes mellitus with hyperosmolarity without nonketotic hyperglycemic-hyperosmolar coma (NKHHC) | Diagnosis | ICD-10-CM |
| E13.01 | Other specified diabetes mellitus with hyperosmolarity with coma | Diagnosis | ICD-10-CM |
| E13.10 | Other specified diabetes mellitus with ketoacidosis without coma | Diagnosis | ICD-10-CM |
| E13.11 | Other specified diabetes mellitus with ketoacidosis with coma | Diagnosis | ICD-10-CM |
| E13.21 | Other specified diabetes mellitus with diabetic nephropathy | Diagnosis | ICD-10-CM |
| E13.22 | Other specified diabetes mellitus with diabetic chronic kidney disease | Diagnosis | ICD-10-CM |
| E13.29 | Other specified diabetes mellitus with other diabetic kidney complication | Diagnosis | ICD-10-CM |
| E13.311 | Other specified diabetes mellitus with unspecified diabetic retinopathy with macular edema | Diagnosis | ICD-10-CM |
| E13.319 | Other specified diabetes mellitus with unspecified diabetic retinopathy without macular edema | Diagnosis | ICD-10-CM |
| E13.3211 | Other specified diabetes mellitus with mild nonproliferative diabetic retinopathy with macular edema, right eye | Diagnosis | ICD-10-CM |
| E13.3212 | Other specified diabetes mellitus with mild nonproliferative diabetic retinopathy with macular edema, left eye | Diagnosis | ICD-10-CM |
| E13.3213 | Other specified diabetes mellitus with mild nonproliferative diabetic retinopathy with macular edema, bilateral | Diagnosis | ICD-10-CM |
| E13.3219 | Other specified diabetes mellitus with mild nonproliferative diabetic retinopathy with macular edema, unspecified eye | Diagnosis | ICD-10-CM |
| E13.3291 | Other specified diabetes mellitus with mild nonproliferative diabetic retinopathy without macular edema, right eye | Diagnosis | ICD-10-CM |
| E13.3292 | Other specified diabetes mellitus with mild nonproliferative diabetic retinopathy without macular edema, left eye | Diagnosis | ICD-10-CM |
| E13.3293 | Other specified diabetes mellitus with mild nonproliferative diabetic retinopathy without macular edema, bilateral | Diagnosis | ICD-10-CM |
| E13.3299 | Other specified diabetes mellitus with mild nonproliferative diabetic retinopathy without macular edema, unspecified eye | Diagnosis | ICD-10-CM |
| E13.3311 | Other specified diabetes mellitus with moderate nonproliferative diabetic retinopathy with macular edema, right eye | Diagnosis | ICD-10-CM |
| E13.3312 | Other specified diabetes mellitus with moderate nonproliferative diabetic retinopathy with macular edema, left eye | Diagnosis | ICD-10-CM |
| E13.3313 | Other specified diabetes mellitus with moderate nonproliferative diabetic retinopathy with macular edema, bilateral | Diagnosis | ICD-10-CM |
| E13.3319 | Other specified diabetes mellitus with moderate nonproliferative diabetic retinopathy with macular edema, unspecified eye | Diagnosis | ICD-10-CM |
| E13.3391 | Other specified diabetes mellitus with moderate nonproliferative diabetic retinopathy without macular edema, right eye | Diagnosis | ICD-10-CM |
| E13.3392 | Other specified diabetes mellitus with moderate nonproliferative diabetic retinopathy without macular edema, left eye | Diagnosis | ICD-10-CM |
| E13.3393 | Other specified diabetes mellitus with moderate nonproliferative diabetic retinopathy without macular edema, bilateral | Diagnosis | ICD-10-CM |
| E13.3399 | Other specified diabetes mellitus with moderate nonproliferative diabetic retinopathy without macular edema, unspecified eye | Diagnosis | ICD-10-CM |
| E13.3411 | Other specified diabetes mellitus with severe nonproliferative diabetic retinopathy with macular edema, right eye | Diagnosis | ICD-10-CM |
| E13.3412 | Other specified diabetes mellitus with severe nonproliferative diabetic retinopathy with macular edema, left eye | Diagnosis | ICD-10-CM |
| E13.3413 | Other specified diabetes mellitus with severe nonproliferative diabetic retinopathy with macular edema, bilateral | Diagnosis | ICD-10-CM |
| E13.3419 | Other specified diabetes mellitus with severe nonproliferative diabetic retinopathy with macular edema, unspecified eye | Diagnosis | ICD-10-CM |
| E13.3491 | Other specified diabetes mellitus with severe nonproliferative diabetic retinopathy without macular edema, right eye | Diagnosis | ICD-10-CM |
| E13.3492 | Other specified diabetes mellitus with severe nonproliferative diabetic retinopathy without macular edema, left eye | Diagnosis | ICD-10-CM |
| E13.3493 | Other specified diabetes mellitus with severe nonproliferative diabetic retinopathy without macular edema, bilateral | Diagnosis | ICD-10-CM |
| E13.3499 | Other specified diabetes mellitus with severe nonproliferative diabetic retinopathy without macular edema, unspecified eye | Diagnosis | ICD-10-CM |
| E13.3511 | Other specified diabetes mellitus with proliferative diabetic retinopathy with macular edema, right eye | Diagnosis | ICD-10-CM |
| E13.3512 | Other specified diabetes mellitus with proliferative diabetic retinopathy with macular edema, left eye | Diagnosis | ICD-10-CM |
| E13.3513 | Other specified diabetes mellitus with proliferative diabetic retinopathy with macular edema, bilateral | Diagnosis | ICD-10-CM |
| E13.3519 | Other specified diabetes mellitus with proliferative diabetic retinopathy with macular edema, unspecified eye | Diagnosis | ICD-10-CM |
| E13.3521 | Other specified diabetes mellitus with proliferative diabetic retinopathy with traction retinal detachment involving the macula, right eye | Diagnosis | ICD-10-CM |
| E13.3522 | Other specified diabetes mellitus with proliferative diabetic retinopathy with traction retinal detachment involving the macula, left eye | Diagnosis | ICD-10-CM |
| E13.3523 | Other specified diabetes mellitus with proliferative diabetic retinopathy with traction retinal detachment involving the macula, bilateral | Diagnosis | ICD-10-CM |
| E13.3529 | Other specified diabetes mellitus with proliferative diabetic retinopathy with traction retinal detachment involving the macula, unspecified eye | Diagnosis | ICD-10-CM |
| E13.3531 | Other specified diabetes mellitus with proliferative diabetic retinopathy with traction retinal detachment not involving the macula, right eye | Diagnosis | ICD-10-CM |
| E13.3532 | Other specified diabetes mellitus with proliferative diabetic retinopathy with traction retinal detachment not involving the macula, left eye | Diagnosis | ICD-10-CM |
| E13.3533 | Other specified diabetes mellitus with proliferative diabetic retinopathy with traction retinal detachment not involving the macula, bilateral | Diagnosis | ICD-10-CM |
| E13.3539 | Other specified diabetes mellitus with proliferative diabetic retinopathy with traction retinal detachment not involving the macula, unspecified eye | Diagnosis | ICD-10-CM |
| E13.3541 | Other specified diabetes mellitus with proliferative diabetic retinopathy with combined traction retinal detachment and rhegmatogenous retinal detachment, right eye | Diagnosis | ICD-10-CM |
| E13.3542 | Other specified diabetes mellitus with proliferative diabetic retinopathy with combined traction retinal detachment and rhegmatogenous retinal detachment, left eye | Diagnosis | ICD-10-CM |
| E13.3543 | Other specified diabetes mellitus with proliferative diabetic retinopathy with combined traction retinal detachment and rhegmatogenous retinal detachment, bilateral | Diagnosis | ICD-10-CM |
| E13.3549 | Other specified diabetes mellitus with proliferative diabetic retinopathy with combined traction retinal detachment and rhegmatogenous retinal detachment, unspecified eye | Diagnosis | ICD-10-CM |
| E13.3551 | Other specified diabetes mellitus with stable proliferative diabetic retinopathy, right eye | Diagnosis | ICD-10-CM |
| E13.3552 | Other specified diabetes mellitus with stable proliferative diabetic retinopathy, left eye | Diagnosis | ICD-10-CM |
| E13.3553 | Other specified diabetes mellitus with stable proliferative diabetic retinopathy, bilateral | Diagnosis | ICD-10-CM |
| E13.3559 | Other specified diabetes mellitus with stable proliferative diabetic retinopathy, unspecified eye | Diagnosis | ICD-10-CM |
| E13.3591 | Other specified diabetes mellitus with proliferative diabetic retinopathy without macular edema, right eye | Diagnosis | ICD-10-CM |
| E13.3592 | Other specified diabetes mellitus with proliferative diabetic retinopathy without macular edema, left eye | Diagnosis | ICD-10-CM |
| E13.3593 | Other specified diabetes mellitus with proliferative diabetic retinopathy without macular edema, bilateral | Diagnosis | ICD-10-CM |
| E13.3599 | Other specified diabetes mellitus with proliferative diabetic retinopathy without macular edema, unspecified eye | Diagnosis | ICD-10-CM |
| E13.36 | Other specified diabetes mellitus with diabetic cataract | Diagnosis | ICD-10-CM |
| E13.37X1 | Other specified diabetes mellitus with diabetic macular edema, resolved following treatment, right eye | Diagnosis | ICD-10-CM |
| E13.37X2 | Other specified diabetes mellitus with diabetic macular edema, resolved following treatment, left eye | Diagnosis | ICD-10-CM |
| E13.37X3 | Other specified diabetes mellitus with diabetic macular edema, resolved following treatment, bilateral | Diagnosis | ICD-10-CM |
| E13.37X9 | Other specified diabetes mellitus with diabetic macular edema, resolved following treatment, unspecified eye | Diagnosis | ICD-10-CM |
| E13.39 | Other specified diabetes mellitus with other diabetic ophthalmic complication | Diagnosis | ICD-10-CM |
| E13.40 | Other specified diabetes mellitus with diabetic neuropathy, unspecified | Diagnosis | ICD-10-CM |
| E13.41 | Other specified diabetes mellitus with diabetic mononeuropathy | Diagnosis | ICD-10-CM |
| E13.42 | Other specified diabetes mellitus with diabetic polyneuropathy | Diagnosis | ICD-10-CM |
| E13.43 | Other specified diabetes mellitus with diabetic autonomic (poly)neuropathy | Diagnosis | ICD-10-CM |
| E13.44 | Other specified diabetes mellitus with diabetic amyotrophy | Diagnosis | ICD-10-CM |
| E13.49 | Other specified diabetes mellitus with other diabetic neurological complication | Diagnosis | ICD-10-CM |
| E13.51 | Other specified diabetes mellitus with diabetic peripheral angiopathy without gangrene | Diagnosis | ICD-10-CM |
| E13.52 | Other specified diabetes mellitus with diabetic peripheral angiopathy with gangrene | Diagnosis | ICD-10-CM |
| E13.59 | Other specified diabetes mellitus with other circulatory complications | Diagnosis | ICD-10-CM |
| E13.610 | Other specified diabetes mellitus with diabetic neuropathic arthropathy | Diagnosis | ICD-10-CM |
| E13.618 | Other specified diabetes mellitus with other diabetic arthropathy | Diagnosis | ICD-10-CM |
| E13.620 | Other specified diabetes mellitus with diabetic dermatitis | Diagnosis | ICD-10-CM |
| E13.621 | Other specified diabetes mellitus with foot ulcer | Diagnosis | ICD-10-CM |
| E13.622 | Other specified diabetes mellitus with other skin ulcer | Diagnosis | ICD-10-CM |
| E13.628 | Other specified diabetes mellitus with other skin complications | Diagnosis | ICD-10-CM |
| E13.630 | Other specified diabetes mellitus with periodontal disease | Diagnosis | ICD-10-CM |
| E13.638 | Other specified diabetes mellitus with other oral complications | Diagnosis | ICD-10-CM |
| E13.641 | Other specified diabetes mellitus with hypoglycemia with coma | Diagnosis | ICD-10-CM |
| E13.649 | Other specified diabetes mellitus with hypoglycemia without coma | Diagnosis | ICD-10-CM |
| E13.65 | Other specified diabetes mellitus with hyperglycemia | Diagnosis | ICD-10-CM |
| E13.69 | Other specified diabetes mellitus with other specified complication | Diagnosis | ICD-10-CM |
| E13.8 | Other specified diabetes mellitus with unspecified complications | Diagnosis | ICD-10-CM |
| E13.9 | Other specified diabetes mellitus without complications | Diagnosis | ICD-10-CM |
| O24319 | Unspecified pre-existing diabetes mellitus in pregnancy, unspecified trimester | Diagnosis | ICD-10-CM |
| O2432 | Unspecified pre-existing diabetes mellitus in childbirth | Diagnosis | ICD-10-CM |
| O2492 | Unspecified diabetes mellitus in childbirth | Diagnosis | ICD-10-CM |
| O24913 | Unspecified diabetes mellitus in pregnancy, third trimester | Diagnosis | ICD-10-CM |
| O24912 | Unspecified diabetes mellitus in pregnancy, second trimester | Diagnosis | ICD-10-CM |
| O24911 | Unspecified diabetes mellitus in pregnancy, first trimester | Diagnosis | ICD-10-CM |
| O2493 | Unspecified diabetes mellitus in the puerperium | Diagnosis | ICD-10-CM |
| O24019 | Pre-existing type 1 diabetes mellitus, in pregnancy, unspecified trimester | Diagnosis | ICD-10-CM |
| O24919 | Unspecified diabetes mellitus in pregnancy, unspecified trimester | Diagnosis | ICD-10-CM |
| O24819 | Other pre-existing diabetes mellitus in pregnancy, unspecified trimester | Diagnosis | ICD-10-CM |
| O24119 | Pre-existing type 2 diabetes mellitus, in pregnancy, unspecified trimester | Diagnosis | ICD-10-CM |
| O24011 | Pre-existing type 1 diabetes mellitus, in pregnancy, first trimester | Diagnosis | ICD-10-CM |
| O2482 | Other pre-existing diabetes mellitus in childbirth | Diagnosis | ICD-10-CM |
| O24813 | Other pre-existing diabetes mellitus in pregnancy, third trimester | Diagnosis | ICD-10-CM |
| O24812 | Other pre-existing diabetes mellitus in pregnancy, second trimester | Diagnosis | ICD-10-CM |
| O24811 | Other pre-existing diabetes mellitus in pregnancy, first trimester | Diagnosis | ICD-10-CM |
| O24313 | Unspecified pre-existing diabetes mellitus in pregnancy, third trimester | Diagnosis | ICD-10-CM |
| O24312 | Unspecified pre-existing diabetes mellitus in pregnancy, second trimester | Diagnosis | ICD-10-CM |
| O24311 | Unspecified pre-existing diabetes mellitus in pregnancy, first trimester | Diagnosis | ICD-10-CM |
| O2412 | Pre-existing type 2 diabetes mellitus, in childbirth | Diagnosis | ICD-10-CM |
| O24113 | Pre-existing type 2 diabetes mellitus, in pregnancy, third trimester | Diagnosis | ICD-10-CM |
| O24112 | Pre-existing type 2 diabetes mellitus, in pregnancy, second trimester | Diagnosis | ICD-10-CM |
| O24111 | Pre-existing type 2 diabetes mellitus, in pregnancy, first trimester | Diagnosis | ICD-10-CM |
| O2402 | Pre-existing type 1 diabetes mellitus, in childbirth | Diagnosis | ICD-10-CM |
| O24013 | Pre-existing type 1 diabetes mellitus, in pregnancy, third trimester | Diagnosis | ICD-10-CM |
| O24012 | Pre-existing type 1 diabetes mellitus, in pregnancy, second trimester | Diagnosis | ICD-10-CM |
| O2403 | Pre-existing type 1 diabetes mellitus, in the puerperium | Diagnosis | ICD-10-CM |
| O2483 | Other pre-existing diabetes mellitus in the puerperium | Diagnosis | ICD-10-CM |
| O2433 | Unspecified pre-existing diabetes mellitus in the puerperium | Diagnosis | ICD-10-CM |
| O2413 | Pre-existing type 2 diabetes mellitus, in the puerperium | Diagnosis | ICD-10-CM |
| O24.414 | Gestational diabetes mellitus in pregnancy, insulin controlled | Diagnosis | ICD-10-CM |
| O24.415 | Gestational diabetes mellitus in pregnancy, controlled by oral hypoglycemic drugs | Diagnosis | ICD-10-CM |
| O24.419 | Gestational diabetes mellitus in pregnancy, unspecified control | Diagnosis | ICD-10-CM |
| O24.425 | Gestational diabetes mellitus in childbirth, controlled by oral hypoglycemic drugs | Diagnosis | ICD-10-CM |
| O24.429 | Gestational diabetes mellitus in childbirth, unspecified control | Diagnosis | ICD-10-CM |
| O24.434 | Gestational diabetes mellitus in the puerperium, insulin controlled | Diagnosis | ICD-10-CM |
| O24.435 | Gestational diabetes mellitus in puerperium, controlled by oral hypoglycemic drugs | Diagnosis | ICD-10-CM |
| O24.439 | Gestational diabetes mellitus in the puerperium, unspecified control | Diagnosis | ICD-10-CM |
| E08.610 | Diabetes mellitus due to underlying condition with diabetic neuropathic arthropathy | Diagnosis | ICD-10-CM |
| E08.618 | Diabetes mellitus due to underlying condition with other diabetic arthropathy | Diagnosis | ICD-10-CM |
| E09.610 | Drug or chemical induced diabetes mellitus with diabetic neuropathic arthropathy | Diagnosis | ICD-10-CM |
| E09.618 | Drug or chemical induced diabetes mellitus with other diabetic arthropathy | Diagnosis | ICD-10-CM |
| E10.610 | Type 1 diabetes mellitus with diabetic neuropathic arthropathy | Diagnosis | ICD-10-CM |
| E10.618 | Type 1 diabetes mellitus with other diabetic arthropathy | Diagnosis | ICD-10-CM |
| E11.610 | Type 2 diabetes mellitus with diabetic neuropathic arthropathy | Diagnosis | ICD-10-CM |
| E11.618 | Type 2 diabetes mellitus with other diabetic arthropathy | Diagnosis | ICD-10-CM |
| E13.610 | Other specified diabetes mellitus with diabetic neuropathic arthropathy | Diagnosis | ICD-10-CM |
| E13.618 | Other specified diabetes mellitus with other diabetic arthropathy | Diagnosis | ICD-10-CM |
| E08.21 | Diabetes mellitus due to underlying condition with diabetic nephropathy | Diagnosis | ICD-10-CM |
| E08.22 | Diabetes mellitus due to underlying condition with diabetic chronic kidney disease | Diagnosis | ICD-10-CM |
| E08.29 | Diabetes mellitus due to underlying condition with other diabetic kidney complication | Diagnosis | ICD-10-CM |
| E08.00 | Diabetes mellitus due to underlying condition with hyperosmolarity without nonketotic hyperglycemic-hyperosmolar coma (NKHHC) | Diagnosis | ICD-10-CM |
| E08.01 | Diabetes mellitus due to underlying condition with hyperosmolarity with coma | Diagnosis | ICD-10-CM |
| E08.10 | Diabetes mellitus due to underlying condition with ketoacidosis without coma | Diagnosis | ICD-10-CM |
| E08.11 | Diabetes mellitus due to underlying condition with ketoacidosis with coma | Diagnosis | ICD-10-CM |
| E08.311 | Diabetes mellitus due to underlying condition with unspecified diabetic retinopathy with macular edema | Diagnosis | ICD-10-CM |
| E08.319 | Diabetes mellitus due to underlying condition with unspecified diabetic retinopathy without macular edema | Diagnosis | ICD-10-CM |
| E08.3211 | Diabetes mellitus due to underlying condition with mild nonproliferative diabetic retinopathy with macular edema, right eye | Diagnosis | ICD-10-CM |
| E08.3212 | Diabetes mellitus due to underlying condition with mild nonproliferative diabetic retinopathy with macular edema, left eye | Diagnosis | ICD-10-CM |
| E08.3213 | Diabetes mellitus due to underlying condition with mild nonproliferative diabetic retinopathy with macular edema, bilateral | Diagnosis | ICD-10-CM |
| E08.3219 | Diabetes mellitus due to underlying condition with mild nonproliferative diabetic retinopathy with macular edema, unspecified eye | Diagnosis | ICD-10-CM |
| E08.3291 | Diabetes mellitus due to underlying condition with mild nonproliferative diabetic retinopathy without macular edema, right eye | Diagnosis | ICD-10-CM |
| E08.3292 | Diabetes mellitus due to underlying condition with mild nonproliferative diabetic retinopathy without macular edema, left eye | Diagnosis | ICD-10-CM |
| E08.3293 | Diabetes mellitus due to underlying condition with mild nonproliferative diabetic retinopathy without macular edema, bilateral | Diagnosis | ICD-10-CM |
| E08.3299 | Diabetes mellitus due to underlying condition with mild nonproliferative diabetic retinopathy without macular edema, unspecified eye | Diagnosis | ICD-10-CM |
| E08.3311 | Diabetes mellitus due to underlying condition with moderate nonproliferative diabetic retinopathy with macular edema, right eye | Diagnosis | ICD-10-CM |
| E08.3312 | Diabetes mellitus due to underlying condition with moderate nonproliferative diabetic retinopathy with macular edema, left eye | Diagnosis | ICD-10-CM |
| E08.3313 | Diabetes mellitus due to underlying condition with moderate nonproliferative diabetic retinopathy with macular edema, bilateral | Diagnosis | ICD-10-CM |
| E08.3319 | Diabetes mellitus due to underlying condition with moderate nonproliferative diabetic retinopathy with macular edema, unspecified eye | Diagnosis | ICD-10-CM |
| E08.3391 | Diabetes mellitus due to underlying condition with moderate nonproliferative diabetic retinopathy without macular edema, right eye | Diagnosis | ICD-10-CM |
| E08.3392 | Diabetes mellitus due to underlying condition with moderate nonproliferative diabetic retinopathy without macular edema, left eye | Diagnosis | ICD-10-CM |
| E08.3393 | Diabetes mellitus due to underlying condition with moderate nonproliferative diabetic retinopathy without macular edema, bilateral | Diagnosis | ICD-10-CM |
| E08.3399 | Diabetes mellitus due to underlying condition with moderate nonproliferative diabetic retinopathy without macular edema, unspecified eye | Diagnosis | ICD-10-CM |
| E08.3411 | Diabetes mellitus due to underlying condition with severe nonproliferative diabetic retinopathy with macular edema, right eye | Diagnosis | ICD-10-CM |
| E08.3412 | Diabetes mellitus due to underlying condition with severe nonproliferative diabetic retinopathy with macular edema, left eye | Diagnosis | ICD-10-CM |
| E08.3413 | Diabetes mellitus due to underlying condition with severe nonproliferative diabetic retinopathy with macular edema, bilateral | Diagnosis | ICD-10-CM |
| E08.3419 | Diabetes mellitus due to underlying condition with severe nonproliferative diabetic retinopathy with macular edema, unspecified eye | Diagnosis | ICD-10-CM |
| E08.3491 | Diabetes mellitus due to underlying condition with severe nonproliferative diabetic retinopathy without macular edema, right eye | Diagnosis | ICD-10-CM |
| E08.3492 | Diabetes mellitus due to underlying condition with severe nonproliferative diabetic retinopathy without macular edema, left eye | Diagnosis | ICD-10-CM |
| E08.3493 | Diabetes mellitus due to underlying condition with severe nonproliferative diabetic retinopathy without macular edema, bilateral | Diagnosis | ICD-10-CM |
| E08.3499 | Diabetes mellitus due to underlying condition with severe nonproliferative diabetic retinopathy without macular edema, unspecified eye | Diagnosis | ICD-10-CM |
| E08.3511 | Diabetes mellitus due to underlying condition with proliferative diabetic retinopathy with macular edema, right eye | Diagnosis | ICD-10-CM |
| E08.3512 | Diabetes mellitus due to underlying condition with proliferative diabetic retinopathy with macular edema, left eye | Diagnosis | ICD-10-CM |
| E08.3513 | Diabetes mellitus due to underlying condition with proliferative diabetic retinopathy with macular edema, bilateral | Diagnosis | ICD-10-CM |
| E08.3519 | Diabetes mellitus due to underlying condition with proliferative diabetic retinopathy with macular edema, unspecified eye | Diagnosis | ICD-10-CM |
| E08.3521 | Diabetes mellitus due to underlying condition with proliferative diabetic retinopathy with traction retinal detachment involving the macula, right eye | Diagnosis | ICD-10-CM |
| E08.3522 | Diabetes mellitus due to underlying condition with proliferative diabetic retinopathy with traction retinal detachment involving the macula, left eye | Diagnosis | ICD-10-CM |
| E08.3523 | Diabetes mellitus due to underlying condition with proliferative diabetic retinopathy with traction retinal detachment involving the macula, bilateral | Diagnosis | ICD-10-CM |
| E08.3529 | Diabetes mellitus due to underlying condition with proliferative diabetic retinopathy with traction retinal detachment involving the macula, unspecified eye | Diagnosis | ICD-10-CM |
| E08.3531 | Diabetes mellitus due to underlying condition with proliferative diabetic retinopathy with traction retinal detachment not involving the macula, right eye | Diagnosis | ICD-10-CM |
| E08.3532 | Diabetes mellitus due to underlying condition with proliferative diabetic retinopathy with traction retinal detachment not involving the macula, left eye | Diagnosis | ICD-10-CM |
| E08.3533 | Diabetes mellitus due to underlying condition with proliferative diabetic retinopathy with traction retinal detachment not involving the macula, bilateral | Diagnosis | ICD-10-CM |
| E08.3539 | Diabetes mellitus due to underlying condition with proliferative diabetic retinopathy with traction retinal detachment not involving the macula, unspecified eye | Diagnosis | ICD-10-CM |
| E08.3541 | Diabetes mellitus due to underlying condition with proliferative diabetic retinopathy with combined traction retinal detachment and rhegmatogenous retinal detachment, right eye | Diagnosis | ICD-10-CM |
| E08.3542 | Diabetes mellitus due to underlying condition with proliferative diabetic retinopathy with combined traction retinal detachment and rhegmatogenous retinal detachment, left eye | Diagnosis | ICD-10-CM |
| E08.3543 | Diabetes mellitus due to underlying condition with proliferative diabetic retinopathy with combined traction retinal detachment and rhegmatogenous retinal detachment, bilateral | Diagnosis | ICD-10-CM |
| E08.3549 | Diabetes mellitus due to underlying condition with proliferative diabetic retinopathy with combined traction retinal detachment and rhegmatogenous retinal detachment, unspecified eye | Diagnosis | ICD-10-CM |
| E08.3551 | Diabetes mellitus due to underlying condition with stable proliferative diabetic retinopathy, right eye | Diagnosis | ICD-10-CM |
| E08.3552 | Diabetes mellitus due to underlying condition with stable proliferative diabetic retinopathy, left eye | Diagnosis | ICD-10-CM |
| E08.3553 | Diabetes mellitus due to underlying condition with stable proliferative diabetic retinopathy, bilateral | Diagnosis | ICD-10-CM |
| E08.3559 | Diabetes mellitus due to underlying condition with stable proliferative diabetic retinopathy, unspecified eye | Diagnosis | ICD-10-CM |
| E08.3591 | Diabetes mellitus due to underlying condition with proliferative diabetic retinopathy without macular edema, right eye | Diagnosis | ICD-10-CM |
| E08.3592 | Diabetes mellitus due to underlying condition with proliferative diabetic retinopathy without macular edema, left eye | Diagnosis | ICD-10-CM |
| E08.3593 | Diabetes mellitus due to underlying condition with proliferative diabetic retinopathy without macular edema, bilateral | Diagnosis | ICD-10-CM |
| E08.3599 | Diabetes mellitus due to underlying condition with proliferative diabetic retinopathy without macular edema, unspecified eye | Diagnosis | ICD-10-CM |
| E08.36 | Diabetes mellitus due to underlying condition with diabetic cataract | Diagnosis | ICD-10-CM |
| E08.37X1 | Diabetes mellitus due to underlying condition with diabetic macular edema, resolved following treatment, right eye | Diagnosis | ICD-10-CM |
| E08.37X2 | Diabetes mellitus due to underlying condition with diabetic macular edema, resolved following treatment, left eye | Diagnosis | ICD-10-CM |
| E08.37X3 | Diabetes mellitus due to underlying condition with diabetic macular edema, resolved following treatment, bilateral | Diagnosis | ICD-10-CM |
| E08.37X9 | Diabetes mellitus due to underlying condition with diabetic macular edema, resolved following treatment, unspecified eye | Diagnosis | ICD-10-CM |
| E08.39 | Diabetes mellitus due to underlying condition with other diabetic ophthalmic complication | Diagnosis | ICD-10-CM |
| E08.40 | Diabetes mellitus due to underlying condition with diabetic neuropathy, unspecified | Diagnosis | ICD-10-CM |
| E08.41 | Diabetes mellitus due to underlying condition with diabetic mononeuropathy | Diagnosis | ICD-10-CM |
| E08.42 | Diabetes mellitus due to underlying condition with diabetic polyneuropathy | Diagnosis | ICD-10-CM |
| E08.43 | Diabetes mellitus due to underlying condition with diabetic autonomic (poly)neuropathy | Diagnosis | ICD-10-CM |
| E08.44 | Diabetes mellitus due to underlying condition with diabetic amyotrophy | Diagnosis | ICD-10-CM |
| E08.49 | Diabetes mellitus due to underlying condition with other diabetic neurological complication | Diagnosis | ICD-10-CM |
| E08.51 | Diabetes mellitus due to underlying condition with diabetic peripheral angiopathy without gangrene | Diagnosis | ICD-10-CM |
| E08.52 | Diabetes mellitus due to underlying condition with diabetic peripheral angiopathy with gangrene | Diagnosis | ICD-10-CM |
| E08.59 | Diabetes mellitus due to underlying condition with other circulatory complications | Diagnosis | ICD-10-CM |
| E08.610 | Diabetes mellitus due to underlying condition with diabetic neuropathic arthropathy | Diagnosis | ICD-10-CM |
| E08.618 | Diabetes mellitus due to underlying condition with other diabetic arthropathy | Diagnosis | ICD-10-CM |
| E08.620 | Diabetes mellitus due to underlying condition with diabetic dermatitis | Diagnosis | ICD-10-CM |
| E08.621 | Diabetes mellitus due to underlying condition with foot ulcer | Diagnosis | ICD-10-CM |
| E08.622 | Diabetes mellitus due to underlying condition with other skin ulcer | Diagnosis | ICD-10-CM |
| E08.628 | Diabetes mellitus due to underlying condition with other skin complications | Diagnosis | ICD-10-CM |
| E08.630 | Diabetes mellitus due to underlying condition with periodontal disease | Diagnosis | ICD-10-CM |
| E08.638 | Diabetes mellitus due to underlying condition with other oral complications | Diagnosis | ICD-10-CM |
| E08.641 | Diabetes mellitus due to underlying condition with hypoglycemia with coma | Diagnosis | ICD-10-CM |
| E08.649 | Diabetes mellitus due to underlying condition with hypoglycemia without coma | Diagnosis | ICD-10-CM |
| E08.65 | Diabetes mellitus due to underlying condition with hyperglycemia | Diagnosis | ICD-10-CM |
| E08.69 | Diabetes mellitus due to underlying condition with other specified complication | Diagnosis | ICD-10-CM |
| E08.8 | Diabetes mellitus due to underlying condition with unspecified complications | Diagnosis | ICD-10-CM |
| E08.9 | Diabetes mellitus due to underlying condition without complications | Diagnosis | ICD-10-CM |
| E09.00 | Drug or chemical induced diabetes mellitus with hyperosmolarity without nonketotic hyperglycemic-hyperosmolar coma (NKHHC) | Diagnosis | ICD-10-CM |
| E09.01 | Drug or chemical induced diabetes mellitus with hyperosmolarity with coma | Diagnosis | ICD-10-CM |
| E09.10 | Drug or chemical induced diabetes mellitus with ketoacidosis without coma | Diagnosis | ICD-10-CM |
| E09.11 | Drug or chemical induced diabetes mellitus with ketoacidosis with coma | Diagnosis | ICD-10-CM |
| E09.21 | Drug or chemical induced diabetes mellitus with diabetic nephropathy | Diagnosis | ICD-10-CM |
| E09.22 | Drug or chemical induced diabetes mellitus with diabetic chronic kidney disease | Diagnosis | ICD-10-CM |
| E09.29 | Drug or chemical induced diabetes mellitus with other diabetic kidney complication | Diagnosis | ICD-10-CM |
| E09.311 | Drug or chemical induced diabetes mellitus with unspecified diabetic retinopathy with macular edema | Diagnosis | ICD-10-CM |
| E09.319 | Drug or chemical induced diabetes mellitus with unspecified diabetic retinopathy without macular edema | Diagnosis | ICD-10-CM |
| E09.3211 | Drug or chemical induced diabetes mellitus with mild nonproliferative diabetic retinopathy with macular edema, right eye | Diagnosis | ICD-10-CM |
| E09.3212 | Drug or chemical induced diabetes mellitus with mild nonproliferative diabetic retinopathy with macular edema, left eye | Diagnosis | ICD-10-CM |
| E09.3213 | Drug or chemical induced diabetes mellitus with mild nonproliferative diabetic retinopathy with macular edema, bilateral | Diagnosis | ICD-10-CM |
| E09.3219 | Drug or chemical induced diabetes mellitus with mild nonproliferative diabetic retinopathy with macular edema, unspecified eye | Diagnosis | ICD-10-CM |
| E09.3291 | Drug or chemical induced diabetes mellitus with mild nonproliferative diabetic retinopathy without macular edema, right eye | Diagnosis | ICD-10-CM |
| E09.3292 | Drug or chemical induced diabetes mellitus with mild nonproliferative diabetic retinopathy without macular edema, left eye | Diagnosis | ICD-10-CM |
| E09.3293 | Drug or chemical induced diabetes mellitus with mild nonproliferative diabetic retinopathy without macular edema, bilateral | Diagnosis | ICD-10-CM |
| E09.3299 | Drug or chemical induced diabetes mellitus with mild nonproliferative diabetic retinopathy without macular edema, unspecified eye | Diagnosis | ICD-10-CM |
| E09.3311 | Drug or chemical induced diabetes mellitus with moderate nonproliferative diabetic retinopathy with macular edema, right eye | Diagnosis | ICD-10-CM |
| E09.3312 | Drug or chemical induced diabetes mellitus with moderate nonproliferative diabetic retinopathy with macular edema, left eye | Diagnosis | ICD-10-CM |
| E09.3313 | Drug or chemical induced diabetes mellitus with moderate nonproliferative diabetic retinopathy with macular edema, bilateral | Diagnosis | ICD-10-CM |
| E09.3319 | Drug or chemical induced diabetes mellitus with moderate nonproliferative diabetic retinopathy with macular edema, unspecified eye | Diagnosis | ICD-10-CM |
| E09.3391 | Drug or chemical induced diabetes mellitus with moderate nonproliferative diabetic retinopathy without macular edema, right eye | Diagnosis | ICD-10-CM |
| E09.3392 | Drug or chemical induced diabetes mellitus with moderate nonproliferative diabetic retinopathy without macular edema, left eye | Diagnosis | ICD-10-CM |
| E09.3393 | Drug or chemical induced diabetes mellitus with moderate nonproliferative diabetic retinopathy without macular edema, bilateral | Diagnosis | ICD-10-CM |
| E09.3399 | Drug or chemical induced diabetes mellitus with moderate nonproliferative diabetic retinopathy without macular edema, unspecified eye | Diagnosis | ICD-10-CM |
| E09.3411 | Drug or chemical induced diabetes mellitus with severe nonproliferative diabetic retinopathy with macular edema, right eye | Diagnosis | ICD-10-CM |
| E09.3412 | Drug or chemical induced diabetes mellitus with severe nonproliferative diabetic retinopathy with macular edema, left eye | Diagnosis | ICD-10-CM |
| E09.3413 | Drug or chemical induced diabetes mellitus with severe nonproliferative diabetic retinopathy with macular edema, bilateral | Diagnosis | ICD-10-CM |
| E09.3419 | Drug or chemical induced diabetes mellitus with severe nonproliferative diabetic retinopathy with macular edema, unspecified eye | Diagnosis | ICD-10-CM |
| E09.3491 | Drug or chemical induced diabetes mellitus with severe nonproliferative diabetic retinopathy without macular edema, right eye | Diagnosis | ICD-10-CM |
| E09.3492 | Drug or chemical induced diabetes mellitus with severe nonproliferative diabetic retinopathy without macular edema, left eye | Diagnosis | ICD-10-CM |
| E09.3493 | Drug or chemical induced diabetes mellitus with severe nonproliferative diabetic retinopathy without macular edema, bilateral | Diagnosis | ICD-10-CM |
| E09.3499 | Drug or chemical induced diabetes mellitus with severe nonproliferative diabetic retinopathy without macular edema, unspecified eye | Diagnosis | ICD-10-CM |
| E09.3511 | Drug or chemical induced diabetes mellitus with proliferative diabetic retinopathy with macular edema, right eye | Diagnosis | ICD-10-CM |
| E09.3512 | Drug or chemical induced diabetes mellitus with proliferative diabetic retinopathy with macular edema, left eye | Diagnosis | ICD-10-CM |
| E09.3513 | Drug or chemical induced diabetes mellitus with proliferative diabetic retinopathy with macular edema, bilateral | Diagnosis | ICD-10-CM |
| E09.3519 | Drug or chemical induced diabetes mellitus with proliferative diabetic retinopathy with macular edema, unspecified eye | Diagnosis | ICD-10-CM |
| E09.3521 | Drug or chemical induced diabetes mellitus with proliferative diabetic retinopathy with traction retinal detachment involving the macula, right eye | Diagnosis | ICD-10-CM |
| E09.3522 | Drug or chemical induced diabetes mellitus with proliferative diabetic retinopathy with traction retinal detachment involving the macula, left eye | Diagnosis | ICD-10-CM |
| E09.3523 | Drug or chemical induced diabetes mellitus with proliferative diabetic retinopathy with traction retinal detachment involving the macula, bilateral | Diagnosis | ICD-10-CM |
| E09.3529 | Drug or chemical induced diabetes mellitus with proliferative diabetic retinopathy with traction retinal detachment involving the macula, unspecified eye | Diagnosis | ICD-10-CM |
| E09.3531 | Drug or chemical induced diabetes mellitus with proliferative diabetic retinopathy with traction retinal detachment not involving the macula, right eye | Diagnosis | ICD-10-CM |
| E09.3532 | Drug or chemical induced diabetes mellitus with proliferative diabetic retinopathy with traction retinal detachment not involving the macula, left eye | Diagnosis | ICD-10-CM |
| E09.3533 | Drug or chemical induced diabetes mellitus with proliferative diabetic retinopathy with traction retinal detachment not involving the macula, bilateral | Diagnosis | ICD-10-CM |
| E09.3539 | Drug or chemical induced diabetes mellitus with proliferative diabetic retinopathy with traction retinal detachment not involving the macula, unspecified eye | Diagnosis | ICD-10-CM |
| E09.3541 | Drug or chemical induced diabetes mellitus with proliferative diabetic retinopathy with combined traction retinal detachment and rhegmatogenous retinal detachment, right eye | Diagnosis | ICD-10-CM |
| E09.3542 | Drug or chemical induced diabetes mellitus with proliferative diabetic retinopathy with combined traction retinal detachment and rhegmatogenous retinal detachment, left eye | Diagnosis | ICD-10-CM |
| E09.3543 | Drug or chemical induced diabetes mellitus with proliferative diabetic retinopathy with combined traction retinal detachment and rhegmatogenous retinal detachment, bilateral | Diagnosis | ICD-10-CM |
| E09.3549 | Drug or chemical induced diabetes mellitus with proliferative diabetic retinopathy with combined traction retinal detachment and rhegmatogenous retinal detachment, unspecified eye | Diagnosis | ICD-10-CM |
| E09.3551 | Drug or chemical induced diabetes mellitus with stable proliferative diabetic retinopathy, right eye | Diagnosis | ICD-10-CM |
| E09.3552 | Drug or chemical induced diabetes mellitus with stable proliferative diabetic retinopathy, left eye | Diagnosis | ICD-10-CM |
| E09.3553 | Drug or chemical induced diabetes mellitus with stable proliferative diabetic retinopathy, bilateral | Diagnosis | ICD-10-CM |
| E09.3559 | Drug or chemical induced diabetes mellitus with stable proliferative diabetic retinopathy, unspecified eye | Diagnosis | ICD-10-CM |
| E09.3591 | Drug or chemical induced diabetes mellitus with proliferative diabetic retinopathy without macular edema, right eye | Diagnosis | ICD-10-CM |
| E09.3592 | Drug or chemical induced diabetes mellitus with proliferative diabetic retinopathy without macular edema, left eye | Diagnosis | ICD-10-CM |
| E09.3593 | Drug or chemical induced diabetes mellitus with proliferative diabetic retinopathy without macular edema, bilateral | Diagnosis | ICD-10-CM |
| E09.3599 | Drug or chemical induced diabetes mellitus with proliferative diabetic retinopathy without macular edema, unspecified eye | Diagnosis | ICD-10-CM |
| E09.36 | Drug or chemical induced diabetes mellitus with diabetic cataract | Diagnosis | ICD-10-CM |
| E09.37X1 | Drug or chemical induced diabetes mellitus with diabetic macular edema, resolved following treatment, right eye | Diagnosis | ICD-10-CM |
| E09.37X2 | Drug or chemical induced diabetes mellitus with diabetic macular edema, resolved following treatment, left eye | Diagnosis | ICD-10-CM |
| E09.37X3 | Drug or chemical induced diabetes mellitus with diabetic macular edema, resolved following treatment, bilateral | Diagnosis | ICD-10-CM |
| E09.37X9 | Drug or chemical induced diabetes mellitus with diabetic macular edema, resolved following treatment, unspecified eye | Diagnosis | ICD-10-CM |
| E09.39 | Drug or chemical induced diabetes mellitus with other diabetic ophthalmic complication | Diagnosis | ICD-10-CM |
| E09.40 | Drug or chemical induced diabetes mellitus with neurological complications with diabetic neuropathy, unspecified | Diagnosis | ICD-10-CM |
| E09.41 | Drug or chemical induced diabetes mellitus with neurological complications with diabetic mononeuropathy | Diagnosis | ICD-10-CM |
| E09.42 | Drug or chemical induced diabetes mellitus with neurological complications with diabetic polyneuropathy | Diagnosis | ICD-10-CM |
| E09.43 | Drug or chemical induced diabetes mellitus with neurological complications with diabetic autonomic (poly)neuropathy | Diagnosis | ICD-10-CM |
| E09.44 | Drug or chemical induced diabetes mellitus with neurological complications with diabetic amyotrophy | Diagnosis | ICD-10-CM |
| E09.49 | Drug or chemical induced diabetes mellitus with neurological complications with other diabetic neurological complication | Diagnosis | ICD-10-CM |
| E09.51 | Drug or chemical induced diabetes mellitus with diabetic peripheral angiopathy without gangrene | Diagnosis | ICD-10-CM |
| E09.52 | Drug or chemical induced diabetes mellitus with diabetic peripheral angiopathy with gangrene | Diagnosis | ICD-10-CM |
| E09.59 | Drug or chemical induced diabetes mellitus with other circulatory complications | Diagnosis | ICD-10-CM |
| E09.610 | Drug or chemical induced diabetes mellitus with diabetic neuropathic arthropathy | Diagnosis | ICD-10-CM |
| E09.618 | Drug or chemical induced diabetes mellitus with other diabetic arthropathy | Diagnosis | ICD-10-CM |
| E09.620 | Drug or chemical induced diabetes mellitus with diabetic dermatitis | Diagnosis | ICD-10-CM |
| E09.621 | Drug or chemical induced diabetes mellitus with foot ulcer | Diagnosis | ICD-10-CM |
| E09.622 | Drug or chemical induced diabetes mellitus with other skin ulcer | Diagnosis | ICD-10-CM |
| E09.628 | Drug or chemical induced diabetes mellitus with other skin complications | Diagnosis | ICD-10-CM |
| E09.630 | Drug or chemical induced diabetes mellitus with periodontal disease | Diagnosis | ICD-10-CM |
| E09.638 | Drug or chemical induced diabetes mellitus with other oral complications | Diagnosis | ICD-10-CM |
| E09.641 | Drug or chemical induced diabetes mellitus with hypoglycemia with coma | Diagnosis | ICD-10-CM |
| E09.649 | Drug or chemical induced diabetes mellitus with hypoglycemia without coma | Diagnosis | ICD-10-CM |
| E09.65 | Drug or chemical induced diabetes mellitus with hyperglycemia | Diagnosis | ICD-10-CM |
| E09.69 | Drug or chemical induced diabetes mellitus with other specified complication | Diagnosis | ICD-10-CM |
| E09.8 | Drug or chemical induced diabetes mellitus with unspecified complications | Diagnosis | ICD-10-CM |
| E09.9 | Drug or chemical induced diabetes mellitus without complications | Diagnosis | ICD-10-CM |
| E13.00 | Other specified diabetes mellitus with hyperosmolarity without nonketotic hyperglycemic-hyperosmolar coma (NKHHC) | Diagnosis | ICD-10-CM |
| E13.01 | Other specified diabetes mellitus with hyperosmolarity with coma | Diagnosis | ICD-10-CM |
| E13.10 | Other specified diabetes mellitus with ketoacidosis without coma | Diagnosis | ICD-10-CM |
| E13.11 | Other specified diabetes mellitus with ketoacidosis with coma | Diagnosis | ICD-10-CM |
| E13.311 | Other specified diabetes mellitus with unspecified diabetic retinopathy with macular edema | Diagnosis | ICD-10-CM |
| E13.319 | Other specified diabetes mellitus with unspecified diabetic retinopathy without macular edema | Diagnosis | ICD-10-CM |
| E13.3211 | Other specified diabetes mellitus with mild nonproliferative diabetic retinopathy with macular edema, right eye | Diagnosis | ICD-10-CM |
| E13.3212 | Other specified diabetes mellitus with mild nonproliferative diabetic retinopathy with macular edema, left eye | Diagnosis | ICD-10-CM |
| E13.3213 | Other specified diabetes mellitus with mild nonproliferative diabetic retinopathy with macular edema, bilateral | Diagnosis | ICD-10-CM |
| E13.3219 | Other specified diabetes mellitus with mild nonproliferative diabetic retinopathy with macular edema, unspecified eye | Diagnosis | ICD-10-CM |
| E13.3291 | Other specified diabetes mellitus with mild nonproliferative diabetic retinopathy without macular edema, right eye | Diagnosis | ICD-10-CM |
| E13.3292 | Other specified diabetes mellitus with mild nonproliferative diabetic retinopathy without macular edema, left eye | Diagnosis | ICD-10-CM |
| E13.3293 | Other specified diabetes mellitus with mild nonproliferative diabetic retinopathy without macular edema, bilateral | Diagnosis | ICD-10-CM |
| E13.3299 | Other specified diabetes mellitus with mild nonproliferative diabetic retinopathy without macular edema, unspecified eye | Diagnosis | ICD-10-CM |
| E13.3311 | Other specified diabetes mellitus with moderate nonproliferative diabetic retinopathy with macular edema, right eye | Diagnosis | ICD-10-CM |
| E13.3312 | Other specified diabetes mellitus with moderate nonproliferative diabetic retinopathy with macular edema, left eye | Diagnosis | ICD-10-CM |
| E13.3313 | Other specified diabetes mellitus with moderate nonproliferative diabetic retinopathy with macular edema, bilateral | Diagnosis | ICD-10-CM |
| E13.3319 | Other specified diabetes mellitus with moderate nonproliferative diabetic retinopathy with macular edema, unspecified eye | Diagnosis | ICD-10-CM |
| E13.3391 | Other specified diabetes mellitus with moderate nonproliferative diabetic retinopathy without macular edema, right eye | Diagnosis | ICD-10-CM |
| E13.3392 | Other specified diabetes mellitus with moderate nonproliferative diabetic retinopathy without macular edema, left eye | Diagnosis | ICD-10-CM |
| E13.3393 | Other specified diabetes mellitus with moderate nonproliferative diabetic retinopathy without macular edema, bilateral | Diagnosis | ICD-10-CM |
| E13.3399 | Other specified diabetes mellitus with moderate nonproliferative diabetic retinopathy without macular edema, unspecified eye | Diagnosis | ICD-10-CM |
| E13.3411 | Other specified diabetes mellitus with severe nonproliferative diabetic retinopathy with macular edema, right eye | Diagnosis | ICD-10-CM |
| E13.3412 | Other specified diabetes mellitus with severe nonproliferative diabetic retinopathy with macular edema, left eye | Diagnosis | ICD-10-CM |
| E13.3413 | Other specified diabetes mellitus with severe nonproliferative diabetic retinopathy with macular edema, bilateral | Diagnosis | ICD-10-CM |
| E13.3419 | Other specified diabetes mellitus with severe nonproliferative diabetic retinopathy with macular edema, unspecified eye | Diagnosis | ICD-10-CM |
| E13.3491 | Other specified diabetes mellitus with severe nonproliferative diabetic retinopathy without macular edema, right eye | Diagnosis | ICD-10-CM |
| E13.3492 | Other specified diabetes mellitus with severe nonproliferative diabetic retinopathy without macular edema, left eye | Diagnosis | ICD-10-CM |
| E13.3493 | Other specified diabetes mellitus with severe nonproliferative diabetic retinopathy without macular edema, bilateral | Diagnosis | ICD-10-CM |
| E13.3499 | Other specified diabetes mellitus with severe nonproliferative diabetic retinopathy without macular edema, unspecified eye | Diagnosis | ICD-10-CM |
| E13.3511 | Other specified diabetes mellitus with proliferative diabetic retinopathy with macular edema, right eye | Diagnosis | ICD-10-CM |
| E13.3512 | Other specified diabetes mellitus with proliferative diabetic retinopathy with macular edema, left eye | Diagnosis | ICD-10-CM |
| E13.3513 | Other specified diabetes mellitus with proliferative diabetic retinopathy with macular edema, bilateral | Diagnosis | ICD-10-CM |
| E13.3519 | Other specified diabetes mellitus with proliferative diabetic retinopathy with macular edema, unspecified eye | Diagnosis | ICD-10-CM |
| E13.3521 | Other specified diabetes mellitus with proliferative diabetic retinopathy with traction retinal detachment involving the macula, right eye | Diagnosis | ICD-10-CM |
| E13.3522 | Other specified diabetes mellitus with proliferative diabetic retinopathy with traction retinal detachment involving the macula, left eye | Diagnosis | ICD-10-CM |
| E13.3523 | Other specified diabetes mellitus with proliferative diabetic retinopathy with traction retinal detachment involving the macula, bilateral | Diagnosis | ICD-10-CM |
| E13.3529 | Other specified diabetes mellitus with proliferative diabetic retinopathy with traction retinal detachment involving the macula, unspecified eye | Diagnosis | ICD-10-CM |
| E13.3531 | Other specified diabetes mellitus with proliferative diabetic retinopathy with traction retinal detachment not involving the macula, right eye | Diagnosis | ICD-10-CM |
| E13.3532 | Other specified diabetes mellitus with proliferative diabetic retinopathy with traction retinal detachment not involving the macula, left eye | Diagnosis | ICD-10-CM |
| E13.3533 | Other specified diabetes mellitus with proliferative diabetic retinopathy with traction retinal detachment not involving the macula, bilateral | Diagnosis | ICD-10-CM |
| E13.3539 | Other specified diabetes mellitus with proliferative diabetic retinopathy with traction retinal detachment not involving the macula, unspecified eye | Diagnosis | ICD-10-CM |
| E13.3541 | Other specified diabetes mellitus with proliferative diabetic retinopathy with combined traction retinal detachment and rhegmatogenous retinal detachment, right eye | Diagnosis | ICD-10-CM |
| E13.3542 | Other specified diabetes mellitus with proliferative diabetic retinopathy with combined traction retinal detachment and rhegmatogenous retinal detachment, left eye | Diagnosis | ICD-10-CM |
| E13.3543 | Other specified diabetes mellitus with proliferative diabetic retinopathy with combined traction retinal detachment and rhegmatogenous retinal detachment, bilateral | Diagnosis | ICD-10-CM |
| E13.3549 | Other specified diabetes mellitus with proliferative diabetic retinopathy with combined traction retinal detachment and rhegmatogenous retinal detachment, unspecified eye | Diagnosis | ICD-10-CM |
| E13.3551 | Other specified diabetes mellitus with stable proliferative diabetic retinopathy, right eye | Diagnosis | ICD-10-CM |
| E13.3552 | Other specified diabetes mellitus with stable proliferative diabetic retinopathy, left eye | Diagnosis | ICD-10-CM |
| E13.3553 | Other specified diabetes mellitus with stable proliferative diabetic retinopathy, bilateral | Diagnosis | ICD-10-CM |
| E13.3559 | Other specified diabetes mellitus with stable proliferative diabetic retinopathy, unspecified eye | Diagnosis | ICD-10-CM |
| E13.3591 | Other specified diabetes mellitus with proliferative diabetic retinopathy without macular edema, right eye | Diagnosis | ICD-10-CM |
| E13.3592 | Other specified diabetes mellitus with proliferative diabetic retinopathy without macular edema, left eye | Diagnosis | ICD-10-CM |
| E13.3593 | Other specified diabetes mellitus with proliferative diabetic retinopathy without macular edema, bilateral | Diagnosis | ICD-10-CM |
| E13.3599 | Other specified diabetes mellitus with proliferative diabetic retinopathy without macular edema, unspecified eye | Diagnosis | ICD-10-CM |
| E13.36 | Other specified diabetes mellitus with diabetic cataract | Diagnosis | ICD-10-CM |
| E13.37X1 | Other specified diabetes mellitus with diabetic macular edema, resolved following treatment, right eye | Diagnosis | ICD-10-CM |
| E13.37X2 | Other specified diabetes mellitus with diabetic macular edema, resolved following treatment, left eye | Diagnosis | ICD-10-CM |
| E13.37X3 | Other specified diabetes mellitus with diabetic macular edema, resolved following treatment, bilateral | Diagnosis | ICD-10-CM |
| E13.37X9 | Other specified diabetes mellitus with diabetic macular edema, resolved following treatment, unspecified eye | Diagnosis | ICD-10-CM |
| E13.39 | Other specified diabetes mellitus with other diabetic ophthalmic complication | Diagnosis | ICD-10-CM |
| E13.618 | Other specified diabetes mellitus with other diabetic arthropathy | Diagnosis | ICD-10-CM |
| E13.620 | Other specified diabetes mellitus with diabetic dermatitis | Diagnosis | ICD-10-CM |
| E13.621 | Other specified diabetes mellitus with foot ulcer | Diagnosis | ICD-10-CM |
| E13.622 | Other specified diabetes mellitus with other skin ulcer | Diagnosis | ICD-10-CM |
| E13.628 | Other specified diabetes mellitus with other skin complications | Diagnosis | ICD-10-CM |
| E13.630 | Other specified diabetes mellitus with periodontal disease | Diagnosis | ICD-10-CM |
| E13.638 | Other specified diabetes mellitus with other oral complications | Diagnosis | ICD-10-CM |
| E13.641 | Other specified diabetes mellitus with hypoglycemia with coma | Diagnosis | ICD-10-CM |
| E13.649 | Other specified diabetes mellitus with hypoglycemia without coma | Diagnosis | ICD-10-CM |
| E13.65 | Other specified diabetes mellitus with hyperglycemia | Diagnosis | ICD-10-CM |
| E13.69 | Other specified diabetes mellitus with other specified complication | Diagnosis | ICD-10-CM |
| E13.9 | Other specified diabetes mellitus without complications | Diagnosis | ICD-10-CM |
| **Obesity** | | | |
| E66.01 | Morbid (severe) obesity due to excess calories | Diagnosis | ICD-10-CM |
| E66.09 | Other obesity due to excess calories | Diagnosis | ICD-10-CM |
| E66.1 | Drug-induced obesity | Diagnosis | ICD-10-CM |
| E66.8 | Other obesity | Diagnosis | ICD-10-CM |
| E66.9 | Obesity, unspecified | Diagnosis | ICD-10-CM |
| K95.01 | Infection due to gastric band procedure | Diagnosis | ICD-10-CM |
| K95.09 | Other complications of gastric band procedure | Diagnosis | ICD-10-CM |
| K95.81 | Infection due to other bariatric procedure | Diagnosis | ICD-10-CM |
| K95.89 | Other complications of other bariatric procedure | Diagnosis | ICD-10-CM |
| O99.210 | Obesity complicating pregnancy, unspecified trimester | Diagnosis | ICD-10-CM |
| O99.211 | Obesity complicating pregnancy, first trimester | Diagnosis | ICD-10-CM |
| O99.212 | Obesity complicating pregnancy, second trimester | Diagnosis | ICD-10-CM |
| O99.213 | Obesity complicating pregnancy, third trimester | Diagnosis | ICD-10-CM |
| O99.214 | Obesity complicating childbirth | Diagnosis | ICD-10-CM |
| O99.215 | Obesity complicating the puerperium | Diagnosis | ICD-10-CM |
| O99.840 | Bariatric surgery status complicating pregnancy, unspecified trimester | Diagnosis | ICD-10-CM |
| O99.841 | Bariatric surgery status complicating pregnancy, first trimester | Diagnosis | ICD-10-CM |
| O99.842 | Bariatric surgery status complicating pregnancy, second trimester | Diagnosis | ICD-10-CM |
| O99.843 | Bariatric surgery status complicating pregnancy, third trimester | Diagnosis | ICD-10-CM |
| O99.844 | Bariatric surgery status complicating childbirth | Diagnosis | ICD-10-CM |
| O99.845 | Bariatric surgery status complicating the puerperium | Diagnosis | ICD-10-CM |
| Z68.30 | Body mass index (BMI) 30.0-30.9, adult | Diagnosis | ICD-10-CM |
| Z68.31 | Body mass index (BMI) 31.0-31.9, adult | Diagnosis | ICD-10-CM |
| Z68.32 | Body mass index (BMI) 32.0-32.9, adult | Diagnosis | ICD-10-CM |
| Z68.33 | Body mass index (BMI) 33.0-33.9, adult | Diagnosis | ICD-10-CM |
| Z68.34 | Body mass index (BMI) 34.0-34.9, adult | Diagnosis | ICD-10-CM |
| Z68.35 | Body mass index (BMI) 35.0-35.9, adult | Diagnosis | ICD-10-CM |
| Z68.36 | Body mass index (BMI) 36.0-36.9, adult | Diagnosis | ICD-10-CM |
| Z68.37 | Body mass index (BMI) 37.0-37.9, adult | Diagnosis | ICD-10-CM |
| Z68.38 | Body mass index (BMI) 38.0-38.9, adult | Diagnosis | ICD-10-CM |
| Z68.39 | Body mass index (BMI) 39.0-39.9, adult | Diagnosis | ICD-10-CM |
| Z68.41 | Body mass index (BMI) 40.0-44.9, adult | Diagnosis | ICD-10-CM |
| Z68.42 | Body mass index (BMI) 45.0-49.9, adult | Diagnosis | ICD-10-CM |
| Z68.43 | Body mass index (BMI) 50-59.9, adult | Diagnosis | ICD-10-CM |
| Z68.44 | Body mass index (BMI) 60.0-69.9, adult | Diagnosis | ICD-10-CM |
| Z68.45 | Body mass index (BMI) 70 or greater, adult | Diagnosis | ICD-10-CM |
| E66.2 | Morbid (severe) obesity with alveolar hypoventilation | Diagnosis | ICD-10-CM |
| 43644 | Laparoscopy, surgical, gastric restrictive procedure; with gastric bypass and Roux-en-Y gastroenterostomy (roux limb 150 cm or less) | Procedure | CPT-4 |
| 43645 | Laparoscopy, surgical, gastric restrictive procedure; with gastric bypass and small intestine reconstruction to limit absorption | Procedure | CPT-4 |
| 43770 | Laparoscopy, surgical, gastric restrictive procedure; placement of adjustable gastric restrictive device (eg, gastric band and subcutaneous port components) | Procedure | CPT-4 |
| 43842 | Gastric restrictive procedure, without gastric bypass, for morbid obesity; vertical-banded gastroplasty | Procedure | CPT-4 |
| 43843 | Gastric restrictive procedure, without gastric bypass, for morbid obesity; other than vertical-banded gastroplasty | Procedure | CPT-4 |
| 43845 | Gastric restrictive procedure with partial gastrectomy, pylorus-preserving duodenoileostomy and ileoileostomy (50 to 100 cm common channel) to limit absorption (biliopancreatic diversion with duodenal switch) | Procedure | CPT-4 |
| 43846 | Gastric restrictive procedure, with gastric bypass for morbid obesity; with short limb (150 cm or less) Roux-en-Y gastroenterostomy | Procedure | CPT-4 |
| 43847 | Gastric restrictive procedure, with gastric bypass for morbid obesity; with small intestine reconstruction to limit absorption | Procedure | CPT-4 |
| 0D16079 | Bypass Stomach to Duodenum with Autologous Tissue Substitute, Open Approach | Procedure | ICD-10-PCS |
| 0D1607A | Bypass Stomach to Jejunum with Autologous Tissue Substitute, Open Approach | Procedure | ICD-10-PCS |
| 0D1607B | Bypass Stomach to Ileum with Autologous Tissue Substitute, Open Approach | Procedure | ICD-10-PCS |
| 0D1607L | Bypass Stomach to Transverse Colon with Autologous Tissue Substitute, Open Approach | Procedure | ICD-10-PCS |
| 0D160J9 | Bypass Stomach to Duodenum with Synthetic Substitute, Open Approach | Procedure | ICD-10-PCS |
| 0D160JA | Bypass Stomach to Jejunum with Synthetic Substitute, Open Approach | Procedure | ICD-10-PCS |
| 0D160JB | Bypass Stomach to Ileum with Synthetic Substitute, Open Approach | Procedure | ICD-10-PCS |
| 0D160JL | Bypass Stomach to Transverse Colon with Synthetic Substitute, Open Approach | Procedure | ICD-10-PCS |
| 0D160K9 | Bypass Stomach to Duodenum with Nonautologous Tissue Substitute, Open Approach | Procedure | ICD-10-PCS |
| 0D160KA | Bypass Stomach to Jejunum with Nonautologous Tissue Substitute, Open Approach | Procedure | ICD-10-PCS |
| 0D160KB | Bypass Stomach to Ileum with Nonautologous Tissue Substitute, Open Approach | Procedure | ICD-10-PCS |
| 0D160KL | Bypass Stomach to Transverse Colon with Nonautologous Tissue Substitute, Open Approach | Procedure | ICD-10-PCS |
| 0D160Z9 | Bypass Stomach to Duodenum, Open Approach | Procedure | ICD-10-PCS |
| 0D160ZA | Bypass Stomach to Jejunum, Open Approach | Procedure | ICD-10-PCS |
| 0D160ZB | Bypass Stomach to Ileum, Open Approach | Procedure | ICD-10-PCS |
| 0D160ZL | Bypass Stomach to Transverse Colon, Open Approach | Procedure | ICD-10-PCS |
| 0D16479 | Bypass Stomach to Duodenum with Autologous Tissue Substitute, Percutaneous Endoscopic Approach | Procedure | ICD-10-PCS |
| 0D1647A | Bypass Stomach to Jejunum with Autologous Tissue Substitute, Percutaneous Endoscopic Approach | Procedure | ICD-10-PCS |
| 0D1647B | Bypass Stomach to Ileum with Autologous Tissue Substitute, Percutaneous Endoscopic Approach | Procedure | ICD-10-PCS |
| 0D1647L | Bypass Stomach to Transverse Colon with Autologous Tissue Substitute, Percutaneous Endoscopic Approach | Procedure | ICD-10-PCS |
| 0D164J9 | Bypass Stomach to Duodenum with Synthetic Substitute, Percutaneous Endoscopic Approach | Procedure | ICD-10-PCS |
| 0D164JA | Bypass Stomach to Jejunum with Synthetic Substitute, Percutaneous Endoscopic Approach | Procedure | ICD-10-PCS |
| 0D164JB | Bypass Stomach to Ileum with Synthetic Substitute, Percutaneous Endoscopic Approach | Procedure | ICD-10-PCS |
| 0D164JL | Bypass Stomach to Transverse Colon with Synthetic Substitute, Percutaneous Endoscopic Approach | Procedure | ICD-10-PCS |
| 0D164K9 | Bypass Stomach to Duodenum with Nonautologous Tissue Substitute, Percutaneous Endoscopic Approach | Procedure | ICD-10-PCS |
| 0D164KA | Bypass Stomach to Jejunum with Nonautologous Tissue Substitute, Percutaneous Endoscopic Approach | Procedure | ICD-10-PCS |
| 0D164KB | Bypass Stomach to Ileum with Nonautologous Tissue Substitute, Percutaneous Endoscopic Approach | Procedure | ICD-10-PCS |
| 0D164KL | Bypass Stomach to Transverse Colon with Nonautologous Tissue Substitute, Percutaneous Endoscopic Approach | Procedure | ICD-10-PCS |
| 0D164Z9 | Bypass Stomach to Duodenum, Percutaneous Endoscopic Approach | Procedure | ICD-10-PCS |
| 0D164ZA | Bypass Stomach to Jejunum, Percutaneous Endoscopic Approach | Procedure | ICD-10-PCS |
| 0D164ZB | Bypass Stomach to Ileum, Percutaneous Endoscopic Approach | Procedure | ICD-10-PCS |
| 0D164ZL | Bypass Stomach to Transverse Colon, Percutaneous Endoscopic Approach | Procedure | ICD-10-PCS |
| 0D16879 | Bypass Stomach to Duodenum with Autologous Tissue Substitute, Via Natural or Artificial Opening Endoscopic | Procedure | ICD-10-PCS |
| 0D1687A | Bypass Stomach to Jejunum with Autologous Tissue Substitute, Via Natural or Artificial Opening Endoscopic | Procedure | ICD-10-PCS |
| 0D1687B | Bypass Stomach to Ileum with Autologous Tissue Substitute, Via Natural or Artificial Opening Endoscopic | Procedure | ICD-10-PCS |
| 0D1687L | Bypass Stomach to Transverse Colon with Autologous Tissue Substitute, Via Natural or Artificial Opening Endoscopic | Procedure | ICD-10-PCS |
| 0D168J9 | Bypass Stomach to Duodenum with Synthetic Substitute, Via Natural or Artificial Opening Endoscopic | Procedure | ICD-10-PCS |
| 0D168JA | Bypass Stomach to Jejunum with Synthetic Substitute, Via Natural or Artificial Opening Endoscopic | Procedure | ICD-10-PCS |
| 0D168JB | Bypass Stomach to Ileum with Synthetic Substitute, Via Natural or Artificial Opening Endoscopic | Procedure | ICD-10-PCS |
| 0D168JL | Bypass Stomach to Transverse Colon with Synthetic Substitute, Via Natural or Artificial Opening Endoscopic | Procedure | ICD-10-PCS |
| 0D168K9 | Bypass Stomach to Duodenum with Nonautologous Tissue Substitute, Via Natural or Artificial Opening Endoscopic | Procedure | ICD-10-PCS |
| 0D168KA | Bypass Stomach to Jejunum with Nonautologous Tissue Substitute, Via Natural or Artificial Opening Endoscopic | Procedure | ICD-10-PCS |
| 0D168KB | Bypass Stomach to Ileum with Nonautologous Tissue Substitute, Via Natural or Artificial Opening Endoscopic | Procedure | ICD-10-PCS |
| 0D168KL | Bypass Stomach to Transverse Colon with Nonautologous Tissue Substitute, Via Natural or Artificial Opening Endoscopic | Procedure | ICD-10-PCS |
| 0D168Z9 | Bypass Stomach to Duodenum, Via Natural or Artificial Opening Endoscopic | Procedure | ICD-10-PCS |
| 0D168ZA | Bypass Stomach to Jejunum, Via Natural or Artificial Opening Endoscopic | Procedure | ICD-10-PCS |
| 0D168ZB | Bypass Stomach to Ileum, Via Natural or Artificial Opening Endoscopic | Procedure | ICD-10-PCS |
| 0D168ZL | Bypass Stomach to Transverse Colon, Via Natural or Artificial Opening Endoscopic | Procedure | ICD-10-PCS |
| 0D19079 | Bypass Duodenum to Duodenum with Autologous Tissue Substitute, Open Approach | Procedure | ICD-10-PCS |
| 0D1907A | Bypass Duodenum to Jejunum with Autologous Tissue Substitute, Open Approach | Procedure | ICD-10-PCS |
| 0D1907B | Bypass Duodenum to Ileum with Autologous Tissue Substitute, Open Approach | Procedure | ICD-10-PCS |
| 0D190J9 | Bypass Duodenum to Duodenum with Synthetic Substitute, Open Approach | Procedure | ICD-10-PCS |
| 0D190JA | Bypass Duodenum to Jejunum with Synthetic Substitute, Open Approach | Procedure | ICD-10-PCS |
| 0D190JB | Bypass Duodenum to Ileum with Synthetic Substitute, Open Approach | Procedure | ICD-10-PCS |
| 0D190K9 | Bypass Duodenum to Duodenum with Nonautologous Tissue Substitute, Open Approach | Procedure | ICD-10-PCS |
| 0D190KA | Bypass Duodenum to Jejunum with Nonautologous Tissue Substitute, Open Approach | Procedure | ICD-10-PCS |
| 0D190KB | Bypass Duodenum to Ileum with Nonautologous Tissue Substitute, Open Approach | Procedure | ICD-10-PCS |
| 0D190Z9 | Bypass Duodenum to Duodenum, Open Approach | Procedure | ICD-10-PCS |
| 0D190ZA | Bypass Duodenum to Jejunum, Open Approach | Procedure | ICD-10-PCS |
| 0D190ZB | Bypass Duodenum to Ileum, Open Approach | Procedure | ICD-10-PCS |
| 0D19479 | Bypass Duodenum to Duodenum with Autologous Tissue Substitute, Percutaneous Endoscopic Approach | Procedure | ICD-10-PCS |
| 0D1947A | Bypass Duodenum to Jejunum with Autologous Tissue Substitute, Percutaneous Endoscopic Approach | Procedure | ICD-10-PCS |
| 0D1947B | Bypass Duodenum to Ileum with Autologous Tissue Substitute, Percutaneous Endoscopic Approach | Procedure | ICD-10-PCS |
| 0D194J9 | Bypass Duodenum to Duodenum with Synthetic Substitute, Percutaneous Endoscopic Approach | Procedure | ICD-10-PCS |
| 0D194JA | Bypass Duodenum to Jejunum with Synthetic Substitute, Percutaneous Endoscopic Approach | Procedure | ICD-10-PCS |
| 0D194JB | Bypass Duodenum to Ileum with Synthetic Substitute, Percutaneous Endoscopic Approach | Procedure | ICD-10-PCS |
| 0D194K9 | Bypass Duodenum to Duodenum with Nonautologous Tissue Substitute, Percutaneous Endoscopic Approach | Procedure | ICD-10-PCS |
| 0D194KA | Bypass Duodenum to Jejunum with Nonautologous Tissue Substitute, Percutaneous Endoscopic Approach | Procedure | ICD-10-PCS |
| 0D194KB | Bypass Duodenum to Ileum with Nonautologous Tissue Substitute, Percutaneous Endoscopic Approach | Procedure | ICD-10-PCS |
| 0D194Z9 | Bypass Duodenum to Duodenum, Percutaneous Endoscopic Approach | Procedure | ICD-10-PCS |
| 0D194ZA | Bypass Duodenum to Jejunum, Percutaneous Endoscopic Approach | Procedure | ICD-10-PCS |
| 0D194ZB | Bypass Duodenum to Ileum, Percutaneous Endoscopic Approach | Procedure | ICD-10-PCS |
| 0D19879 | Bypass Duodenum to Duodenum with Autologous Tissue Substitute, Via Natural or Artificial Opening Endoscopic | Procedure | ICD-10-PCS |
| 0D1987A | Bypass Duodenum to Jejunum with Autologous Tissue Substitute, Via Natural or Artificial Opening Endoscopic | Procedure | ICD-10-PCS |
| 0D1987B | Bypass Duodenum to Ileum with Autologous Tissue Substitute, Via Natural or Artificial Opening Endoscopic | Procedure | ICD-10-PCS |
| 0D198J9 | Bypass Duodenum to Duodenum with Synthetic Substitute, Via Natural or Artificial Opening Endoscopic | Procedure | ICD-10-PCS |
| 0D198JA | Bypass Duodenum to Jejunum with Synthetic Substitute, Via Natural or Artificial Opening Endoscopic | Procedure | ICD-10-PCS |
| 0D198JB | Bypass Duodenum to Ileum with Synthetic Substitute, Via Natural or Artificial Opening Endoscopic | Procedure | ICD-10-PCS |
| 0D198K9 | Bypass Duodenum to Duodenum with Nonautologous Tissue Substitute, Via Natural or Artificial Opening Endoscopic | Procedure | ICD-10-PCS |
| 0D198KA | Bypass Duodenum to Jejunum with Nonautologous Tissue Substitute, Via Natural or Artificial Opening Endoscopic | Procedure | ICD-10-PCS |
| 0D198KB | Bypass Duodenum to Ileum with Nonautologous Tissue Substitute, Via Natural or Artificial Opening Endoscopic | Procedure | ICD-10-PCS |
| 0D198Z9 | Bypass Duodenum to Duodenum, Via Natural or Artificial Opening Endoscopic | Procedure | ICD-10-PCS |
| 0D198ZA | Bypass Duodenum to Jejunum, Via Natural or Artificial Opening Endoscopic | Procedure | ICD-10-PCS |
| 0D198ZB | Bypass Duodenum to Ileum, Via Natural or Artificial Opening Endoscopic | Procedure | ICD-10-PCS |
| 0D1A07A | Bypass Jejunum to Jejunum with Autologous Tissue Substitute, Open Approach | Procedure | ICD-10-PCS |
| 0D1A07B | Bypass Jejunum to Ileum with Autologous Tissue Substitute, Open Approach | Procedure | ICD-10-PCS |
| 0D1A0JA | Bypass Jejunum to Jejunum with Synthetic Substitute, Open Approach | Procedure | ICD-10-PCS |
| 0D1A0JB | Bypass Jejunum to Ileum with Synthetic Substitute, Open Approach | Procedure | ICD-10-PCS |
| 0D1A0KA | Bypass Jejunum to Jejunum with Nonautologous Tissue Substitute, Open Approach | Procedure | ICD-10-PCS |
| 0D1A0KB | Bypass Jejunum to Ileum with Nonautologous Tissue Substitute, Open Approach | Procedure | ICD-10-PCS |
| 0D1A0ZA | Bypass Jejunum to Jejunum, Open Approach | Procedure | ICD-10-PCS |
| 0D1A0ZB | Bypass Jejunum to Ileum, Open Approach | Procedure | ICD-10-PCS |
| 0D1A47A | Bypass Jejunum to Jejunum with Autologous Tissue Substitute, Percutaneous Endoscopic Approach | Procedure | ICD-10-PCS |
| 0D1A47B | Bypass Jejunum to Ileum with Autologous Tissue Substitute, Percutaneous Endoscopic Approach | Procedure | ICD-10-PCS |
| 0D1A4JA | Bypass Jejunum to Jejunum with Synthetic Substitute, Percutaneous Endoscopic Approach | Procedure | ICD-10-PCS |
| 0D1A4JB | Bypass Jejunum to Ileum with Synthetic Substitute, Percutaneous Endoscopic Approach | Procedure | ICD-10-PCS |
| 0D1A4KA | Bypass Jejunum to Jejunum with Nonautologous Tissue Substitute, Percutaneous Endoscopic Approach | Procedure | ICD-10-PCS |
| 0D1A4KB | Bypass Jejunum to Ileum with Nonautologous Tissue Substitute, Percutaneous Endoscopic Approach | Procedure | ICD-10-PCS |
| 0D1A4ZA | Bypass Jejunum to Jejunum, Percutaneous Endoscopic Approach | Procedure | ICD-10-PCS |
| 0D1A4ZB | Bypass Jejunum to Ileum, Percutaneous Endoscopic Approach | Procedure | ICD-10-PCS |
| 0D1A87A | Bypass Jejunum to Jejunum with Autologous Tissue Substitute, Via Natural or Artificial Opening Endoscopic | Procedure | ICD-10-PCS |
| 0D1A87B | Bypass Jejunum to Ileum with Autologous Tissue Substitute, Via Natural or Artificial Opening Endoscopic | Procedure | ICD-10-PCS |
| 0D1A8JA | Bypass Jejunum to Jejunum with Synthetic Substitute, Via Natural or Artificial Opening Endoscopic | Procedure | ICD-10-PCS |
| 0D1A8JB | Bypass Jejunum to Ileum with Synthetic Substitute, Via Natural or Artificial Opening Endoscopic | Procedure | ICD-10-PCS |
| 0D1A8KA | Bypass Jejunum to Jejunum with Nonautologous Tissue Substitute, Via Natural or Artificial Opening Endoscopic | Procedure | ICD-10-PCS |
| 0D1A8KB | Bypass Jejunum to Ileum with Nonautologous Tissue Substitute, Via Natural or Artificial Opening Endoscopic | Procedure | ICD-10-PCS |
| 0D1A8ZA | Bypass Jejunum to Jejunum, Via Natural or Artificial Opening Endoscopic | Procedure | ICD-10-PCS |
| 0D1A8ZB | Bypass Jejunum to Ileum, Via Natural or Artificial Opening Endoscopic | Procedure | ICD-10-PCS |
| 0D1A8ZH | Bypass Jejunum to Cecum, Via Natural or Artificial Opening Endoscopic | Procedure | ICD-10-PCS |
| 0D1B07B | Bypass Ileum to Ileum with Autologous Tissue Substitute, Open Approach | Procedure | ICD-10-PCS |
| 0D1B0JB | Bypass Ileum to Ileum with Synthetic Substitute, Open Approach | Procedure | ICD-10-PCS |
| 0D1B0KB | Bypass Ileum to Ileum with Nonautologous Tissue Substitute, Open Approach | Procedure | ICD-10-PCS |
| 0D1B0ZB | Bypass Ileum to Ileum, Open Approach | Procedure | ICD-10-PCS |
| 0D1B47B | Bypass Ileum to Ileum with Autologous Tissue Substitute, Percutaneous Endoscopic Approach | Procedure | ICD-10-PCS |
| 0D1B4JB | Bypass Ileum to Ileum with Synthetic Substitute, Percutaneous Endoscopic Approach | Procedure | ICD-10-PCS |
| 0D1B4KB | Bypass Ileum to Ileum with Nonautologous Tissue Substitute, Percutaneous Endoscopic Approach | Procedure | ICD-10-PCS |
| 0D1B4ZB | Bypass Ileum to Ileum, Percutaneous Endoscopic Approach | Procedure | ICD-10-PCS |
| 0D1B87B | Bypass Ileum to Ileum with Autologous Tissue Substitute, Via Natural or Artificial Opening Endoscopic | Procedure | ICD-10-PCS |
| 0D1B8JB | Bypass Ileum to Ileum with Synthetic Substitute, Via Natural or Artificial Opening Endoscopic | Procedure | ICD-10-PCS |
| 0D1B8KB | Bypass Ileum to Ileum with Nonautologous Tissue Substitute, Via Natural or Artificial Opening Endoscopic | Procedure | ICD-10-PCS |
| 0D1B8ZB | Bypass Ileum to Ileum, Via Natural or Artificial Opening Endoscopic | Procedure | ICD-10-PCS |
| 0D1B8ZH | Bypass Ileum to Cecum, Via Natural or Artificial Opening Endoscopic | Procedure | ICD-10-PCS |
| 0DB60Z3 | Excision of Stomach, Open Approach, Vertical | Procedure | ICD-10-PCS |
| 0DB60ZZ | Excision of Stomach, Open Approach | Procedure | ICD-10-PCS |
| 0DB63Z3 | Excision of Stomach, Percutaneous Approach, Vertical | Procedure | ICD-10-PCS |
| 0DB63ZZ | Excision of Stomach, Percutaneous Approach | Procedure | ICD-10-PCS |
| 0DB67Z3 | Excision of Stomach, Via Natural or Artificial Opening, Vertical | Procedure | ICD-10-PCS |
| 0DB67ZZ | Excision of Stomach, Via Natural or Artificial Opening | Procedure | ICD-10-PCS |
| 0DB68Z3 | Excision of Stomach, Via Natural or Artificial Opening Endoscopic, Vertical | Procedure | ICD-10-PCS |
| 0DB80ZZ | Excision of Small Intestine, Open Approach | Procedure | ICD-10-PCS |
| 0DB90ZZ | Excision of Duodenum, Open Approach | Procedure | ICD-10-PCS |
| 0DBB0ZZ | Excision of Ileum, Open Approach | Procedure | ICD-10-PCS |
| 0DQ64ZZ | Repair Stomach, Percutaneous Endoscopic Approach | Procedure | ICD-10-PCS |
| 0DV64CZ | Restriction of Stomach with Extraluminal Device, Percutaneous Endoscopic Approach | Procedure | ICD-10-PCS |
| 0F190Z3 | Bypass Common Bile Duct to Duodenum, Open Approach | Procedure | ICD-10-PCS |
| 43659 | Unlisted laparoscopy procedure, stomach | Procedure | CPT-4 |
| 43844 | GASTRIC BYPASS NOT ROUX-EN-Y GASTROENTEROSTOMY | Procedure | CPT-4 |
| S2082 | Laparoscopy, surgical; gastric restrictive procedure, adjustable gastric band includes placement of subcutaneous port | Procedure | HCPCS |
| S2085 | Laparoscopy, gastric restrictive procedure, with gastric bypass for morbid obesity, with short limb (less than 100 cm) roux-en-y gastroenterostomy | Procedure | HCPCS |
| **Ischemic Heart Disease** | | | |
| I20.0 | Unstable angina | Diagnosis | ICD-10-CM |
| I20.1 | Angina pectoris with documented spasm | Diagnosis | ICD-10-CM |
| I20.8 | Other forms of angina pectoris | Diagnosis | ICD-10-CM |
| I20.9 | Angina pectoris, unspecified | Diagnosis | ICD-10-CM |
| I21.01 | ST elevation (STEMI) myocardial infarction involving left main coronary artery | Diagnosis | ICD-10-CM |
| I21.02 | ST elevation (STEMI) myocardial infarction involving left anterior descending coronary artery | Diagnosis | ICD-10-CM |
| I21.09 | ST elevation (STEMI) myocardial infarction involving other coronary artery of anterior wall | Diagnosis | ICD-10-CM |
| I21.11 | ST elevation (STEMI) myocardial infarction involving right coronary artery | Diagnosis | ICD-10-CM |
| I21.19 | ST elevation (STEMI) myocardial infarction involving other coronary artery of inferior wall | Diagnosis | ICD-10-CM |
| I21.21 | ST elevation (STEMI) myocardial infarction involving left circumflex coronary artery | Diagnosis | ICD-10-CM |
| I21.29 | ST elevation (STEMI) myocardial infarction involving other sites | Diagnosis | ICD-10-CM |
| I21.3 | ST elevation (STEMI) myocardial infarction of unspecified site | Diagnosis | ICD-10-CM |
| I21.4 | Non-ST elevation (NSTEMI) myocardial infarction | Diagnosis | ICD-10-CM |
| I21.9 | Acute myocardial infarction, unspecified | Diagnosis | ICD-10-CM |
| I21.A1 | Myocardial infarction type 2 | Diagnosis | ICD-10-CM |
| I21.A9 | Other myocardial infarction type | Diagnosis | ICD-10-CM |
| I22.0 | Subsequent ST elevation (STEMI) myocardial infarction of anterior wall | Diagnosis | ICD-10-CM |
| I22.1 | Subsequent ST elevation (STEMI) myocardial infarction of inferior wall | Diagnosis | ICD-10-CM |
| I22.2 | Subsequent non-ST elevation (NSTEMI) myocardial infarction | Diagnosis | ICD-10-CM |
| I22.8 | Subsequent ST elevation (STEMI) myocardial infarction of other sites | Diagnosis | ICD-10-CM |
| I22.9 | Subsequent ST elevation (STEMI) myocardial infarction of unspecified site | Diagnosis | ICD-10-CM |
| I24.0 | Acute coronary thrombosis not resulting in myocardial infarction | Diagnosis | ICD-10-CM |
| I24.1 | Dressler's syndrome | Diagnosis | ICD-10-CM |
| I24.8 | Other forms of acute ischemic heart disease | Diagnosis | ICD-10-CM |
| I24.9 | Acute ischemic heart disease, unspecified | Diagnosis | ICD-10-CM |
| I25.10 | Atherosclerotic heart disease of native coronary artery without angina pectoris | Diagnosis | ICD-10-CM |
| I25.110 | Atherosclerotic heart disease of native coronary artery with unstable angina pectoris | Diagnosis | ICD-10-CM |
| I25.111 | Atherosclerotic heart disease of native coronary artery with angina pectoris with documented spasm | Diagnosis | ICD-10-CM |
| I25.118 | Atherosclerotic heart disease of native coronary artery with other forms of angina pectoris | Diagnosis | ICD-10-CM |
| I25.119 | Atherosclerotic heart disease of native coronary artery with unspecified angina pectoris | Diagnosis | ICD-10-CM |
| I25.2 | Old myocardial infarction | Diagnosis | ICD-10-CM |
| I25.3 | Aneurysm of heart | Diagnosis | ICD-10-CM |
| I25.41 | Coronary artery aneurysm | Diagnosis | ICD-10-CM |
| I25.42 | Coronary artery dissection | Diagnosis | ICD-10-CM |
| I25.5 | Ischemic cardiomyopathy | Diagnosis | ICD-10-CM |
| I25.6 | Silent myocardial ischemia | Diagnosis | ICD-10-CM |
| I25.700 | Atherosclerosis of coronary artery bypass graft(s), unspecified, with unstable angina pectoris | Diagnosis | ICD-10-CM |
| I25.701 | Atherosclerosis of coronary artery bypass graft(s), unspecified, with angina pectoris with documented spasm | Diagnosis | ICD-10-CM |
| I25.708 | Atherosclerosis of coronary artery bypass graft(s), unspecified, with other forms of angina pectoris | Diagnosis | ICD-10-CM |
| I25.709 | Atherosclerosis of coronary artery bypass graft(s), unspecified, with unspecified angina pectoris | Diagnosis | ICD-10-CM |
| I25.710 | Atherosclerosis of autologous vein coronary artery bypass graft(s) with unstable angina pectoris | Diagnosis | ICD-10-CM |
| I25.711 | Atherosclerosis of autologous vein coronary artery bypass graft(s) with angina pectoris with documented spasm | Diagnosis | ICD-10-CM |
| I25.718 | Atherosclerosis of autologous vein coronary artery bypass graft(s) with other forms of angina pectoris | Diagnosis | ICD-10-CM |
| I25.719 | Atherosclerosis of autologous vein coronary artery bypass graft(s) with unspecified angina pectoris | Diagnosis | ICD-10-CM |
| I25.720 | Atherosclerosis of autologous artery coronary artery bypass graft(s) with unstable angina pectoris | Diagnosis | ICD-10-CM |
| I25.721 | Atherosclerosis of autologous artery coronary artery bypass graft(s) with angina pectoris with documented spasm | Diagnosis | ICD-10-CM |
| I25.728 | Atherosclerosis of autologous artery coronary artery bypass graft(s) with other forms of angina pectoris | Diagnosis | ICD-10-CM |
| I25.729 | Atherosclerosis of autologous artery coronary artery bypass graft(s) with unspecified angina pectoris | Diagnosis | ICD-10-CM |
| I25.730 | Atherosclerosis of nonautologous biological coronary artery bypass graft(s) with unstable angina pectoris | Diagnosis | ICD-10-CM |
| I25.731 | Atherosclerosis of nonautologous biological coronary artery bypass graft(s) with angina pectoris with documented spasm | Diagnosis | ICD-10-CM |
| I25.738 | Atherosclerosis of nonautologous biological coronary artery bypass graft(s) with other forms of angina pectoris | Diagnosis | ICD-10-CM |
| I25.739 | Atherosclerosis of nonautologous biological coronary artery bypass graft(s) with unspecified angina pectoris | Diagnosis | ICD-10-CM |
| I25.750 | Atherosclerosis of native coronary artery of transplanted heart with unstable angina | Diagnosis | ICD-10-CM |
| I25.751 | Atherosclerosis of native coronary artery of transplanted heart with angina pectoris with documented spasm | Diagnosis | ICD-10-CM |
| I25.758 | Atherosclerosis of native coronary artery of transplanted heart with other forms of angina pectoris | Diagnosis | ICD-10-CM |
| I25.759 | Atherosclerosis of native coronary artery of transplanted heart with unspecified angina pectoris | Diagnosis | ICD-10-CM |
| I25.760 | Atherosclerosis of bypass graft of coronary artery of transplanted heart with unstable angina | Diagnosis | ICD-10-CM |
| I25.761 | Atherosclerosis of bypass graft of coronary artery of transplanted heart with angina pectoris with documented spasm | Diagnosis | ICD-10-CM |
| I25.768 | Atherosclerosis of bypass graft of coronary artery of transplanted heart with other forms of angina pectoris | Diagnosis | ICD-10-CM |
| I25.769 | Atherosclerosis of bypass graft of coronary artery of transplanted heart with unspecified angina pectoris | Diagnosis | ICD-10-CM |
| I25.790 | Atherosclerosis of other coronary artery bypass graft(s) with unstable angina pectoris | Diagnosis | ICD-10-CM |
| I25.791 | Atherosclerosis of other coronary artery bypass graft(s) with angina pectoris with documented spasm | Diagnosis | ICD-10-CM |
| I25.798 | Atherosclerosis of other coronary artery bypass graft(s) with other forms of angina pectoris | Diagnosis | ICD-10-CM |
| I25.799 | Atherosclerosis of other coronary artery bypass graft(s) with unspecified angina pectoris | Diagnosis | ICD-10-CM |
| I25.810 | Atherosclerosis of coronary artery bypass graft(s) without angina pectoris | Diagnosis | ICD-10-CM |
| I25.811 | Atherosclerosis of native coronary artery of transplanted heart without angina pectoris | Diagnosis | ICD-10-CM |
| I25.812 | Atherosclerosis of bypass graft of coronary artery of transplanted heart without angina pectoris | Diagnosis | ICD-10-CM |
| I25.82 | Chronic total occlusion of coronary artery | Diagnosis | ICD-10-CM |
| I25.83 | Coronary atherosclerosis due to lipid rich plaque | Diagnosis | ICD-10-CM |
| I25.84 | Coronary atherosclerosis due to calcified coronary lesion | Diagnosis | ICD-10-CM |
| I25.89 | Other forms of chronic ischemic heart disease | Diagnosis | ICD-10-CM |
| I25.9 | Chronic ischemic heart disease, unspecified | Diagnosis | ICD-10-CM |
| **Heart Failure** | | | |
| I50.1 | Left ventricular failure, unspecified | Diagnosis | ICD-10-CM |
| I50.20 | Unspecified systolic (congestive) heart failure | Diagnosis | ICD-10-CM |
| I50.21 | Acute systolic (congestive) heart failure | Diagnosis | ICD-10-CM |
| I50.22 | Chronic systolic (congestive) heart failure | Diagnosis | ICD-10-CM |
| I50.23 | Acute on chronic systolic (congestive) heart failure | Diagnosis | ICD-10-CM |
| I50.30 | Unspecified diastolic (congestive) heart failure | Diagnosis | ICD-10-CM |
| I50.31 | Acute diastolic (congestive) heart failure | Diagnosis | ICD-10-CM |
| I50.32 | Chronic diastolic (congestive) heart failure | Diagnosis | ICD-10-CM |
| I50.33 | Acute on chronic diastolic (congestive) heart failure | Diagnosis | ICD-10-CM |
| I50.40 | Unspecified combined systolic (congestive) and diastolic (congestive) heart failure | Diagnosis | ICD-10-CM |
| I50.41 | Acute combined systolic (congestive) and diastolic (congestive) heart failure | Diagnosis | ICD-10-CM |
| I50.42 | Chronic combined systolic (congestive) and diastolic (congestive) heart failure | Diagnosis | ICD-10-CM |
| I50.43 | Acute on chronic combined systolic (congestive) and diastolic (congestive) heart failure | Diagnosis | ICD-10-CM |
| I50.810 | Right heart failure, unspecified | Diagnosis | ICD-10-CM |
| I50.811 | Acute right heart failure | Diagnosis | ICD-10-CM |
| I50.812 | Chronic right heart failure | Diagnosis | ICD-10-CM |
| I50.813 | Acute on chronic right heart failure | Diagnosis | ICD-10-CM |
| I50.814 | Right heart failure due to left heart failure | Diagnosis | ICD-10-CM |
| I50.82 | Biventricular heart failure | Diagnosis | ICD-10-CM |
| I50.83 | High output heart failure | Diagnosis | ICD-10-CM |
| I50.84 | End stage heart failure | Diagnosis | ICD-10-CM |
| I50.89 | Other heart failure | Diagnosis | ICD-10-CM |
| I50.9 | Heart failure, unspecified | Diagnosis | ICD-10-CM |
| **Other Heart Diseases** | | | |
| I23.0 | Hemopericardium as current complication following acute myocardial infarction | Diagnosis | ICD-10-CM |
| I23.1 | Atrial septal defect as current complication following acute myocardial infarction | Diagnosis | ICD-10-CM |
| I23.2 | Ventricular septal defect as current complication following acute myocardial infarction | Diagnosis | ICD-10-CM |
| I23.3 | Rupture of cardiac wall without hemopericardium as current complication following acute myocardial infarction | Diagnosis | ICD-10-CM |
| I23.4 | Rupture of chordae tendineae as current complication following acute myocardial infarction | Diagnosis | ICD-10-CM |
| I23.5 | Rupture of papillary muscle as current complication following acute myocardial infarction | Diagnosis | ICD-10-CM |
| I23.6 | Thrombosis of atrium, auricular appendage, and ventricle as current complications following acute myocardial infarction | Diagnosis | ICD-10-CM |
| I23.7 | Postinfarction angina | Diagnosis | ICD-10-CM |
| I23.8 | Other current complications following acute myocardial infarction | Diagnosis | ICD-10-CM |
| I25.10 | Atherosclerotic heart disease of native coronary artery without angina pectoris | Diagnosis | ICD-10-CM |
| I30.0 | Acute nonspecific idiopathic pericarditis | Diagnosis | ICD-10-CM |
| I30.1 | Infective pericarditis | Diagnosis | ICD-10-CM |
| I30.8 | Other forms of acute pericarditis | Diagnosis | ICD-10-CM |
| I30.9 | Acute pericarditis, unspecified | Diagnosis | ICD-10-CM |
| I31.0 | Chronic adhesive pericarditis | Diagnosis | ICD-10-CM |
| I31.1 | Chronic constrictive pericarditis | Diagnosis | ICD-10-CM |
| I31.2 | Hemopericardium, not elsewhere classified | Diagnosis | ICD-10-CM |
| I31.3 | Pericardial effusion (noninflammatory) | Diagnosis | ICD-10-CM |
| I31.4 | Cardiac tamponade | Diagnosis | ICD-10-CM |
| I31.8 | Other specified diseases of pericardium | Diagnosis | ICD-10-CM |
| I31.9 | Disease of pericardium, unspecified | Diagnosis | ICD-10-CM |
| I32 | Pericarditis in diseases classified elsewhere | Diagnosis | ICD-10-CM |
| I33.0 | Acute and subacute infective endocarditis | Diagnosis | ICD-10-CM |
| I33.9 | Acute and subacute endocarditis, unspecified | Diagnosis | ICD-10-CM |
| I34.0 | Nonrheumatic mitral (valve) insufficiency | Diagnosis | ICD-10-CM |
| I34.1 | Nonrheumatic mitral (valve) prolapse | Diagnosis | ICD-10-CM |
| I34.2 | Nonrheumatic mitral (valve) stenosis | Diagnosis | ICD-10-CM |
| I34.8 | Other nonrheumatic mitral valve disorders | Diagnosis | ICD-10-CM |
| I34.9 | Nonrheumatic mitral valve disorder, unspecified | Diagnosis | ICD-10-CM |
| I35.0 | Nonrheumatic aortic (valve) stenosis | Diagnosis | ICD-10-CM |
| I35.1 | Nonrheumatic aortic (valve) insufficiency | Diagnosis | ICD-10-CM |
| I35.2 | Nonrheumatic aortic (valve) stenosis with insufficiency | Diagnosis | ICD-10-CM |
| I35.8 | Other nonrheumatic aortic valve disorders | Diagnosis | ICD-10-CM |
| I35.9 | Nonrheumatic aortic valve disorder, unspecified | Diagnosis | ICD-10-CM |
| I36.0 | Nonrheumatic tricuspid (valve) stenosis | Diagnosis | ICD-10-CM |
| I36.1 | Nonrheumatic tricuspid (valve) insufficiency | Diagnosis | ICD-10-CM |
| I36.2 | Nonrheumatic tricuspid (valve) stenosis with insufficiency | Diagnosis | ICD-10-CM |
| I36.8 | Other nonrheumatic tricuspid valve disorders | Diagnosis | ICD-10-CM |
| I36.9 | Nonrheumatic tricuspid valve disorder, unspecified | Diagnosis | ICD-10-CM |
| I37.0 | Nonrheumatic pulmonary valve stenosis | Diagnosis | ICD-10-CM |
| I37.1 | Nonrheumatic pulmonary valve insufficiency | Diagnosis | ICD-10-CM |
| I37.2 | Nonrheumatic pulmonary valve stenosis with insufficiency | Diagnosis | ICD-10-CM |
| I37.8 | Other nonrheumatic pulmonary valve disorders | Diagnosis | ICD-10-CM |
| I37.9 | Nonrheumatic pulmonary valve disorder, unspecified | Diagnosis | ICD-10-CM |
| I38 | Endocarditis, valve unspecified | Diagnosis | ICD-10-CM |
| I39 | Endocarditis and heart valve disorders in diseases classified elsewhere | Diagnosis | ICD-10-CM |
| I40.0 | Infective myocarditis | Diagnosis | ICD-10-CM |
| I40.1 | Isolated myocarditis | Diagnosis | ICD-10-CM |
| I40.8 | Other acute myocarditis | Diagnosis | ICD-10-CM |
| I40.9 | Acute myocarditis, unspecified | Diagnosis | ICD-10-CM |
| I41 | Myocarditis in diseases classified elsewhere | Diagnosis | ICD-10-CM |
| I42.0 | Dilated cardiomyopathy | Diagnosis | ICD-10-CM |
| I42.1 | Obstructive hypertrophic cardiomyopathy | Diagnosis | ICD-10-CM |
| I42.2 | Other hypertrophic cardiomyopathy | Diagnosis | ICD-10-CM |
| I42.3 | Endomyocardial (eosinophilic) disease | Diagnosis | ICD-10-CM |
| I42.4 | Endocardial fibroelastosis | Diagnosis | ICD-10-CM |
| I42.5 | Other restrictive cardiomyopathy | Diagnosis | ICD-10-CM |
| I42.6 | Alcoholic cardiomyopathy | Diagnosis | ICD-10-CM |
| I42.7 | Cardiomyopathy due to drug and external agent | Diagnosis | ICD-10-CM |
| I42.8 | Other cardiomyopathies | Diagnosis | ICD-10-CM |
| I42.9 | Cardiomyopathy, unspecified | Diagnosis | ICD-10-CM |
| I43 | Cardiomyopathy in diseases classified elsewhere | Diagnosis | ICD-10-CM |
| I44.0 | Atrioventricular block, first degree | Diagnosis | ICD-10-CM |
| I44.1 | Atrioventricular block, second degree | Diagnosis | ICD-10-CM |
| I44.2 | Atrioventricular block, complete | Diagnosis | ICD-10-CM |
| I44.30 | Unspecified atrioventricular block | Diagnosis | ICD-10-CM |
| I44.39 | Other atrioventricular block | Diagnosis | ICD-10-CM |
| I44.4 | Left anterior fascicular block | Diagnosis | ICD-10-CM |
| I44.5 | Left posterior fascicular block | Diagnosis | ICD-10-CM |
| I44.60 | Unspecified fascicular block | Diagnosis | ICD-10-CM |
| I44.69 | Other fascicular block | Diagnosis | ICD-10-CM |
| I44.7 | Left bundle-branch block, unspecified | Diagnosis | ICD-10-CM |
| I45.0 | Right fascicular block | Diagnosis | ICD-10-CM |
| I45.10 | Unspecified right bundle-branch block | Diagnosis | ICD-10-CM |
| I45.19 | Other right bundle-branch block | Diagnosis | ICD-10-CM |
| I45.2 | Bifascicular block | Diagnosis | ICD-10-CM |
| I45.3 | Trifascicular block | Diagnosis | ICD-10-CM |
| I45.4 | Nonspecific intraventricular block | Diagnosis | ICD-10-CM |
| I45.5 | Other specified heart block | Diagnosis | ICD-10-CM |
| I45.6 | Pre-excitation syndrome | Diagnosis | ICD-10-CM |
| I45.81 | Long QT syndrome | Diagnosis | ICD-10-CM |
| I45.89 | Other specified conduction disorders | Diagnosis | ICD-10-CM |
| I45.9 | Conduction disorder, unspecified | Diagnosis | ICD-10-CM |
| I46.2 | Cardiac arrest due to underlying cardiac condition | Diagnosis | ICD-10-CM |
| I46.8 | Cardiac arrest due to other underlying condition | Diagnosis | ICD-10-CM |
| I46.9 | Cardiac arrest, cause unspecified | Diagnosis | ICD-10-CM |
| I47.0 | Re-entry ventricular arrhythmia | Diagnosis | ICD-10-CM |
| I47.1 | Supraventricular tachycardia | Diagnosis | ICD-10-CM |
| I47.2 | Ventricular tachycardia | Diagnosis | ICD-10-CM |
| I47.9 | Paroxysmal tachycardia, unspecified | Diagnosis | ICD-10-CM |
| I48.0 | Paroxysmal atrial fibrillation | Diagnosis | ICD-10-CM |
| I48.1 | Persistent atrial fibrillation | Diagnosis | ICD-10-CM |
| I48.2 | Chronic atrial fibrillation | Diagnosis | ICD-10-CM |
| I48.3 | Typical atrial flutter | Diagnosis | ICD-10-CM |
| I48.4 | Atypical atrial flutter | Diagnosis | ICD-10-CM |
| I48.91 | Unspecified atrial fibrillation | Diagnosis | ICD-10-CM |
| I48.92 | Unspecified atrial flutter | Diagnosis | ICD-10-CM |
| I49.01 | Ventricular fibrillation | Diagnosis | ICD-10-CM |
| I49.02 | Ventricular flutter | Diagnosis | ICD-10-CM |
| I49.1 | Atrial premature depolarization | Diagnosis | ICD-10-CM |
| I49.2 | Junctional premature depolarization | Diagnosis | ICD-10-CM |
| I49.3 | Ventricular premature depolarization | Diagnosis | ICD-10-CM |
| I49.40 | Unspecified premature depolarization | Diagnosis | ICD-10-CM |
| I49.49 | Other premature depolarization | Diagnosis | ICD-10-CM |
| I49.5 | Sick sinus syndrome | Diagnosis | ICD-10-CM |
| I49.8 | Other specified cardiac arrhythmias | Diagnosis | ICD-10-CM |
| I49.9 | Cardiac arrhythmia, unspecified | Diagnosis | ICD-10-CM |
| I51.0 | Cardiac septal defect, acquired | Diagnosis | ICD-10-CM |
| I51.1 | Rupture of chordae tendineae, not elsewhere classified | Diagnosis | ICD-10-CM |
| I51.2 | Rupture of papillary muscle, not elsewhere classified | Diagnosis | ICD-10-CM |
| I51.3 | Intracardiac thrombosis, not elsewhere classified | Diagnosis | ICD-10-CM |
| I51.4 | Myocarditis, unspecified | Diagnosis | ICD-10-CM |
| I51.5 | Myocardial degeneration | Diagnosis | ICD-10-CM |
| I51.7 | Cardiomegaly | Diagnosis | ICD-10-CM |
| I51.81 | Takotsubo syndrome | Diagnosis | ICD-10-CM |
| I51.89 | Other ill-defined heart diseases | Diagnosis | ICD-10-CM |
| I51.9 | Heart disease, unspecified | Diagnosis | ICD-10-CM |
| I52 | Other heart disorders in diseases classified elsewhere | Diagnosis | ICD-10-CM |
| I97.0 | Postcardiotomy syndrome | Diagnosis | ICD-10-CM |
| I97.110 | Postprocedural cardiac insufficiency following cardiac surgery | Diagnosis | ICD-10-CM |
| I97.111 | Postprocedural cardiac insufficiency following other surgery | Diagnosis | ICD-10-CM |
| I97.120 | Postprocedural cardiac arrest following cardiac surgery | Diagnosis | ICD-10-CM |
| I97.121 | Postprocedural cardiac arrest following other surgery | Diagnosis | ICD-10-CM |
| I97.130 | Postprocedural heart failure following cardiac surgery | Diagnosis | ICD-10-CM |
| I97.131 | Postprocedural heart failure following other surgery | Diagnosis | ICD-10-CM |
| I97.190 | Other postprocedural cardiac functional disturbances following cardiac surgery | Diagnosis | ICD-10-CM |
| I97.191 | Other postprocedural cardiac functional disturbances following other surgery | Diagnosis | ICD-10-CM |
| M32.11 | Endocarditis in systemic lupus erythematosus | Diagnosis | ICD-10-CM |
| M32.12 | Pericarditis in systemic lupus erythematosus | Diagnosis | ICD-10-CM |
| R00.1 | Bradycardia, unspecified | Diagnosis | ICD-10-CM |
| **Renal Disorders** | | | |
| E09.21 | Drug or chemical induced diabetes mellitus with diabetic nephropathy | Diagnosis | ICD-10-CM |
| E09.22 | Drug or chemical induced diabetes mellitus with diabetic chronic kidney disease | Diagnosis | ICD-10-CM |
| E09.29 | Drug or chemical induced diabetes mellitus with other diabetic kidney complication | Diagnosis | ICD-10-CM |
| M32.14 | Glomerular disease in systemic lupus erythematosus | Diagnosis | ICD-10-CM |
| M32.15 | Tubulo-interstitial nephropathy in systemic lupus erythematosus | Diagnosis | ICD-10-CM |
| M35.04 | Sicca syndrome with tubulo-interstitial nephropathy | Diagnosis | ICD-10-CM |
| N00.0 | Acute nephritic syndrome with minor glomerular abnormality | Diagnosis | ICD-10-CM |
| N00.1 | Acute nephritic syndrome with focal and segmental glomerular lesions | Diagnosis | ICD-10-CM |
| N00.2 | Acute nephritic syndrome with diffuse membranous glomerulonephritis | Diagnosis | ICD-10-CM |
| N00.3 | Acute nephritic syndrome with diffuse mesangial proliferative glomerulonephritis | Diagnosis | ICD-10-CM |
| N00.4 | Acute nephritic syndrome with diffuse endocapillary proliferative glomerulonephritis | Diagnosis | ICD-10-CM |
| N00.5 | Acute nephritic syndrome with diffuse mesangiocapillary glomerulonephritis | Diagnosis | ICD-10-CM |
| N00.6 | Acute nephritic syndrome with dense deposit disease | Diagnosis | ICD-10-CM |
| N00.7 | Acute nephritic syndrome with diffuse crescentic glomerulonephritis | Diagnosis | ICD-10-CM |
| N00.8 | Acute nephritic syndrome with other morphologic changes | Diagnosis | ICD-10-CM |
| N00.9 | Acute nephritic syndrome with unspecified morphologic changes | Diagnosis | ICD-10-CM |
| N01.0 | Rapidly progressive nephritic syndrome with minor glomerular abnormality | Diagnosis | ICD-10-CM |
| N01.1 | Rapidly progressive nephritic syndrome with focal and segmental glomerular lesions | Diagnosis | ICD-10-CM |
| N01.2 | Rapidly progressive nephritic syndrome with diffuse membranous glomerulonephritis | Diagnosis | ICD-10-CM |
| N01.3 | Rapidly progressive nephritic syndrome with diffuse mesangial proliferative glomerulonephritis | Diagnosis | ICD-10-CM |
| N01.4 | Rapidly progressive nephritic syndrome with diffuse endocapillary proliferative glomerulonephritis | Diagnosis | ICD-10-CM |
| N01.5 | Rapidly progressive nephritic syndrome with diffuse mesangiocapillary glomerulonephritis | Diagnosis | ICD-10-CM |
| N01.6 | Rapidly progressive nephritic syndrome with dense deposit disease | Diagnosis | ICD-10-CM |
| N01.7 | Rapidly progressive nephritic syndrome with diffuse crescentic glomerulonephritis | Diagnosis | ICD-10-CM |
| N01.8 | Rapidly progressive nephritic syndrome with other morphologic changes | Diagnosis | ICD-10-CM |
| N01.9 | Rapidly progressive nephritic syndrome with unspecified morphologic changes | Diagnosis | ICD-10-CM |
| N02.0 | Recurrent and persistent hematuria with minor glomerular abnormality | Diagnosis | ICD-10-CM |
| N02.1 | Recurrent and persistent hematuria with focal and segmental glomerular lesions | Diagnosis | ICD-10-CM |
| N02.2 | Recurrent and persistent hematuria with diffuse membranous glomerulonephritis | Diagnosis | ICD-10-CM |
| N02.3 | Recurrent and persistent hematuria with diffuse mesangial proliferative glomerulonephritis | Diagnosis | ICD-10-CM |
| N02.4 | Recurrent and persistent hematuria with diffuse endocapillary proliferative glomerulonephritis | Diagnosis | ICD-10-CM |
| N02.5 | Recurrent and persistent hematuria with diffuse mesangiocapillary glomerulonephritis | Diagnosis | ICD-10-CM |
| N02.6 | Recurrent and persistent hematuria with dense deposit disease | Diagnosis | ICD-10-CM |
| N02.7 | Recurrent and persistent hematuria with diffuse crescentic glomerulonephritis | Diagnosis | ICD-10-CM |
| N02.8 | Recurrent and persistent hematuria with other morphologic changes | Diagnosis | ICD-10-CM |
| N02.9 | Recurrent and persistent hematuria with unspecified morphologic changes | Diagnosis | ICD-10-CM |
| N03.0 | Chronic nephritic syndrome with minor glomerular abnormality | Diagnosis | ICD-10-CM |
| N03.1 | Chronic nephritic syndrome with focal and segmental glomerular lesions | Diagnosis | ICD-10-CM |
| N03.2 | Chronic nephritic syndrome with diffuse membranous glomerulonephritis | Diagnosis | ICD-10-CM |
| N03.3 | Chronic nephritic syndrome with diffuse mesangial proliferative glomerulonephritis | Diagnosis | ICD-10-CM |
| N03.4 | Chronic nephritic syndrome with diffuse endocapillary proliferative glomerulonephritis | Diagnosis | ICD-10-CM |
| N03.5 | Chronic nephritic syndrome with diffuse mesangiocapillary glomerulonephritis | Diagnosis | ICD-10-CM |
| N03.6 | Chronic nephritic syndrome with dense deposit disease | Diagnosis | ICD-10-CM |
| N03.7 | Chronic nephritic syndrome with diffuse crescentic glomerulonephritis | Diagnosis | ICD-10-CM |
| N03.8 | Chronic nephritic syndrome with other morphologic changes | Diagnosis | ICD-10-CM |
| N03.9 | Chronic nephritic syndrome with unspecified morphologic changes | Diagnosis | ICD-10-CM |
| N04.0 | Nephrotic syndrome with minor glomerular abnormality | Diagnosis | ICD-10-CM |
| N04.1 | Nephrotic syndrome with focal and segmental glomerular lesions | Diagnosis | ICD-10-CM |
| N04.2 | Nephrotic syndrome with diffuse membranous glomerulonephritis | Diagnosis | ICD-10-CM |
| N04.3 | Nephrotic syndrome with diffuse mesangial proliferative glomerulonephritis | Diagnosis | ICD-10-CM |
| N04.4 | Nephrotic syndrome with diffuse endocapillary proliferative glomerulonephritis | Diagnosis | ICD-10-CM |
| N04.5 | Nephrotic syndrome with diffuse mesangiocapillary glomerulonephritis | Diagnosis | ICD-10-CM |
| N04.6 | Nephrotic syndrome with dense deposit disease | Diagnosis | ICD-10-CM |
| N04.7 | Nephrotic syndrome with diffuse crescentic glomerulonephritis | Diagnosis | ICD-10-CM |
| N04.8 | Nephrotic syndrome with other morphologic changes | Diagnosis | ICD-10-CM |
| N04.9 | Nephrotic syndrome with unspecified morphologic changes | Diagnosis | ICD-10-CM |
| N05.0 | Unspecified nephritic syndrome with minor glomerular abnormality | Diagnosis | ICD-10-CM |
| N05.1 | Unspecified nephritic syndrome with focal and segmental glomerular lesions | Diagnosis | ICD-10-CM |
| N05.2 | Unspecified nephritic syndrome with diffuse membranous glomerulonephritis | Diagnosis | ICD-10-CM |
| N05.3 | Unspecified nephritic syndrome with diffuse mesangial proliferative glomerulonephritis | Diagnosis | ICD-10-CM |
| N05.4 | Unspecified nephritic syndrome with diffuse endocapillary proliferative glomerulonephritis | Diagnosis | ICD-10-CM |
| N05.5 | Unspecified nephritic syndrome with diffuse mesangiocapillary glomerulonephritis | Diagnosis | ICD-10-CM |
| N05.6 | Unspecified nephritic syndrome with dense deposit disease | Diagnosis | ICD-10-CM |
| N05.7 | Unspecified nephritic syndrome with diffuse crescentic glomerulonephritis | Diagnosis | ICD-10-CM |
| N05.8 | Unspecified nephritic syndrome with other morphologic changes | Diagnosis | ICD-10-CM |
| N05.9 | Unspecified nephritic syndrome with unspecified morphologic changes | Diagnosis | ICD-10-CM |
| N07.0 | Hereditary nephropathy, not elsewhere classified with minor glomerular abnormality | Diagnosis | ICD-10-CM |
| N07.1 | Hereditary nephropathy, not elsewhere classified with focal and segmental glomerular lesions | Diagnosis | ICD-10-CM |
| N07.2 | Hereditary nephropathy, not elsewhere classified with diffuse membranous glomerulonephritis | Diagnosis | ICD-10-CM |
| N07.3 | Hereditary nephropathy, not elsewhere classified with diffuse mesangial proliferative glomerulonephritis | Diagnosis | ICD-10-CM |
| N07.4 | Hereditary nephropathy, not elsewhere classified with diffuse endocapillary proliferative glomerulonephritis | Diagnosis | ICD-10-CM |
| N07.5 | Hereditary nephropathy, not elsewhere classified with diffuse mesangiocapillary glomerulonephritis | Diagnosis | ICD-10-CM |
| N07.6 | Hereditary nephropathy, not elsewhere classified with dense deposit disease | Diagnosis | ICD-10-CM |
| N07.7 | Hereditary nephropathy, not elsewhere classified with diffuse crescentic glomerulonephritis | Diagnosis | ICD-10-CM |
| N07.8 | Hereditary nephropathy, not elsewhere classified with other morphologic lesions | Diagnosis | ICD-10-CM |
| N07.9 | Hereditary nephropathy, not elsewhere classified with unspecified morphologic lesions | Diagnosis | ICD-10-CM |
| N08 | Glomerular disorders in diseases classified elsewhere | Diagnosis | ICD-10-CM |
| N10 | Acute pyelonephritis | Diagnosis | ICD-10-CM |
| N11.0 | Nonobstructive reflux-associated chronic pyelonephritis | Diagnosis | ICD-10-CM |
| N11.1 | Chronic obstructive pyelonephritis | Diagnosis | ICD-10-CM |
| N11.8 | Other chronic tubulo-interstitial nephritis | Diagnosis | ICD-10-CM |
| N11.9 | Chronic tubulo-interstitial nephritis, unspecified | Diagnosis | ICD-10-CM |
| N12 | Tubulo-interstitial nephritis, not specified as acute or chronic | Diagnosis | ICD-10-CM |
| N13.0 | Hydronephrosis with ureteropelvic junction obstruction | Diagnosis | ICD-10-CM |
| N13.1 | Hydronephrosis with ureteral stricture, not elsewhere classified | Diagnosis | ICD-10-CM |
| N13.2 | Hydronephrosis with renal and ureteral calculous obstruction | Diagnosis | ICD-10-CM |
| N13.30 | Unspecified hydronephrosis | Diagnosis | ICD-10-CM |
| N13.39 | Other hydronephrosis | Diagnosis | ICD-10-CM |
| N13.4 | Hydroureter | Diagnosis | ICD-10-CM |
| N13.5 | Crossing vessel and stricture of ureter without hydronephrosis | Diagnosis | ICD-10-CM |
| N13.6 | Pyonephrosis | Diagnosis | ICD-10-CM |
| N13.70 | Vesicoureteral-reflux, unspecified | Diagnosis | ICD-10-CM |
| N13.71 | Vesicoureteral-reflux without reflux nephropathy | Diagnosis | ICD-10-CM |
| N13.721 | Vesicoureteral-reflux with reflux nephropathy without hydroureter, unilateral | Diagnosis | ICD-10-CM |
| N13.722 | Vesicoureteral-reflux with reflux nephropathy without hydroureter, bilateral | Diagnosis | ICD-10-CM |
| N13.729 | Vesicoureteral-reflux with reflux nephropathy without hydroureter, unspecified | Diagnosis | ICD-10-CM |
| N13.731 | Vesicoureteral-reflux with reflux nephropathy with hydroureter, unilateral | Diagnosis | ICD-10-CM |
| N13.732 | Vesicoureteral-reflux with reflux nephropathy with hydroureter, bilateral | Diagnosis | ICD-10-CM |
| N13.739 | Vesicoureteral-reflux with reflux nephropathy with hydroureter, unspecified | Diagnosis | ICD-10-CM |
| N13.8 | Other obstructive and reflux uropathy | Diagnosis | ICD-10-CM |
| N13.9 | Obstructive and reflux uropathy, unspecified | Diagnosis | ICD-10-CM |
| N14.0 | Analgesic nephropathy | Diagnosis | ICD-10-CM |
| N14.1 | Nephropathy induced by other drugs, medicaments and biological substances | Diagnosis | ICD-10-CM |
| N14.2 | Nephropathy induced by unspecified drug, medicament or biological substance | Diagnosis | ICD-10-CM |
| N14.3 | Nephropathy induced by heavy metals | Diagnosis | ICD-10-CM |
| N14.4 | Toxic nephropathy, not elsewhere classified | Diagnosis | ICD-10-CM |
| N15.0 | Balkan nephropathy | Diagnosis | ICD-10-CM |
| N15.1 | Renal and perinephric abscess | Diagnosis | ICD-10-CM |
| N15.8 | Other specified renal tubulo-interstitial diseases | Diagnosis | ICD-10-CM |
| N15.9 | Renal tubulo-interstitial disease, unspecified | Diagnosis | ICD-10-CM |
| N16 | Renal tubulo-interstitial disorders in diseases classified elsewhere | Diagnosis | ICD-10-CM |
| N17.0 | Acute kidney failure with tubular necrosis | Diagnosis | ICD-10-CM |
| N17.1 | Acute kidney failure with acute cortical necrosis | Diagnosis | ICD-10-CM |
| N17.2 | Acute kidney failure with medullary necrosis | Diagnosis | ICD-10-CM |
| N17.8 | Other acute kidney failure | Diagnosis | ICD-10-CM |
| N17.9 | Acute kidney failure, unspecified | Diagnosis | ICD-10-CM |
| N18.1 | Chronic kidney disease, stage 1 | Diagnosis | ICD-10-CM |
| N18.2 | Chronic kidney disease, stage 2 (mild) | Diagnosis | ICD-10-CM |
| N18.3 | Chronic kidney disease, stage 3 (moderate) | Diagnosis | ICD-10-CM |
| N18.4 | Chronic kidney disease, stage 4 (severe) | Diagnosis | ICD-10-CM |
| N18.5 | Chronic kidney disease, stage 5 | Diagnosis | ICD-10-CM |
| N18.6 | End stage renal disease | Diagnosis | ICD-10-CM |
| N18.9 | Chronic kidney disease, unspecified | Diagnosis | ICD-10-CM |
| N19 | Unspecified kidney failure | Diagnosis | ICD-10-CM |
| N25.89 | Other disorders resulting from impaired renal tubular function | Diagnosis | ICD-10-CM |
| N25.9 | Disorder resulting from impaired renal tubular function, unspecified | Diagnosis | ICD-10-CM |
| N28.89 | Other specified disorders of kidney and ureter | Diagnosis | ICD-10-CM |
| N28.9 | Disorder of kidney and ureter, unspecified | Diagnosis | ICD-10-CM |
| N29 | Other disorders of kidney and ureter in diseases classified elsewhere | Diagnosis | ICD-10-CM |
| **Immune Disorders** | | | |
| C88.0 | Waldenstrom macroglobulinemia | Diagnosis | ICD-10-CM |
| C96.5 | Multifocal and unisystemic Langerhans-cell histiocytosis | Diagnosis | ICD-10-CM |
| C96.6 | Unifocal Langerhans-cell histiocytosis | Diagnosis | ICD-10-CM |
| D47.2 | Monoclonal gammopathy | Diagnosis | ICD-10-CM |
| D80.0 | Hereditary hypogammaglobulinemia | Diagnosis | ICD-10-CM |
| D80.1 | Nonfamilial hypogammaglobulinemia | Diagnosis | ICD-10-CM |
| D80.2 | Selective deficiency of immunoglobulin A [IgA] | Diagnosis | ICD-10-CM |
| D80.3 | Selective deficiency of immunoglobulin G [IgG] subclasses | Diagnosis | ICD-10-CM |
| D80.4 | Selective deficiency of immunoglobulin M [IgM] | Diagnosis | ICD-10-CM |
| D80.5 | Immunodeficiency with increased immunoglobulin M [IgM] | Diagnosis | ICD-10-CM |
| D80.6 | Antibody deficiency with near-normal immunoglobulins or with hyperimmunoglobulinemia | Diagnosis | ICD-10-CM |
| D80.7 | Transient hypogammaglobulinemia of infancy | Diagnosis | ICD-10-CM |
| D80.8 | Other immunodeficiencies with predominantly antibody defects | Diagnosis | ICD-10-CM |
| D80.9 | Immunodeficiency with predominantly antibody defects, unspecified | Diagnosis | ICD-10-CM |
| D81.0 | Severe combined immunodeficiency [SCID] with reticular dysgenesis | Diagnosis | ICD-10-CM |
| D81.1 | Severe combined immunodeficiency [SCID] with low T- and B-cell numbers | Diagnosis | ICD-10-CM |
| D81.2 | Severe combined immunodeficiency [SCID] with low or normal B-cell numbers | Diagnosis | ICD-10-CM |
| D81.3 | Adenosine deaminase [ADA] deficiency | Diagnosis | ICD-10-CM |
| D81.4 | Nezelof's syndrome | Diagnosis | ICD-10-CM |
| D81.5 | Purine nucleoside phosphorylase [PNP] deficiency | Diagnosis | ICD-10-CM |
| D81.6 | Major histocompatibility complex class I deficiency | Diagnosis | ICD-10-CM |
| D81.7 | Major histocompatibility complex class II deficiency | Diagnosis | ICD-10-CM |
| D81.810 | Biotinidase deficiency | Diagnosis | ICD-10-CM |
| D81.818 | Other biotin-dependent carboxylase deficiency | Diagnosis | ICD-10-CM |
| D81.819 | Biotin-dependent carboxylase deficiency, unspecified | Diagnosis | ICD-10-CM |
| D81.89 | Other combined immunodeficiencies | Diagnosis | ICD-10-CM |
| D81.9 | Combined immunodeficiency, unspecified | Diagnosis | ICD-10-CM |
| D82.0 | Wiskott-Aldrich syndrome | Diagnosis | ICD-10-CM |
| D82.1 | Di George's syndrome | Diagnosis | ICD-10-CM |
| D82.2 | Immunodeficiency with short-limbed stature | Diagnosis | ICD-10-CM |
| D82.3 | Immunodeficiency following hereditary defective response to Epstein-Barr virus | Diagnosis | ICD-10-CM |
| D82.4 | Hyperimmunoglobulin E [IgE] syndrome | Diagnosis | ICD-10-CM |
| D82.8 | Immunodeficiency associated with other specified major defects | Diagnosis | ICD-10-CM |
| D82.9 | Immunodeficiency associated with major defect, unspecified | Diagnosis | ICD-10-CM |
| D83.0 | Common variable immunodeficiency with predominant abnormalities of B-cell numbers and function | Diagnosis | ICD-10-CM |
| D83.1 | Common variable immunodeficiency with predominant immunoregulatory T-cell disorders | Diagnosis | ICD-10-CM |
| D83.2 | Common variable immunodeficiency with autoantibodies to B- or T-cells | Diagnosis | ICD-10-CM |
| D83.8 | Other common variable immunodeficiencies | Diagnosis | ICD-10-CM |
| D83.9 | Common variable immunodeficiency, unspecified | Diagnosis | ICD-10-CM |
| D84.0 | Lymphocyte function antigen-1 [LFA-1] defect | Diagnosis | ICD-10-CM |
| D84.1 | Defects in the complement system | Diagnosis | ICD-10-CM |
| D84.8 | Other specified immunodeficiencies | Diagnosis | ICD-10-CM |
| D84.9 | Immunodeficiency, unspecified | Diagnosis | ICD-10-CM |
| D89.0 | Polyclonal hypergammaglobulinemia | Diagnosis | ICD-10-CM |
| D89.1 | Cryoglobulinemia | Diagnosis | ICD-10-CM |
| D89.2 | Hypergammaglobulinemia, unspecified | Diagnosis | ICD-10-CM |
| D89.3 | Immune reconstitution syndrome | Diagnosis | ICD-10-CM |
| D89.40 | Mast cell activation, unspecified | Diagnosis | ICD-10-CM |
| D89.41 | Monoclonal mast cell activation syndrome | Diagnosis | ICD-10-CM |
| D89.42 | Idiopathic mast cell activation syndrome | Diagnosis | ICD-10-CM |
| D89.43 | Secondary mast cell activation | Diagnosis | ICD-10-CM |
| D89.49 | Other mast cell activation disorder | Diagnosis | ICD-10-CM |
| D89.810 | Acute graft-versus-host disease | Diagnosis | ICD-10-CM |
| D89.811 | Chronic graft-versus-host disease | Diagnosis | ICD-10-CM |
| D89.812 | Acute on chronic graft-versus-host disease | Diagnosis | ICD-10-CM |
| D89.813 | Graft-versus-host disease, unspecified | Diagnosis | ICD-10-CM |
| D89.82 | Autoimmune lymphoproliferative syndrome [ALPS] | Diagnosis | ICD-10-CM |
| D89.89 | Other specified disorders involving the immune mechanism, not elsewhere classified | Diagnosis | ICD-10-CM |
| D89.9 | Disorder involving the immune mechanism, unspecified | Diagnosis | ICD-10-CM |
| D70.1 | Agranulocytosis secondary to cancer chemotherapy | Diagnosis | ICD-10-CM |
| D70.2 | Other drug-induced agranulocytosis | Diagnosis | ICD-10-CM |
| D70.3 | Neutropenia due to infection | Diagnosis | ICD-10-CM |
| D70.4 | Cyclic neutropenia | Diagnosis | ICD-10-CM |
| D70.8 | Other neutropenia | Diagnosis | ICD-10-CM |
| D70.9 | Neutropenia, unspecified | Diagnosis | ICD-10-CM |
| D72.819 | Decreased white blood cell count, unspecified | Diagnosis | ICD-10-CM |
| T86.91 | Unspecified transplanted organ and tissue rejection | Diagnosis | ICD-10-CM |
| Z94 | Transplanted organ and tissue status | Diagnosis | ICD-10-CM |
| Z94.0 | Kidney transplant status | Diagnosis | ICD-10-CM |
| Z94.1 | Heart transplant status | Diagnosis | ICD-10-CM |
| Z94.2 | Lung transplant status | Diagnosis | ICD-10-CM |
| Z94.3 | Heart and lungs transplant status | Diagnosis | ICD-10-CM |
| Z94.4 | Liver transplant status | Diagnosis | ICD-10-CM |
| Z94.5 | Skin transplant status | Diagnosis | ICD-10-CM |
| Z94.6 | Bone transplant status | Diagnosis | ICD-10-CM |
| Z94.7 | Corneal transplant status | Diagnosis | ICD-10-CM |
| Z94.8 | Other transplanted organ and tissue status | Diagnosis | ICD-10-CM |
| Z94.81 | Bone marrow transplant status | Diagnosis | ICD-10-CM |
| Z94.82 | Intestine transplant status | Diagnosis | ICD-10-CM |
| Z94.83 | Pancreas transplant status | Diagnosis | ICD-10-CM |
| Z94.84 | Stem cells transplant status | Diagnosis | ICD-10-CM |
| Z94.89 | Other transplanted organ and tissue status | Diagnosis | ICD-10-CM |
| Z94.9 | Transplanted organ and tissue status, unspecified | Diagnosis | ICD-10-CM |
| B20 | Human immunodeficiency virus [HIV] disease | Diagnosis | ICD-10-CM |
| B97.35 | Human immunodeficiency virus, type 2 [HIV 2] as the cause of diseases classified elsewhere | Diagnosis | ICD-10-CM |
| Z21 | Asymptomatic human immunodeficiency virus [HIV] infection status | Diagnosis | ICD-10-CM |
| **Other Chronic Respiratory Disorders** | | | |
| E84.0 | Cystic fibrosis with pulmonary manifestations | Diagnosis | ICD-10-CM |
| E84.11 | Meconium ileus in cystic fibrosis | Diagnosis | ICD-10-CM |
| E84.19 | Cystic fibrosis with other intestinal manifestations | Diagnosis | ICD-10-CM |
| E84.8 | Cystic fibrosis with other manifestations | Diagnosis | ICD-10-CM |
| E84.9 | Cystic fibrosis, unspecified | Diagnosis | ICD-10-CM |
| A15.0 | Tuberculosis of lung | Diagnosis | ICD-10-CM |
| A15.5 | Tuberculosis of larynx, trachea and bronchus | Diagnosis | ICD-10-CM |
| A15.6 | Tuberculous pleurisy | Diagnosis | ICD-10-CM |
| A15.7 | Primary respiratory tuberculosis | Diagnosis | ICD-10-CM |
| A15.8 | Other respiratory tuberculosis | Diagnosis | ICD-10-CM |
| A15.9 | Respiratory tuberculosis unspecified | Diagnosis | ICD-10-CM |
| Z86.11 | personal history of tuberculosis | Diagnosis | ICD-10-CM |
| J65 | Pneumoconiosis associated with tuberculosis | Diagnosis | ICD-10-CM |
| D86.0 | Sarcoidosis of lung | Diagnosis | ICD-10-CM |
| D86.1 | Sarcoidosis of lymph nodes | Diagnosis | ICD-10-CM |
| D86.2 | Sarcoidosis of lung with sarcoidosis of lymph nodes | Diagnosis | ICD-10-CM |
| D86.3 | Sarcoidosis of skin | Diagnosis | ICD-10-CM |
| D86.81 | Sarcoid meningitis | Diagnosis | ICD-10-CM |
| D86.82 | Multiple cranial nerve palsies in sarcoidosis | Diagnosis | ICD-10-CM |
| D86.83 | Sarcoid iridocyclitis | Diagnosis | ICD-10-CM |
| D86.84 | Sarcoid pyelonephritis | Diagnosis | ICD-10-CM |
| D86.85 | Sarcoid myocarditis | Diagnosis | ICD-10-CM |
| D86.86 | Sarcoid arthropathy | Diagnosis | ICD-10-CM |
| D86.87 | Sarcoid myositis | Diagnosis | ICD-10-CM |
| D86.89 | Sarcoidosis of other sites | Diagnosis | ICD-10-CM |
| D86.9 | Sarcoidosis, unspecified | Diagnosis | ICD-10-CM |
| G02 | Meningitis in other infectious and parasitic diseases classified elsewhere | Diagnosis | ICD-10-CM |
| **Metabolic Disorders** | | | |
| E01.8 | Other iodine-deficiency related thyroid disorders and allied conditions | Diagnosis | ICD-10-CM |
| E02 | Subclinical iodine-deficiency hypothyroidism | Diagnosis | ICD-10-CM |
| E03.0 | Congenital hypothyroidism with diffuse goiter | Diagnosis | ICD-10-CM |
| E03.1 | Congenital hypothyroidism without goiter | Diagnosis | ICD-10-CM |
| E03.2 | Hypothyroidism due to medicaments and other exogenous substances | Diagnosis | ICD-10-CM |
| E03.3 | Postinfectious hypothyroidism | Diagnosis | ICD-10-CM |
| E03.4 | Atrophy of thyroid (acquired) | Diagnosis | ICD-10-CM |
| E03.8 | Other specified hypothyroidism | Diagnosis | ICD-10-CM |
| E03.9 | Hypothyroidism, unspecified | Diagnosis | ICD-10-CM |
| E04.1 | Nontoxic single thyroid nodule | Diagnosis | ICD-10-CM |
| E04.2 | Nontoxic multinodular goiter | Diagnosis | ICD-10-CM |
| E04.8 | Other specified nontoxic goiter | Diagnosis | ICD-10-CM |
| E04.9 | Nontoxic goiter, unspecified | Diagnosis | ICD-10-CM |
| E05.00 | Thyrotoxicosis with diffuse goiter without thyrotoxic crisis or storm | Diagnosis | ICD-10-CM |
| E05.01 | Thyrotoxicosis with diffuse goiter with thyrotoxic crisis or storm | Diagnosis | ICD-10-CM |
| E05.10 | Thyrotoxicosis with toxic single thyroid nodule without thyrotoxic crisis or storm | Diagnosis | ICD-10-CM |
| E05.11 | Thyrotoxicosis with toxic single thyroid nodule with thyrotoxic crisis or storm | Diagnosis | ICD-10-CM |
| E05.20 | Thyrotoxicosis with toxic multinodular goiter without thyrotoxic crisis or storm | Diagnosis | ICD-10-CM |
| E05.21 | Thyrotoxicosis with toxic multinodular goiter with thyrotoxic crisis or storm | Diagnosis | ICD-10-CM |
| E05.30 | Thyrotoxicosis from ectopic thyroid tissue without thyrotoxic crisis or storm | Diagnosis | ICD-10-CM |
| E05.31 | Thyrotoxicosis from ectopic thyroid tissue with thyrotoxic crisis or storm | Diagnosis | ICD-10-CM |
| E05.40 | Thyrotoxicosis factitia without thyrotoxic crisis or storm | Diagnosis | ICD-10-CM |
| E05.41 | Thyrotoxicosis factitia with thyrotoxic crisis or storm | Diagnosis | ICD-10-CM |
| E05.80 | Other thyrotoxicosis without thyrotoxic crisis or storm | Diagnosis | ICD-10-CM |
| E05.81 | Other thyrotoxicosis with thyrotoxic crisis or storm | Diagnosis | ICD-10-CM |
| E05.90 | Thyrotoxicosis, unspecified without thyrotoxic crisis or storm | Diagnosis | ICD-10-CM |
| E05.91 | Thyrotoxicosis, unspecified with thyrotoxic crisis or storm | Diagnosis | ICD-10-CM |
| E06.0 | Acute thyroiditis | Diagnosis | ICD-10-CM |
| E06.1 | Subacute thyroiditis | Diagnosis | ICD-10-CM |
| E06.2 | Chronic thyroiditis with transient thyrotoxicosis | Diagnosis | ICD-10-CM |
| E06.3 | Autoimmune thyroiditis | Diagnosis | ICD-10-CM |
| E06.4 | Drug-induced thyroiditis | Diagnosis | ICD-10-CM |
| E06.5 | Other chronic thyroiditis | Diagnosis | ICD-10-CM |
| E06.9 | Thyroiditis, unspecified | Diagnosis | ICD-10-CM |
| E07.0 | Hypersecretion of calcitonin | Diagnosis | ICD-10-CM |
| E07.1 | Dyshormogenetic goiter | Diagnosis | ICD-10-CM |
| E07.89 | Other specified disorders of thyroid | Diagnosis | ICD-10-CM |
| E07.9 | Disorder of thyroid, unspecified | Diagnosis | ICD-10-CM |
| E15 | Nondiabetic hypoglycemic coma | Diagnosis | ICD-10-CM |
| E16.0 | Drug-induced hypoglycemia without coma | Diagnosis | ICD-10-CM |
| E16.1 | Other hypoglycemia | Diagnosis | ICD-10-CM |
| E16.2 | Hypoglycemia, unspecified | Diagnosis | ICD-10-CM |
| E16.3 | Increased secretion of glucagon | Diagnosis | ICD-10-CM |
| E16.4 | Increased secretion of gastrin | Diagnosis | ICD-10-CM |
| E16.8 | Other specified disorders of pancreatic internal secretion | Diagnosis | ICD-10-CM |
| E16.9 | Disorder of pancreatic internal secretion, unspecified | Diagnosis | ICD-10-CM |
| E20.0 | Idiopathic hypoparathyroidism | Diagnosis | ICD-10-CM |
| E20.1 | Pseudohypoparathyroidism | Diagnosis | ICD-10-CM |
| E20.8 | Other hypoparathyroidism | Diagnosis | ICD-10-CM |
| E20.9 | Hypoparathyroidism, unspecified | Diagnosis | ICD-10-CM |
| E21.0 | Primary hyperparathyroidism | Diagnosis | ICD-10-CM |
| E21.1 | Secondary hyperparathyroidism, not elsewhere classified | Diagnosis | ICD-10-CM |
| E21.2 | Other hyperparathyroidism | Diagnosis | ICD-10-CM |
| E21.3 | Hyperparathyroidism, unspecified | Diagnosis | ICD-10-CM |
| E21.4 | Other specified disorders of parathyroid gland | Diagnosis | ICD-10-CM |
| E21.5 | Disorder of parathyroid gland, unspecified | Diagnosis | ICD-10-CM |
| E22.0 | Acromegaly and pituitary gigantism | Diagnosis | ICD-10-CM |
| E22.1 | Hyperprolactinemia | Diagnosis | ICD-10-CM |
| E22.2 | Syndrome of inappropriate secretion of antidiuretic hormone | Diagnosis | ICD-10-CM |
| E22.8 | Other hyperfunction of pituitary gland | Diagnosis | ICD-10-CM |
| E22.9 | Hyperfunction of pituitary gland, unspecified | Diagnosis | ICD-10-CM |
| E23.0 | Hypopituitarism | Diagnosis | ICD-10-CM |
| E23.1 | Drug-induced hypopituitarism | Diagnosis | ICD-10-CM |
| E23.2 | Diabetes insipidus | Diagnosis | ICD-10-CM |
| E23.3 | Hypothalamic dysfunction, not elsewhere classified | Diagnosis | ICD-10-CM |
| E23.6 | Other disorders of pituitary gland | Diagnosis | ICD-10-CM |
| E23.7 | Disorder of pituitary gland, unspecified | Diagnosis | ICD-10-CM |
| E24.0 | Pituitary-dependent Cushing's disease | Diagnosis | ICD-10-CM |
| E24.1 | Nelson's syndrome | Diagnosis | ICD-10-CM |
| E24.2 | Drug-induced Cushing's syndrome | Diagnosis | ICD-10-CM |
| E24.3 | Ectopic ACTH syndrome | Diagnosis | ICD-10-CM |
| E24.4 | Alcohol-induced pseudo-Cushing's syndrome | Diagnosis | ICD-10-CM |
| E24.8 | Other Cushing's syndrome | Diagnosis | ICD-10-CM |
| E24.9 | Cushing's syndrome, unspecified | Diagnosis | ICD-10-CM |
| E25.0 | Congenital adrenogenital disorders associated with enzyme deficiency | Diagnosis | ICD-10-CM |
| E25.8 | Other adrenogenital disorders | Diagnosis | ICD-10-CM |
| E25.9 | Adrenogenital disorder, unspecified | Diagnosis | ICD-10-CM |
| E26.01 | Conn's syndrome | Diagnosis | ICD-10-CM |
| E26.02 | Glucocorticoid-remediable aldosteronism | Diagnosis | ICD-10-CM |
| E26.09 | Other primary hyperaldosteronism | Diagnosis | ICD-10-CM |
| E26.1 | Secondary hyperaldosteronism | Diagnosis | ICD-10-CM |
| E26.81 | Bartter's syndrome | Diagnosis | ICD-10-CM |
| E26.89 | Other hyperaldosteronism | Diagnosis | ICD-10-CM |
| E26.9 | Hyperaldosteronism, unspecified | Diagnosis | ICD-10-CM |
| E27.0 | Other adrenocortical overactivity | Diagnosis | ICD-10-CM |
| E27.1 | Primary adrenocortical insufficiency | Diagnosis | ICD-10-CM |
| E27.2 | Addisonian crisis | Diagnosis | ICD-10-CM |
| E27.3 | Drug-induced adrenocortical insufficiency | Diagnosis | ICD-10-CM |
| E27.40 | Unspecified adrenocortical insufficiency | Diagnosis | ICD-10-CM |
| E27.49 | Other adrenocortical insufficiency | Diagnosis | ICD-10-CM |
| E27.5 | Adrenomedullary hyperfunction | Diagnosis | ICD-10-CM |
| E27.8 | Other specified disorders of adrenal gland | Diagnosis | ICD-10-CM |
| E27.9 | Disorder of adrenal gland, unspecified | Diagnosis | ICD-10-CM |
| E28.0 | Estrogen excess | Diagnosis | ICD-10-CM |
| E28.1 | Androgen excess | Diagnosis | ICD-10-CM |
| E28.2 | Polycystic ovarian syndrome | Diagnosis | ICD-10-CM |
| E28.310 | Symptomatic premature menopause | Diagnosis | ICD-10-CM |
| E28.319 | Asymptomatic premature menopause | Diagnosis | ICD-10-CM |
| E28.39 | Other primary ovarian failure | Diagnosis | ICD-10-CM |
| E28.8 | Other ovarian dysfunction | Diagnosis | ICD-10-CM |
| E28.9 | Ovarian dysfunction, unspecified | Diagnosis | ICD-10-CM |
| E29.0 | Testicular hyperfunction | Diagnosis | ICD-10-CM |
| E29.1 | Testicular hypofunction | Diagnosis | ICD-10-CM |
| E29.8 | Other testicular dysfunction | Diagnosis | ICD-10-CM |
| E29.9 | Testicular dysfunction, unspecified | Diagnosis | ICD-10-CM |
| E30.0 | Delayed puberty | Diagnosis | ICD-10-CM |
| E30.1 | Precocious puberty | Diagnosis | ICD-10-CM |
| E31.0 | Autoimmune polyglandular failure | Diagnosis | ICD-10-CM |
| E31.1 | Polyglandular hyperfunction | Diagnosis | ICD-10-CM |
| E31.20 | Multiple endocrine neoplasia [MEN] syndrome, unspecified | Diagnosis | ICD-10-CM |
| E31.21 | Multiple endocrine neoplasia [MEN] type I | Diagnosis | ICD-10-CM |
| E31.22 | Multiple endocrine neoplasia [MEN] type IIA | Diagnosis | ICD-10-CM |
| E31.23 | Multiple endocrine neoplasia [MEN] type IIB | Diagnosis | ICD-10-CM |
| E31.8 | Other polyglandular dysfunction | Diagnosis | ICD-10-CM |
| E31.9 | Polyglandular dysfunction, unspecified | Diagnosis | ICD-10-CM |
| E32.0 | Persistent hyperplasia of thymus | Diagnosis | ICD-10-CM |
| E32.1 | Abscess of thymus | Diagnosis | ICD-10-CM |
| E32.8 | Other diseases of thymus | Diagnosis | ICD-10-CM |
| E32.9 | Disease of thymus, unspecified | Diagnosis | ICD-10-CM |
| E34.0 | Carcinoid syndrome | Diagnosis | ICD-10-CM |
| E34.1 | Other hypersecretion of intestinal hormones | Diagnosis | ICD-10-CM |
| E34.2 | Ectopic hormone secretion, not elsewhere classified | Diagnosis | ICD-10-CM |
| E34.50 | Androgen insensitivity syndrome, unspecified | Diagnosis | ICD-10-CM |
| E34.51 | Complete androgen insensitivity syndrome | Diagnosis | ICD-10-CM |
| E34.52 | Partial androgen insensitivity syndrome | Diagnosis | ICD-10-CM |
| E34.8 | Other specified endocrine disorders | Diagnosis | ICD-10-CM |
| E34.9 | Endocrine disorder, unspecified | Diagnosis | ICD-10-CM |
| E35 | Disorders of endocrine glands in diseases classified elsewhere | Diagnosis | ICD-10-CM |
| E70.0 | Classical phenylketonuria | Diagnosis | ICD-10-CM |
| E70.1 | Other hyperphenylalaninemias | Diagnosis | ICD-10-CM |
| E70.20 | Disorder of tyrosine metabolism, unspecified | Diagnosis | ICD-10-CM |
| E70.21 | Tyrosinemia | Diagnosis | ICD-10-CM |
| E70.29 | Other disorders of tyrosine metabolism | Diagnosis | ICD-10-CM |
| E70.30 | Albinism, unspecified | Diagnosis | ICD-10-CM |
| E70.310 | X-linked ocular albinism | Diagnosis | ICD-10-CM |
| E70.311 | Autosomal recessive ocular albinism | Diagnosis | ICD-10-CM |
| E70.318 | Other ocular albinism | Diagnosis | ICD-10-CM |
| E70.319 | Ocular albinism, unspecified | Diagnosis | ICD-10-CM |
| E70.320 | Tyrosinase negative oculocutaneous albinism | Diagnosis | ICD-10-CM |
| E70.321 | Tyrosinase positive oculocutaneous albinism | Diagnosis | ICD-10-CM |
| E70.328 | Other oculocutaneous albinism | Diagnosis | ICD-10-CM |
| E70.329 | Oculocutaneous albinism, unspecified | Diagnosis | ICD-10-CM |
| E70.330 | Chediak-Higashi syndrome | Diagnosis | ICD-10-CM |
| E70.331 | Hermansky-Pudlak syndrome | Diagnosis | ICD-10-CM |
| E70.338 | Other albinism with hematologic abnormality | Diagnosis | ICD-10-CM |
| E70.339 | Albinism with hematologic abnormality, unspecified | Diagnosis | ICD-10-CM |
| E70.39 | Other specified albinism | Diagnosis | ICD-10-CM |
| E70.40 | Disorders of histidine metabolism, unspecified | Diagnosis | ICD-10-CM |
| E70.41 | Histidinemia | Diagnosis | ICD-10-CM |
| E70.49 | Other disorders of histidine metabolism | Diagnosis | ICD-10-CM |
| E70.5 | Disorders of tryptophan metabolism | Diagnosis | ICD-10-CM |
| E70.8 | Other disorders of aromatic amino-acid metabolism | Diagnosis | ICD-10-CM |
| E70.9 | Disorder of aromatic amino-acid metabolism, unspecified | Diagnosis | ICD-10-CM |
| E71.0 | Maple-syrup-urine disease | Diagnosis | ICD-10-CM |
| E71.110 | Isovaleric acidemia | Diagnosis | ICD-10-CM |
| E71.111 | 3-methylglutaconic aciduria | Diagnosis | ICD-10-CM |
| E71.118 | Other branched-chain organic acidurias | Diagnosis | ICD-10-CM |
| E71.120 | Methylmalonic acidemia | Diagnosis | ICD-10-CM |
| E71.121 | Propionic acidemia | Diagnosis | ICD-10-CM |
| E71.128 | Other disorders of propionate metabolism | Diagnosis | ICD-10-CM |
| E71.19 | Other disorders of branched-chain amino-acid metabolism | Diagnosis | ICD-10-CM |
| E71.2 | Disorder of branched-chain amino-acid metabolism, unspecified | Diagnosis | ICD-10-CM |
| E71.30 | Disorder of fatty-acid metabolism, unspecified | Diagnosis | ICD-10-CM |
| E71.310 | Long chain/very long chain acyl CoA dehydrogenase deficiency | Diagnosis | ICD-10-CM |
| E71.311 | Medium chain acyl CoA dehydrogenase deficiency | Diagnosis | ICD-10-CM |
| E71.312 | Short chain acyl CoA dehydrogenase deficiency | Diagnosis | ICD-10-CM |
| E71.313 | Glutaric aciduria type II | Diagnosis | ICD-10-CM |
| E71.314 | Muscle carnitine palmitoyltransferase deficiency | Diagnosis | ICD-10-CM |
| E71.318 | Other disorders of fatty-acid oxidation | Diagnosis | ICD-10-CM |
| E71.32 | Disorders of ketone metabolism | Diagnosis | ICD-10-CM |
| E71.39 | Other disorders of fatty-acid metabolism | Diagnosis | ICD-10-CM |
| E71.40 | Disorder of carnitine metabolism, unspecified | Diagnosis | ICD-10-CM |
| E71.41 | Primary carnitine deficiency | Diagnosis | ICD-10-CM |
| E71.42 | Carnitine deficiency due to inborn errors of metabolism | Diagnosis | ICD-10-CM |
| E71.43 | Iatrogenic carnitine deficiency | Diagnosis | ICD-10-CM |
| E71.440 | Ruvalcaba-Myhre-Smith syndrome | Diagnosis | ICD-10-CM |
| E71.448 | Other secondary carnitine deficiency | Diagnosis | ICD-10-CM |
| E71.50 | Peroxisomal disorder, unspecified | Diagnosis | ICD-10-CM |
| E71.510 | Zellweger syndrome | Diagnosis | ICD-10-CM |
| E71.511 | Neonatal adrenoleukodystrophy | Diagnosis | ICD-10-CM |
| E71.518 | Other disorders of peroxisome biogenesis | Diagnosis | ICD-10-CM |
| E71.520 | Childhood cerebral X-linked adrenoleukodystrophy | Diagnosis | ICD-10-CM |
| E71.521 | Adolescent X-linked adrenoleukodystrophy | Diagnosis | ICD-10-CM |
| E71.522 | Adrenomyeloneuropathy | Diagnosis | ICD-10-CM |
| E71.528 | Other X-linked adrenoleukodystrophy | Diagnosis | ICD-10-CM |
| E71.529 | X-linked adrenoleukodystrophy, unspecified type | Diagnosis | ICD-10-CM |
| E71.53 | Other group 2 peroxisomal disorders | Diagnosis | ICD-10-CM |
| E71.540 | Rhizomelic chondrodysplasia punctata | Diagnosis | ICD-10-CM |
| E71.541 | Zellweger-like syndrome | Diagnosis | ICD-10-CM |
| E71.542 | Other group 3 peroxisomal disorders | Diagnosis | ICD-10-CM |
| E71.548 | Other peroxisomal disorders | Diagnosis | ICD-10-CM |
| E72.00 | Disorders of amino-acid transport, unspecified | Diagnosis | ICD-10-CM |
| E72.01 | Cystinuria | Diagnosis | ICD-10-CM |
| E72.02 | Hartnup's disease | Diagnosis | ICD-10-CM |
| E72.03 | Lowe's syndrome | Diagnosis | ICD-10-CM |
| E72.04 | Cystinosis | Diagnosis | ICD-10-CM |
| E72.09 | Other disorders of amino-acid transport | Diagnosis | ICD-10-CM |
| E72.10 | Disorders of sulfur-bearing amino-acid metabolism, unspecified | Diagnosis | ICD-10-CM |
| E72.11 | Homocystinuria | Diagnosis | ICD-10-CM |
| E72.12 | Methylenetetrahydrofolate reductase deficiency | Diagnosis | ICD-10-CM |
| E72.19 | Other disorders of sulfur-bearing amino-acid metabolism | Diagnosis | ICD-10-CM |
| E72.20 | Disorder of urea cycle metabolism, unspecified | Diagnosis | ICD-10-CM |
| E72.21 | Argininemia | Diagnosis | ICD-10-CM |
| E72.22 | Arginosuccinic aciduria | Diagnosis | ICD-10-CM |
| E72.23 | Citrullinemia | Diagnosis | ICD-10-CM |
| E72.29 | Other disorders of urea cycle metabolism | Diagnosis | ICD-10-CM |
| E72.3 | Disorders of lysine and hydroxylysine metabolism | Diagnosis | ICD-10-CM |
| E72.4 | Disorders of ornithine metabolism | Diagnosis | ICD-10-CM |
| E72.50 | Disorder of glycine metabolism, unspecified | Diagnosis | ICD-10-CM |
| E72.51 | Non-ketotic hyperglycinemia | Diagnosis | ICD-10-CM |
| E72.52 | Trimethylaminuria | Diagnosis | ICD-10-CM |
| E72.53 | Primary hyperoxaluria | Diagnosis | ICD-10-CM |
| E72.59 | Other disorders of glycine metabolism | Diagnosis | ICD-10-CM |
| E72.8 | Other specified disorders of amino-acid metabolism | Diagnosis | ICD-10-CM |
| E72.9 | Disorder of amino-acid metabolism, unspecified | Diagnosis | ICD-10-CM |
| E73.0 | Congenital lactase deficiency | Diagnosis | ICD-10-CM |
| E73.1 | Secondary lactase deficiency | Diagnosis | ICD-10-CM |
| E73.8 | Other lactose intolerance | Diagnosis | ICD-10-CM |
| E73.9 | Lactose intolerance, unspecified | Diagnosis | ICD-10-CM |
| E74.00 | Glycogen storage disease, unspecified | Diagnosis | ICD-10-CM |
| E74.01 | von Gierke disease | Diagnosis | ICD-10-CM |
| E74.02 | Pompe disease | Diagnosis | ICD-10-CM |
| E74.03 | Cori disease | Diagnosis | ICD-10-CM |
| E74.04 | McArdle disease | Diagnosis | ICD-10-CM |
| E74.09 | Other glycogen storage disease | Diagnosis | ICD-10-CM |
| E74.10 | Disorder of fructose metabolism, unspecified | Diagnosis | ICD-10-CM |
| E74.11 | Essential fructosuria | Diagnosis | ICD-10-CM |
| E74.12 | Hereditary fructose intolerance | Diagnosis | ICD-10-CM |
| E74.19 | Other disorders of fructose metabolism | Diagnosis | ICD-10-CM |
| E74.20 | Disorders of galactose metabolism, unspecified | Diagnosis | ICD-10-CM |
| E74.21 | Galactosemia | Diagnosis | ICD-10-CM |
| E74.29 | Other disorders of galactose metabolism | Diagnosis | ICD-10-CM |
| E74.31 | Sucrase-isomaltase deficiency | Diagnosis | ICD-10-CM |
| E74.39 | Other disorders of intestinal carbohydrate absorption | Diagnosis | ICD-10-CM |
| E74.4 | Disorders of pyruvate metabolism and gluconeogenesis | Diagnosis | ICD-10-CM |
| E74.8 | Other specified disorders of carbohydrate metabolism | Diagnosis | ICD-10-CM |
| E74.9 | Disorder of carbohydrate metabolism, unspecified | Diagnosis | ICD-10-CM |
| E75.21 | Fabry (-Anderson) disease | Diagnosis | ICD-10-CM |
| E75.22 | Gaucher disease | Diagnosis | ICD-10-CM |
| E75.240 | Niemann-Pick disease type A | Diagnosis | ICD-10-CM |
| E75.241 | Niemann-Pick disease type B | Diagnosis | ICD-10-CM |
| E75.242 | Niemann-Pick disease type C | Diagnosis | ICD-10-CM |
| E75.243 | Niemann-Pick disease type D | Diagnosis | ICD-10-CM |
| E75.248 | Other Niemann-Pick disease | Diagnosis | ICD-10-CM |
| E75.249 | Niemann-Pick disease, unspecified | Diagnosis | ICD-10-CM |
| E75.3 | Sphingolipidosis, unspecified | Diagnosis | ICD-10-CM |
| E75.5 | Other lipid storage disorders | Diagnosis | ICD-10-CM |
| E75.6 | Lipid storage disorder, unspecified | Diagnosis | ICD-10-CM |
| E76.01 | Hurler's syndrome | Diagnosis | ICD-10-CM |
| E76.02 | Hurler-Scheie syndrome | Diagnosis | ICD-10-CM |
| E76.03 | Scheie's syndrome | Diagnosis | ICD-10-CM |
| E76.1 | Mucopolysaccharidosis, type II | Diagnosis | ICD-10-CM |
| E76.210 | Morquio A mucopolysaccharidoses | Diagnosis | ICD-10-CM |
| E76.211 | Morquio B mucopolysaccharidoses | Diagnosis | ICD-10-CM |
| E76.219 | Morquio mucopolysaccharidoses, unspecified | Diagnosis | ICD-10-CM |
| E76.22 | Sanfilippo mucopolysaccharidoses | Diagnosis | ICD-10-CM |
| E76.29 | Other mucopolysaccharidoses | Diagnosis | ICD-10-CM |
| E76.3 | Mucopolysaccharidosis, unspecified | Diagnosis | ICD-10-CM |
| E76.8 | Other disorders of glucosaminoglycan metabolism | Diagnosis | ICD-10-CM |
| E76.9 | Glucosaminoglycan metabolism disorder, unspecified | Diagnosis | ICD-10-CM |
| E77.0 | Defects in post-translational modification of lysosomal enzymes | Diagnosis | ICD-10-CM |
| E77.1 | Defects in glycoprotein degradation | Diagnosis | ICD-10-CM |
| E77.8 | Other disorders of glycoprotein metabolism | Diagnosis | ICD-10-CM |
| E77.9 | Disorder of glycoprotein metabolism, unspecified | Diagnosis | ICD-10-CM |
| E78.00 | Pure hypercholesterolemia, unspecified | Diagnosis | ICD-10-CM |
| E78.01 | Familial hypercholesterolemia | Diagnosis | ICD-10-CM |
| E78.1 | Pure hyperglyceridemia | Diagnosis | ICD-10-CM |
| E78.2 | Mixed hyperlipidemia | Diagnosis | ICD-10-CM |
| E78.3 | Hyperchylomicronemia | Diagnosis | ICD-10-CM |
| E78.4 | Other hyperlipidemia | Diagnosis | ICD-10-CM |
| E78.5 | Hyperlipidemia, unspecified | Diagnosis | ICD-10-CM |
| E78.6 | Lipoprotein deficiency | Diagnosis | ICD-10-CM |
| E78.70 | Disorder of bile acid and cholesterol metabolism, unspecified | Diagnosis | ICD-10-CM |
| E78.79 | Other disorders of bile acid and cholesterol metabolism | Diagnosis | ICD-10-CM |
| E78.81 | Lipoid dermatoarthritis | Diagnosis | ICD-10-CM |
| E78.89 | Other lipoprotein metabolism disorders | Diagnosis | ICD-10-CM |
| E78.9 | Disorder of lipoprotein metabolism, unspecified | Diagnosis | ICD-10-CM |
| E79.1 | Lesch-Nyhan syndrome | Diagnosis | ICD-10-CM |
| E79.2 | Myoadenylate deaminase deficiency | Diagnosis | ICD-10-CM |
| E79.8 | Other disorders of purine and pyrimidine metabolism | Diagnosis | ICD-10-CM |
| E79.9 | Disorder of purine and pyrimidine metabolism, unspecified | Diagnosis | ICD-10-CM |
| E80.0 | Hereditary erythropoietic porphyria | Diagnosis | ICD-10-CM |
| E80.1 | Porphyria cutanea tarda | Diagnosis | ICD-10-CM |
| E80.20 | Unspecified porphyria | Diagnosis | ICD-10-CM |
| E80.21 | Acute intermittent (hepatic) porphyria | Diagnosis | ICD-10-CM |
| E80.29 | Other porphyria | Diagnosis | ICD-10-CM |
| E80.3 | Defects of catalase and peroxidase | Diagnosis | ICD-10-CM |
| E80.4 | Gilbert syndrome | Diagnosis | ICD-10-CM |
| E80.5 | Crigler-Najjar syndrome | Diagnosis | ICD-10-CM |
| E80.6 | Other disorders of bilirubin metabolism | Diagnosis | ICD-10-CM |
| E80.7 | Disorder of bilirubin metabolism, unspecified | Diagnosis | ICD-10-CM |
| E83.00 | Disorder of copper metabolism, unspecified | Diagnosis | ICD-10-CM |
| E83.01 | Wilson's disease | Diagnosis | ICD-10-CM |
| E83.09 | Other disorders of copper metabolism | Diagnosis | ICD-10-CM |
| E83.10 | Disorder of iron metabolism, unspecified | Diagnosis | ICD-10-CM |
| E83.110 | Hereditary hemochromatosis | Diagnosis | ICD-10-CM |
| E83.111 | Hemochromatosis due to repeated red blood cell transfusions | Diagnosis | ICD-10-CM |
| E83.118 | Other hemochromatosis | Diagnosis | ICD-10-CM |
| E83.119 | Hemochromatosis, unspecified | Diagnosis | ICD-10-CM |
| E83.19 | Other disorders of iron metabolism | Diagnosis | ICD-10-CM |
| E83.30 | Disorder of phosphorus metabolism, unspecified | Diagnosis | ICD-10-CM |
| E83.31 | Familial hypophosphatemia | Diagnosis | ICD-10-CM |
| E83.32 | Hereditary vitamin D-dependent rickets (type 1) (type 2) | Diagnosis | ICD-10-CM |
| E83.39 | Other disorders of phosphorus metabolism | Diagnosis | ICD-10-CM |
| E83.40 | Disorders of magnesium metabolism, unspecified | Diagnosis | ICD-10-CM |
| E83.41 | Hypermagnesemia | Diagnosis | ICD-10-CM |
| E83.42 | Hypomagnesemia | Diagnosis | ICD-10-CM |
| E83.49 | Other disorders of magnesium metabolism | Diagnosis | ICD-10-CM |
| E83.50 | Unspecified disorder of calcium metabolism | Diagnosis | ICD-10-CM |
| E83.51 | Hypocalcemia | Diagnosis | ICD-10-CM |
| E83.52 | Hypercalcemia | Diagnosis | ICD-10-CM |
| E83.59 | Other disorders of calcium metabolism | Diagnosis | ICD-10-CM |
| E83.81 | Hungry bone syndrome | Diagnosis | ICD-10-CM |
| E83.89 | Other disorders of mineral metabolism | Diagnosis | ICD-10-CM |
| E83.9 | Disorder of mineral metabolism, unspecified | Diagnosis | ICD-10-CM |
| E84.0 | Cystic fibrosis with pulmonary manifestations | Diagnosis | ICD-10-CM |
| E84.11 | Meconium ileus in cystic fibrosis | Diagnosis | ICD-10-CM |
| E84.19 | Cystic fibrosis with other intestinal manifestations | Diagnosis | ICD-10-CM |
| E84.8 | Cystic fibrosis with other manifestations | Diagnosis | ICD-10-CM |
| E84.9 | Cystic fibrosis, unspecified | Diagnosis | ICD-10-CM |
| E85.0 | Non-neuropathic heredofamilial amyloidosis | Diagnosis | ICD-10-CM |
| E85.1 | Neuropathic heredofamilial amyloidosis | Diagnosis | ICD-10-CM |
| E85.2 | Heredofamilial amyloidosis, unspecified | Diagnosis | ICD-10-CM |
| E85.3 | Secondary systemic amyloidosis | Diagnosis | ICD-10-CM |
| E85.4 | Organ-limited amyloidosis | Diagnosis | ICD-10-CM |
| E85.81 | Light chain (AL) amyloidosis | Diagnosis | ICD-10-CM |
| E85.82 | Wild-type transthyretin-related (ATTR) amyloidosis | Diagnosis | ICD-10-CM |
| E85.89 | Other amyloidosis | Diagnosis | ICD-10-CM |
| E85.9 | Amyloidosis, unspecified | Diagnosis | ICD-10-CM |
| E86.0 | Dehydration | Diagnosis | ICD-10-CM |
| E86.1 | Hypovolemia | Diagnosis | ICD-10-CM |
| E86.9 | Volume depletion, unspecified | Diagnosis | ICD-10-CM |
| E87.0 | Hyperosmolality and hypernatremia | Diagnosis | ICD-10-CM |
| E87.1 | Hypo-osmolality and hyponatremia | Diagnosis | ICD-10-CM |
| E87.2 | Acidosis | Diagnosis | ICD-10-CM |
| E87.3 | Alkalosis | Diagnosis | ICD-10-CM |
| E87.4 | Mixed disorder of acid-base balance | Diagnosis | ICD-10-CM |
| E87.5 | Hyperkalemia | Diagnosis | ICD-10-CM |
| E87.6 | Hypokalemia | Diagnosis | ICD-10-CM |
| E87.70 | Fluid overload, unspecified | Diagnosis | ICD-10-CM |
| E87.71 | Transfusion associated circulatory overload | Diagnosis | ICD-10-CM |
| E87.79 | Other fluid overload | Diagnosis | ICD-10-CM |
| E87.8 | Other disorders of electrolyte and fluid balance, not elsewhere classified | Diagnosis | ICD-10-CM |
| E88.01 | Alpha-1-antitrypsin deficiency | Diagnosis | ICD-10-CM |
| E88.09 | Other disorders of plasma-protein metabolism, not elsewhere classified | Diagnosis | ICD-10-CM |
| E88.1 | Lipodystrophy, not elsewhere classified | Diagnosis | ICD-10-CM |
| E88.2 | Lipomatosis, not elsewhere classified | Diagnosis | ICD-10-CM |
| E88.3 | Tumor lysis syndrome | Diagnosis | ICD-10-CM |
| E88.40 | Mitochondrial metabolism disorder, unspecified | Diagnosis | ICD-10-CM |
| E88.41 | MELAS syndrome | Diagnosis | ICD-10-CM |
| E88.42 | MERRF syndrome | Diagnosis | ICD-10-CM |
| E88.49 | Other mitochondrial metabolism disorders | Diagnosis | ICD-10-CM |
| E88.81 | Metabolic syndrome | Diagnosis | ICD-10-CM |
| E88.89 | Other specified metabolic disorders | Diagnosis | ICD-10-CM |
| E88.9 | Metabolic disorder, unspecified | Diagnosis | ICD-10-CM |
| E89.0 | Postprocedural hypothyroidism | Diagnosis | ICD-10-CM |
| E89.1 | Postprocedural hypoinsulinemia | Diagnosis | ICD-10-CM |
| E89.2 | Postprocedural hypoparathyroidism | Diagnosis | ICD-10-CM |
| E89.3 | Postprocedural hypopituitarism | Diagnosis | ICD-10-CM |
| E89.40 | Asymptomatic postprocedural ovarian failure | Diagnosis | ICD-10-CM |
| E89.41 | Symptomatic postprocedural ovarian failure | Diagnosis | ICD-10-CM |
| E89.5 | Postprocedural testicular hypofunction | Diagnosis | ICD-10-CM |
| E89.6 | Postprocedural adrenocortical (-medullary) hypofunction | Diagnosis | ICD-10-CM |
| M10.00 | Idiopathic gout, unspecified site | Diagnosis | ICD-10-CM |
| M10.09 | Idiopathic gout, multiple sites | Diagnosis | ICD-10-CM |
| M10.10 | Lead-induced gout, unspecified site | Diagnosis | ICD-10-CM |
| M10.20 | Drug-induced gout, unspecified site | Diagnosis | ICD-10-CM |
| M10.29 | Drug-induced gout, multiple sites | Diagnosis | ICD-10-CM |
| M10.30 | Gout due to renal impairment, unspecified site | Diagnosis | ICD-10-CM |
| M10.39 | Gout due to renal impairment, multiple sites | Diagnosis | ICD-10-CM |
| M10.40 | Other secondary gout, unspecified site | Diagnosis | ICD-10-CM |
| M10.49 | Other secondary gout, multiple sites | Diagnosis | ICD-10-CM |
| M10.9 | Gout, unspecified | Diagnosis | ICD-10-CM |
| M1A.00X0 | Idiopathic chronic gout, unspecified site, without tophus (tophi) | Diagnosis | ICD-10-CM |
| M1A.00X1 | Idiopathic chronic gout, unspecified site, with tophus (tophi) | Diagnosis | ICD-10-CM |
| M1A.09X0 | Idiopathic chronic gout, multiple sites, without tophus (tophi) | Diagnosis | ICD-10-CM |
| M1A.09X1 | Idiopathic chronic gout, multiple sites, with tophus (tophi) | Diagnosis | ICD-10-CM |
| M1A.20X0 | Drug-induced chronic gout, unspecified site, without tophus (tophi) | Diagnosis | ICD-10-CM |
| M1A.20X1 | Drug-induced chronic gout, unspecified site, with tophus (tophi) | Diagnosis | ICD-10-CM |
| M1A.2290 | Drug-induced chronic gout, unspecified elbow, without tophus (tophi) | Diagnosis | ICD-10-CM |
| M1A.2291 | Drug-induced chronic gout, unspecified elbow, with tophus (tophi) | Diagnosis | ICD-10-CM |
| M1A.29X0 | Drug-induced chronic gout, multiple sites, without tophus (tophi) | Diagnosis | ICD-10-CM |
| M1A.29X1 | Drug-induced chronic gout, multiple sites, with tophus (tophi) | Diagnosis | ICD-10-CM |
| M1A.30X0 | Chronic gout due to renal impairment, unspecified site, without tophus (tophi) | Diagnosis | ICD-10-CM |
| M1A.30X1 | Chronic gout due to renal impairment, unspecified site, with tophus (tophi) | Diagnosis | ICD-10-CM |
| M1A.39X0 | Chronic gout due to renal impairment, multiple sites, without tophus (tophi) | Diagnosis | ICD-10-CM |
| M1A.39X1 | Chronic gout due to renal impairment, multiple sites, with tophus (tophi) | Diagnosis | ICD-10-CM |
| M1A.9XX0 | Chronic gout, unspecified, without tophus (tophi) | Diagnosis | ICD-10-CM |
| M1A.9XX1 | Chronic gout, unspecified, with tophus (tophi) | Diagnosis | ICD-10-CM |
| M35.9 | Systemic involvement of connective tissue, unspecified | Diagnosis | ICD-10-CM |
| M83.0 | Puerperal osteomalacia | Diagnosis | ICD-10-CM |
| M83.1 | Senile osteomalacia | Diagnosis | ICD-10-CM |
| M83.2 | Adult osteomalacia due to malabsorption | Diagnosis | ICD-10-CM |
| M83.4 | Aluminum bone disease | Diagnosis | ICD-10-CM |
| M83.5 | Other drug-induced osteomalacia in adults | Diagnosis | ICD-10-CM |
| M83.8 | Other adult osteomalacia | Diagnosis | ICD-10-CM |
| M83.9 | Adult osteomalacia, unspecified | Diagnosis | ICD-10-CM |
| **Hematological Disorders** | | | |
| D47.4 | Osteomyelofibrosis | Diagnosis | ICD-10-CM |
| D50.9 | Iron deficiency anemia, unspecified | Diagnosis | ICD-10-CM |
| D51.0 | Vitamin B12 deficiency anemia due to intrinsic factor deficiency | Diagnosis | ICD-10-CM |
| D51.1 | Vitamin B12 deficiency anemia due to selective vitamin B12 malabsorption with proteinuria | Diagnosis | ICD-10-CM |
| D51.2 | Transcobalamin II deficiency | Diagnosis | ICD-10-CM |
| D51.3 | Other dietary vitamin B12 deficiency anemia | Diagnosis | ICD-10-CM |
| D51.8 | Other vitamin B12 deficiency anemias | Diagnosis | ICD-10-CM |
| D51.9 | Vitamin B12 deficiency anemia, unspecified | Diagnosis | ICD-10-CM |
| D52.0 | Dietary folate deficiency anemia | Diagnosis | ICD-10-CM |
| D52.1 | Drug-induced folate deficiency anemia | Diagnosis | ICD-10-CM |
| D52.8 | Other folate deficiency anemias | Diagnosis | ICD-10-CM |
| D52.9 | Folate deficiency anemia, unspecified | Diagnosis | ICD-10-CM |
| D53.0 | Protein deficiency anemia | Diagnosis | ICD-10-CM |
| D53.1 | Other megaloblastic anemias, not elsewhere classified | Diagnosis | ICD-10-CM |
| D56.0 | Alpha thalassemia | Diagnosis | ICD-10-CM |
| D56.1 | Beta thalassemia | Diagnosis | ICD-10-CM |
| D56.2 | Delta-beta thalassemia | Diagnosis | ICD-10-CM |
| D56.3 | Thalassemia minor | Diagnosis | ICD-10-CM |
| D56.4 | Hereditary persistence of fetal hemoglobin [HPFH] | Diagnosis | ICD-10-CM |
| D56.5 | Hemoglobin E-beta thalassemia | Diagnosis | ICD-10-CM |
| D56.8 | Other thalassemias | Diagnosis | ICD-10-CM |
| D56.9 | Thalassemia, unspecified | Diagnosis | ICD-10-CM |
| D57.00 | Hb-SS disease with crisis, unspecified | Diagnosis | ICD-10-CM |
| D57.01 | Hb-SS disease with acute chest syndrome | Diagnosis | ICD-10-CM |
| D57.02 | Hb-SS disease with splenic sequestration | Diagnosis | ICD-10-CM |
| D57.1 | Sickle-cell disease without crisis | Diagnosis | ICD-10-CM |
| D57.20 | Sickle-cell/Hb-C disease without crisis | Diagnosis | ICD-10-CM |
| D57.211 | Sickle-cell/Hb-C disease with acute chest syndrome | Diagnosis | ICD-10-CM |
| D57.212 | Sickle-cell/Hb-C disease with splenic sequestration | Diagnosis | ICD-10-CM |
| D57.219 | Sickle-cell/Hb-C disease with crisis, unspecified | Diagnosis | ICD-10-CM |
| D57.3 | Sickle-cell trait | Diagnosis | ICD-10-CM |
| D57.40 | Sickle-cell thalassemia without crisis | Diagnosis | ICD-10-CM |
| D57.411 | Sickle-cell thalassemia with acute chest syndrome | Diagnosis | ICD-10-CM |
| D57.412 | Sickle-cell thalassemia with splenic sequestration | Diagnosis | ICD-10-CM |
| D57.419 | Sickle-cell thalassemia with crisis, unspecified | Diagnosis | ICD-10-CM |
| D57.80 | Other sickle-cell disorders without crisis | Diagnosis | ICD-10-CM |
| D57.811 | Other sickle-cell disorders with acute chest syndrome | Diagnosis | ICD-10-CM |
| D57.812 | Other sickle-cell disorders with splenic sequestration | Diagnosis | ICD-10-CM |
| D57.819 | Other sickle-cell disorders with crisis, unspecified | Diagnosis | ICD-10-CM |
| D58.0 | Hereditary spherocytosis | Diagnosis | ICD-10-CM |
| D58.1 | Hereditary elliptocytosis | Diagnosis | ICD-10-CM |
| D58.2 | Other hemoglobinopathies | Diagnosis | ICD-10-CM |
| D58.8 | Other specified hereditary hemolytic anemias | Diagnosis | ICD-10-CM |
| D58.9 | Hereditary hemolytic anemia, unspecified | Diagnosis | ICD-10-CM |
| D59.0 | Drug-induced autoimmune hemolytic anemia | Diagnosis | ICD-10-CM |
| D59.1 | Other autoimmune hemolytic anemias | Diagnosis | ICD-10-CM |
| D59.2 | Drug-induced nonautoimmune hemolytic anemia | Diagnosis | ICD-10-CM |
| D59.3 | Hemolytic-uremic syndrome | Diagnosis | ICD-10-CM |
| D59.4 | Other nonautoimmune hemolytic anemias | Diagnosis | ICD-10-CM |
| D59.5 | Paroxysmal nocturnal hemoglobinuria [Marchiafava-Micheli] | Diagnosis | ICD-10-CM |
| D59.6 | Hemoglobinuria due to hemolysis from other external causes | Diagnosis | ICD-10-CM |
| D59.8 | Other acquired hemolytic anemias | Diagnosis | ICD-10-CM |
| D59.9 | Acquired hemolytic anemia, unspecified | Diagnosis | ICD-10-CM |
| D60.0 | Chronic acquired pure red cell aplasia | Diagnosis | ICD-10-CM |
| D60.1 | Transient acquired pure red cell aplasia | Diagnosis | ICD-10-CM |
| D60.8 | Other acquired pure red cell aplasias | Diagnosis | ICD-10-CM |
| D60.9 | Acquired pure red cell aplasia, unspecified | Diagnosis | ICD-10-CM |
| D61.01 | Constitutional (pure) red blood cell aplasia | Diagnosis | ICD-10-CM |
| D61.09 | Other constitutional aplastic anemia | Diagnosis | ICD-10-CM |
| D61.1 | Drug-induced aplastic anemia | Diagnosis | ICD-10-CM |
| D61.2 | Aplastic anemia due to other external agents | Diagnosis | ICD-10-CM |
| D61.3 | Idiopathic aplastic anemia | Diagnosis | ICD-10-CM |
| D61.810 | Antineoplastic chemotherapy induced pancytopenia | Diagnosis | ICD-10-CM |
| D61.811 | Other drug-induced pancytopenia | Diagnosis | ICD-10-CM |
| D61.818 | Other pancytopenia | Diagnosis | ICD-10-CM |
| D61.82 | Myelophthisis | Diagnosis | ICD-10-CM |
| D61.89 | Other specified aplastic anemias and other bone marrow failure syndromes | Diagnosis | ICD-10-CM |
| D61.9 | Aplastic anemia, unspecified | Diagnosis | ICD-10-CM |
| D62 | Acute posthemorrhagic anemia | Diagnosis | ICD-10-CM |
| D63.0 | Anemia in neoplastic disease | Diagnosis | ICD-10-CM |
| D63.1 | Anemia in chronic kidney disease | Diagnosis | ICD-10-CM |
| D63.8 | Anemia in other chronic diseases classified elsewhere | Diagnosis | ICD-10-CM |
| D64.0 | Hereditary sideroblastic anemia | Diagnosis | ICD-10-CM |
| D64.1 | Secondary sideroblastic anemia due to disease | Diagnosis | ICD-10-CM |
| D64.2 | Secondary sideroblastic anemia due to drugs and toxins | Diagnosis | ICD-10-CM |
| D64.3 | Other sideroblastic anemias | Diagnosis | ICD-10-CM |
| D64.4 | Congenital dyserythropoietic anemia | Diagnosis | ICD-10-CM |
| D64.81 | Anemia due to antineoplastic chemotherapy | Diagnosis | ICD-10-CM |
| D64.89 | Other specified anemias | Diagnosis | ICD-10-CM |
| D64.9 | Anemia, unspecified | Diagnosis | ICD-10-CM |
| D65 | Disseminated intravascular coagulation [defibrination syndrome] | Diagnosis | ICD-10-CM |
| D66 | Hereditary factor VIII deficiency | Diagnosis | ICD-10-CM |
| D67 | Hereditary factor IX deficiency | Diagnosis | ICD-10-CM |
| D68.0 | Von Willebrand's disease | Diagnosis | ICD-10-CM |
| D68.1 | Hereditary factor XI deficiency | Diagnosis | ICD-10-CM |
| D68.2 | Hereditary deficiency of other clotting factors | Diagnosis | ICD-10-CM |
| D68.311 | Acquired hemophilia | Diagnosis | ICD-10-CM |
| D68.312 | Antiphospholipid antibody with hemorrhagic disorder | Diagnosis | ICD-10-CM |
| D68.318 | Other hemorrhagic disorder due to intrinsic circulating anticoagulants, antibodies, or inhibitors | Diagnosis | ICD-10-CM |
| D68.32 | Hemorrhagic disorder due to extrinsic circulating anticoagulants | Diagnosis | ICD-10-CM |
| D68.4 | Acquired coagulation factor deficiency | Diagnosis | ICD-10-CM |
| D68.51 | Activated protein C resistance | Diagnosis | ICD-10-CM |
| D68.52 | Prothrombin gene mutation | Diagnosis | ICD-10-CM |
| D68.59 | Other primary thrombophilia | Diagnosis | ICD-10-CM |
| D68.61 | Antiphospholipid syndrome | Diagnosis | ICD-10-CM |
| D68.62 | Lupus anticoagulant syndrome | Diagnosis | ICD-10-CM |
| D68.69 | Other thrombophilia | Diagnosis | ICD-10-CM |
| D68.8 | Other specified coagulation defects | Diagnosis | ICD-10-CM |
| D68.9 | Coagulation defect, unspecified | Diagnosis | ICD-10-CM |
| D69.2 | Other nonthrombocytopenic purpura | Diagnosis | ICD-10-CM |
| D69.3 | Immune thrombocytopenic purpura | Diagnosis | ICD-10-CM |
| D69.41 | Evans syndrome | Diagnosis | ICD-10-CM |
| D69.42 | Congenital and hereditary thrombocytopenia purpura | Diagnosis | ICD-10-CM |
| D69.49 | Other primary thrombocytopenia | Diagnosis | ICD-10-CM |
| D69.51 | Posttransfusion purpura | Diagnosis | ICD-10-CM |
| D69.59 | Other secondary thrombocytopenia | Diagnosis | ICD-10-CM |
| D69.6 | Thrombocytopenia, unspecified | Diagnosis | ICD-10-CM |
| D69.8 | Other specified hemorrhagic conditions | Diagnosis | ICD-10-CM |
| D69.9 | Hemorrhagic condition, unspecified | Diagnosis | ICD-10-CM |
| D70.0 | Congenital agranulocytosis | Diagnosis | ICD-10-CM |
| D70.1 | Agranulocytosis secondary to cancer chemotherapy | Diagnosis | ICD-10-CM |
| D70.2 | Other drug-induced agranulocytosis | Diagnosis | ICD-10-CM |
| D70.3 | Neutropenia due to infection | Diagnosis | ICD-10-CM |
| D70.4 | Cyclic neutropenia | Diagnosis | ICD-10-CM |
| D70.8 | Other neutropenia | Diagnosis | ICD-10-CM |
| D70.9 | Neutropenia, unspecified | Diagnosis | ICD-10-CM |
| D71 | Functional disorders of polymorphonuclear neutrophils | Diagnosis | ICD-10-CM |
| D72.0 | Genetic anomalies of leukocytes | Diagnosis | ICD-10-CM |
| D72.810 | Lymphocytopenia | Diagnosis | ICD-10-CM |
| D72.818 | Other decreased white blood cell count | Diagnosis | ICD-10-CM |
| D72.819 | Decreased white blood cell count, unspecified | Diagnosis | ICD-10-CM |
| D72.825 | Bandemia | Diagnosis | ICD-10-CM |
| D72.828 | Other elevated white blood cell count | Diagnosis | ICD-10-CM |
| D72.829 | Elevated white blood cell count, unspecified | Diagnosis | ICD-10-CM |
| D72.89 | Other specified disorders of white blood cells | Diagnosis | ICD-10-CM |
| D72.9 | Disorder of white blood cells, unspecified | Diagnosis | ICD-10-CM |
| D73.0 | Hyposplenism | Diagnosis | ICD-10-CM |
| D73.1 | Hypersplenism | Diagnosis | ICD-10-CM |
| D73.2 | Chronic congestive splenomegaly | Diagnosis | ICD-10-CM |
| D73.81 | Neutropenic splenomegaly | Diagnosis | ICD-10-CM |
| D73.89 | Other diseases of spleen | Diagnosis | ICD-10-CM |
| D73.9 | Disease of spleen, unspecified | Diagnosis | ICD-10-CM |
| D75.81 | Myelofibrosis | Diagnosis | ICD-10-CM |
| D75.82 | Heparin induced thrombocytopenia (HIT) | Diagnosis | ICD-10-CM |
| D75.89 | Other specified diseases of blood and blood-forming organs | Diagnosis | ICD-10-CM |
| D75.9 | Disease of blood and blood-forming organs, unspecified | Diagnosis | ICD-10-CM |
| D76.1 | Hemophagocytic lymphohistiocytosis | Diagnosis | ICD-10-CM |
| D76.2 | Hemophagocytic syndrome, infection-associated | Diagnosis | ICD-10-CM |
| D76.3 | Other histiocytosis syndromes | Diagnosis | ICD-10-CM |
| D77 | Other disorders of blood and blood-forming organs in diseases classified elsewhere | Diagnosis | ICD-10-CM |
| D89.2 | Hypergammaglobulinemia, unspecified | Diagnosis | ICD-10-CM |
| **Nutritional Disorders** | | | |
| E40 | Kwashiorkor | Diagnosis | ICD-10-CM |
| E41 | Nutritional marasmus | Diagnosis | ICD-10-CM |
| E42 | Marasmic kwashiorkor | Diagnosis | ICD-10-CM |
| E43 | Unspecified severe protein-calorie malnutrition | Diagnosis | ICD-10-CM |
| E44.0 | Moderate protein-calorie malnutrition | Diagnosis | ICD-10-CM |
| E44.1 | Mild protein-calorie malnutrition | Diagnosis | ICD-10-CM |
| E45 | Retarded development following protein-calorie malnutrition | Diagnosis | ICD-10-CM |
| E46 | Unspecified protein-calorie malnutrition | Diagnosis | ICD-10-CM |
| E50.0 | Vitamin A deficiency with conjunctival xerosis | Diagnosis | ICD-10-CM |
| E50.1 | Vitamin A deficiency with Bitot's spot and conjunctival xerosis | Diagnosis | ICD-10-CM |
| E50.2 | Vitamin A deficiency with corneal xerosis | Diagnosis | ICD-10-CM |
| E50.3 | Vitamin A deficiency with corneal ulceration and xerosis | Diagnosis | ICD-10-CM |
| E50.4 | Vitamin A deficiency with keratomalacia | Diagnosis | ICD-10-CM |
| E50.5 | Vitamin A deficiency with night blindness | Diagnosis | ICD-10-CM |
| E50.6 | Vitamin A deficiency with xerophthalmic scars of cornea | Diagnosis | ICD-10-CM |
| E50.7 | Other ocular manifestations of vitamin A deficiency | Diagnosis | ICD-10-CM |
| E50.8 | Other manifestations of vitamin A deficiency | Diagnosis | ICD-10-CM |
| E50.9 | Vitamin A deficiency, unspecified | Diagnosis | ICD-10-CM |
| E51.11 | Dry beriberi | Diagnosis | ICD-10-CM |
| E51.12 | Wet beriberi | Diagnosis | ICD-10-CM |
| E51.2 | Wernicke's encephalopathy | Diagnosis | ICD-10-CM |
| E51.8 | Other manifestations of thiamine deficiency | Diagnosis | ICD-10-CM |
| E51.9 | Thiamine deficiency, unspecified | Diagnosis | ICD-10-CM |
| E52 | Niacin deficiency [pellagra] | Diagnosis | ICD-10-CM |
| E53.0 | Riboflavin deficiency | Diagnosis | ICD-10-CM |
| E53.1 | Pyridoxine deficiency | Diagnosis | ICD-10-CM |
| E53.8 | Deficiency of other specified B group vitamins | Diagnosis | ICD-10-CM |
| E53.9 | Vitamin B deficiency, unspecified | Diagnosis | ICD-10-CM |
| E54 | Ascorbic acid deficiency | Diagnosis | ICD-10-CM |
| E55.0 | Rickets, active | Diagnosis | ICD-10-CM |
| E55.9 | Vitamin D deficiency, unspecified | Diagnosis | ICD-10-CM |
| E56.0 | Deficiency of vitamin E | Diagnosis | ICD-10-CM |
| E56.1 | Deficiency of vitamin K | Diagnosis | ICD-10-CM |
| E56.8 | Deficiency of other vitamins | Diagnosis | ICD-10-CM |
| E56.9 | Vitamin deficiency, unspecified | Diagnosis | ICD-10-CM |
| E58 | Dietary calcium deficiency | Diagnosis | ICD-10-CM |
| E59 | Dietary selenium deficiency | Diagnosis | ICD-10-CM |
| E60 | Dietary zinc deficiency | Diagnosis | ICD-10-CM |
| E61.0 | Copper deficiency | Diagnosis | ICD-10-CM |
| E61.1 | Iron deficiency | Diagnosis | ICD-10-CM |
| E61.2 | Magnesium deficiency | Diagnosis | ICD-10-CM |
| E61.3 | Manganese deficiency | Diagnosis | ICD-10-CM |
| E61.4 | Chromium deficiency | Diagnosis | ICD-10-CM |
| E61.5 | Molybdenum deficiency | Diagnosis | ICD-10-CM |
| E61.6 | Vanadium deficiency | Diagnosis | ICD-10-CM |
| E61.7 | Deficiency of multiple nutrient elements | Diagnosis | ICD-10-CM |
| E61.8 | Deficiency of other specified nutrient elements | Diagnosis | ICD-10-CM |
| E61.9 | Deficiency of nutrient element, unspecified | Diagnosis | ICD-10-CM |
| E63.0 | Essential fatty acid [EFA] deficiency | Diagnosis | ICD-10-CM |
| E63.1 | Imbalance of constituents of food intake | Diagnosis | ICD-10-CM |
| E63.8 | Other specified nutritional deficiencies | Diagnosis | ICD-10-CM |
| E63.9 | Nutritional deficiency, unspecified | Diagnosis | ICD-10-CM |
| E64.0 | Sequelae of protein-calorie malnutrition | Diagnosis | ICD-10-CM |
| E64.1 | Sequelae of vitamin A deficiency | Diagnosis | ICD-10-CM |
| E64.2 | Sequelae of vitamin C deficiency | Diagnosis | ICD-10-CM |
| E64.3 | Sequelae of rickets | Diagnosis | ICD-10-CM |
| E64.8 | Sequelae of other nutritional deficiencies | Diagnosis | ICD-10-CM |
| E64.9 | Sequelae of unspecified nutritional deficiency | Diagnosis | ICD-10-CM |
| E67.0 | Hypervitaminosis A | Diagnosis | ICD-10-CM |
| E67.1 | Hypercarotenemia | Diagnosis | ICD-10-CM |
| E67.2 | Megavitamin-B6 syndrome | Diagnosis | ICD-10-CM |
| E67.3 | Hypervitaminosis D | Diagnosis | ICD-10-CM |
| E67.8 | Other specified hyperalimentation | Diagnosis | ICD-10-CM |
| E68 | Sequelae of hyperalimentation | Diagnosis | ICD-10-CM |
| M83.3 | Adult osteomalacia due to malnutrition | Diagnosis | ICD-10-CM |
| **Smoking** | | | |
| F17.200 | Nicotine dependence, unspecified, uncomplicated | Diagnosis | ICD-10-CM |
| F17.201 | Nicotine dependence, unspecified, in remission | Diagnosis | ICD-10-CM |
| F17.210 | Nicotine dependence, cigarettes, uncomplicated | Diagnosis | ICD-10-CM |
| F17.211 | Nicotine dependence, cigarettes, in remission | Diagnosis | ICD-10-CM |
| F17.290 | Nicotine dependence, other tobacco product, uncomplicated | Diagnosis | ICD-10-CM |
| F17.291 | Nicotine dependence, other tobacco product, in remission | Diagnosis | ICD-10-CM |
| O99.330 | Smoking (tobacco) complicating pregnancy, unspecified trimester | Diagnosis | ICD-10-CM |
| O99.331 | Smoking (tobacco) complicating pregnancy, first trimester | Diagnosis | ICD-10-CM |
| O99.332 | Smoking (tobacco) complicating pregnancy, second trimester | Diagnosis | ICD-10-CM |
| O99.333 | Smoking (tobacco) complicating pregnancy, third trimester | Diagnosis | ICD-10-CM |
| O99.334 | Smoking (tobacco) complicating childbirth | Diagnosis | ICD-10-CM |
| O99.335 | Smoking (tobacco) complicating the puerperium | Diagnosis | ICD-10-CM |
| T65.221A | Toxic effect of tobacco cigarettes, accidental (unintentional), initial encounter | Diagnosis | ICD-10-CM |
| T65.222A | Toxic effect of tobacco cigarettes, intentional self-harm, initial encounter | Diagnosis | ICD-10-CM |
| T65.223A | Toxic effect of tobacco cigarettes, assault, initial encounter | Diagnosis | ICD-10-CM |
| T65.224A | Toxic effect of tobacco cigarettes, undetermined, initial encounter | Diagnosis | ICD-10-CM |
| T65.291A | Toxic effect of other tobacco and nicotine, accidental (unintentional), initial encounter | Diagnosis | ICD-10-CM |
| T65.292A | Toxic effect of other tobacco and nicotine, intentional self-harm, initial encounter | Diagnosis | ICD-10-CM |
| T65.293A | Toxic effect of other tobacco and nicotine, assault, initial encounter | Diagnosis | ICD-10-CM |
| T65.294A | Toxic effect of other tobacco and nicotine, undetermined, initial encounter | Diagnosis | ICD-10-CM |
| F17.203 | Nicotine dependence unspecified, with withdrawal | Diagnosis | ICD-10-CM |
| F17.208 | Nicotine dependence unspecified, with other nicotine induced disorders | Diagnosis | ICD-10-CM |
| F17.209 | Nicotine dependence unspecified, unspecified nicotine induced disorders | Diagnosis | ICD-10-CM |
| F17.213 | Nicotine dependence, cigarettes, with withdrawal | Diagnosis | ICD-10-CM |
| F17.218 | Nicotine dependence, cigarettes, with other nicotine induced disorders | Diagnosis | ICD-10-CM |
| F17.219 | Nicotine dependence, cigarettes, with unspecified nicotine induced disorders | Diagnosis | ICD-10-CM |
| F17.291 | Nicotine dependence, other tobacco product, in remission | Diagnosis | ICD-10-CM |
| F17.293 | Nicotine dependence, other tobacco product, with withdrawal | Diagnosis | ICD-10-CM |
| F17.298 | Nicotine dependence, other tobacco product, with other nicotine induced disorders | Diagnosis | ICD-10-CM |
| F17.299 | Nicotine dependence, other tobacco product, with unspecified nicotine induced disorders | Diagnosis | ICD-10-CM |
| Z72.0 | Tobacco use | Diagnosis | ICD-10-CM |
| 1034F | Current tobacco smoker (CAD, CAP, COPD, PV) (DM) | Procedure | CPT Category II |
| 4001F | Tobacco use cessation intervention, pharmacologic therapy (COPD, CAD, CAP, PV, Asthma) (DM) (PV) | Procedure | CPT Category II |
| G9276 | Documentation that patient is a current tobacco user | Procedure | HCPCS |
| G9458 | Patient documented as tobacco user and received tobacco cessation intervention (must include at least one of the following: advice given to quit smoking or tobacco use, counseling on the benefits of quitting smoking or tobacco use, assistance with or referral to external smoking or tobacco cessation support programs, or current enrollment in smoking or tobacco use cessation program) if identified as a tobacco user | Procedure | HCPCS |
| **Documented Pregnancy Marker or Gestational Age** | | | |
| H35.101 | Retinopathy of prematurity, unspecified, right eye | Diagnosis | ICD-10-CM |
| H35.102 | Retinopathy of prematurity, unspecified, left eye | Diagnosis | ICD-10-CM |
| H35.103 | Retinopathy of prematurity, unspecified, bilateral | Diagnosis | ICD-10-CM |
| H35.109 | Retinopathy of prematurity, unspecified, unspecified eye | Diagnosis | ICD-10-CM |
| H35.111 | Retinopathy of prematurity, stage 0, right eye | Diagnosis | ICD-10-CM |
| H35.112 | Retinopathy of prematurity, stage 0, left eye | Diagnosis | ICD-10-CM |
| H35.113 | Retinopathy of prematurity, stage 0, bilateral | Diagnosis | ICD-10-CM |
| H35.119 | Retinopathy of prematurity, stage 0, unspecified eye | Diagnosis | ICD-10-CM |
| H35.121 | Retinopathy of prematurity, stage 1, right eye | Diagnosis | ICD-10-CM |
| H35.122 | Retinopathy of prematurity, stage 1, left eye | Diagnosis | ICD-10-CM |
| H35.123 | Retinopathy of prematurity, stage 1, bilateral | Diagnosis | ICD-10-CM |
| H35.129 | Retinopathy of prematurity, stage 1, unspecified eye | Diagnosis | ICD-10-CM |
| H35.131 | Retinopathy of prematurity, stage 2, right eye | Diagnosis | ICD-10-CM |
| H35.132 | Retinopathy of prematurity, stage 2, left eye | Diagnosis | ICD-10-CM |
| H35.133 | Retinopathy of prematurity, stage 2, bilateral | Diagnosis | ICD-10-CM |
| H35.139 | Retinopathy of prematurity, stage 2, unspecified eye | Diagnosis | ICD-10-CM |
| H35.141 | Retinopathy of prematurity, stage 3, right eye | Diagnosis | ICD-10-CM |
| H35.142 | Retinopathy of prematurity, stage 3, left eye | Diagnosis | ICD-10-CM |
| H35.143 | Retinopathy of prematurity, stage 3, bilateral | Diagnosis | ICD-10-CM |
| H35.149 | Retinopathy of prematurity, stage 3, unspecified eye | Diagnosis | ICD-10-CM |
| H35.151 | Retinopathy of prematurity, stage 4, right eye | Diagnosis | ICD-10-CM |
| H35.152 | Retinopathy of prematurity, stage 4, left eye | Diagnosis | ICD-10-CM |
| H35.153 | Retinopathy of prematurity, stage 4, bilateral | Diagnosis | ICD-10-CM |
| H35.159 | Retinopathy of prematurity, stage 4, unspecified eye | Diagnosis | ICD-10-CM |
| H35.161 | Retinopathy of prematurity, stage 5, right eye | Diagnosis | ICD-10-CM |
| H35.162 | Retinopathy of prematurity, stage 5, left eye | Diagnosis | ICD-10-CM |
| H35.163 | Retinopathy of prematurity, stage 5, bilateral | Diagnosis | ICD-10-CM |
| H35.169 | Retinopathy of prematurity, stage 5, unspecified eye | Diagnosis | ICD-10-CM |
| O00.00 | Abdominal pregnancy without intrauterine pregnancy | Diagnosis | ICD-10-CM |
| O00.01 | Abdominal pregnancy with intrauterine pregnancy | Diagnosis | ICD-10-CM |
| O00.101 | Right tubal pregnancy without intrauterine pregnancy | Diagnosis | ICD-10-CM |
| O00.102 | Left tubal pregnancy without intrauterine pregnancy | Diagnosis | ICD-10-CM |
| O00.109 | Unspecified tubal pregnancy without intrauterine pregnancy | Diagnosis | ICD-10-CM |
| O00.111 | Right tubal pregnancy with intrauterine pregnancy | Diagnosis | ICD-10-CM |
| O00.112 | Left tubal pregnancy with intrauterine pregnancy | Diagnosis | ICD-10-CM |
| O00.119 | Unspecified tubal pregnancy with intrauterine pregnancy | Diagnosis | ICD-10-CM |
| O00.201 | Right ovarian pregnancy without intrauterine pregnancy | Diagnosis | ICD-10-CM |
| O00.202 | Left ovarian pregnancy without intrauterine pregnancy | Diagnosis | ICD-10-CM |
| O00.209 | Unspecified ovarian pregnancy without intrauterine pregnancy | Diagnosis | ICD-10-CM |
| O00.211 | Right ovarian pregnancy with intrauterine pregnancy | Diagnosis | ICD-10-CM |
| O00.212 | Left ovarian pregnancy with intrauterine pregnancy | Diagnosis | ICD-10-CM |
| O00.219 | Unspecified ovarian pregnancy with intrauterine pregnancy | Diagnosis | ICD-10-CM |
| O00.80 | Other ectopic pregnancy without intrauterine pregnancy | Diagnosis | ICD-10-CM |
| O00.81 | Other ectopic pregnancy with intrauterine pregnancy | Diagnosis | ICD-10-CM |
| O00.90 | Unspecified ectopic pregnancy without intrauterine pregnancy | Diagnosis | ICD-10-CM |
| O00.91 | Unspecified ectopic pregnancy with intrauterine pregnancy | Diagnosis | ICD-10-CM |
| O02.1 | Missed abortion | Diagnosis | ICD-10-CM |
| O03.0 | Genital tract and pelvic infection following incomplete spontaneous abortion | Diagnosis | ICD-10-CM |
| O03.1 | Delayed or excessive hemorrhage following incomplete spontaneous abortion | Diagnosis | ICD-10-CM |
| O03.2 | Embolism following incomplete spontaneous abortion | Diagnosis | ICD-10-CM |
| O03.30 | Unspecified complication following incomplete spontaneous abortion | Diagnosis | ICD-10-CM |
| O03.31 | Shock following incomplete spontaneous abortion | Diagnosis | ICD-10-CM |
| O03.32 | Renal failure following incomplete spontaneous abortion | Diagnosis | ICD-10-CM |
| O03.33 | Metabolic disorder following incomplete spontaneous abortion | Diagnosis | ICD-10-CM |
| O03.34 | Damage to pelvic organs following incomplete spontaneous abortion | Diagnosis | ICD-10-CM |
| O03.35 | Other venous complications following incomplete spontaneous abortion | Diagnosis | ICD-10-CM |
| O03.36 | Cardiac arrest following incomplete spontaneous abortion | Diagnosis | ICD-10-CM |
| O03.37 | Sepsis following incomplete spontaneous abortion | Diagnosis | ICD-10-CM |
| O03.38 | Urinary tract infection following incomplete spontaneous abortion | Diagnosis | ICD-10-CM |
| O03.39 | Incomplete spontaneous abortion with other complications | Diagnosis | ICD-10-CM |
| O03.4 | Incomplete spontaneous abortion without complication | Diagnosis | ICD-10-CM |
| O03.5 | Genital tract and pelvic infection following complete or unspecified spontaneous abortion | Diagnosis | ICD-10-CM |
| O03.6 | Delayed or excessive hemorrhage following complete or unspecified spontaneous abortion | Diagnosis | ICD-10-CM |
| O03.7 | Embolism following complete or unspecified spontaneous abortion | Diagnosis | ICD-10-CM |
| O03.80 | Unspecified complication following complete or unspecified spontaneous abortion | Diagnosis | ICD-10-CM |
| O03.81 | Shock following complete or unspecified spontaneous abortion | Diagnosis | ICD-10-CM |
| O03.82 | Renal failure following complete or unspecified spontaneous abortion | Diagnosis | ICD-10-CM |
| O03.83 | Metabolic disorder following complete or unspecified spontaneous abortion | Diagnosis | ICD-10-CM |
| O03.84 | Damage to pelvic organs following complete or unspecified spontaneous abortion | Diagnosis | ICD-10-CM |
| O03.85 | Other venous complications following complete or unspecified spontaneous abortion | Diagnosis | ICD-10-CM |
| O03.86 | Cardiac arrest following complete or unspecified spontaneous abortion | Diagnosis | ICD-10-CM |
| O03.87 | Sepsis following complete or unspecified spontaneous abortion | Diagnosis | ICD-10-CM |
| O03.88 | Urinary tract infection following complete or unspecified spontaneous abortion | Diagnosis | ICD-10-CM |
| O03.89 | Complete or unspecified spontaneous abortion with other complications | Diagnosis | ICD-10-CM |
| O03.9 | Complete or unspecified spontaneous abortion without complication | Diagnosis | ICD-10-CM |
| O04.5 | Genital tract and pelvic infection following (induced) termination of pregnancy | Diagnosis | ICD-10-CM |
| O04.6 | Delayed or excessive hemorrhage following (induced) termination of pregnancy | Diagnosis | ICD-10-CM |
| O04.7 | Embolism following (induced) termination of pregnancy | Diagnosis | ICD-10-CM |
| O04.80 | (Induced) termination of pregnancy with unspecified complications | Diagnosis | ICD-10-CM |
| O04.81 | Shock following (induced) termination of pregnancy | Diagnosis | ICD-10-CM |
| O04.82 | Renal failure following (induced) termination of pregnancy | Diagnosis | ICD-10-CM |
| O04.83 | Metabolic disorder following (induced) termination of pregnancy | Diagnosis | ICD-10-CM |
| O04.84 | Damage to pelvic organs following (induced) termination of pregnancy | Diagnosis | ICD-10-CM |
| O04.85 | Other venous complications following (induced) termination of pregnancy | Diagnosis | ICD-10-CM |
| O04.86 | Cardiac arrest following (induced) termination of pregnancy | Diagnosis | ICD-10-CM |
| O04.87 | Sepsis following (induced) termination of pregnancy | Diagnosis | ICD-10-CM |
| O04.88 | Urinary tract infection following (induced) termination of pregnancy | Diagnosis | ICD-10-CM |
| O04.89 | (Induced) termination of pregnancy with other complications | Diagnosis | ICD-10-CM |
| O07.0 | Genital tract and pelvic infection following failed attempted termination of pregnancy | Diagnosis | ICD-10-CM |
| O07.1 | Delayed or excessive hemorrhage following failed attempted termination of pregnancy | Diagnosis | ICD-10-CM |
| O07.2 | Embolism following failed attempted termination of pregnancy | Diagnosis | ICD-10-CM |
| O07.30 | Failed attempted termination of pregnancy with unspecified complications | Diagnosis | ICD-10-CM |
| O07.31 | Shock following failed attempted termination of pregnancy | Diagnosis | ICD-10-CM |
| O07.32 | Renal failure following failed attempted termination of pregnancy | Diagnosis | ICD-10-CM |
| O07.33 | Metabolic disorder following failed attempted termination of pregnancy | Diagnosis | ICD-10-CM |
| O07.34 | Damage to pelvic organs following failed attempted termination of pregnancy | Diagnosis | ICD-10-CM |
| O07.35 | Other venous complications following failed attempted termination of pregnancy | Diagnosis | ICD-10-CM |
| O07.36 | Cardiac arrest following failed attempted termination of pregnancy | Diagnosis | ICD-10-CM |
| O07.37 | Sepsis following failed attempted termination of pregnancy | Diagnosis | ICD-10-CM |
| O07.38 | Urinary tract infection following failed attempted termination of pregnancy | Diagnosis | ICD-10-CM |
| O07.39 | Failed attempted termination of pregnancy with other complications | Diagnosis | ICD-10-CM |
| O07.4 | Failed attempted termination of pregnancy without complication | Diagnosis | ICD-10-CM |
| O09.00 | Supervision of pregnancy with history of infertility, unspecified trimester | Diagnosis | ICD-10-CM |
| O09.01 | Supervision of pregnancy with history of infertility, first trimester | Diagnosis | ICD-10-CM |
| O09.02 | Supervision of pregnancy with history of infertility, second trimester | Diagnosis | ICD-10-CM |
| O09.03 | Supervision of pregnancy with history of infertility, third trimester | Diagnosis | ICD-10-CM |
| O09.10 | Supervision of pregnancy with history of ectopic pregnancy, unspecified trimester | Diagnosis | ICD-10-CM |
| O09.11 | Supervision of pregnancy with history of ectopic pregnancy, first trimester | Diagnosis | ICD-10-CM |
| O09.12 | Supervision of pregnancy with history of ectopic pregnancy, second trimester | Diagnosis | ICD-10-CM |
| O09.13 | Supervision of pregnancy with history of ectopic pregnancy, third trimester | Diagnosis | ICD-10-CM |
| O09.211 | Supervision of pregnancy with history of pre-term labor, first trimester | Diagnosis | ICD-10-CM |
| O09.212 | Supervision of pregnancy with history of pre-term labor, second trimester | Diagnosis | ICD-10-CM |
| O09.213 | Supervision of pregnancy with history of pre-term labor, third trimester | Diagnosis | ICD-10-CM |
| O09.219 | Supervision of pregnancy with history of pre-term labor, unspecified trimester | Diagnosis | ICD-10-CM |
| O09.291 | Supervision of pregnancy with other poor reproductive or obstetric history, first trimester | Diagnosis | ICD-10-CM |
| O09.292 | Supervision of pregnancy with other poor reproductive or obstetric history, second trimester | Diagnosis | ICD-10-CM |
| O09.293 | Supervision of pregnancy with other poor reproductive or obstetric history, third trimester | Diagnosis | ICD-10-CM |
| O09.299 | Supervision of pregnancy with other poor reproductive or obstetric history, unspecified trimester | Diagnosis | ICD-10-CM |
| O09.30 | Supervision of pregnancy with insufficient antenatal care, unspecified trimester | Diagnosis | ICD-10-CM |
| O09.31 | Supervision of pregnancy with insufficient antenatal care, first trimester | Diagnosis | ICD-10-CM |
| O09.32 | Supervision of pregnancy with insufficient antenatal care, second trimester | Diagnosis | ICD-10-CM |
| O09.33 | Supervision of pregnancy with insufficient antenatal care, third trimester | Diagnosis | ICD-10-CM |
| O09.40 | Supervision of pregnancy with grand multiparity, unspecified trimester | Diagnosis | ICD-10-CM |
| O09.41 | Supervision of pregnancy with grand multiparity, first trimester | Diagnosis | ICD-10-CM |
| O09.42 | Supervision of pregnancy with grand multiparity, second trimester | Diagnosis | ICD-10-CM |
| O09.43 | Supervision of pregnancy with grand multiparity, third trimester | Diagnosis | ICD-10-CM |
| O09.511 | Supervision of elderly primigravida, first trimester | Diagnosis | ICD-10-CM |
| O09.512 | Supervision of elderly primigravida, second trimester | Diagnosis | ICD-10-CM |
| O09.513 | Supervision of elderly primigravida, third trimester | Diagnosis | ICD-10-CM |
| O09.519 | Supervision of elderly primigravida, unspecified trimester | Diagnosis | ICD-10-CM |
| O09.521 | Supervision of elderly multigravida, first trimester | Diagnosis | ICD-10-CM |
| O09.522 | Supervision of elderly multigravida, second trimester | Diagnosis | ICD-10-CM |
| O09.523 | Supervision of elderly multigravida, third trimester | Diagnosis | ICD-10-CM |
| O09.529 | Supervision of elderly multigravida, unspecified trimester | Diagnosis | ICD-10-CM |
| O09.611 | Supervision of young primigravida, first trimester | Diagnosis | ICD-10-CM |
| O09.612 | Supervision of young primigravida, second trimester | Diagnosis | ICD-10-CM |
| O09.613 | Supervision of young primigravida, third trimester | Diagnosis | ICD-10-CM |
| O09.619 | Supervision of young primigravida, unspecified trimester | Diagnosis | ICD-10-CM |
| O09.621 | Supervision of young multigravida, first trimester | Diagnosis | ICD-10-CM |
| O09.622 | Supervision of young multigravida, second trimester | Diagnosis | ICD-10-CM |
| O09.623 | Supervision of young multigravida, third trimester | Diagnosis | ICD-10-CM |
| O09.629 | Supervision of young multigravida, unspecified trimester | Diagnosis | ICD-10-CM |
| O09.70 | Supervision of high risk pregnancy due to social problems, unspecified trimester | Diagnosis | ICD-10-CM |
| O09.71 | Supervision of high risk pregnancy due to social problems, first trimester | Diagnosis | ICD-10-CM |
| O09.72 | Supervision of high risk pregnancy due to social problems, second trimester | Diagnosis | ICD-10-CM |
| O09.73 | Supervision of high risk pregnancy due to social problems, third trimester | Diagnosis | ICD-10-CM |
| O09.811 | Supervision of pregnancy resulting from assisted reproductive technology, first trimester | Diagnosis | ICD-10-CM |
| O09.812 | Supervision of pregnancy resulting from assisted reproductive technology, second trimester | Diagnosis | ICD-10-CM |
| O09.813 | Supervision of pregnancy resulting from assisted reproductive technology, third trimester | Diagnosis | ICD-10-CM |
| O09.819 | Supervision of pregnancy resulting from assisted reproductive technology, unspecified trimester | Diagnosis | ICD-10-CM |
| O09.821 | Supervision of pregnancy with history of in utero procedure during previous pregnancy, first trimester | Diagnosis | ICD-10-CM |
| O09.822 | Supervision of pregnancy with history of in utero procedure during previous pregnancy, second trimester | Diagnosis | ICD-10-CM |
| O09.823 | Supervision of pregnancy with history of in utero procedure during previous pregnancy, third trimester | Diagnosis | ICD-10-CM |
| O09.829 | Supervision of pregnancy with history of in utero procedure during previous pregnancy, unspecified trimester | Diagnosis | ICD-10-CM |
| O09.891 | Supervision of other high risk pregnancies, first trimester | Diagnosis | ICD-10-CM |
| O09.892 | Supervision of other high risk pregnancies, second trimester | Diagnosis | ICD-10-CM |
| O09.893 | Supervision of other high risk pregnancies, third trimester | Diagnosis | ICD-10-CM |
| O09.899 | Supervision of other high risk pregnancies, unspecified trimester | Diagnosis | ICD-10-CM |
| O09.90 | Supervision of high risk pregnancy, unspecified, unspecified trimester | Diagnosis | ICD-10-CM |
| O09.91 | Supervision of high risk pregnancy, unspecified, first trimester | Diagnosis | ICD-10-CM |
| O09.92 | Supervision of high risk pregnancy, unspecified, second trimester | Diagnosis | ICD-10-CM |
| O09.93 | Supervision of high risk pregnancy, unspecified, third trimester | Diagnosis | ICD-10-CM |
| O09.A0 | Supervision of pregnancy with history of molar pregnancy, unspecified trimester | Diagnosis | ICD-10-CM |
| O09.A1 | Supervision of pregnancy with history of molar pregnancy, first trimester | Diagnosis | ICD-10-CM |
| O09.A2 | Supervision of pregnancy with history of molar pregnancy, second trimester | Diagnosis | ICD-10-CM |
| O09.A3 | Supervision of pregnancy with history of molar pregnancy, third trimester | Diagnosis | ICD-10-CM |
| O10.011 | Pre-existing essential hypertension complicating pregnancy, first trimester | Diagnosis | ICD-10-CM |
| O10.012 | Pre-existing essential hypertension complicating pregnancy, second trimester | Diagnosis | ICD-10-CM |
| O10.013 | Pre-existing essential hypertension complicating pregnancy, third trimester | Diagnosis | ICD-10-CM |
| O10.019 | Pre-existing essential hypertension complicating pregnancy, unspecified trimester | Diagnosis | ICD-10-CM |
| O10.02 | Pre-existing essential hypertension complicating childbirth | Diagnosis | ICD-10-CM |
| O10.03 | Pre-existing essential hypertension complicating the puerperium | Diagnosis | ICD-10-CM |
| O10.111 | Pre-existing hypertensive heart disease complicating pregnancy, first trimester | Diagnosis | ICD-10-CM |
| O10.112 | Pre-existing hypertensive heart disease complicating pregnancy, second trimester | Diagnosis | ICD-10-CM |
| O10.113 | Pre-existing hypertensive heart disease complicating pregnancy, third trimester | Diagnosis | ICD-10-CM |
| O10.119 | Pre-existing hypertensive heart disease complicating pregnancy, unspecified trimester | Diagnosis | ICD-10-CM |
| O10.12 | Pre-existing hypertensive heart disease complicating childbirth | Diagnosis | ICD-10-CM |
| O10.13 | Pre-existing hypertensive heart disease complicating the puerperium | Diagnosis | ICD-10-CM |
| O10.211 | Pre-existing hypertensive chronic kidney disease complicating pregnancy, first trimester | Diagnosis | ICD-10-CM |
| O10.212 | Pre-existing hypertensive chronic kidney disease complicating pregnancy, second trimester | Diagnosis | ICD-10-CM |
| O10.213 | Pre-existing hypertensive chronic kidney disease complicating pregnancy, third trimester | Diagnosis | ICD-10-CM |
| O10.219 | Pre-existing hypertensive chronic kidney disease complicating pregnancy, unspecified trimester | Diagnosis | ICD-10-CM |
| O10.22 | Pre-existing hypertensive chronic kidney disease complicating childbirth | Diagnosis | ICD-10-CM |
| O10.23 | Pre-existing hypertensive chronic kidney disease complicating the puerperium | Diagnosis | ICD-10-CM |
| O10.311 | Pre-existing hypertensive heart and chronic kidney disease complicating pregnancy, first trimester | Diagnosis | ICD-10-CM |
| O10.312 | Pre-existing hypertensive heart and chronic kidney disease complicating pregnancy, second trimester | Diagnosis | ICD-10-CM |
| O10.313 | Pre-existing hypertensive heart and chronic kidney disease complicating pregnancy, third trimester | Diagnosis | ICD-10-CM |
| O10.319 | Pre-existing hypertensive heart and chronic kidney disease complicating pregnancy, unspecified trimester | Diagnosis | ICD-10-CM |
| O10.32 | Pre-existing hypertensive heart and chronic kidney disease complicating childbirth | Diagnosis | ICD-10-CM |
| O10.33 | Pre-existing hypertensive heart and chronic kidney disease complicating the puerperium | Diagnosis | ICD-10-CM |
| O10.411 | Pre-existing secondary hypertension complicating pregnancy, first trimester | Diagnosis | ICD-10-CM |
| O10.412 | Pre-existing secondary hypertension complicating pregnancy, second trimester | Diagnosis | ICD-10-CM |
| O10.413 | Pre-existing secondary hypertension complicating pregnancy, third trimester | Diagnosis | ICD-10-CM |
| O10.419 | Pre-existing secondary hypertension complicating pregnancy, unspecified trimester | Diagnosis | ICD-10-CM |
| O10.42 | Pre-existing secondary hypertension complicating childbirth | Diagnosis | ICD-10-CM |
| O10.43 | Pre-existing secondary hypertension complicating the puerperium | Diagnosis | ICD-10-CM |
| O10.911 | Unspecified pre-existing hypertension complicating pregnancy, first trimester | Diagnosis | ICD-10-CM |
| O10.912 | Unspecified pre-existing hypertension complicating pregnancy, second trimester | Diagnosis | ICD-10-CM |
| O10.913 | Unspecified pre-existing hypertension complicating pregnancy, third trimester | Diagnosis | ICD-10-CM |
| O10.919 | Unspecified pre-existing hypertension complicating pregnancy, unspecified trimester | Diagnosis | ICD-10-CM |
| O10.92 | Unspecified pre-existing hypertension complicating childbirth | Diagnosis | ICD-10-CM |
| O10.93 | Unspecified pre-existing hypertension complicating the puerperium | Diagnosis | ICD-10-CM |
| O11.1 | Pre-existing hypertension with pre-eclampsia, first trimester | Diagnosis | ICD-10-CM |
| O11.2 | Pre-existing hypertension with pre-eclampsia, second trimester | Diagnosis | ICD-10-CM |
| O11.3 | Pre-existing hypertension with pre-eclampsia, third trimester | Diagnosis | ICD-10-CM |
| O11.4 | Pre-existing hypertension with pre-eclampsia, complicating childbirth | Diagnosis | ICD-10-CM |
| O11.5 | Pre-existing hypertension with pre-eclampsia, complicating the puerperium | Diagnosis | ICD-10-CM |
| O11.9 | Pre-existing hypertension with pre-eclampsia, unspecified trimester | Diagnosis | ICD-10-CM |
| O12.00 | Gestational edema, unspecified trimester | Diagnosis | ICD-10-CM |
| O12.01 | Gestational edema, first trimester | Diagnosis | ICD-10-CM |
| O12.02 | Gestational edema, second trimester | Diagnosis | ICD-10-CM |
| O12.03 | Gestational edema, third trimester | Diagnosis | ICD-10-CM |
| O12.04 | Gestational edema, complicating childbirth | Diagnosis | ICD-10-CM |
| O12.05 | Gestational edema, complicating the puerperium | Diagnosis | ICD-10-CM |
| O12.10 | Gestational proteinuria, unspecified trimester | Diagnosis | ICD-10-CM |
| O12.11 | Gestational proteinuria, first trimester | Diagnosis | ICD-10-CM |
| O12.12 | Gestational proteinuria, second trimester | Diagnosis | ICD-10-CM |
| O12.13 | Gestational proteinuria, third trimester | Diagnosis | ICD-10-CM |
| O12.14 | Gestational proteinuria, complicating childbirth | Diagnosis | ICD-10-CM |
| O12.15 | Gestational proteinuria, complicating the puerperium | Diagnosis | ICD-10-CM |
| O12.20 | Gestational edema with proteinuria, unspecified trimester | Diagnosis | ICD-10-CM |
| O12.21 | Gestational edema with proteinuria, first trimester | Diagnosis | ICD-10-CM |
| O12.22 | Gestational edema with proteinuria, second trimester | Diagnosis | ICD-10-CM |
| O12.23 | Gestational edema with proteinuria, third trimester | Diagnosis | ICD-10-CM |
| O12.24 | Gestational edema with proteinuria, complicating childbirth | Diagnosis | ICD-10-CM |
| O12.25 | Gestational edema with proteinuria, complicating the puerperium | Diagnosis | ICD-10-CM |
| O13.1 | Gestational [pregnancy-induced] hypertension without significant proteinuria, first trimester | Diagnosis | ICD-10-CM |
| O13.2 | Gestational [pregnancy-induced] hypertension without significant proteinuria, second trimester | Diagnosis | ICD-10-CM |
| O13.3 | Gestational [pregnancy-induced] hypertension without significant proteinuria, third trimester | Diagnosis | ICD-10-CM |
| O13.4 | Gestational [pregnancy-induced] hypertension without significant proteinuria, complicating childbirth | Diagnosis | ICD-10-CM |
| O13.5 | Gestational [pregnancy-induced] hypertension without significant proteinuria, complicating the puerperium | Diagnosis | ICD-10-CM |
| O13.9 | Gestational [pregnancy-induced] hypertension without significant proteinuria, unspecified trimester | Diagnosis | ICD-10-CM |
| O14.00 | Mild to moderate pre-eclampsia, unspecified trimester | Diagnosis | ICD-10-CM |
| O14.02 | Mild to moderate pre-eclampsia, second trimester | Diagnosis | ICD-10-CM |
| O14.03 | Mild to moderate pre-eclampsia, third trimester | Diagnosis | ICD-10-CM |
| O14.04 | Mild to moderate pre-eclampsia, complicating childbirth | Diagnosis | ICD-10-CM |
| O14.05 | Mild to moderate pre-eclampsia, complicating the puerperium | Diagnosis | ICD-10-CM |
| O14.10 | Severe pre-eclampsia, unspecified trimester | Diagnosis | ICD-10-CM |
| O14.12 | Severe pre-eclampsia, second trimester | Diagnosis | ICD-10-CM |
| O14.13 | Severe pre-eclampsia, third trimester | Diagnosis | ICD-10-CM |
| O14.14 | Severe pre-eclampsia complicating childbirth | Diagnosis | ICD-10-CM |
| O14.15 | Severe pre-eclampsia, complicating the puerperium | Diagnosis | ICD-10-CM |
| O14.20 | HELLP syndrome (HELLP), unspecified trimester | Diagnosis | ICD-10-CM |
| O14.22 | HELLP syndrome (HELLP), second trimester | Diagnosis | ICD-10-CM |
| O14.23 | HELLP syndrome (HELLP), third trimester | Diagnosis | ICD-10-CM |
| O14.24 | HELLP syndrome, complicating childbirth | Diagnosis | ICD-10-CM |
| O14.25 | HELLP syndrome, complicating the puerperium | Diagnosis | ICD-10-CM |
| O14.90 | Unspecified pre-eclampsia, unspecified trimester | Diagnosis | ICD-10-CM |
| O14.92 | Unspecified pre-eclampsia, second trimester | Diagnosis | ICD-10-CM |
| O14.93 | Unspecified pre-eclampsia, third trimester | Diagnosis | ICD-10-CM |
| O14.94 | Unspecified pre-eclampsia, complicating childbirth | Diagnosis | ICD-10-CM |
| O14.95 | Unspecified pre-eclampsia, complicating the puerperium | Diagnosis | ICD-10-CM |
| O15.00 | Eclampsia complicating pregnancy, unspecified trimester | Diagnosis | ICD-10-CM |
| O15.02 | Eclampsia complicating pregnancy, second trimester | Diagnosis | ICD-10-CM |
| O15.03 | Eclampsia complicating pregnancy, third trimester | Diagnosis | ICD-10-CM |
| O15.1 | Eclampsia complicating labor | Diagnosis | ICD-10-CM |
| O15.2 | Eclampsia complicating the puerperium | Diagnosis | ICD-10-CM |
| O15.9 | Eclampsia, unspecified as to time period | Diagnosis | ICD-10-CM |
| O16.1 | Unspecified maternal hypertension, first trimester | Diagnosis | ICD-10-CM |
| O16.2 | Unspecified maternal hypertension, second trimester | Diagnosis | ICD-10-CM |
| O16.3 | Unspecified maternal hypertension, third trimester | Diagnosis | ICD-10-CM |
| O16.4 | Unspecified maternal hypertension, complicating childbirth | Diagnosis | ICD-10-CM |
| O16.5 | Unspecified maternal hypertension, complicating the puerperium | Diagnosis | ICD-10-CM |
| O16.9 | Unspecified maternal hypertension, unspecified trimester | Diagnosis | ICD-10-CM |
| O20.0 | Threatened abortion | Diagnosis | ICD-10-CM |
| O20.8 | Other hemorrhage in early pregnancy | Diagnosis | ICD-10-CM |
| O20.9 | Hemorrhage in early pregnancy, unspecified | Diagnosis | ICD-10-CM |
| O21.0 | Mild hyperemesis gravidarum | Diagnosis | ICD-10-CM |
| O21.1 | Hyperemesis gravidarum with metabolic disturbance | Diagnosis | ICD-10-CM |
| O21.2 | Late vomiting of pregnancy | Diagnosis | ICD-10-CM |
| O21.8 | Other vomiting complicating pregnancy | Diagnosis | ICD-10-CM |
| O21.9 | Vomiting of pregnancy, unspecified | Diagnosis | ICD-10-CM |
| O22.00 | Varicose veins of lower extremity in pregnancy, unspecified trimester | Diagnosis | ICD-10-CM |
| O22.01 | Varicose veins of lower extremity in pregnancy, first trimester | Diagnosis | ICD-10-CM |
| O22.02 | Varicose veins of lower extremity in pregnancy, second trimester | Diagnosis | ICD-10-CM |
| O22.03 | Varicose veins of lower extremity in pregnancy, third trimester | Diagnosis | ICD-10-CM |
| O22.10 | Genital varices in pregnancy, unspecified trimester | Diagnosis | ICD-10-CM |
| O22.11 | Genital varices in pregnancy, first trimester | Diagnosis | ICD-10-CM |
| O22.12 | Genital varices in pregnancy, second trimester | Diagnosis | ICD-10-CM |
| O22.13 | Genital varices in pregnancy, third trimester | Diagnosis | ICD-10-CM |
| O22.20 | Superficial thrombophlebitis in pregnancy, unspecified trimester | Diagnosis | ICD-10-CM |
| O22.21 | Superficial thrombophlebitis in pregnancy, first trimester | Diagnosis | ICD-10-CM |
| O22.22 | Superficial thrombophlebitis in pregnancy, second trimester | Diagnosis | ICD-10-CM |
| O22.23 | Superficial thrombophlebitis in pregnancy, third trimester | Diagnosis | ICD-10-CM |
| O22.30 | Deep phlebothrombosis in pregnancy, unspecified trimester | Diagnosis | ICD-10-CM |
| O22.31 | Deep phlebothrombosis in pregnancy, first trimester | Diagnosis | ICD-10-CM |
| O22.32 | Deep phlebothrombosis in pregnancy, second trimester | Diagnosis | ICD-10-CM |
| O22.33 | Deep phlebothrombosis in pregnancy, third trimester | Diagnosis | ICD-10-CM |
| O22.40 | Hemorrhoids in pregnancy, unspecified trimester | Diagnosis | ICD-10-CM |
| O22.41 | Hemorrhoids in pregnancy, first trimester | Diagnosis | ICD-10-CM |
| O22.42 | Hemorrhoids in pregnancy, second trimester | Diagnosis | ICD-10-CM |
| O22.43 | Hemorrhoids in pregnancy, third trimester | Diagnosis | ICD-10-CM |
| O22.50 | Cerebral venous thrombosis in pregnancy, unspecified trimester | Diagnosis | ICD-10-CM |
| O22.51 | Cerebral venous thrombosis in pregnancy, first trimester | Diagnosis | ICD-10-CM |
| O22.52 | Cerebral venous thrombosis in pregnancy, second trimester | Diagnosis | ICD-10-CM |
| O22.53 | Cerebral venous thrombosis in pregnancy, third trimester | Diagnosis | ICD-10-CM |
| O22.8X1 | Other venous complications in pregnancy, first trimester | Diagnosis | ICD-10-CM |
| O22.8X2 | Other venous complications in pregnancy, second trimester | Diagnosis | ICD-10-CM |
| O22.8X3 | Other venous complications in pregnancy, third trimester | Diagnosis | ICD-10-CM |
| O22.8X9 | Other venous complications in pregnancy, unspecified trimester | Diagnosis | ICD-10-CM |
| O22.90 | Venous complication in pregnancy, unspecified, unspecified trimester | Diagnosis | ICD-10-CM |
| O22.91 | Venous complication in pregnancy, unspecified, first trimester | Diagnosis | ICD-10-CM |
| O22.92 | Venous complication in pregnancy, unspecified, second trimester | Diagnosis | ICD-10-CM |
| O22.93 | Venous complication in pregnancy, unspecified, third trimester | Diagnosis | ICD-10-CM |
| O23.00 | Infections of kidney in pregnancy, unspecified trimester | Diagnosis | ICD-10-CM |
| O23.01 | Infections of kidney in pregnancy, first trimester | Diagnosis | ICD-10-CM |
| O23.02 | Infections of kidney in pregnancy, second trimester | Diagnosis | ICD-10-CM |
| O23.03 | Infections of kidney in pregnancy, third trimester | Diagnosis | ICD-10-CM |
| O23.10 | Infections of bladder in pregnancy, unspecified trimester | Diagnosis | ICD-10-CM |
| O23.11 | Infections of bladder in pregnancy, first trimester | Diagnosis | ICD-10-CM |
| O23.12 | Infections of bladder in pregnancy, second trimester | Diagnosis | ICD-10-CM |
| O23.13 | Infections of bladder in pregnancy, third trimester | Diagnosis | ICD-10-CM |
| O23.20 | Infections of urethra in pregnancy, unspecified trimester | Diagnosis | ICD-10-CM |
| O23.21 | Infections of urethra in pregnancy, first trimester | Diagnosis | ICD-10-CM |
| O23.22 | Infections of urethra in pregnancy, second trimester | Diagnosis | ICD-10-CM |
| O23.23 | Infections of urethra in pregnancy, third trimester | Diagnosis | ICD-10-CM |
| O23.30 | Infections of other parts of urinary tract in pregnancy, unspecified trimester | Diagnosis | ICD-10-CM |
| O23.31 | Infections of other parts of urinary tract in pregnancy, first trimester | Diagnosis | ICD-10-CM |
| O23.32 | Infections of other parts of urinary tract in pregnancy, second trimester | Diagnosis | ICD-10-CM |
| O23.33 | Infections of other parts of urinary tract in pregnancy, third trimester | Diagnosis | ICD-10-CM |
| O23.40 | Unspecified infection of urinary tract in pregnancy, unspecified trimester | Diagnosis | ICD-10-CM |
| O23.41 | Unspecified infection of urinary tract in pregnancy, first trimester | Diagnosis | ICD-10-CM |
| O23.42 | Unspecified infection of urinary tract in pregnancy, second trimester | Diagnosis | ICD-10-CM |
| O23.43 | Unspecified infection of urinary tract in pregnancy, third trimester | Diagnosis | ICD-10-CM |
| O23.511 | Infections of cervix in pregnancy, first trimester | Diagnosis | ICD-10-CM |
| O23.512 | Infections of cervix in pregnancy, second trimester | Diagnosis | ICD-10-CM |
| O23.513 | Infections of cervix in pregnancy, third trimester | Diagnosis | ICD-10-CM |
| O23.519 | Infections of cervix in pregnancy, unspecified trimester | Diagnosis | ICD-10-CM |
| O23.521 | Salpingo-oophoritis in pregnancy, first trimester | Diagnosis | ICD-10-CM |
| O23.522 | Salpingo-oophoritis in pregnancy, second trimester | Diagnosis | ICD-10-CM |
| O23.523 | Salpingo-oophoritis in pregnancy, third trimester | Diagnosis | ICD-10-CM |
| O23.529 | Salpingo-oophoritis in pregnancy, unspecified trimester | Diagnosis | ICD-10-CM |
| O23.591 | Infection of other part of genital tract in pregnancy, first trimester | Diagnosis | ICD-10-CM |
| O23.592 | Infection of other part of genital tract in pregnancy, second trimester | Diagnosis | ICD-10-CM |
| O23.593 | Infection of other part of genital tract in pregnancy, third trimester | Diagnosis | ICD-10-CM |
| O23.599 | Infection of other part of genital tract in pregnancy, unspecified trimester | Diagnosis | ICD-10-CM |
| O23.90 | Unspecified genitourinary tract infection in pregnancy, unspecified trimester | Diagnosis | ICD-10-CM |
| O23.91 | Unspecified genitourinary tract infection in pregnancy, first trimester | Diagnosis | ICD-10-CM |
| O23.92 | Unspecified genitourinary tract infection in pregnancy, second trimester | Diagnosis | ICD-10-CM |
| O23.93 | Unspecified genitourinary tract infection in pregnancy, third trimester | Diagnosis | ICD-10-CM |
| O24.011 | Pre-existing type 1 diabetes mellitus, in pregnancy, first trimester | Diagnosis | ICD-10-CM |
| O24.012 | Pre-existing type 1 diabetes mellitus, in pregnancy, second trimester | Diagnosis | ICD-10-CM |
| O24.013 | Pre-existing type 1 diabetes mellitus, in pregnancy, third trimester | Diagnosis | ICD-10-CM |
| O24.019 | Pre-existing type 1 diabetes mellitus, in pregnancy, unspecified trimester | Diagnosis | ICD-10-CM |
| O24.02 | Pre-existing type 1 diabetes mellitus, in childbirth | Diagnosis | ICD-10-CM |
| O24.03 | Pre-existing type 1 diabetes mellitus, in the puerperium | Diagnosis | ICD-10-CM |
| O24.111 | Pre-existing type 2 diabetes mellitus, in pregnancy, first trimester | Diagnosis | ICD-10-CM |
| O24.112 | Pre-existing type 2 diabetes mellitus, in pregnancy, second trimester | Diagnosis | ICD-10-CM |
| O24.113 | Pre-existing type 2 diabetes mellitus, in pregnancy, third trimester | Diagnosis | ICD-10-CM |
| O24.119 | Pre-existing type 2 diabetes mellitus, in pregnancy, unspecified trimester | Diagnosis | ICD-10-CM |
| O24.12 | Pre-existing type 2 diabetes mellitus, in childbirth | Diagnosis | ICD-10-CM |
| O24.13 | Pre-existing type 2 diabetes mellitus, in the puerperium | Diagnosis | ICD-10-CM |
| O24.311 | Unspecified pre-existing diabetes mellitus in pregnancy, first trimester | Diagnosis | ICD-10-CM |
| O24.312 | Unspecified pre-existing diabetes mellitus in pregnancy, second trimester | Diagnosis | ICD-10-CM |
| O24.313 | Unspecified pre-existing diabetes mellitus in pregnancy, third trimester | Diagnosis | ICD-10-CM |
| O24.319 | Unspecified pre-existing diabetes mellitus in pregnancy, unspecified trimester | Diagnosis | ICD-10-CM |
| O24.32 | Unspecified pre-existing diabetes mellitus in childbirth | Diagnosis | ICD-10-CM |
| O24.33 | Unspecified pre-existing diabetes mellitus in the puerperium | Diagnosis | ICD-10-CM |
| O24.410 | Gestational diabetes mellitus in pregnancy, diet controlled | Diagnosis | ICD-10-CM |
| O24.414 | Gestational diabetes mellitus in pregnancy, insulin controlled | Diagnosis | ICD-10-CM |
| O24.415 | Gestational diabetes mellitus in pregnancy, controlled by oral hypoglycemic drugs | Diagnosis | ICD-10-CM |
| O24.419 | Gestational diabetes mellitus in pregnancy, unspecified control | Diagnosis | ICD-10-CM |
| O24.420 | Gestational diabetes mellitus in childbirth, diet controlled | Diagnosis | ICD-10-CM |
| O24.424 | Gestational diabetes mellitus in childbirth, insulin controlled | Diagnosis | ICD-10-CM |
| O24.425 | Gestational diabetes mellitus in childbirth, controlled by oral hypoglycemic drugs | Diagnosis | ICD-10-CM |
| O24.429 | Gestational diabetes mellitus in childbirth, unspecified control | Diagnosis | ICD-10-CM |
| O24.430 | Gestational diabetes mellitus in the puerperium, diet controlled | Diagnosis | ICD-10-CM |
| O24.434 | Gestational diabetes mellitus in the puerperium, insulin controlled | Diagnosis | ICD-10-CM |
| O24.435 | Gestational diabetes mellitus in puerperium, controlled by oral hypoglycemic drugs | Diagnosis | ICD-10-CM |
| O24.439 | Gestational diabetes mellitus in the puerperium, unspecified control | Diagnosis | ICD-10-CM |
| O24.811 | Other pre-existing diabetes mellitus in pregnancy, first trimester | Diagnosis | ICD-10-CM |
| O24.812 | Other pre-existing diabetes mellitus in pregnancy, second trimester | Diagnosis | ICD-10-CM |
| O24.813 | Other pre-existing diabetes mellitus in pregnancy, third trimester | Diagnosis | ICD-10-CM |
| O24.819 | Other pre-existing diabetes mellitus in pregnancy, unspecified trimester | Diagnosis | ICD-10-CM |
| O24.82 | Other pre-existing diabetes mellitus in childbirth | Diagnosis | ICD-10-CM |
| O24.83 | Other pre-existing diabetes mellitus in the puerperium | Diagnosis | ICD-10-CM |
| O24.911 | Unspecified diabetes mellitus in pregnancy, first trimester | Diagnosis | ICD-10-CM |
| O24.912 | Unspecified diabetes mellitus in pregnancy, second trimester | Diagnosis | ICD-10-CM |
| O24.913 | Unspecified diabetes mellitus in pregnancy, third trimester | Diagnosis | ICD-10-CM |
| O24.919 | Unspecified diabetes mellitus in pregnancy, unspecified trimester | Diagnosis | ICD-10-CM |
| O24.92 | Unspecified diabetes mellitus in childbirth | Diagnosis | ICD-10-CM |
| O24.93 | Unspecified diabetes mellitus in the puerperium | Diagnosis | ICD-10-CM |
| O25.10 | Malnutrition in pregnancy, unspecified trimester | Diagnosis | ICD-10-CM |
| O25.11 | Malnutrition in pregnancy, first trimester | Diagnosis | ICD-10-CM |
| O25.12 | Malnutrition in pregnancy, second trimester | Diagnosis | ICD-10-CM |
| O25.13 | Malnutrition in pregnancy, third trimester | Diagnosis | ICD-10-CM |
| O25.2 | Malnutrition in childbirth | Diagnosis | ICD-10-CM |
| O25.3 | Malnutrition in the puerperium | Diagnosis | ICD-10-CM |
| O26.00 | Excessive weight gain in pregnancy, unspecified trimester | Diagnosis | ICD-10-CM |
| O26.01 | Excessive weight gain in pregnancy, first trimester | Diagnosis | ICD-10-CM |
| O26.02 | Excessive weight gain in pregnancy, second trimester | Diagnosis | ICD-10-CM |
| O26.03 | Excessive weight gain in pregnancy, third trimester | Diagnosis | ICD-10-CM |
| O26.10 | Low weight gain in pregnancy, unspecified trimester | Diagnosis | ICD-10-CM |
| O26.11 | Low weight gain in pregnancy, first trimester | Diagnosis | ICD-10-CM |
| O26.12 | Low weight gain in pregnancy, second trimester | Diagnosis | ICD-10-CM |
| O26.13 | Low weight gain in pregnancy, third trimester | Diagnosis | ICD-10-CM |
| O26.20 | Pregnancy care for patient with recurrent pregnancy loss, unspecified trimester | Diagnosis | ICD-10-CM |
| O26.21 | Pregnancy care for patient with recurrent pregnancy loss, first trimester | Diagnosis | ICD-10-CM |
| O26.22 | Pregnancy care for patient with recurrent pregnancy loss, second trimester | Diagnosis | ICD-10-CM |
| O26.23 | Pregnancy care for patient with recurrent pregnancy loss, third trimester | Diagnosis | ICD-10-CM |
| O26.30 | Retained intrauterine contraceptive device in pregnancy, unspecified trimester | Diagnosis | ICD-10-CM |
| O26.31 | Retained intrauterine contraceptive device in pregnancy, first trimester | Diagnosis | ICD-10-CM |
| O26.32 | Retained intrauterine contraceptive device in pregnancy, second trimester | Diagnosis | ICD-10-CM |
| O26.33 | Retained intrauterine contraceptive device in pregnancy, third trimester | Diagnosis | ICD-10-CM |
| O26.40 | Herpes gestationis, unspecified trimester | Diagnosis | ICD-10-CM |
| O26.41 | Herpes gestationis, first trimester | Diagnosis | ICD-10-CM |
| O26.42 | Herpes gestationis, second trimester | Diagnosis | ICD-10-CM |
| O26.43 | Herpes gestationis, third trimester | Diagnosis | ICD-10-CM |
| O26.50 | Maternal hypotension syndrome, unspecified trimester | Diagnosis | ICD-10-CM |
| O26.51 | Maternal hypotension syndrome, first trimester | Diagnosis | ICD-10-CM |
| O26.52 | Maternal hypotension syndrome, second trimester | Diagnosis | ICD-10-CM |
| O26.53 | Maternal hypotension syndrome, third trimester | Diagnosis | ICD-10-CM |
| O26.611 | Liver and biliary tract disorders in pregnancy, first trimester | Diagnosis | ICD-10-CM |
| O26.612 | Liver and biliary tract disorders in pregnancy, second trimester | Diagnosis | ICD-10-CM |
| O26.613 | Liver and biliary tract disorders in pregnancy, third trimester | Diagnosis | ICD-10-CM |
| O26.619 | Liver and biliary tract disorders in pregnancy, unspecified trimester | Diagnosis | ICD-10-CM |
| O26.62 | Liver and biliary tract disorders in childbirth | Diagnosis | ICD-10-CM |
| O26.63 | Liver and biliary tract disorders in the puerperium | Diagnosis | ICD-10-CM |
| O26.711 | Subluxation of symphysis (pubis) in pregnancy, first trimester | Diagnosis | ICD-10-CM |
| O26.712 | Subluxation of symphysis (pubis) in pregnancy, second trimester | Diagnosis | ICD-10-CM |
| O26.713 | Subluxation of symphysis (pubis) in pregnancy, third trimester | Diagnosis | ICD-10-CM |
| O26.719 | Subluxation of symphysis (pubis) in pregnancy, unspecified trimester | Diagnosis | ICD-10-CM |
| O26.72 | Subluxation of symphysis (pubis) in childbirth | Diagnosis | ICD-10-CM |
| O26.73 | Subluxation of symphysis (pubis) in the puerperium | Diagnosis | ICD-10-CM |
| O26.811 | Pregnancy related exhaustion and fatigue, first trimester | Diagnosis | ICD-10-CM |
| O26.812 | Pregnancy related exhaustion and fatigue, second trimester | Diagnosis | ICD-10-CM |
| O26.813 | Pregnancy related exhaustion and fatigue, third trimester | Diagnosis | ICD-10-CM |
| O26.819 | Pregnancy related exhaustion and fatigue, unspecified trimester | Diagnosis | ICD-10-CM |
| O26.821 | Pregnancy related peripheral neuritis, first trimester | Diagnosis | ICD-10-CM |
| O26.822 | Pregnancy related peripheral neuritis, second trimester | Diagnosis | ICD-10-CM |
| O26.823 | Pregnancy related peripheral neuritis, third trimester | Diagnosis | ICD-10-CM |
| O26.829 | Pregnancy related peripheral neuritis, unspecified trimester | Diagnosis | ICD-10-CM |
| O26.831 | Pregnancy related renal disease, first trimester | Diagnosis | ICD-10-CM |
| O26.832 | Pregnancy related renal disease, second trimester | Diagnosis | ICD-10-CM |
| O26.833 | Pregnancy related renal disease, third trimester | Diagnosis | ICD-10-CM |
| O26.839 | Pregnancy related renal disease, unspecified trimester | Diagnosis | ICD-10-CM |
| O26.841 | Uterine size-date discrepancy, first trimester | Diagnosis | ICD-10-CM |
| O26.842 | Uterine size-date discrepancy, second trimester | Diagnosis | ICD-10-CM |
| O26.843 | Uterine size-date discrepancy, third trimester | Diagnosis | ICD-10-CM |
| O26.849 | Uterine size-date discrepancy, unspecified trimester | Diagnosis | ICD-10-CM |
| O26.851 | Spotting complicating pregnancy, first trimester | Diagnosis | ICD-10-CM |
| O26.852 | Spotting complicating pregnancy, second trimester | Diagnosis | ICD-10-CM |
| O26.853 | Spotting complicating pregnancy, third trimester | Diagnosis | ICD-10-CM |
| O26.859 | Spotting complicating pregnancy, unspecified trimester | Diagnosis | ICD-10-CM |
| O26.86 | Pruritic urticarial papules and plaques of pregnancy (PUPPP) | Diagnosis | ICD-10-CM |
| O26.872 | Cervical shortening, second trimester | Diagnosis | ICD-10-CM |
| O26.873 | Cervical shortening, third trimester | Diagnosis | ICD-10-CM |
| O26.879 | Cervical shortening, unspecified trimester | Diagnosis | ICD-10-CM |
| O26.891 | Other specified pregnancy related conditions, first trimester | Diagnosis | ICD-10-CM |
| O26.892 | Other specified pregnancy related conditions, second trimester | Diagnosis | ICD-10-CM |
| O26.893 | Other specified pregnancy related conditions, third trimester | Diagnosis | ICD-10-CM |
| O26.899 | Other specified pregnancy related conditions, unspecified trimester | Diagnosis | ICD-10-CM |
| O26.90 | Pregnancy related conditions, unspecified, unspecified trimester | Diagnosis | ICD-10-CM |
| O26.91 | Pregnancy related conditions, unspecified, first trimester | Diagnosis | ICD-10-CM |
| O26.92 | Pregnancy related conditions, unspecified, second trimester | Diagnosis | ICD-10-CM |
| O26.93 | Pregnancy related conditions, unspecified, third trimester | Diagnosis | ICD-10-CM |
| O29.011 | Aspiration pneumonitis due to anesthesia during pregnancy, first trimester | Diagnosis | ICD-10-CM |
| O29.012 | Aspiration pneumonitis due to anesthesia during pregnancy, second trimester | Diagnosis | ICD-10-CM |
| O29.013 | Aspiration pneumonitis due to anesthesia during pregnancy, third trimester | Diagnosis | ICD-10-CM |
| O29.019 | Aspiration pneumonitis due to anesthesia during pregnancy, unspecified trimester | Diagnosis | ICD-10-CM |
| O29.021 | Pressure collapse of lung due to anesthesia during pregnancy, first trimester | Diagnosis | ICD-10-CM |
| O29.022 | Pressure collapse of lung due to anesthesia during pregnancy, second trimester | Diagnosis | ICD-10-CM |
| O29.023 | Pressure collapse of lung due to anesthesia during pregnancy, third trimester | Diagnosis | ICD-10-CM |
| O29.029 | Pressure collapse of lung due to anesthesia during pregnancy, unspecified trimester | Diagnosis | ICD-10-CM |
| O29.091 | Other pulmonary complications of anesthesia during pregnancy, first trimester | Diagnosis | ICD-10-CM |
| O29.092 | Other pulmonary complications of anesthesia during pregnancy, second trimester | Diagnosis | ICD-10-CM |
| O29.093 | Other pulmonary complications of anesthesia during pregnancy, third trimester | Diagnosis | ICD-10-CM |
| O29.099 | Other pulmonary complications of anesthesia during pregnancy, unspecified trimester | Diagnosis | ICD-10-CM |
| O29.111 | Cardiac arrest due to anesthesia during pregnancy, first trimester | Diagnosis | ICD-10-CM |
| O29.112 | Cardiac arrest due to anesthesia during pregnancy, second trimester | Diagnosis | ICD-10-CM |
| O29.113 | Cardiac arrest due to anesthesia during pregnancy, third trimester | Diagnosis | ICD-10-CM |
| O29.119 | Cardiac arrest due to anesthesia during pregnancy, unspecified trimester | Diagnosis | ICD-10-CM |
| O29.121 | Cardiac failure due to anesthesia during pregnancy, first trimester | Diagnosis | ICD-10-CM |
| O29.122 | Cardiac failure due to anesthesia during pregnancy, second trimester | Diagnosis | ICD-10-CM |
| O29.123 | Cardiac failure due to anesthesia during pregnancy, third trimester | Diagnosis | ICD-10-CM |
| O29.129 | Cardiac failure due to anesthesia during pregnancy, unspecified trimester | Diagnosis | ICD-10-CM |
| O29.191 | Other cardiac complications of anesthesia during pregnancy, first trimester | Diagnosis | ICD-10-CM |
| O29.192 | Other cardiac complications of anesthesia during pregnancy, second trimester | Diagnosis | ICD-10-CM |
| O29.193 | Other cardiac complications of anesthesia during pregnancy, third trimester | Diagnosis | ICD-10-CM |
| O29.199 | Other cardiac complications of anesthesia during pregnancy, unspecified trimester | Diagnosis | ICD-10-CM |
| O29.211 | Cerebral anoxia due to anesthesia during pregnancy, first trimester | Diagnosis | ICD-10-CM |
| O29.212 | Cerebral anoxia due to anesthesia during pregnancy, second trimester | Diagnosis | ICD-10-CM |
| O29.213 | Cerebral anoxia due to anesthesia during pregnancy, third trimester | Diagnosis | ICD-10-CM |
| O29.219 | Cerebral anoxia due to anesthesia during pregnancy, unspecified trimester | Diagnosis | ICD-10-CM |
| O29.291 | Other central nervous system complications of anesthesia during pregnancy, first trimester | Diagnosis | ICD-10-CM |
| O29.292 | Other central nervous system complications of anesthesia during pregnancy, second trimester | Diagnosis | ICD-10-CM |
| O29.293 | Other central nervous system complications of anesthesia during pregnancy, third trimester | Diagnosis | ICD-10-CM |
| O29.299 | Other central nervous system complications of anesthesia during pregnancy, unspecified trimester | Diagnosis | ICD-10-CM |
| O29.3X1 | Toxic reaction to local anesthesia during pregnancy, first trimester | Diagnosis | ICD-10-CM |
| O29.3X2 | Toxic reaction to local anesthesia during pregnancy, second trimester | Diagnosis | ICD-10-CM |
| O29.3X3 | Toxic reaction to local anesthesia during pregnancy, third trimester | Diagnosis | ICD-10-CM |
| O29.3X9 | Toxic reaction to local anesthesia during pregnancy, unspecified trimester | Diagnosis | ICD-10-CM |
| O29.40 | Spinal and epidural anesthesia induced headache during pregnancy, unspecified trimester | Diagnosis | ICD-10-CM |
| O29.41 | Spinal and epidural anesthesia induced headache during pregnancy, first trimester | Diagnosis | ICD-10-CM |
| O29.42 | Spinal and epidural anesthesia induced headache during pregnancy, second trimester | Diagnosis | ICD-10-CM |
| O29.43 | Spinal and epidural anesthesia induced headache during pregnancy, third trimester | Diagnosis | ICD-10-CM |
| O29.5X1 | Other complications of spinal and epidural anesthesia during pregnancy, first trimester | Diagnosis | ICD-10-CM |
| O29.5X2 | Other complications of spinal and epidural anesthesia during pregnancy, second trimester | Diagnosis | ICD-10-CM |
| O29.5X3 | Other complications of spinal and epidural anesthesia during pregnancy, third trimester | Diagnosis | ICD-10-CM |
| O29.5X9 | Other complications of spinal and epidural anesthesia during pregnancy, unspecified trimester | Diagnosis | ICD-10-CM |
| O29.60 | Failed or difficult intubation for anesthesia during pregnancy, unspecified trimester | Diagnosis | ICD-10-CM |
| O29.61 | Failed or difficult intubation for anesthesia during pregnancy, first trimester | Diagnosis | ICD-10-CM |
| O29.62 | Failed or difficult intubation for anesthesia during pregnancy, second trimester | Diagnosis | ICD-10-CM |
| O29.63 | Failed or difficult intubation for anesthesia during pregnancy, third trimester | Diagnosis | ICD-10-CM |
| O29.8X1 | Other complications of anesthesia during pregnancy, first trimester | Diagnosis | ICD-10-CM |
| O29.8X2 | Other complications of anesthesia during pregnancy, second trimester | Diagnosis | ICD-10-CM |
| O29.8X3 | Other complications of anesthesia during pregnancy, third trimester | Diagnosis | ICD-10-CM |
| O29.8X9 | Other complications of anesthesia during pregnancy, unspecified trimester | Diagnosis | ICD-10-CM |
| O29.90 | Unspecified complication of anesthesia during pregnancy, unspecified trimester | Diagnosis | ICD-10-CM |
| O29.91 | Unspecified complication of anesthesia during pregnancy, first trimester | Diagnosis | ICD-10-CM |
| O29.92 | Unspecified complication of anesthesia during pregnancy, second trimester | Diagnosis | ICD-10-CM |
| O29.93 | Unspecified complication of anesthesia during pregnancy, third trimester | Diagnosis | ICD-10-CM |
| O30.001 | Twin pregnancy, unspecified number of placenta and unspecified number of amniotic sacs, first trimester | Diagnosis | ICD-10-CM |
| O30.002 | Twin pregnancy, unspecified number of placenta and unspecified number of amniotic sacs, second trimester | Diagnosis | ICD-10-CM |
| O30.003 | Twin pregnancy, unspecified number of placenta and unspecified number of amniotic sacs, third trimester | Diagnosis | ICD-10-CM |
| O30.009 | Twin pregnancy, unspecified number of placenta and unspecified number of amniotic sacs, unspecified trimester | Diagnosis | ICD-10-CM |
| O30.011 | Twin pregnancy, monochorionic/monoamniotic, first trimester | Diagnosis | ICD-10-CM |
| O30.012 | Twin pregnancy, monochorionic/monoamniotic, second trimester | Diagnosis | ICD-10-CM |
| O30.013 | Twin pregnancy, monochorionic/monoamniotic, third trimester | Diagnosis | ICD-10-CM |
| O30.019 | Twin pregnancy, monochorionic/monoamniotic, unspecified trimester | Diagnosis | ICD-10-CM |
| O30.021 | Conjoined twin pregnancy, first trimester | Diagnosis | ICD-10-CM |
| O30.022 | Conjoined twin pregnancy, second trimester | Diagnosis | ICD-10-CM |
| O30.023 | Conjoined twin pregnancy, third trimester | Diagnosis | ICD-10-CM |
| O30.029 | Conjoined twin pregnancy, unspecified trimester | Diagnosis | ICD-10-CM |
| O30.031 | Twin pregnancy, monochorionic/diamniotic, first trimester | Diagnosis | ICD-10-CM |
| O30.032 | Twin pregnancy, monochorionic/diamniotic, second trimester | Diagnosis | ICD-10-CM |
| O30.033 | Twin pregnancy, monochorionic/diamniotic, third trimester | Diagnosis | ICD-10-CM |
| O30.039 | Twin pregnancy, monochorionic/diamniotic, unspecified trimester | Diagnosis | ICD-10-CM |
| O30.041 | Twin pregnancy, dichorionic/diamniotic, first trimester | Diagnosis | ICD-10-CM |
| O30.042 | Twin pregnancy, dichorionic/diamniotic, second trimester | Diagnosis | ICD-10-CM |
| O30.043 | Twin pregnancy, dichorionic/diamniotic, third trimester | Diagnosis | ICD-10-CM |
| O30.049 | Twin pregnancy, dichorionic/diamniotic, unspecified trimester | Diagnosis | ICD-10-CM |
| O30.091 | Twin pregnancy, unable to determine number of placenta and number of amniotic sacs, first trimester | Diagnosis | ICD-10-CM |
| O30.092 | Twin pregnancy, unable to determine number of placenta and number of amniotic sacs, second trimester | Diagnosis | ICD-10-CM |
| O30.093 | Twin pregnancy, unable to determine number of placenta and number of amniotic sacs, third trimester | Diagnosis | ICD-10-CM |
| O30.099 | Twin pregnancy, unable to determine number of placenta and number of amniotic sacs, unspecified trimester | Diagnosis | ICD-10-CM |
| O30.101 | Triplet pregnancy, unspecified number of placenta and unspecified number of amniotic sacs, first trimester | Diagnosis | ICD-10-CM |
| O30.102 | Triplet pregnancy, unspecified number of placenta and unspecified number of amniotic sacs, second trimester | Diagnosis | ICD-10-CM |
| O30.103 | Triplet pregnancy, unspecified number of placenta and unspecified number of amniotic sacs, third trimester | Diagnosis | ICD-10-CM |
| O30.109 | Triplet pregnancy, unspecified number of placenta and unspecified number of amniotic sacs, unspecified trimester | Diagnosis | ICD-10-CM |
| O30.111 | Triplet pregnancy with two or more monochorionic fetuses, first trimester | Diagnosis | ICD-10-CM |
| O30.112 | Triplet pregnancy with two or more monochorionic fetuses, second trimester | Diagnosis | ICD-10-CM |
| O30.113 | Triplet pregnancy with two or more monochorionic fetuses, third trimester | Diagnosis | ICD-10-CM |
| O30.119 | Triplet pregnancy with two or more monochorionic fetuses, unspecified trimester | Diagnosis | ICD-10-CM |
| O30.121 | Triplet pregnancy with two or more monoamniotic fetuses, first trimester | Diagnosis | ICD-10-CM |
| O30.122 | Triplet pregnancy with two or more monoamniotic fetuses, second trimester | Diagnosis | ICD-10-CM |
| O30.123 | Triplet pregnancy with two or more monoamniotic fetuses, third trimester | Diagnosis | ICD-10-CM |
| O30.129 | Triplet pregnancy with two or more monoamniotic fetuses, unspecified trimester | Diagnosis | ICD-10-CM |
| O30.191 | Triplet pregnancy, unable to determine number of placenta and number of amniotic sacs, first trimester | Diagnosis | ICD-10-CM |
| O30.192 | Triplet pregnancy, unable to determine number of placenta and number of amniotic sacs, second trimester | Diagnosis | ICD-10-CM |
| O30.193 | Triplet pregnancy, unable to determine number of placenta and number of amniotic sacs, third trimester | Diagnosis | ICD-10-CM |
| O30.199 | Triplet pregnancy, unable to determine number of placenta and number of amniotic sacs, unspecified trimester | Diagnosis | ICD-10-CM |
| O30.201 | Quadruplet pregnancy, unspecified number of placenta and unspecified number of amniotic sacs, first trimester | Diagnosis | ICD-10-CM |
| O30.202 | Quadruplet pregnancy, unspecified number of placenta and unspecified number of amniotic sacs, second trimester | Diagnosis | ICD-10-CM |
| O30.203 | Quadruplet pregnancy, unspecified number of placenta and unspecified number of amniotic sacs, third trimester | Diagnosis | ICD-10-CM |
| O30.209 | Quadruplet pregnancy, unspecified number of placenta and unspecified number of amniotic sacs, unspecified trimester | Diagnosis | ICD-10-CM |
| O30.211 | Quadruplet pregnancy with two or more monochorionic fetuses, first trimester | Diagnosis | ICD-10-CM |
| O30.212 | Quadruplet pregnancy with two or more monochorionic fetuses, second trimester | Diagnosis | ICD-10-CM |
| O30.213 | Quadruplet pregnancy with two or more monochorionic fetuses, third trimester | Diagnosis | ICD-10-CM |
| O30.219 | Quadruplet pregnancy with two or more monochorionic fetuses, unspecified trimester | Diagnosis | ICD-10-CM |
| O30.221 | Quadruplet pregnancy with two or more monoamniotic fetuses, first trimester | Diagnosis | ICD-10-CM |
| O30.222 | Quadruplet pregnancy with two or more monoamniotic fetuses, second trimester | Diagnosis | ICD-10-CM |
| O30.223 | Quadruplet pregnancy with two or more monoamniotic fetuses, third trimester | Diagnosis | ICD-10-CM |
| O30.229 | Quadruplet pregnancy with two or more monoamniotic fetuses, unspecified trimester | Diagnosis | ICD-10-CM |
| O30.291 | Quadruplet pregnancy, unable to determine number of placenta and number of amniotic sacs, first trimester | Diagnosis | ICD-10-CM |
| O30.292 | Quadruplet pregnancy, unable to determine number of placenta and number of amniotic sacs, second trimester | Diagnosis | ICD-10-CM |
| O30.293 | Quadruplet pregnancy, unable to determine number of placenta and number of amniotic sacs, third trimester | Diagnosis | ICD-10-CM |
| O30.299 | Quadruplet pregnancy, unable to determine number of placenta and number of amniotic sacs, unspecified trimester | Diagnosis | ICD-10-CM |
| O30.801 | Other specified multiple gestation, unspecified number of placenta and unspecified number of amniotic sacs, first trimester | Diagnosis | ICD-10-CM |
| O30.802 | Other specified multiple gestation, unspecified number of placenta and unspecified number of amniotic sacs, second trimester | Diagnosis | ICD-10-CM |
| O30.803 | Other specified multiple gestation, unspecified number of placenta and unspecified number of amniotic sacs, third trimester | Diagnosis | ICD-10-CM |
| O30.809 | Other specified multiple gestation, unspecified number of placenta and unspecified number of amniotic sacs, unspecified trimester | Diagnosis | ICD-10-CM |
| O30.811 | Other specified multiple gestation with two or more monochorionic fetuses, first trimester | Diagnosis | ICD-10-CM |
| O30.812 | Other specified multiple gestation with two or more monochorionic fetuses, second trimester | Diagnosis | ICD-10-CM |
| O30.813 | Other specified multiple gestation with two or more monochorionic fetuses, third trimester | Diagnosis | ICD-10-CM |
| O30.819 | Other specified multiple gestation with two or more monochorionic fetuses, unspecified trimester | Diagnosis | ICD-10-CM |
| O30.821 | Other specified multiple gestation with two or more monoamniotic fetuses, first trimester | Diagnosis | ICD-10-CM |
| O30.822 | Other specified multiple gestation with two or more monoamniotic fetuses, second trimester | Diagnosis | ICD-10-CM |
| O30.823 | Other specified multiple gestation with two or more monoamniotic fetuses, third trimester | Diagnosis | ICD-10-CM |
| O30.829 | Other specified multiple gestation with two or more monoamniotic fetuses, unspecified trimester | Diagnosis | ICD-10-CM |
| O30.891 | Other specified multiple gestation, unable to determine number of placenta and number of amniotic sacs, first trimester | Diagnosis | ICD-10-CM |
| O30.892 | Other specified multiple gestation, unable to determine number of placenta and number of amniotic sacs, second trimester | Diagnosis | ICD-10-CM |
| O30.893 | Other specified multiple gestation, unable to determine number of placenta and number of amniotic sacs, third trimester | Diagnosis | ICD-10-CM |
| O30.899 | Other specified multiple gestation, unable to determine number of placenta and number of amniotic sacs, unspecified trimester | Diagnosis | ICD-10-CM |
| O30.90 | Multiple gestation, unspecified, unspecified trimester | Diagnosis | ICD-10-CM |
| O30.91 | Multiple gestation, unspecified, first trimester | Diagnosis | ICD-10-CM |
| O30.92 | Multiple gestation, unspecified, second trimester | Diagnosis | ICD-10-CM |
| O30.93 | Multiple gestation, unspecified, third trimester | Diagnosis | ICD-10-CM |
| O31.00X0 | Papyraceous fetus, unspecified trimester, not applicable or unspecified | Diagnosis | ICD-10-CM |
| O31.00X1 | Papyraceous fetus, unspecified trimester, fetus 1 | Diagnosis | ICD-10-CM |
| O31.00X2 | Papyraceous fetus, unspecified trimester, fetus 2 | Diagnosis | ICD-10-CM |
| O31.00X3 | Papyraceous fetus, unspecified trimester, fetus 3 | Diagnosis | ICD-10-CM |
| O31.00X4 | Papyraceous fetus, unspecified trimester, fetus 4 | Diagnosis | ICD-10-CM |
| O31.00X5 | Papyraceous fetus, unspecified trimester, fetus 5 | Diagnosis | ICD-10-CM |
| O31.00X9 | Papyraceous fetus, unspecified trimester, other fetus | Diagnosis | ICD-10-CM |
| O31.01X0 | Papyraceous fetus, first trimester, not applicable or unspecified | Diagnosis | ICD-10-CM |
| O31.01X1 | Papyraceous fetus, first trimester, fetus 1 | Diagnosis | ICD-10-CM |
| O31.01X2 | Papyraceous fetus, first trimester, fetus 2 | Diagnosis | ICD-10-CM |
| O31.01X3 | Papyraceous fetus, first trimester, fetus 3 | Diagnosis | ICD-10-CM |
| O31.01X4 | Papyraceous fetus, first trimester, fetus 4 | Diagnosis | ICD-10-CM |
| O31.01X5 | Papyraceous fetus, first trimester, fetus 5 | Diagnosis | ICD-10-CM |
| O31.01X9 | Papyraceous fetus, first trimester, other fetus | Diagnosis | ICD-10-CM |
| O31.02X0 | Papyraceous fetus, second trimester, not applicable or unspecified | Diagnosis | ICD-10-CM |
| O31.02X1 | Papyraceous fetus, second trimester, fetus 1 | Diagnosis | ICD-10-CM |
| O31.02X2 | Papyraceous fetus, second trimester, fetus 2 | Diagnosis | ICD-10-CM |
| O31.02X3 | Papyraceous fetus, second trimester, fetus 3 | Diagnosis | ICD-10-CM |
| O31.02X4 | Papyraceous fetus, second trimester, fetus 4 | Diagnosis | ICD-10-CM |
| O31.02X5 | Papyraceous fetus, second trimester, fetus 5 | Diagnosis | ICD-10-CM |
| O31.02X9 | Papyraceous fetus, second trimester, other fetus | Diagnosis | ICD-10-CM |
| O31.03X0 | Papyraceous fetus, third trimester, not applicable or unspecified | Diagnosis | ICD-10-CM |
| O31.03X1 | Papyraceous fetus, third trimester, fetus 1 | Diagnosis | ICD-10-CM |
| O31.03X2 | Papyraceous fetus, third trimester, fetus 2 | Diagnosis | ICD-10-CM |
| O31.03X3 | Papyraceous fetus, third trimester, fetus 3 | Diagnosis | ICD-10-CM |
| O31.03X4 | Papyraceous fetus, third trimester, fetus 4 | Diagnosis | ICD-10-CM |
| O31.03X5 | Papyraceous fetus, third trimester, fetus 5 | Diagnosis | ICD-10-CM |
| O31.03X9 | Papyraceous fetus, third trimester, other fetus | Diagnosis | ICD-10-CM |
| O31.10X0 | Continuing pregnancy after spontaneous abortion of one fetus or more, unspecified trimester, not applicable or unspecified | Diagnosis | ICD-10-CM |
| O31.10X1 | Continuing pregnancy after spontaneous abortion of one fetus or more, unspecified trimester, fetus 1 | Diagnosis | ICD-10-CM |
| O31.10X2 | Continuing pregnancy after spontaneous abortion of one fetus or more, unspecified trimester, fetus 2 | Diagnosis | ICD-10-CM |
| O31.10X3 | Continuing pregnancy after spontaneous abortion of one fetus or more, unspecified trimester, fetus 3 | Diagnosis | ICD-10-CM |
| O31.10X4 | Continuing pregnancy after spontaneous abortion of one fetus or more, unspecified trimester, fetus 4 | Diagnosis | ICD-10-CM |
| O31.10X5 | Continuing pregnancy after spontaneous abortion of one fetus or more, unspecified trimester, fetus 5 | Diagnosis | ICD-10-CM |
| O31.10X9 | Continuing pregnancy after spontaneous abortion of one fetus or more, unspecified trimester, other fetus | Diagnosis | ICD-10-CM |
| O31.11X0 | Continuing pregnancy after spontaneous abortion of one fetus or more, first trimester, not applicable or unspecified | Diagnosis | ICD-10-CM |
| O31.11X1 | Continuing pregnancy after spontaneous abortion of one fetus or more, first trimester, fetus 1 | Diagnosis | ICD-10-CM |
| O31.11X2 | Continuing pregnancy after spontaneous abortion of one fetus or more, first trimester, fetus 2 | Diagnosis | ICD-10-CM |
| O31.11X3 | Continuing pregnancy after spontaneous abortion of one fetus or more, first trimester, fetus 3 | Diagnosis | ICD-10-CM |
| O31.11X4 | Continuing pregnancy after spontaneous abortion of one fetus or more, first trimester, fetus 4 | Diagnosis | ICD-10-CM |
| O31.11X5 | Continuing pregnancy after spontaneous abortion of one fetus or more, first trimester, fetus 5 | Diagnosis | ICD-10-CM |
| O31.11X9 | Continuing pregnancy after spontaneous abortion of one fetus or more, first trimester, other fetus | Diagnosis | ICD-10-CM |
| O31.12X0 | Continuing pregnancy after spontaneous abortion of one fetus or more, second trimester, not applicable or unspecified | Diagnosis | ICD-10-CM |
| O31.12X1 | Continuing pregnancy after spontaneous abortion of one fetus or more, second trimester, fetus 1 | Diagnosis | ICD-10-CM |
| O31.12X2 | Continuing pregnancy after spontaneous abortion of one fetus or more, second trimester, fetus 2 | Diagnosis | ICD-10-CM |
| O31.12X3 | Continuing pregnancy after spontaneous abortion of one fetus or more, second trimester, fetus 3 | Diagnosis | ICD-10-CM |
| O31.12X4 | Continuing pregnancy after spontaneous abortion of one fetus or more, second trimester, fetus 4 | Diagnosis | ICD-10-CM |
| O31.12X5 | Continuing pregnancy after spontaneous abortion of one fetus or more, second trimester, fetus 5 | Diagnosis | ICD-10-CM |
| O31.12X9 | Continuing pregnancy after spontaneous abortion of one fetus or more, second trimester, other fetus | Diagnosis | ICD-10-CM |
| O31.13X0 | Continuing pregnancy after spontaneous abortion of one fetus or more, third trimester, not applicable or unspecified | Diagnosis | ICD-10-CM |
| O31.13X1 | Continuing pregnancy after spontaneous abortion of one fetus or more, third trimester, fetus 1 | Diagnosis | ICD-10-CM |
| O31.13X2 | Continuing pregnancy after spontaneous abortion of one fetus or more, third trimester, fetus 2 | Diagnosis | ICD-10-CM |
| O31.13X3 | Continuing pregnancy after spontaneous abortion of one fetus or more, third trimester, fetus 3 | Diagnosis | ICD-10-CM |
| O31.13X4 | Continuing pregnancy after spontaneous abortion of one fetus or more, third trimester, fetus 4 | Diagnosis | ICD-10-CM |
| O31.13X5 | Continuing pregnancy after spontaneous abortion of one fetus or more, third trimester, fetus 5 | Diagnosis | ICD-10-CM |
| O31.13X9 | Continuing pregnancy after spontaneous abortion of one fetus or more, third trimester, other fetus | Diagnosis | ICD-10-CM |
| O31.20X0 | Continuing pregnancy after intrauterine death of one fetus or more, unspecified trimester, not applicable or unspecified | Diagnosis | ICD-10-CM |
| O31.20X1 | Continuing pregnancy after intrauterine death of one fetus or more, unspecified trimester, fetus 1 | Diagnosis | ICD-10-CM |
| O31.20X2 | Continuing pregnancy after intrauterine death of one fetus or more, unspecified trimester, fetus 2 | Diagnosis | ICD-10-CM |
| O31.20X3 | Continuing pregnancy after intrauterine death of one fetus or more, unspecified trimester, fetus 3 | Diagnosis | ICD-10-CM |
| O31.20X4 | Continuing pregnancy after intrauterine death of one fetus or more, unspecified trimester, fetus 4 | Diagnosis | ICD-10-CM |
| O31.20X5 | Continuing pregnancy after intrauterine death of one fetus or more, unspecified trimester, fetus 5 | Diagnosis | ICD-10-CM |
| O31.20X9 | Continuing pregnancy after intrauterine death of one fetus or more, unspecified trimester, other fetus | Diagnosis | ICD-10-CM |
| O31.21X0 | Continuing pregnancy after intrauterine death of one fetus or more, first trimester, not applicable or unspecified | Diagnosis | ICD-10-CM |
| O31.21X1 | Continuing pregnancy after intrauterine death of one fetus or more, first trimester, fetus 1 | Diagnosis | ICD-10-CM |
| O31.21X2 | Continuing pregnancy after intrauterine death of one fetus or more, first trimester, fetus 2 | Diagnosis | ICD-10-CM |
| O31.21X3 | Continuing pregnancy after intrauterine death of one fetus or more, first trimester, fetus 3 | Diagnosis | ICD-10-CM |
| O31.21X4 | Continuing pregnancy after intrauterine death of one fetus or more, first trimester, fetus 4 | Diagnosis | ICD-10-CM |
| O31.21X5 | Continuing pregnancy after intrauterine death of one fetus or more, first trimester, fetus 5 | Diagnosis | ICD-10-CM |
| O31.21X9 | Continuing pregnancy after intrauterine death of one fetus or more, first trimester, other fetus | Diagnosis | ICD-10-CM |
| O31.22X0 | Continuing pregnancy after intrauterine death of one fetus or more, second trimester, not applicable or unspecified | Diagnosis | ICD-10-CM |
| O31.22X1 | Continuing pregnancy after intrauterine death of one fetus or more, second trimester, fetus 1 | Diagnosis | ICD-10-CM |
| O31.22X2 | Continuing pregnancy after intrauterine death of one fetus or more, second trimester, fetus 2 | Diagnosis | ICD-10-CM |
| O31.22X3 | Continuing pregnancy after intrauterine death of one fetus or more, second trimester, fetus 3 | Diagnosis | ICD-10-CM |
| O31.22X4 | Continuing pregnancy after intrauterine death of one fetus or more, second trimester, fetus 4 | Diagnosis | ICD-10-CM |
| O31.22X5 | Continuing pregnancy after intrauterine death of one fetus or more, second trimester, fetus 5 | Diagnosis | ICD-10-CM |
| O31.22X9 | Continuing pregnancy after intrauterine death of one fetus or more, second trimester, other fetus | Diagnosis | ICD-10-CM |
| O31.23X0 | Continuing pregnancy after intrauterine death of one fetus or more, third trimester, not applicable or unspecified | Diagnosis | ICD-10-CM |
| O31.23X1 | Continuing pregnancy after intrauterine death of one fetus or more, third trimester, fetus 1 | Diagnosis | ICD-10-CM |
| O31.23X2 | Continuing pregnancy after intrauterine death of one fetus or more, third trimester, fetus 2 | Diagnosis | ICD-10-CM |
| O31.23X3 | Continuing pregnancy after intrauterine death of one fetus or more, third trimester, fetus 3 | Diagnosis | ICD-10-CM |
| O31.23X4 | Continuing pregnancy after intrauterine death of one fetus or more, third trimester, fetus 4 | Diagnosis | ICD-10-CM |
| O31.23X5 | Continuing pregnancy after intrauterine death of one fetus or more, third trimester, fetus 5 | Diagnosis | ICD-10-CM |
| O31.23X9 | Continuing pregnancy after intrauterine death of one fetus or more, third trimester, other fetus | Diagnosis | ICD-10-CM |
| O31.30X0 | Continuing pregnancy after elective fetal reduction of one fetus or more, unspecified trimester, not applicable or unspecified | Diagnosis | ICD-10-CM |
| O31.30X1 | Continuing pregnancy after elective fetal reduction of one fetus or more, unspecified trimester, fetus 1 | Diagnosis | ICD-10-CM |
| O31.30X2 | Continuing pregnancy after elective fetal reduction of one fetus or more, unspecified trimester, fetus 2 | Diagnosis | ICD-10-CM |
| O31.30X3 | Continuing pregnancy after elective fetal reduction of one fetus or more, unspecified trimester, fetus 3 | Diagnosis | ICD-10-CM |
| O31.30X4 | Continuing pregnancy after elective fetal reduction of one fetus or more, unspecified trimester, fetus 4 | Diagnosis | ICD-10-CM |
| O31.30X5 | Continuing pregnancy after elective fetal reduction of one fetus or more, unspecified trimester, fetus 5 | Diagnosis | ICD-10-CM |
| O31.30X9 | Continuing pregnancy after elective fetal reduction of one fetus or more, unspecified trimester, other fetus | Diagnosis | ICD-10-CM |
| O31.31X0 | Continuing pregnancy after elective fetal reduction of one fetus or more, first trimester, not applicable or unspecified | Diagnosis | ICD-10-CM |
| O31.31X1 | Continuing pregnancy after elective fetal reduction of one fetus or more, first trimester, fetus 1 | Diagnosis | ICD-10-CM |
| O31.31X2 | Continuing pregnancy after elective fetal reduction of one fetus or more, first trimester, fetus 2 | Diagnosis | ICD-10-CM |
| O31.31X3 | Continuing pregnancy after elective fetal reduction of one fetus or more, first trimester, fetus 3 | Diagnosis | ICD-10-CM |
| O31.31X4 | Continuing pregnancy after elective fetal reduction of one fetus or more, first trimester, fetus 4 | Diagnosis | ICD-10-CM |
| O31.31X5 | Continuing pregnancy after elective fetal reduction of one fetus or more, first trimester, fetus 5 | Diagnosis | ICD-10-CM |
| O31.31X9 | Continuing pregnancy after elective fetal reduction of one fetus or more, first trimester, other fetus | Diagnosis | ICD-10-CM |
| O31.32X0 | Continuing pregnancy after elective fetal reduction of one fetus or more, second trimester, not applicable or unspecified | Diagnosis | ICD-10-CM |
| O31.32X1 | Continuing pregnancy after elective fetal reduction of one fetus or more, second trimester, fetus 1 | Diagnosis | ICD-10-CM |
| O31.32X2 | Continuing pregnancy after elective fetal reduction of one fetus or more, second trimester, fetus 2 | Diagnosis | ICD-10-CM |
| O31.32X3 | Continuing pregnancy after elective fetal reduction of one fetus or more, second trimester, fetus 3 | Diagnosis | ICD-10-CM |
| O31.32X4 | Continuing pregnancy after elective fetal reduction of one fetus or more, second trimester, fetus 4 | Diagnosis | ICD-10-CM |
| O31.32X5 | Continuing pregnancy after elective fetal reduction of one fetus or more, second trimester, fetus 5 | Diagnosis | ICD-10-CM |
| O31.32X9 | Continuing pregnancy after elective fetal reduction of one fetus or more, second trimester, other fetus | Diagnosis | ICD-10-CM |
| O31.33X0 | Continuing pregnancy after elective fetal reduction of one fetus or more, third trimester, not applicable or unspecified | Diagnosis | ICD-10-CM |
| O31.33X1 | Continuing pregnancy after elective fetal reduction of one fetus or more, third trimester, fetus 1 | Diagnosis | ICD-10-CM |
| O31.33X2 | Continuing pregnancy after elective fetal reduction of one fetus or more, third trimester, fetus 2 | Diagnosis | ICD-10-CM |
| O31.33X3 | Continuing pregnancy after elective fetal reduction of one fetus or more, third trimester, fetus 3 | Diagnosis | ICD-10-CM |
| O31.33X4 | Continuing pregnancy after elective fetal reduction of one fetus or more, third trimester, fetus 4 | Diagnosis | ICD-10-CM |
| O31.33X5 | Continuing pregnancy after elective fetal reduction of one fetus or more, third trimester, fetus 5 | Diagnosis | ICD-10-CM |
| O31.33X9 | Continuing pregnancy after elective fetal reduction of one fetus or more, third trimester, other fetus | Diagnosis | ICD-10-CM |
| O31.8X10 | Other complications specific to multiple gestation, first trimester, not applicable or unspecified | Diagnosis | ICD-10-CM |
| O31.8X11 | Other complications specific to multiple gestation, first trimester, fetus 1 | Diagnosis | ICD-10-CM |
| O31.8X12 | Other complications specific to multiple gestation, first trimester, fetus 2 | Diagnosis | ICD-10-CM |
| O31.8X13 | Other complications specific to multiple gestation, first trimester, fetus 3 | Diagnosis | ICD-10-CM |
| O31.8X14 | Other complications specific to multiple gestation, first trimester, fetus 4 | Diagnosis | ICD-10-CM |
| O31.8X15 | Other complications specific to multiple gestation, first trimester, fetus 5 | Diagnosis | ICD-10-CM |
| O31.8X19 | Other complications specific to multiple gestation, first trimester, other fetus | Diagnosis | ICD-10-CM |
| O31.8X20 | Other complications specific to multiple gestation, second trimester, not applicable or unspecified | Diagnosis | ICD-10-CM |
| O31.8X21 | Other complications specific to multiple gestation, second trimester, fetus 1 | Diagnosis | ICD-10-CM |
| O31.8X22 | Other complications specific to multiple gestation, second trimester, fetus 2 | Diagnosis | ICD-10-CM |
| O31.8X23 | Other complications specific to multiple gestation, second trimester, fetus 3 | Diagnosis | ICD-10-CM |
| O31.8X24 | Other complications specific to multiple gestation, second trimester, fetus 4 | Diagnosis | ICD-10-CM |
| O31.8X25 | Other complications specific to multiple gestation, second trimester, fetus 5 | Diagnosis | ICD-10-CM |
| O31.8X29 | Other complications specific to multiple gestation, second trimester, other fetus | Diagnosis | ICD-10-CM |
| O31.8X30 | Other complications specific to multiple gestation, third trimester, not applicable or unspecified | Diagnosis | ICD-10-CM |
| O31.8X31 | Other complications specific to multiple gestation, third trimester, fetus 1 | Diagnosis | ICD-10-CM |
| O31.8X32 | Other complications specific to multiple gestation, third trimester, fetus 2 | Diagnosis | ICD-10-CM |
| O31.8X33 | Other complications specific to multiple gestation, third trimester, fetus 3 | Diagnosis | ICD-10-CM |
| O31.8X34 | Other complications specific to multiple gestation, third trimester, fetus 4 | Diagnosis | ICD-10-CM |
| O31.8X35 | Other complications specific to multiple gestation, third trimester, fetus 5 | Diagnosis | ICD-10-CM |
| O31.8X39 | Other complications specific to multiple gestation, third trimester, other fetus | Diagnosis | ICD-10-CM |
| O31.8X90 | Other complications specific to multiple gestation, unspecified trimester, not applicable or unspecified | Diagnosis | ICD-10-CM |
| O31.8X91 | Other complications specific to multiple gestation, unspecified trimester, fetus 1 | Diagnosis | ICD-10-CM |
| O31.8X92 | Other complications specific to multiple gestation, unspecified trimester, fetus 2 | Diagnosis | ICD-10-CM |
| O31.8X93 | Other complications specific to multiple gestation, unspecified trimester, fetus 3 | Diagnosis | ICD-10-CM |
| O31.8X94 | Other complications specific to multiple gestation, unspecified trimester, fetus 4 | Diagnosis | ICD-10-CM |
| O31.8X95 | Other complications specific to multiple gestation, unspecified trimester, fetus 5 | Diagnosis | ICD-10-CM |
| O31.8X99 | Other complications specific to multiple gestation, unspecified trimester, other fetus | Diagnosis | ICD-10-CM |
| O32.0XX0 | Maternal care for unstable lie, not applicable or unspecified | Diagnosis | ICD-10-CM |
| O32.0XX1 | Maternal care for unstable lie, fetus 1 | Diagnosis | ICD-10-CM |
| O32.0XX2 | Maternal care for unstable lie, fetus 2 | Diagnosis | ICD-10-CM |
| O32.0XX3 | Maternal care for unstable lie, fetus 3 | Diagnosis | ICD-10-CM |
| O32.0XX4 | Maternal care for unstable lie, fetus 4 | Diagnosis | ICD-10-CM |
| O32.0XX5 | Maternal care for unstable lie, fetus 5 | Diagnosis | ICD-10-CM |
| O32.0XX9 | Maternal care for unstable lie, other fetus | Diagnosis | ICD-10-CM |
| O32.1XX0 | Maternal care for breech presentation, not applicable or unspecified | Diagnosis | ICD-10-CM |
| O32.1XX1 | Maternal care for breech presentation, fetus 1 | Diagnosis | ICD-10-CM |
| O32.1XX2 | Maternal care for breech presentation, fetus 2 | Diagnosis | ICD-10-CM |
| O32.1XX3 | Maternal care for breech presentation, fetus 3 | Diagnosis | ICD-10-CM |
| O32.1XX4 | Maternal care for breech presentation, fetus 4 | Diagnosis | ICD-10-CM |
| O32.1XX5 | Maternal care for breech presentation, fetus 5 | Diagnosis | ICD-10-CM |
| O32.1XX9 | Maternal care for breech presentation, other fetus | Diagnosis | ICD-10-CM |
| O32.2XX0 | Maternal care for transverse and oblique lie, not applicable or unspecified | Diagnosis | ICD-10-CM |
| O32.2XX1 | Maternal care for transverse and oblique lie, fetus 1 | Diagnosis | ICD-10-CM |
| O32.2XX2 | Maternal care for transverse and oblique lie, fetus 2 | Diagnosis | ICD-10-CM |
| O32.2XX3 | Maternal care for transverse and oblique lie, fetus 3 | Diagnosis | ICD-10-CM |
| O32.2XX4 | Maternal care for transverse and oblique lie, fetus 4 | Diagnosis | ICD-10-CM |
| O32.2XX5 | Maternal care for transverse and oblique lie, fetus 5 | Diagnosis | ICD-10-CM |
| O32.2XX9 | Maternal care for transverse and oblique lie, other fetus | Diagnosis | ICD-10-CM |
| O32.3XX0 | Maternal care for face, brow and chin presentation, not applicable or unspecified | Diagnosis | ICD-10-CM |
| O32.3XX1 | Maternal care for face, brow and chin presentation, fetus 1 | Diagnosis | ICD-10-CM |
| O32.3XX2 | Maternal care for face, brow and chin presentation, fetus 2 | Diagnosis | ICD-10-CM |
| O32.3XX3 | Maternal care for face, brow and chin presentation, fetus 3 | Diagnosis | ICD-10-CM |
| O32.3XX4 | Maternal care for face, brow and chin presentation, fetus 4 | Diagnosis | ICD-10-CM |
| O32.3XX5 | Maternal care for face, brow and chin presentation, fetus 5 | Diagnosis | ICD-10-CM |
| O32.3XX9 | Maternal care for face, brow and chin presentation, other fetus | Diagnosis | ICD-10-CM |
| O32.4XX0 | Maternal care for high head at term, not applicable or unspecified | Diagnosis | ICD-10-CM |
| O32.4XX1 | Maternal care for high head at term, fetus 1 | Diagnosis | ICD-10-CM |
| O32.4XX2 | Maternal care for high head at term, fetus 2 | Diagnosis | ICD-10-CM |
| O32.4XX3 | Maternal care for high head at term, fetus 3 | Diagnosis | ICD-10-CM |
| O32.4XX4 | Maternal care for high head at term, fetus 4 | Diagnosis | ICD-10-CM |
| O32.4XX5 | Maternal care for high head at term, fetus 5 | Diagnosis | ICD-10-CM |
| O32.4XX9 | Maternal care for high head at term, other fetus | Diagnosis | ICD-10-CM |
| O32.6XX0 | Maternal care for compound presentation, not applicable or unspecified | Diagnosis | ICD-10-CM |
| O32.6XX1 | Maternal care for compound presentation, fetus 1 | Diagnosis | ICD-10-CM |
| O32.6XX2 | Maternal care for compound presentation, fetus 2 | Diagnosis | ICD-10-CM |
| O32.6XX3 | Maternal care for compound presentation, fetus 3 | Diagnosis | ICD-10-CM |
| O32.6XX4 | Maternal care for compound presentation, fetus 4 | Diagnosis | ICD-10-CM |
| O32.6XX5 | Maternal care for compound presentation, fetus 5 | Diagnosis | ICD-10-CM |
| O32.6XX9 | Maternal care for compound presentation, other fetus | Diagnosis | ICD-10-CM |
| O32.8XX0 | Maternal care for other malpresentation of fetus, not applicable or unspecified | Diagnosis | ICD-10-CM |
| O32.8XX1 | Maternal care for other malpresentation of fetus, fetus 1 | Diagnosis | ICD-10-CM |
| O32.8XX2 | Maternal care for other malpresentation of fetus, fetus 2 | Diagnosis | ICD-10-CM |
| O32.8XX3 | Maternal care for other malpresentation of fetus, fetus 3 | Diagnosis | ICD-10-CM |
| O32.8XX4 | Maternal care for other malpresentation of fetus, fetus 4 | Diagnosis | ICD-10-CM |
| O32.8XX5 | Maternal care for other malpresentation of fetus, fetus 5 | Diagnosis | ICD-10-CM |
| O32.8XX9 | Maternal care for other malpresentation of fetus, other fetus | Diagnosis | ICD-10-CM |
| O32.9XX0 | Maternal care for malpresentation of fetus, unspecified, not applicable or unspecified | Diagnosis | ICD-10-CM |
| O32.9XX1 | Maternal care for malpresentation of fetus, unspecified, fetus 1 | Diagnosis | ICD-10-CM |
| O32.9XX2 | Maternal care for malpresentation of fetus, unspecified, fetus 2 | Diagnosis | ICD-10-CM |
| O32.9XX3 | Maternal care for malpresentation of fetus, unspecified, fetus 3 | Diagnosis | ICD-10-CM |
| O32.9XX4 | Maternal care for malpresentation of fetus, unspecified, fetus 4 | Diagnosis | ICD-10-CM |
| O32.9XX5 | Maternal care for malpresentation of fetus, unspecified, fetus 5 | Diagnosis | ICD-10-CM |
| O32.9XX9 | Maternal care for malpresentation of fetus, unspecified, other fetus | Diagnosis | ICD-10-CM |
| O33.0 | Maternal care for disproportion due to deformity of maternal pelvic bones | Diagnosis | ICD-10-CM |
| O33.1 | Maternal care for disproportion due to generally contracted pelvis | Diagnosis | ICD-10-CM |
| O33.2 | Maternal care for disproportion due to inlet contraction of pelvis | Diagnosis | ICD-10-CM |
| O33.3XX0 | Maternal care for disproportion due to outlet contraction of pelvis, not applicable or unspecified | Diagnosis | ICD-10-CM |
| O33.3XX1 | Maternal care for disproportion due to outlet contraction of pelvis, fetus 1 | Diagnosis | ICD-10-CM |
| O33.3XX2 | Maternal care for disproportion due to outlet contraction of pelvis, fetus 2 | Diagnosis | ICD-10-CM |
| O33.3XX3 | Maternal care for disproportion due to outlet contraction of pelvis, fetus 3 | Diagnosis | ICD-10-CM |
| O33.3XX4 | Maternal care for disproportion due to outlet contraction of pelvis, fetus 4 | Diagnosis | ICD-10-CM |
| O33.3XX5 | Maternal care for disproportion due to outlet contraction of pelvis, fetus 5 | Diagnosis | ICD-10-CM |
| O33.3XX9 | Maternal care for disproportion due to outlet contraction of pelvis, other fetus | Diagnosis | ICD-10-CM |
| O33.4XX0 | Maternal care for disproportion of mixed maternal and fetal origin, not applicable or unspecified | Diagnosis | ICD-10-CM |
| O33.4XX1 | Maternal care for disproportion of mixed maternal and fetal origin, fetus 1 | Diagnosis | ICD-10-CM |
| O33.4XX2 | Maternal care for disproportion of mixed maternal and fetal origin, fetus 2 | Diagnosis | ICD-10-CM |
| O33.4XX3 | Maternal care for disproportion of mixed maternal and fetal origin, fetus 3 | Diagnosis | ICD-10-CM |
| O33.4XX4 | Maternal care for disproportion of mixed maternal and fetal origin, fetus 4 | Diagnosis | ICD-10-CM |
| O33.4XX5 | Maternal care for disproportion of mixed maternal and fetal origin, fetus 5 | Diagnosis | ICD-10-CM |
| O33.4XX9 | Maternal care for disproportion of mixed maternal and fetal origin, other fetus | Diagnosis | ICD-10-CM |
| O33.5XX0 | Maternal care for disproportion due to unusually large fetus, not applicable or unspecified | Diagnosis | ICD-10-CM |
| O33.5XX1 | Maternal care for disproportion due to unusually large fetus, fetus 1 | Diagnosis | ICD-10-CM |
| O33.5XX2 | Maternal care for disproportion due to unusually large fetus, fetus 2 | Diagnosis | ICD-10-CM |
| O33.5XX3 | Maternal care for disproportion due to unusually large fetus, fetus 3 | Diagnosis | ICD-10-CM |
| O33.5XX4 | Maternal care for disproportion due to unusually large fetus, fetus 4 | Diagnosis | ICD-10-CM |
| O33.5XX5 | Maternal care for disproportion due to unusually large fetus, fetus 5 | Diagnosis | ICD-10-CM |
| O33.5XX9 | Maternal care for disproportion due to unusually large fetus, other fetus | Diagnosis | ICD-10-CM |
| O33.6XX0 | Maternal care for disproportion due to hydrocephalic fetus, not applicable or unspecified | Diagnosis | ICD-10-CM |
| O33.6XX1 | Maternal care for disproportion due to hydrocephalic fetus, fetus 1 | Diagnosis | ICD-10-CM |
| O33.6XX2 | Maternal care for disproportion due to hydrocephalic fetus, fetus 2 | Diagnosis | ICD-10-CM |
| O33.6XX3 | Maternal care for disproportion due to hydrocephalic fetus, fetus 3 | Diagnosis | ICD-10-CM |
| O33.6XX4 | Maternal care for disproportion due to hydrocephalic fetus, fetus 4 | Diagnosis | ICD-10-CM |
| O33.6XX5 | Maternal care for disproportion due to hydrocephalic fetus, fetus 5 | Diagnosis | ICD-10-CM |
| O33.6XX9 | Maternal care for disproportion due to hydrocephalic fetus, other fetus | Diagnosis | ICD-10-CM |
| O33.7XX0 | Maternal care for disproportion due to other fetal deformities, not applicable or unspecified | Diagnosis | ICD-10-CM |
| O33.7XX1 | Maternal care for disproportion due to other fetal deformities, fetus 1 | Diagnosis | ICD-10-CM |
| O33.7XX2 | Maternal care for disproportion due to other fetal deformities, fetus 2 | Diagnosis | ICD-10-CM |
| O33.7XX3 | Maternal care for disproportion due to other fetal deformities, fetus 3 | Diagnosis | ICD-10-CM |
| O33.7XX4 | Maternal care for disproportion due to other fetal deformities, fetus 4 | Diagnosis | ICD-10-CM |
| O33.7XX5 | Maternal care for disproportion due to other fetal deformities, fetus 5 | Diagnosis | ICD-10-CM |
| O33.7XX9 | Maternal care for disproportion due to other fetal deformities, other fetus | Diagnosis | ICD-10-CM |
| O33.8 | Maternal care for disproportion of other origin | Diagnosis | ICD-10-CM |
| O33.9 | Maternal care for disproportion, unspecified | Diagnosis | ICD-10-CM |
| O34.00 | Maternal care for unspecified congenital malformation of uterus, unspecified trimester | Diagnosis | ICD-10-CM |
| O34.01 | Maternal care for unspecified congenital malformation of uterus, first trimester | Diagnosis | ICD-10-CM |
| O34.02 | Maternal care for unspecified congenital malformation of uterus, second trimester | Diagnosis | ICD-10-CM |
| O34.03 | Maternal care for unspecified congenital malformation of uterus, third trimester | Diagnosis | ICD-10-CM |
| O34.10 | Maternal care for benign tumor of corpus uteri, unspecified trimester | Diagnosis | ICD-10-CM |
| O34.11 | Maternal care for benign tumor of corpus uteri, first trimester | Diagnosis | ICD-10-CM |
| O34.12 | Maternal care for benign tumor of corpus uteri, second trimester | Diagnosis | ICD-10-CM |
| O34.13 | Maternal care for benign tumor of corpus uteri, third trimester | Diagnosis | ICD-10-CM |
| O34.211 | Maternal care for low transverse scar from previous cesarean delivery | Diagnosis | ICD-10-CM |
| O34.212 | Maternal care for vertical scar from previous cesarean delivery | Diagnosis | ICD-10-CM |
| O34.219 | Maternal care for unspecified type scar from previous cesarean delivery | Diagnosis | ICD-10-CM |
| O34.29 | Maternal care due to uterine scar from other previous surgery | Diagnosis | ICD-10-CM |
| O34.30 | Maternal care for cervical incompetence, unspecified trimester | Diagnosis | ICD-10-CM |
| O34.31 | Maternal care for cervical incompetence, first trimester | Diagnosis | ICD-10-CM |
| O34.32 | Maternal care for cervical incompetence, second trimester | Diagnosis | ICD-10-CM |
| O34.33 | Maternal care for cervical incompetence, third trimester | Diagnosis | ICD-10-CM |
| O34.40 | Maternal care for other abnormalities of cervix, unspecified trimester | Diagnosis | ICD-10-CM |
| O34.41 | Maternal care for other abnormalities of cervix, first trimester | Diagnosis | ICD-10-CM |
| O34.42 | Maternal care for other abnormalities of cervix, second trimester | Diagnosis | ICD-10-CM |
| O34.43 | Maternal care for other abnormalities of cervix, third trimester | Diagnosis | ICD-10-CM |
| O34.511 | Maternal care for incarceration of gravid uterus, first trimester | Diagnosis | ICD-10-CM |
| O34.512 | Maternal care for incarceration of gravid uterus, second trimester | Diagnosis | ICD-10-CM |
| O34.513 | Maternal care for incarceration of gravid uterus, third trimester | Diagnosis | ICD-10-CM |
| O34.519 | Maternal care for incarceration of gravid uterus, unspecified trimester | Diagnosis | ICD-10-CM |
| O34.521 | Maternal care for prolapse of gravid uterus, first trimester | Diagnosis | ICD-10-CM |
| O34.522 | Maternal care for prolapse of gravid uterus, second trimester | Diagnosis | ICD-10-CM |
| O34.523 | Maternal care for prolapse of gravid uterus, third trimester | Diagnosis | ICD-10-CM |
| O34.529 | Maternal care for prolapse of gravid uterus, unspecified trimester | Diagnosis | ICD-10-CM |
| O34.531 | Maternal care for retroversion of gravid uterus, first trimester | Diagnosis | ICD-10-CM |
| O34.532 | Maternal care for retroversion of gravid uterus, second trimester | Diagnosis | ICD-10-CM |
| O34.533 | Maternal care for retroversion of gravid uterus, third trimester | Diagnosis | ICD-10-CM |
| O34.539 | Maternal care for retroversion of gravid uterus, unspecified trimester | Diagnosis | ICD-10-CM |
| O34.591 | Maternal care for other abnormalities of gravid uterus, first trimester | Diagnosis | ICD-10-CM |
| O34.592 | Maternal care for other abnormalities of gravid uterus, second trimester | Diagnosis | ICD-10-CM |
| O34.593 | Maternal care for other abnormalities of gravid uterus, third trimester | Diagnosis | ICD-10-CM |
| O34.599 | Maternal care for other abnormalities of gravid uterus, unspecified trimester | Diagnosis | ICD-10-CM |
| O34.60 | Maternal care for abnormality of vagina, unspecified trimester | Diagnosis | ICD-10-CM |
| O34.61 | Maternal care for abnormality of vagina, first trimester | Diagnosis | ICD-10-CM |
| O34.62 | Maternal care for abnormality of vagina, second trimester | Diagnosis | ICD-10-CM |
| O34.63 | Maternal care for abnormality of vagina, third trimester | Diagnosis | ICD-10-CM |
| O34.70 | Maternal care for abnormality of vulva and perineum, unspecified trimester | Diagnosis | ICD-10-CM |
| O34.71 | Maternal care for abnormality of vulva and perineum, first trimester | Diagnosis | ICD-10-CM |
| O34.72 | Maternal care for abnormality of vulva and perineum, second trimester | Diagnosis | ICD-10-CM |
| O34.73 | Maternal care for abnormality of vulva and perineum, third trimester | Diagnosis | ICD-10-CM |
| O34.80 | Maternal care for other abnormalities of pelvic organs, unspecified trimester | Diagnosis | ICD-10-CM |
| O34.81 | Maternal care for other abnormalities of pelvic organs, first trimester | Diagnosis | ICD-10-CM |
| O34.82 | Maternal care for other abnormalities of pelvic organs, second trimester | Diagnosis | ICD-10-CM |
| O34.83 | Maternal care for other abnormalities of pelvic organs, third trimester | Diagnosis | ICD-10-CM |
| O34.90 | Maternal care for abnormality of pelvic organ, unspecified, unspecified trimester | Diagnosis | ICD-10-CM |
| O34.91 | Maternal care for abnormality of pelvic organ, unspecified, first trimester | Diagnosis | ICD-10-CM |
| O34.92 | Maternal care for abnormality of pelvic organ, unspecified, second trimester | Diagnosis | ICD-10-CM |
| O34.93 | Maternal care for abnormality of pelvic organ, unspecified, third trimester | Diagnosis | ICD-10-CM |
| O35.0XX0 | Maternal care for (suspected) central nervous system malformation in fetus, not applicable or unspecified | Diagnosis | ICD-10-CM |
| O35.0XX1 | Maternal care for (suspected) central nervous system malformation in fetus, fetus 1 | Diagnosis | ICD-10-CM |
| O35.0XX2 | Maternal care for (suspected) central nervous system malformation in fetus, fetus 2 | Diagnosis | ICD-10-CM |
| O35.0XX3 | Maternal care for (suspected) central nervous system malformation in fetus, fetus 3 | Diagnosis | ICD-10-CM |
| O35.0XX4 | Maternal care for (suspected) central nervous system malformation in fetus, fetus 4 | Diagnosis | ICD-10-CM |
| O35.0XX5 | Maternal care for (suspected) central nervous system malformation in fetus, fetus 5 | Diagnosis | ICD-10-CM |
| O35.0XX9 | Maternal care for (suspected) central nervous system malformation in fetus, other fetus | Diagnosis | ICD-10-CM |
| O35.1XX0 | Maternal care for (suspected) chromosomal abnormality in fetus, not applicable or unspecified | Diagnosis | ICD-10-CM |
| O35.1XX1 | Maternal care for (suspected) chromosomal abnormality in fetus, fetus 1 | Diagnosis | ICD-10-CM |
| O35.1XX2 | Maternal care for (suspected) chromosomal abnormality in fetus, fetus 2 | Diagnosis | ICD-10-CM |
| O35.1XX3 | Maternal care for (suspected) chromosomal abnormality in fetus, fetus 3 | Diagnosis | ICD-10-CM |
| O35.1XX4 | Maternal care for (suspected) chromosomal abnormality in fetus, fetus 4 | Diagnosis | ICD-10-CM |
| O35.1XX5 | Maternal care for (suspected) chromosomal abnormality in fetus, fetus 5 | Diagnosis | ICD-10-CM |
| O35.1XX9 | Maternal care for (suspected) chromosomal abnormality in fetus, other fetus | Diagnosis | ICD-10-CM |
| O35.2XX0 | Maternal care for (suspected) hereditary disease in fetus, not applicable or unspecified | Diagnosis | ICD-10-CM |
| O35.2XX1 | Maternal care for (suspected) hereditary disease in fetus, fetus 1 | Diagnosis | ICD-10-CM |
| O35.2XX2 | Maternal care for (suspected) hereditary disease in fetus, fetus 2 | Diagnosis | ICD-10-CM |
| O35.2XX3 | Maternal care for (suspected) hereditary disease in fetus, fetus 3 | Diagnosis | ICD-10-CM |
| O35.2XX4 | Maternal care for (suspected) hereditary disease in fetus, fetus 4 | Diagnosis | ICD-10-CM |
| O35.2XX5 | Maternal care for (suspected) hereditary disease in fetus, fetus 5 | Diagnosis | ICD-10-CM |
| O35.2XX9 | Maternal care for (suspected) hereditary disease in fetus, other fetus | Diagnosis | ICD-10-CM |
| O35.3XX0 | Maternal care for (suspected) damage to fetus from viral disease in mother, not applicable or unspecified | Diagnosis | ICD-10-CM |
| O35.3XX1 | Maternal care for (suspected) damage to fetus from viral disease in mother, fetus 1 | Diagnosis | ICD-10-CM |
| O35.3XX2 | Maternal care for (suspected) damage to fetus from viral disease in mother, fetus 2 | Diagnosis | ICD-10-CM |
| O35.3XX3 | Maternal care for (suspected) damage to fetus from viral disease in mother, fetus 3 | Diagnosis | ICD-10-CM |
| O35.3XX4 | Maternal care for (suspected) damage to fetus from viral disease in mother, fetus 4 | Diagnosis | ICD-10-CM |
| O35.3XX5 | Maternal care for (suspected) damage to fetus from viral disease in mother, fetus 5 | Diagnosis | ICD-10-CM |
| O35.3XX9 | Maternal care for (suspected) damage to fetus from viral disease in mother, other fetus | Diagnosis | ICD-10-CM |
| O35.4XX0 | Maternal care for (suspected) damage to fetus from alcohol, not applicable or unspecified | Diagnosis | ICD-10-CM |
| O35.4XX1 | Maternal care for (suspected) damage to fetus from alcohol, fetus 1 | Diagnosis | ICD-10-CM |
| O35.4XX2 | Maternal care for (suspected) damage to fetus from alcohol, fetus 2 | Diagnosis | ICD-10-CM |
| O35.4XX3 | Maternal care for (suspected) damage to fetus from alcohol, fetus 3 | Diagnosis | ICD-10-CM |
| O35.4XX4 | Maternal care for (suspected) damage to fetus from alcohol, fetus 4 | Diagnosis | ICD-10-CM |
| O35.4XX5 | Maternal care for (suspected) damage to fetus from alcohol, fetus 5 | Diagnosis | ICD-10-CM |
| O35.4XX9 | Maternal care for (suspected) damage to fetus from alcohol, other fetus | Diagnosis | ICD-10-CM |
| O35.5XX0 | Maternal care for (suspected) damage to fetus by drugs, not applicable or unspecified | Diagnosis | ICD-10-CM |
| O35.5XX1 | Maternal care for (suspected) damage to fetus by drugs, fetus 1 | Diagnosis | ICD-10-CM |
| O35.5XX2 | Maternal care for (suspected) damage to fetus by drugs, fetus 2 | Diagnosis | ICD-10-CM |
| O35.5XX3 | Maternal care for (suspected) damage to fetus by drugs, fetus 3 | Diagnosis | ICD-10-CM |
| O35.5XX4 | Maternal care for (suspected) damage to fetus by drugs, fetus 4 | Diagnosis | ICD-10-CM |
| O35.5XX5 | Maternal care for (suspected) damage to fetus by drugs, fetus 5 | Diagnosis | ICD-10-CM |
| O35.5XX9 | Maternal care for (suspected) damage to fetus by drugs, other fetus | Diagnosis | ICD-10-CM |
| O35.6XX0 | Maternal care for (suspected) damage to fetus by radiation, not applicable or unspecified | Diagnosis | ICD-10-CM |
| O35.6XX1 | Maternal care for (suspected) damage to fetus by radiation, fetus 1 | Diagnosis | ICD-10-CM |
| O35.6XX2 | Maternal care for (suspected) damage to fetus by radiation, fetus 2 | Diagnosis | ICD-10-CM |
| O35.6XX3 | Maternal care for (suspected) damage to fetus by radiation, fetus 3 | Diagnosis | ICD-10-CM |
| O35.6XX4 | Maternal care for (suspected) damage to fetus by radiation, fetus 4 | Diagnosis | ICD-10-CM |
| O35.6XX5 | Maternal care for (suspected) damage to fetus by radiation, fetus 5 | Diagnosis | ICD-10-CM |
| O35.6XX9 | Maternal care for (suspected) damage to fetus by radiation, other fetus | Diagnosis | ICD-10-CM |
| O35.7XX0 | Maternal care for (suspected) damage to fetus by other medical procedures, not applicable or unspecified | Diagnosis | ICD-10-CM |
| O35.7XX1 | Maternal care for (suspected) damage to fetus by other medical procedures, fetus 1 | Diagnosis | ICD-10-CM |
| O35.7XX2 | Maternal care for (suspected) damage to fetus by other medical procedures, fetus 2 | Diagnosis | ICD-10-CM |
| O35.7XX3 | Maternal care for (suspected) damage to fetus by other medical procedures, fetus 3 | Diagnosis | ICD-10-CM |
| O35.7XX4 | Maternal care for (suspected) damage to fetus by other medical procedures, fetus 4 | Diagnosis | ICD-10-CM |
| O35.7XX5 | Maternal care for (suspected) damage to fetus by other medical procedures, fetus 5 | Diagnosis | ICD-10-CM |
| O35.7XX9 | Maternal care for (suspected) damage to fetus by other medical procedures, other fetus | Diagnosis | ICD-10-CM |
| O35.8XX0 | Maternal care for other (suspected) fetal abnormality and damage, not applicable or unspecified | Diagnosis | ICD-10-CM |
| O35.8XX1 | Maternal care for other (suspected) fetal abnormality and damage, fetus 1 | Diagnosis | ICD-10-CM |
| O35.8XX2 | Maternal care for other (suspected) fetal abnormality and damage, fetus 2 | Diagnosis | ICD-10-CM |
| O35.8XX3 | Maternal care for other (suspected) fetal abnormality and damage, fetus 3 | Diagnosis | ICD-10-CM |
| O35.8XX4 | Maternal care for other (suspected) fetal abnormality and damage, fetus 4 | Diagnosis | ICD-10-CM |
| O35.8XX5 | Maternal care for other (suspected) fetal abnormality and damage, fetus 5 | Diagnosis | ICD-10-CM |
| O35.8XX9 | Maternal care for other (suspected) fetal abnormality and damage, other fetus | Diagnosis | ICD-10-CM |
| O35.9XX0 | Maternal care for (suspected) fetal abnormality and damage, unspecified, not applicable or unspecified | Diagnosis | ICD-10-CM |
| O35.9XX1 | Maternal care for (suspected) fetal abnormality and damage, unspecified, fetus 1 | Diagnosis | ICD-10-CM |
| O35.9XX2 | Maternal care for (suspected) fetal abnormality and damage, unspecified, fetus 2 | Diagnosis | ICD-10-CM |
| O35.9XX3 | Maternal care for (suspected) fetal abnormality and damage, unspecified, fetus 3 | Diagnosis | ICD-10-CM |
| O35.9XX4 | Maternal care for (suspected) fetal abnormality and damage, unspecified, fetus 4 | Diagnosis | ICD-10-CM |
| O35.9XX5 | Maternal care for (suspected) fetal abnormality and damage, unspecified, fetus 5 | Diagnosis | ICD-10-CM |
| O35.9XX9 | Maternal care for (suspected) fetal abnormality and damage, unspecified, other fetus | Diagnosis | ICD-10-CM |
| O36.0110 | Maternal care for anti-D [Rh] antibodies, first trimester, not applicable or unspecified | Diagnosis | ICD-10-CM |
| O36.0111 | Maternal care for anti-D [Rh] antibodies, first trimester, fetus 1 | Diagnosis | ICD-10-CM |
| O36.0112 | Maternal care for anti-D [Rh] antibodies, first trimester, fetus 2 | Diagnosis | ICD-10-CM |
| O36.0113 | Maternal care for anti-D [Rh] antibodies, first trimester, fetus 3 | Diagnosis | ICD-10-CM |
| O36.0114 | Maternal care for anti-D [Rh] antibodies, first trimester, fetus 4 | Diagnosis | ICD-10-CM |
| O36.0115 | Maternal care for anti-D [Rh] antibodies, first trimester, fetus 5 | Diagnosis | ICD-10-CM |
| O36.0119 | Maternal care for anti-D [Rh] antibodies, first trimester, other fetus | Diagnosis | ICD-10-CM |
| O36.0120 | Maternal care for anti-D [Rh] antibodies, second trimester, not applicable or unspecified | Diagnosis | ICD-10-CM |
| O36.0121 | Maternal care for anti-D [Rh] antibodies, second trimester, fetus 1 | Diagnosis | ICD-10-CM |
| O36.0122 | Maternal care for anti-D [Rh] antibodies, second trimester, fetus 2 | Diagnosis | ICD-10-CM |
| O36.0123 | Maternal care for anti-D [Rh] antibodies, second trimester, fetus 3 | Diagnosis | ICD-10-CM |
| O36.0124 | Maternal care for anti-D [Rh] antibodies, second trimester, fetus 4 | Diagnosis | ICD-10-CM |
| O36.0125 | Maternal care for anti-D [Rh] antibodies, second trimester, fetus 5 | Diagnosis | ICD-10-CM |
| O36.0129 | Maternal care for anti-D [Rh] antibodies, second trimester, other fetus | Diagnosis | ICD-10-CM |
| O36.0130 | Maternal care for anti-D [Rh] antibodies, third trimester, not applicable or unspecified | Diagnosis | ICD-10-CM |
| O36.0131 | Maternal care for anti-D [Rh] antibodies, third trimester, fetus 1 | Diagnosis | ICD-10-CM |
| O36.0132 | Maternal care for anti-D [Rh] antibodies, third trimester, fetus 2 | Diagnosis | ICD-10-CM |
| O36.0133 | Maternal care for anti-D [Rh] antibodies, third trimester, fetus 3 | Diagnosis | ICD-10-CM |
| O36.0134 | Maternal care for anti-D [Rh] antibodies, third trimester, fetus 4 | Diagnosis | ICD-10-CM |
| O36.0135 | Maternal care for anti-D [Rh] antibodies, third trimester, fetus 5 | Diagnosis | ICD-10-CM |
| O36.0139 | Maternal care for anti-D [Rh] antibodies, third trimester, other fetus | Diagnosis | ICD-10-CM |
| O36.0190 | Maternal care for anti-D [Rh] antibodies, unspecified trimester, not applicable or unspecified | Diagnosis | ICD-10-CM |
| O36.0191 | Maternal care for anti-D [Rh] antibodies, unspecified trimester, fetus 1 | Diagnosis | ICD-10-CM |
| O36.0192 | Maternal care for anti-D [Rh] antibodies, unspecified trimester, fetus 2 | Diagnosis | ICD-10-CM |
| O36.0193 | Maternal care for anti-D [Rh] antibodies, unspecified trimester, fetus 3 | Diagnosis | ICD-10-CM |
| O36.0194 | Maternal care for anti-D [Rh] antibodies, unspecified trimester, fetus 4 | Diagnosis | ICD-10-CM |
| O36.0195 | Maternal care for anti-D [Rh] antibodies, unspecified trimester, fetus 5 | Diagnosis | ICD-10-CM |
| O36.0199 | Maternal care for anti-D [Rh] antibodies, unspecified trimester, other fetus | Diagnosis | ICD-10-CM |
| O36.0910 | Maternal care for other rhesus isoimmunization, first trimester, not applicable or unspecified | Diagnosis | ICD-10-CM |
| O36.0911 | Maternal care for other rhesus isoimmunization, first trimester, fetus 1 | Diagnosis | ICD-10-CM |
| O36.0912 | Maternal care for other rhesus isoimmunization, first trimester, fetus 2 | Diagnosis | ICD-10-CM |
| O36.0913 | Maternal care for other rhesus isoimmunization, first trimester, fetus 3 | Diagnosis | ICD-10-CM |
| O36.0914 | Maternal care for other rhesus isoimmunization, first trimester, fetus 4 | Diagnosis | ICD-10-CM |
| O36.0915 | Maternal care for other rhesus isoimmunization, first trimester, fetus 5 | Diagnosis | ICD-10-CM |
| O36.0919 | Maternal care for other rhesus isoimmunization, first trimester, other fetus | Diagnosis | ICD-10-CM |
| O36.0920 | Maternal care for other rhesus isoimmunization, second trimester, not applicable or unspecified | Diagnosis | ICD-10-CM |
| O36.0921 | Maternal care for other rhesus isoimmunization, second trimester, fetus 1 | Diagnosis | ICD-10-CM |
| O36.0922 | Maternal care for other rhesus isoimmunization, second trimester, fetus 2 | Diagnosis | ICD-10-CM |
| O36.0923 | Maternal care for other rhesus isoimmunization, second trimester, fetus 3 | Diagnosis | ICD-10-CM |
| O36.0924 | Maternal care for other rhesus isoimmunization, second trimester, fetus 4 | Diagnosis | ICD-10-CM |
| O36.0925 | Maternal care for other rhesus isoimmunization, second trimester, fetus 5 | Diagnosis | ICD-10-CM |
| O36.0929 | Maternal care for other rhesus isoimmunization, second trimester, other fetus | Diagnosis | ICD-10-CM |
| O36.0930 | Maternal care for other rhesus isoimmunization, third trimester, not applicable or unspecified | Diagnosis | ICD-10-CM |
| O36.0931 | Maternal care for other rhesus isoimmunization, third trimester, fetus 1 | Diagnosis | ICD-10-CM |
| O36.0932 | Maternal care for other rhesus isoimmunization, third trimester, fetus 2 | Diagnosis | ICD-10-CM |
| O36.0933 | Maternal care for other rhesus isoimmunization, third trimester, fetus 3 | Diagnosis | ICD-10-CM |
| O36.0934 | Maternal care for other rhesus isoimmunization, third trimester, fetus 4 | Diagnosis | ICD-10-CM |
| O36.0935 | Maternal care for other rhesus isoimmunization, third trimester, fetus 5 | Diagnosis | ICD-10-CM |
| O36.0939 | Maternal care for other rhesus isoimmunization, third trimester, other fetus | Diagnosis | ICD-10-CM |
| O36.0990 | Maternal care for other rhesus isoimmunization, unspecified trimester, not applicable or unspecified | Diagnosis | ICD-10-CM |
| O36.0991 | Maternal care for other rhesus isoimmunization, unspecified trimester, fetus 1 | Diagnosis | ICD-10-CM |
| O36.0992 | Maternal care for other rhesus isoimmunization, unspecified trimester, fetus 2 | Diagnosis | ICD-10-CM |
| O36.0993 | Maternal care for other rhesus isoimmunization, unspecified trimester, fetus 3 | Diagnosis | ICD-10-CM |
| O36.0994 | Maternal care for other rhesus isoimmunization, unspecified trimester, fetus 4 | Diagnosis | ICD-10-CM |
| O36.0995 | Maternal care for other rhesus isoimmunization, unspecified trimester, fetus 5 | Diagnosis | ICD-10-CM |
| O36.0999 | Maternal care for other rhesus isoimmunization, unspecified trimester, other fetus | Diagnosis | ICD-10-CM |
| O36.1110 | Maternal care for Anti-A sensitization, first trimester, not applicable or unspecified | Diagnosis | ICD-10-CM |
| O36.1111 | Maternal care for Anti-A sensitization, first trimester, fetus 1 | Diagnosis | ICD-10-CM |
| O36.1112 | Maternal care for Anti-A sensitization, first trimester, fetus 2 | Diagnosis | ICD-10-CM |
| O36.1113 | Maternal care for Anti-A sensitization, first trimester, fetus 3 | Diagnosis | ICD-10-CM |
| O36.1114 | Maternal care for Anti-A sensitization, first trimester, fetus 4 | Diagnosis | ICD-10-CM |
| O36.1115 | Maternal care for Anti-A sensitization, first trimester, fetus 5 | Diagnosis | ICD-10-CM |
| O36.1119 | Maternal care for Anti-A sensitization, first trimester, other fetus | Diagnosis | ICD-10-CM |
| O36.1120 | Maternal care for Anti-A sensitization, second trimester, not applicable or unspecified | Diagnosis | ICD-10-CM |
| O36.1121 | Maternal care for Anti-A sensitization, second trimester, fetus 1 | Diagnosis | ICD-10-CM |
| O36.1122 | Maternal care for Anti-A sensitization, second trimester, fetus 2 | Diagnosis | ICD-10-CM |
| O36.1123 | Maternal care for Anti-A sensitization, second trimester, fetus 3 | Diagnosis | ICD-10-CM |
| O36.1124 | Maternal care for Anti-A sensitization, second trimester, fetus 4 | Diagnosis | ICD-10-CM |
| O36.1125 | Maternal care for Anti-A sensitization, second trimester, fetus 5 | Diagnosis | ICD-10-CM |
| O36.1129 | Maternal care for Anti-A sensitization, second trimester, other fetus | Diagnosis | ICD-10-CM |
| O36.1130 | Maternal care for Anti-A sensitization, third trimester, not applicable or unspecified | Diagnosis | ICD-10-CM |
| O36.1131 | Maternal care for Anti-A sensitization, third trimester, fetus 1 | Diagnosis | ICD-10-CM |
| O36.1132 | Maternal care for Anti-A sensitization, third trimester, fetus 2 | Diagnosis | ICD-10-CM |
| O36.1133 | Maternal care for Anti-A sensitization, third trimester, fetus 3 | Diagnosis | ICD-10-CM |
| O36.1134 | Maternal care for Anti-A sensitization, third trimester, fetus 4 | Diagnosis | ICD-10-CM |
| O36.1135 | Maternal care for Anti-A sensitization, third trimester, fetus 5 | Diagnosis | ICD-10-CM |
| O36.1139 | Maternal care for Anti-A sensitization, third trimester, other fetus | Diagnosis | ICD-10-CM |
| O36.1190 | Maternal care for Anti-A sensitization, unspecified trimester, not applicable or unspecified | Diagnosis | ICD-10-CM |
| O36.1191 | Maternal care for Anti-A sensitization, unspecified trimester, fetus 1 | Diagnosis | ICD-10-CM |
| O36.1192 | Maternal care for Anti-A sensitization, unspecified trimester, fetus 2 | Diagnosis | ICD-10-CM |
| O36.1193 | Maternal care for Anti-A sensitization, unspecified trimester, fetus 3 | Diagnosis | ICD-10-CM |
| O36.1194 | Maternal care for Anti-A sensitization, unspecified trimester, fetus 4 | Diagnosis | ICD-10-CM |
| O36.1195 | Maternal care for Anti-A sensitization, unspecified trimester, fetus 5 | Diagnosis | ICD-10-CM |
| O36.1199 | Maternal care for Anti-A sensitization, unspecified trimester, other fetus | Diagnosis | ICD-10-CM |
| O36.1910 | Maternal care for other isoimmunization, first trimester, not applicable or unspecified | Diagnosis | ICD-10-CM |
| O36.1911 | Maternal care for other isoimmunization, first trimester, fetus 1 | Diagnosis | ICD-10-CM |
| O36.1912 | Maternal care for other isoimmunization, first trimester, fetus 2 | Diagnosis | ICD-10-CM |
| O36.1913 | Maternal care for other isoimmunization, first trimester, fetus 3 | Diagnosis | ICD-10-CM |
| O36.1914 | Maternal care for other isoimmunization, first trimester, fetus 4 | Diagnosis | ICD-10-CM |
| O36.1915 | Maternal care for other isoimmunization, first trimester, fetus 5 | Diagnosis | ICD-10-CM |
| O36.1919 | Maternal care for other isoimmunization, first trimester, other fetus | Diagnosis | ICD-10-CM |
| O36.1920 | Maternal care for other isoimmunization, second trimester, not applicable or unspecified | Diagnosis | ICD-10-CM |
| O36.1921 | Maternal care for other isoimmunization, second trimester, fetus 1 | Diagnosis | ICD-10-CM |
| O36.1922 | Maternal care for other isoimmunization, second trimester, fetus 2 | Diagnosis | ICD-10-CM |
| O36.1923 | Maternal care for other isoimmunization, second trimester, fetus 3 | Diagnosis | ICD-10-CM |
| O36.1924 | Maternal care for other isoimmunization, second trimester, fetus 4 | Diagnosis | ICD-10-CM |
| O36.1925 | Maternal care for other isoimmunization, second trimester, fetus 5 | Diagnosis | ICD-10-CM |
| O36.1929 | Maternal care for other isoimmunization, second trimester, other fetus | Diagnosis | ICD-10-CM |
| O36.1930 | Maternal care for other isoimmunization, third trimester, not applicable or unspecified | Diagnosis | ICD-10-CM |
| O36.1931 | Maternal care for other isoimmunization, third trimester, fetus 1 | Diagnosis | ICD-10-CM |
| O36.1932 | Maternal care for other isoimmunization, third trimester, fetus 2 | Diagnosis | ICD-10-CM |
| O36.1933 | Maternal care for other isoimmunization, third trimester, fetus 3 | Diagnosis | ICD-10-CM |
| O36.1934 | Maternal care for other isoimmunization, third trimester, fetus 4 | Diagnosis | ICD-10-CM |
| O36.1935 | Maternal care for other isoimmunization, third trimester, fetus 5 | Diagnosis | ICD-10-CM |
| O36.1939 | Maternal care for other isoimmunization, third trimester, other fetus | Diagnosis | ICD-10-CM |
| O36.1990 | Maternal care for other isoimmunization, unspecified trimester, not applicable or unspecified | Diagnosis | ICD-10-CM |
| O36.1991 | Maternal care for other isoimmunization, unspecified trimester, fetus 1 | Diagnosis | ICD-10-CM |
| O36.1992 | Maternal care for other isoimmunization, unspecified trimester, fetus 2 | Diagnosis | ICD-10-CM |
| O36.1993 | Maternal care for other isoimmunization, unspecified trimester, fetus 3 | Diagnosis | ICD-10-CM |
| O36.1994 | Maternal care for other isoimmunization, unspecified trimester, fetus 4 | Diagnosis | ICD-10-CM |
| O36.1995 | Maternal care for other isoimmunization, unspecified trimester, fetus 5 | Diagnosis | ICD-10-CM |
| O36.1999 | Maternal care for other isoimmunization, unspecified trimester, other fetus | Diagnosis | ICD-10-CM |
| O36.20X0 | Maternal care for hydrops fetalis, unspecified trimester, not applicable or unspecified | Diagnosis | ICD-10-CM |
| O36.20X1 | Maternal care for hydrops fetalis, unspecified trimester, fetus 1 | Diagnosis | ICD-10-CM |
| O36.20X2 | Maternal care for hydrops fetalis, unspecified trimester, fetus 2 | Diagnosis | ICD-10-CM |
| O36.20X3 | Maternal care for hydrops fetalis, unspecified trimester, fetus 3 | Diagnosis | ICD-10-CM |
| O36.20X4 | Maternal care for hydrops fetalis, unspecified trimester, fetus 4 | Diagnosis | ICD-10-CM |
| O36.20X5 | Maternal care for hydrops fetalis, unspecified trimester, fetus 5 | Diagnosis | ICD-10-CM |
| O36.20X9 | Maternal care for hydrops fetalis, unspecified trimester, other fetus | Diagnosis | ICD-10-CM |
| O36.21X0 | Maternal care for hydrops fetalis, first trimester, not applicable or unspecified | Diagnosis | ICD-10-CM |
| O36.21X1 | Maternal care for hydrops fetalis, first trimester, fetus 1 | Diagnosis | ICD-10-CM |
| O36.21X2 | Maternal care for hydrops fetalis, first trimester, fetus 2 | Diagnosis | ICD-10-CM |
| O36.21X3 | Maternal care for hydrops fetalis, first trimester, fetus 3 | Diagnosis | ICD-10-CM |
| O36.21X4 | Maternal care for hydrops fetalis, first trimester, fetus 4 | Diagnosis | ICD-10-CM |
| O36.21X5 | Maternal care for hydrops fetalis, first trimester, fetus 5 | Diagnosis | ICD-10-CM |
| O36.21X9 | Maternal care for hydrops fetalis, first trimester, other fetus | Diagnosis | ICD-10-CM |
| O36.22X0 | Maternal care for hydrops fetalis, second trimester, not applicable or unspecified | Diagnosis | ICD-10-CM |
| O36.22X1 | Maternal care for hydrops fetalis, second trimester, fetus 1 | Diagnosis | ICD-10-CM |
| O36.22X2 | Maternal care for hydrops fetalis, second trimester, fetus 2 | Diagnosis | ICD-10-CM |
| O36.22X3 | Maternal care for hydrops fetalis, second trimester, fetus 3 | Diagnosis | ICD-10-CM |
| O36.22X4 | Maternal care for hydrops fetalis, second trimester, fetus 4 | Diagnosis | ICD-10-CM |
| O36.22X5 | Maternal care for hydrops fetalis, second trimester, fetus 5 | Diagnosis | ICD-10-CM |
| O36.22X9 | Maternal care for hydrops fetalis, second trimester, other fetus | Diagnosis | ICD-10-CM |
| O36.23X0 | Maternal care for hydrops fetalis, third trimester, not applicable or unspecified | Diagnosis | ICD-10-CM |
| O36.23X1 | Maternal care for hydrops fetalis, third trimester, fetus 1 | Diagnosis | ICD-10-CM |
| O36.23X2 | Maternal care for hydrops fetalis, third trimester, fetus 2 | Diagnosis | ICD-10-CM |
| O36.23X3 | Maternal care for hydrops fetalis, third trimester, fetus 3 | Diagnosis | ICD-10-CM |
| O36.23X4 | Maternal care for hydrops fetalis, third trimester, fetus 4 | Diagnosis | ICD-10-CM |
| O36.23X5 | Maternal care for hydrops fetalis, third trimester, fetus 5 | Diagnosis | ICD-10-CM |
| O36.23X9 | Maternal care for hydrops fetalis, third trimester, other fetus | Diagnosis | ICD-10-CM |
| O36.4XX0 | Maternal care for intrauterine death, not applicable or unspecified | Diagnosis | ICD-10-CM |
| O36.4XX1 | Maternal care for intrauterine death, fetus 1 | Diagnosis | ICD-10-CM |
| O36.4XX2 | Maternal care for intrauterine death, fetus 2 | Diagnosis | ICD-10-CM |
| O36.4XX3 | Maternal care for intrauterine death, fetus 3 | Diagnosis | ICD-10-CM |
| O36.4XX4 | Maternal care for intrauterine death, fetus 4 | Diagnosis | ICD-10-CM |
| O36.4XX5 | Maternal care for intrauterine death, fetus 5 | Diagnosis | ICD-10-CM |
| O36.4XX9 | Maternal care for intrauterine death, other fetus | Diagnosis | ICD-10-CM |
| O36.5110 | Maternal care for known or suspected placental insufficiency, first trimester, not applicable or unspecified | Diagnosis | ICD-10-CM |
| O36.5111 | Maternal care for known or suspected placental insufficiency, first trimester, fetus 1 | Diagnosis | ICD-10-CM |
[truncated: 214,966 more chars]
